# Supplementary material for: Unlocking mitochondrial dysfunction-associated senescence (MiDAS) with NAD+ – A Boolean model of mitochondrial dynamics and cell cycle control
Source: Transl Oncol. 2024 Aug 19;49:102084. doi: 10.1016/j.tranon.2024.102084 (PMC11380032; doi:10.1016/j.tranon.2024.102084)
Supplement: Supplementary file 12 [file mmc12.pdf]

MiDAS: WT (top) vs. SIRT3 KD - WT (bottom)

TS loss promotes MiDAS

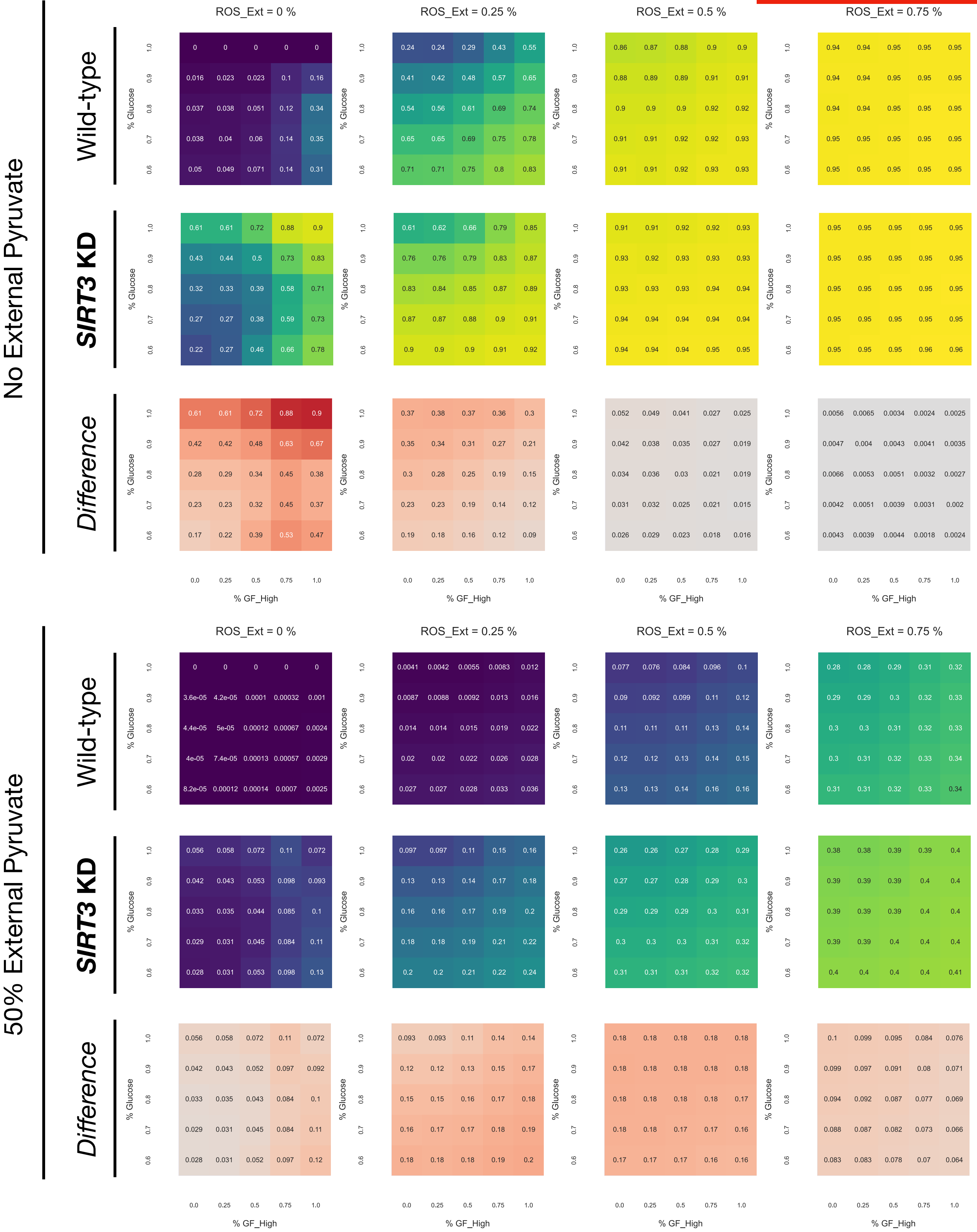

MiDAS: WT (top) vs. pRB KD - WT (bottom)

TS loss promotes MiDAS

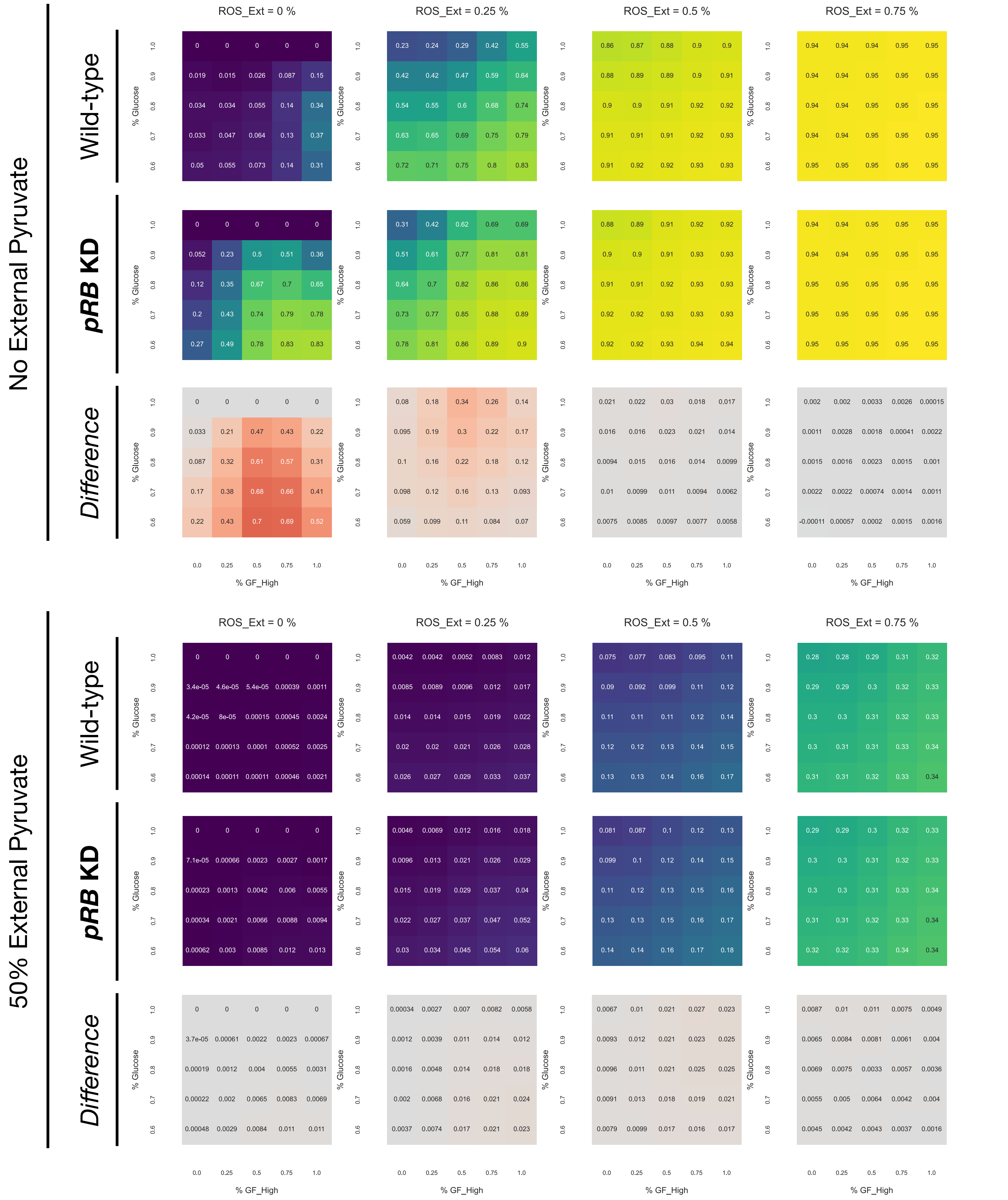

MiDAS: WT (top) vs. CyclinD1 OE - WT (bottom)

oncogene promotes MiDAS

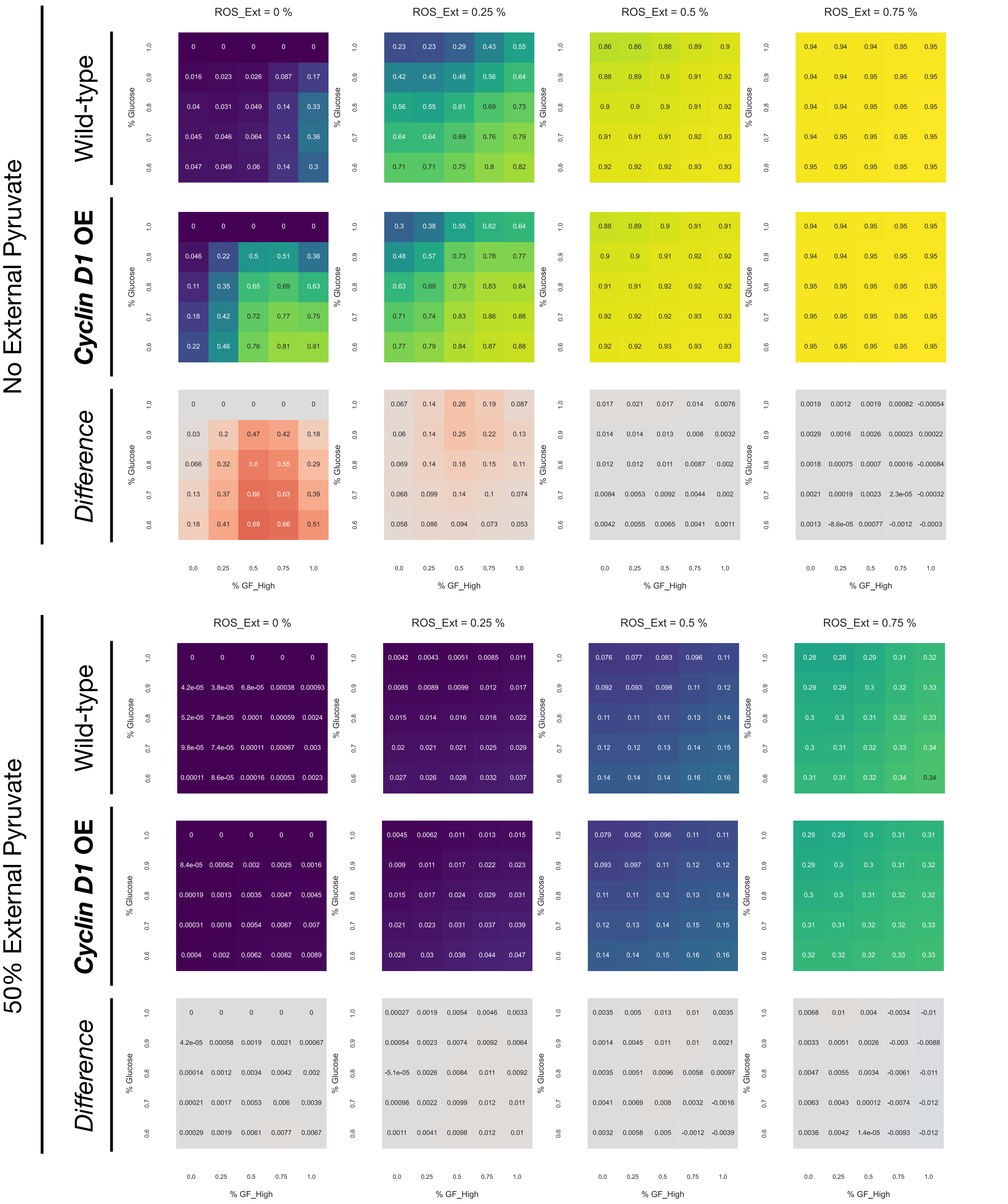

MiDAS: WT (top) vs. p21 KD - WT (bottom)

TS loss promotes MiDAS

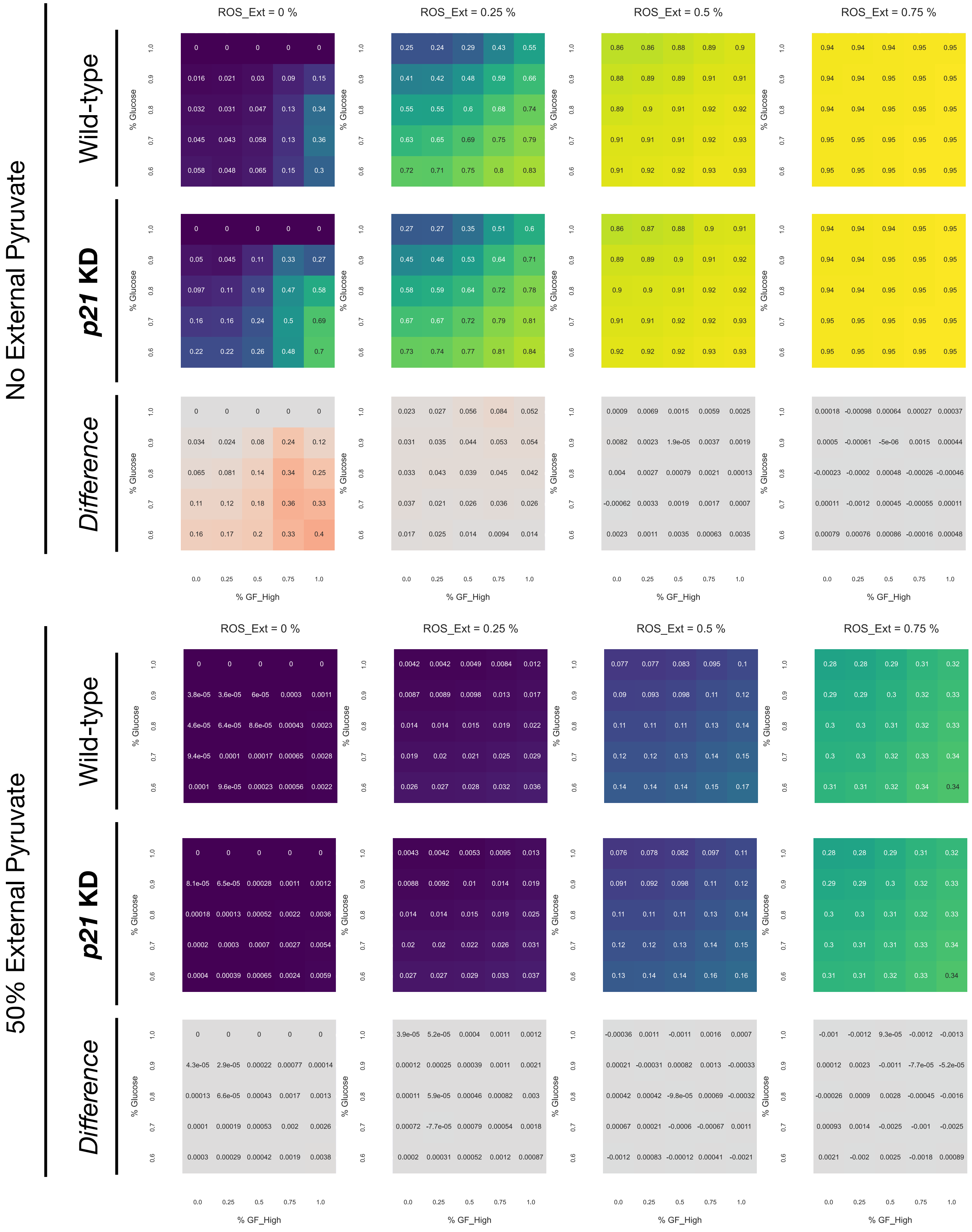

MiDAS: WT (top) vs. Ras OE - WT (bottom)

oncogene promotes MiDAS

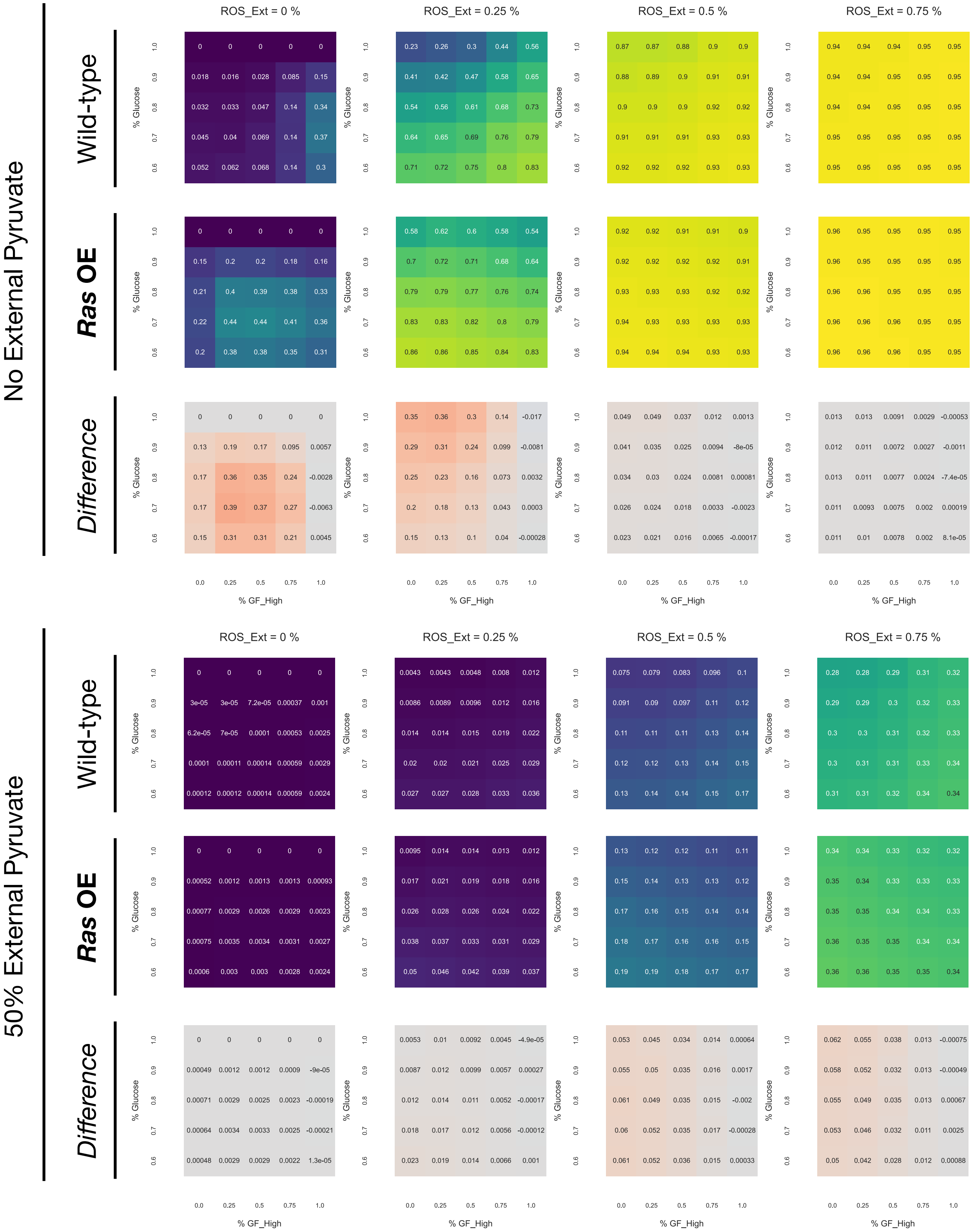

MiDAS: WT (top) vs. AKT_H OE - WT (bottom)

oncogene promotes MiDAS

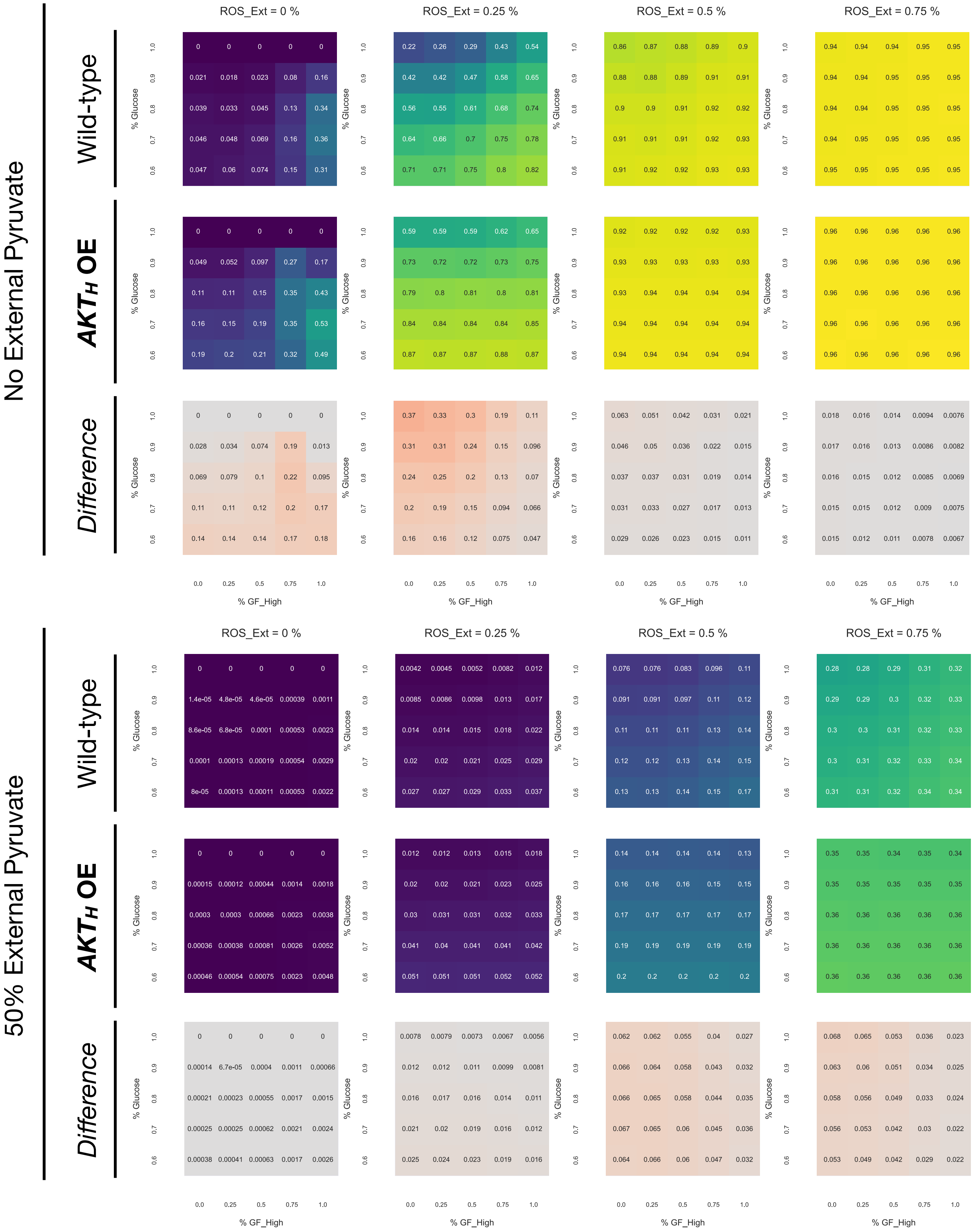

MiDAS: WT (top) vs. FoxO3 KD - WT (bottom)

TS loss promotes MiDAS

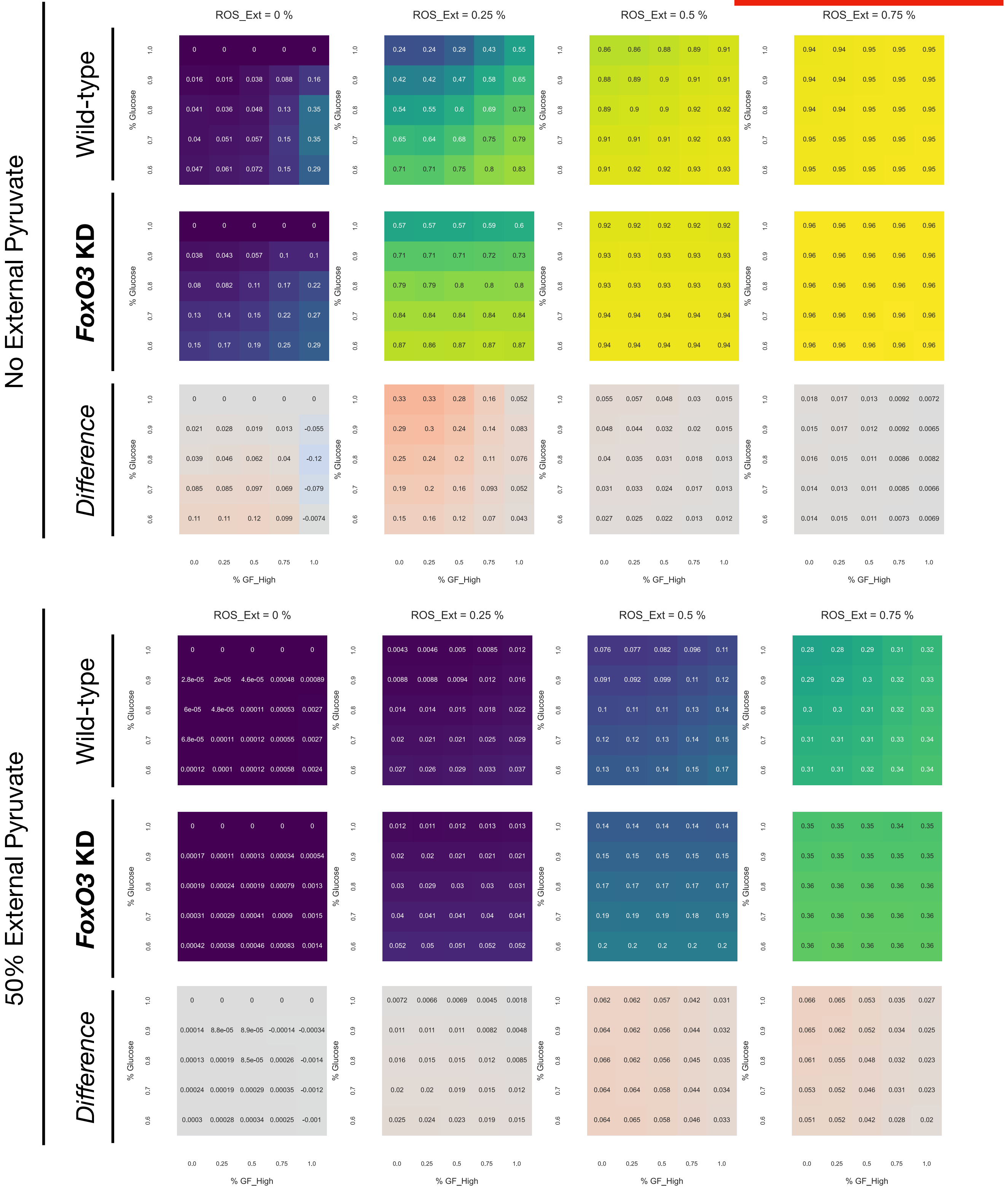

MiDAS: WT (top) vs. p27Kip1 OE - WT (bottom)

TS loss promotes MiDAS

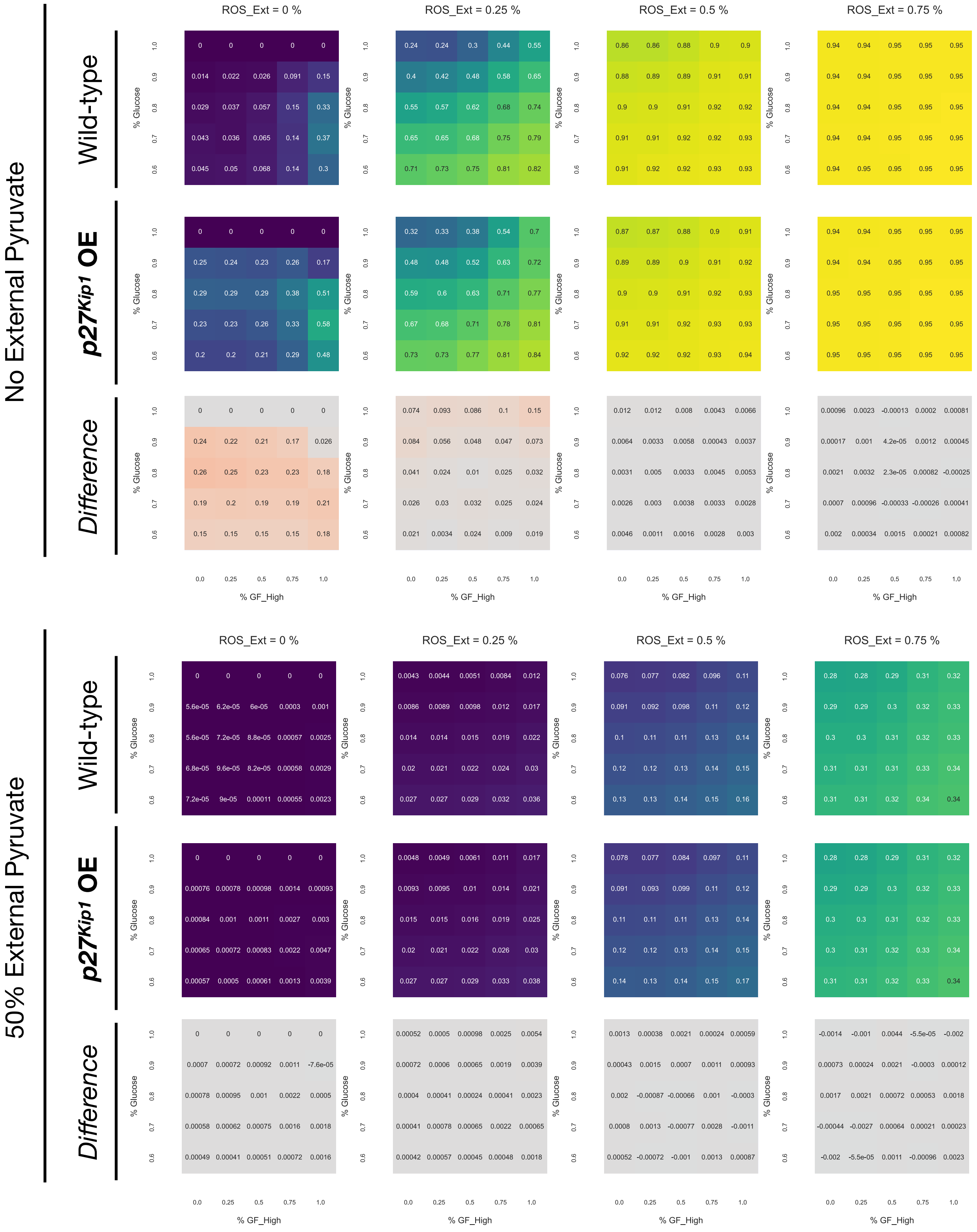

MiDAS: WT (top) vs. Myc OE - WT (bottom)

oncogene promotes MiDAS

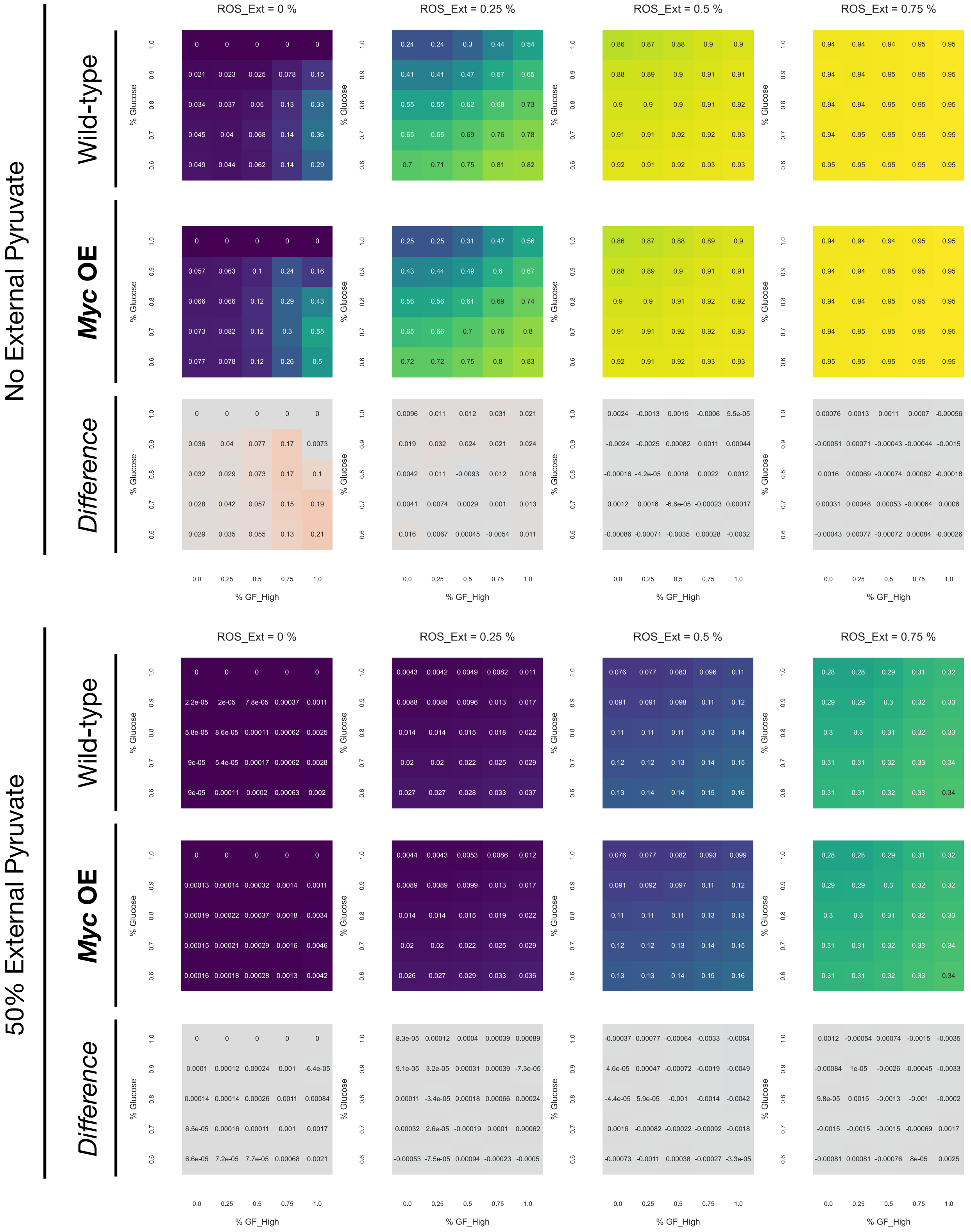

MiDAS: WT (top) vs. mTORC1 OE - WT (bottom)

oncogene promotes MiDAS

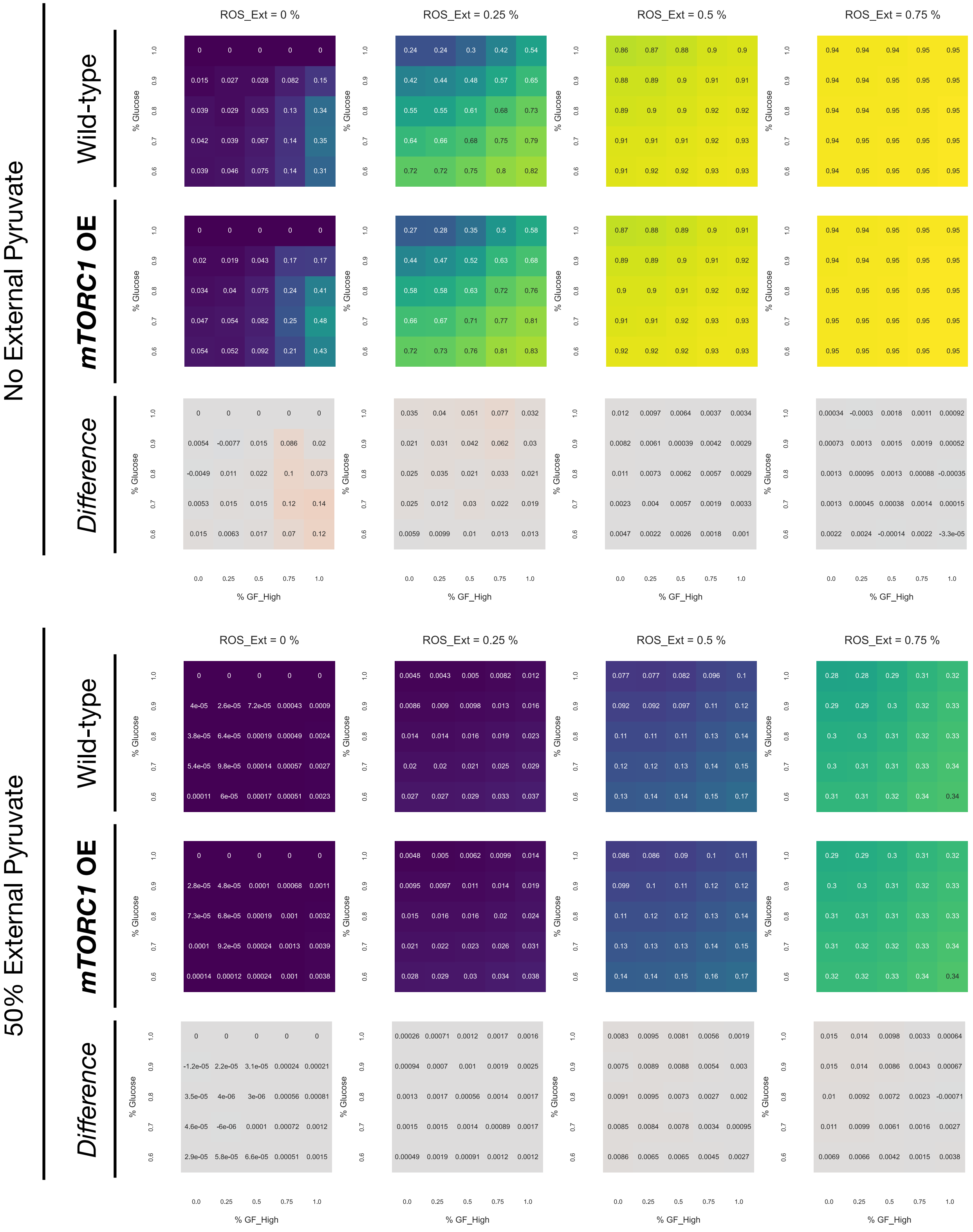

MiDAS: WT (top) vs. FoxO1 KD - WT (bottom)

TS loss promotes MiDAS

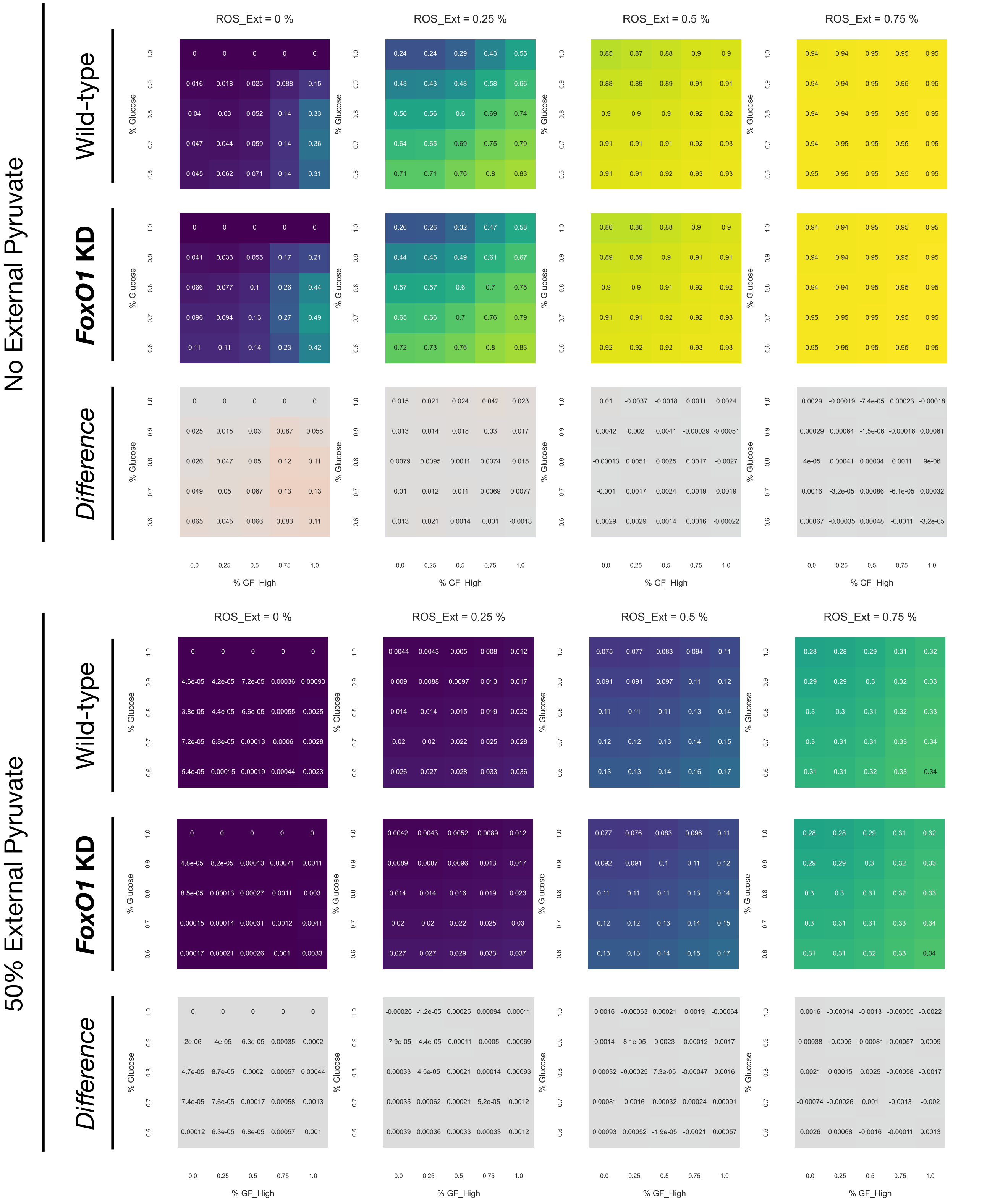

MiDAS: WT (top) vs. p53 KD - WT (bottom)

TS loss blocks MiDAS

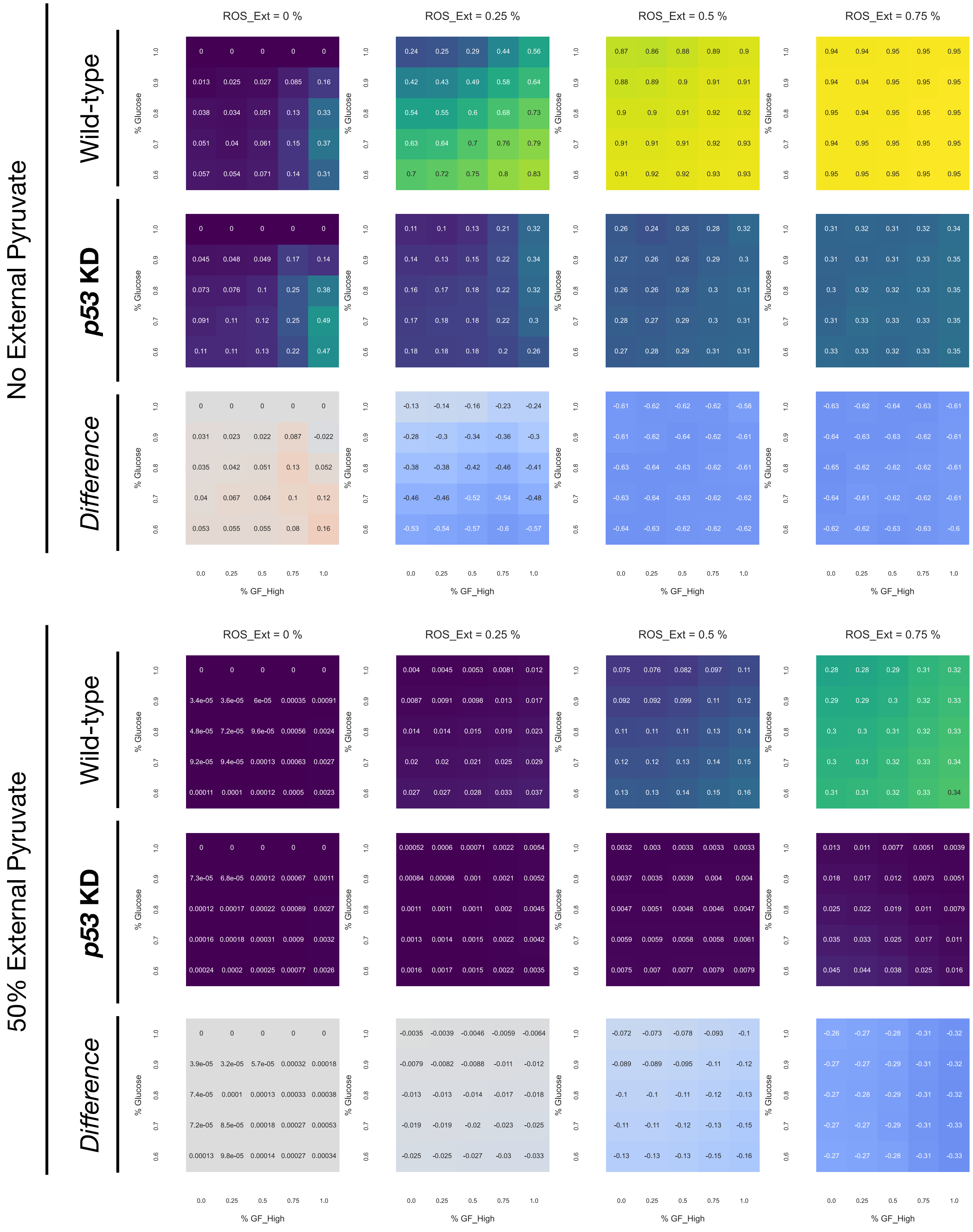

MiDAS: WT (top) vs. p21_H OE - WT (bottom)

oncogene (?) blocks MiDAS

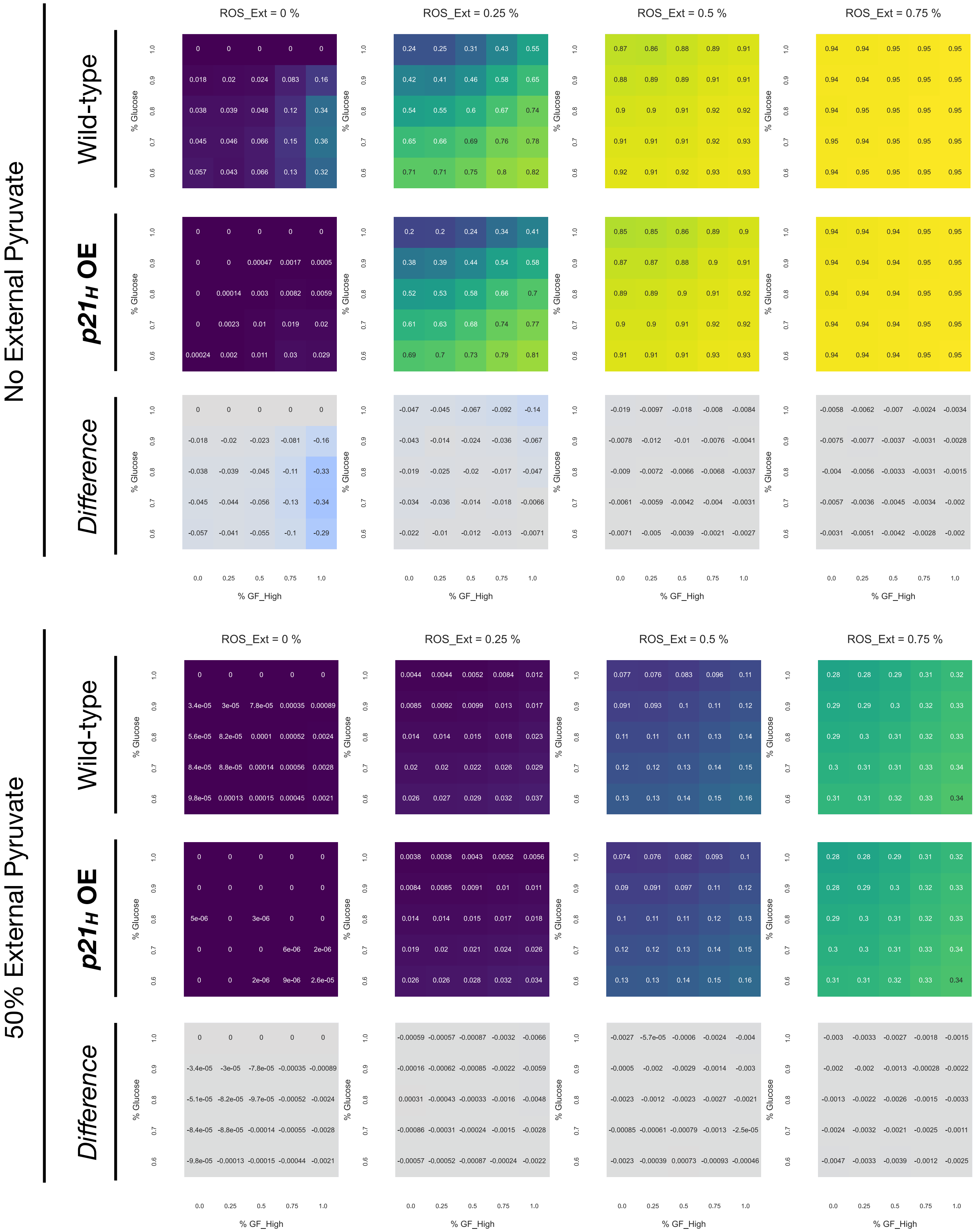

MiDAS: WT (top) vs. CyclinE OE - WT (bottom)

oncogene blocks MiDAS

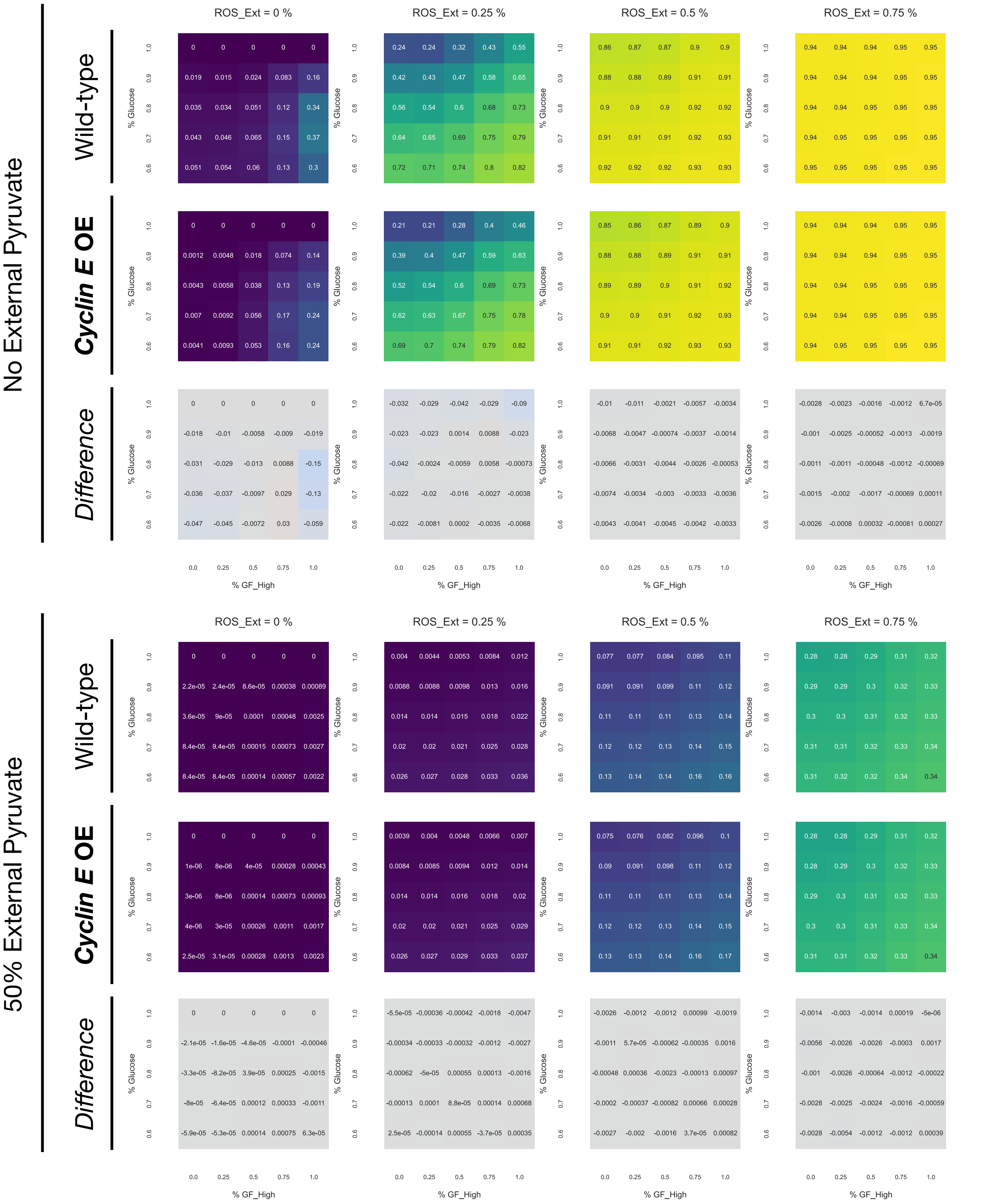

MiDAS: WT (top) vs. PI3K_H OE - WT (bottom)

oncogene does not affect MiDAS

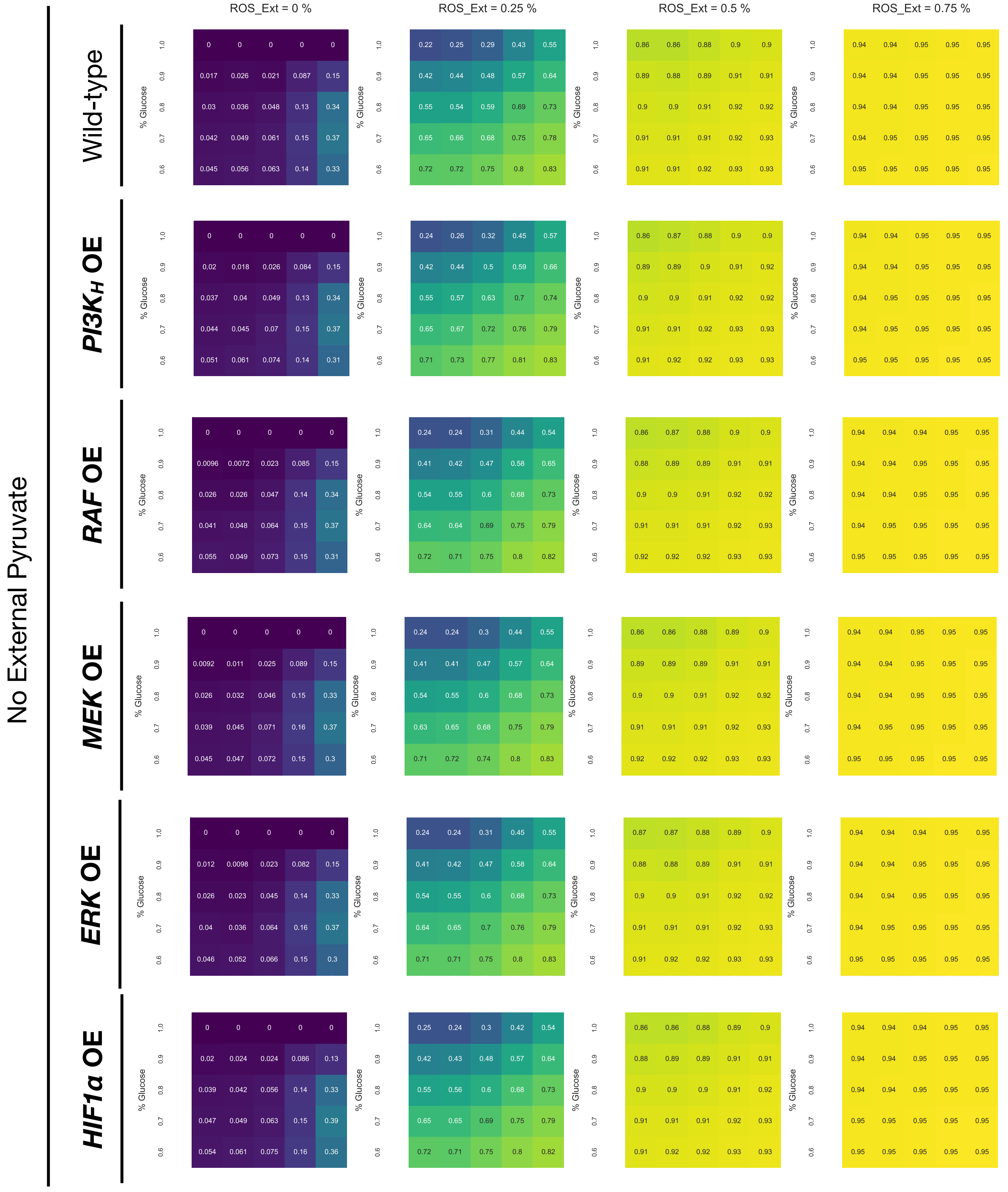

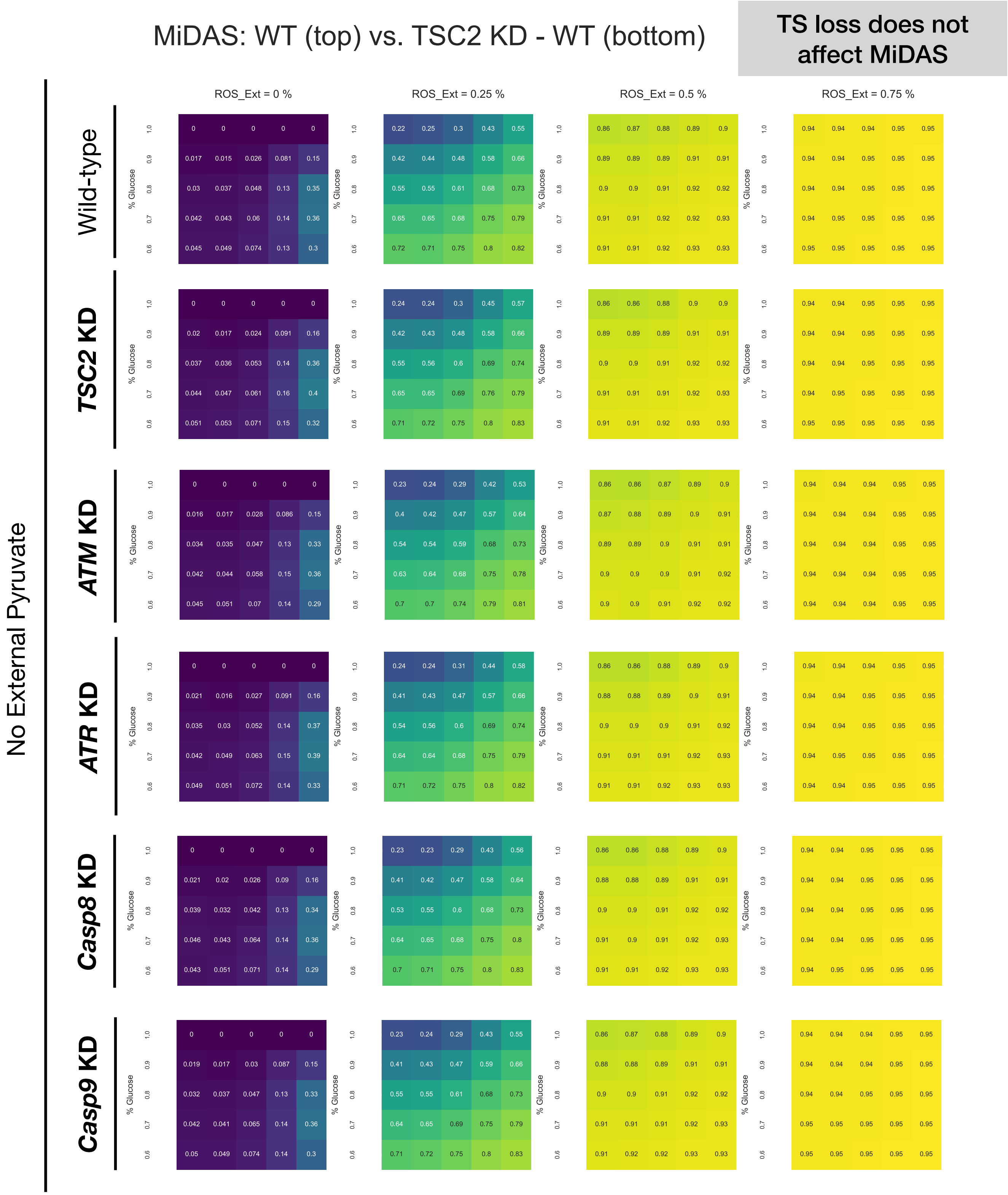
MiDAS: WT (top) vs. TSC2 KD - WT (bottom)
TS loss does not affect MiDAS
No External Pyruvate
ROS_Ext = 0 %
ROS_Ext = 0.25 %
ROS_Ext = 0.5 %
ROS_Ext = 0.75 %
Wild-type
TSC2 KD
ATM KD
ATR KD
Casp8 KD
Casp9 KD
% Glucose
1.0
0.9
0.8
0.7
0.6
