# Supplementary material for: Unlocking mitochondrial dysfunction-associated senescence (MiDAS) with NAD+ – A Boolean model of mitochondrial dynamics and cell cycle control
Source: Transl Oncol. 2024 Aug 19;49:102084. doi: 10.1016/j.tranon.2024.102084 (PMC11380032; doi:10.1016/j.tranon.2024.102084)
Supplement: Supplementary file 14 [file mmc14.pdf]

Supplementary Material

Unlocking Mitochondrial Dysfunction-Associated Senescence (MiDAS) with NAD^+ – a Boolean Model of Mitochondrial Dynamics and Cell Cycle Control

Table of Contents

1. ***Supplementary Methods*** **3**
   a) Advantages of using *dynmod* to model large biological regulatory networks 3
   b) Installing Haskell and *dynmod* 4
   c) Boolean model building in Dynamically Modular Specification (*.dmms*) format 5
      - SM Table 1 — SM Table 1 - MiDAS_Cell_Cycle_Arrests_Apoptosis.pdf
      - SM File 1 — MiDAS_Cell_Cycle_Arrests_Apoptosis.sbml
      - SM File 2 — MiDAS_Cell_Cycle_Arrests_Apoptosis.dmms
      - SM File 3 — MiDAS_Cell_Cycle_Arrests_Apoptosis_Fine.booleannet
      - SM File 4 — MiDAS_Cell_Cycle_Arrests_Apoptosis.gml
      - SM File 5 — Dynamically Modular Model Specification.plist
      - SM File 6 — DMMS_VEX_Language_Notepad++.xml
   d) Synchronous attractor detection with *dynmod* 7
      - SM File 7 — MiDAS_Cell_Cycle_Arrests_Apoptosis_attr_gr20_10_2.0e-2_10.csv
      - SM File 8 — MiDAS_Cell_Cycle_Arrests_Apoptosis_attr_gr20_10_2.0e-2_10.xlsx
      - SM File 9 — Virtual Experiment.plist
      - SM File 10 — MiDAS__Main_Figures.vex
   e) Attractor to cell phenotype mapping, visualization and evaluation of model attractors 8
   f) Running simulations with *dynmod* 9
      - SM File 9 — Virtual Experiment.plist
      - SM File 10 — MiDAS__Main_Figures.vex
      - SM File 11 — MiDAS__SM_Figures.vex
      - SM File 12 — MiDAS__Sizek_Results.vex
2. ***Results, section 2 - Cell Cycle-Dependence of Mitochondrial Morphology*** **12**
   a) Model reproduces experimentally observed mitochondrial dynamics during the cell cycle 12
      - SM Table 2 — summary of model behavior vs. experiments
      - SM Figure 1 — full version of Fig. 3
   b) Model reproduces experimentally observed mitochondrial dynamics under cell cycle perturbations 14
      - SM Table 3 — summary of model behavior vs. experiments
      - SM Figure 2 — phase-dependent effects of Plk1 knockout
      - SM Figure 3 — MFN1/2 knockdown or forced lowering of $\Delta\Psi_M$
      - SM Figure 4 — Drp1 knockdown and/or forced mitochondrial hyperfusion
      - SM Figure 5 — cell cycle arrest and apoptosis with mitochondrial hyperfusion
   c) Model reproduces experimentally observed mitochondrial dynamics under glucose starvation 20
      - SM Table 4 — summary of model behavior vs. experiments
      - SM Figure 6 — endoreduplication or apoptosis prediction following glucose withdrawal
3. ***Results, section 3 - MiDAS*** **22**
      - SM Figure 7 — full version of Fig. 4B (MiDAS in response of SIRT3 loss) and protection in quiescence
      - SM Figure 8 — low ΔΨM-induced MiDAS prevented / rescued by pyruvate
      - SM Figure 9 — external pyruvate protects cells from and can reverse MiDAS
      - SM Figure 10 — sub-lethal MOMP triggers MiDAS
4. ***Results, section 4 - ROS-induced MiDAS*** **27**
      - SM Figure 11 — modeling ROS
      - SM Figure 12 — full version of Fig. 5
      - SM Figure 13 — reversible G2 arrest in brief ROS

- SM Figure 14 — SIRT3 hyper activation protects from / reverses ROS-induced MiDAS

5. ***Results, section 5 - Context-dependence of MiDAS*** **31**
   - SM Figure 15 — model phenotype (attractor) map with all live, diploid phenotypes in all environments
   - SM Figure 16 — model phenotype (attractor) map with all attractors in all environments

6. ***Results, section 6 - MiDAS in cells with cancer-related mutations*** **33**
   - SM Figure 17 — p53 loss blocks MiDAS
   - SM File 13 — MiDAS in cancer-associated mutants.pdf

7. ***Results Reproduced with Biased Asynchronous Update*** **34**
   - SM File 14 — MiDAS__Asynch.vex
   - SM Figure 18 — synchronous limit cycles vs. asynchronous complex attractors
   - SM Figure 19 — asynchronous version of Fig. 3
   - SM Figure 20 — asynchronous version of Fig. 4B, 4D
   - SM Figure 21 — asynchronous version of Fig. 5A-B
   - SM Figure 22 — asynchronous version of Fig. 5C-D

8. ***Model Behavior in Response to Random Network Errors*** **40**
   - SM Figure 23 — cell cycle and MiDAS in ensembles of randomly mutated networks

1. Supplementary Methods

a) Advantages of using dynmod to model large biological regulatory networks

Widely used Boolean modeling software such as GinSim [57], Cell Collective [58], BooleanNet [59], BoolNet [61], CellNetAnalyzer [62] already allow for efficient, probabilistic modeling of regulatory networks representing cell ensembles. Capabilities of these platforms include graphical user interfaces to create, document and simulate models, libraries in common programming languages such as Python or R, synchronous, asynchronous and probabilistic Boolean update options, efficient attractor detection and visualization of the state transition graph. Several of these software packages also handle reconstruction of networks from time series, generation of random network ensembles, robustness analysis via perturbation, or network control [57-59,61,62].

In contrast, our focus in developing *dynmod* was to automate the detection, evaluation and analysis of **biological phenotypes** represented by attractors and/or time-series of our models. While we do replicate some of the functionality common to existing platforms in order to allow *dynmod* autonomy (e.g., attractor detection with synchronous update, synchronous vs. asynchronous time series, or stimulating ensembles), there are a few key reasons for using *dynmod* with a *dmms* model file format (rather than SBML):

a) In the *dmms* model file format, the modeler specify a hierarchy of user-defined signatures attached to regulatory switches; the example in *1a* above shows the Mitochondrial switch. With these profiles *dynmod* can automatically map each attractor to a combinatorial phenotype profile (e.g., quiescent, alive, MiDAS).

b) Using the combinatorial phenotype profile of each attractor, *dynmod* can visualize but also filter attractors of interest. One of its key functions is to organize attractors within a coordinate system of independent environmental input-combinations, and visualize them using barcodes of their combinatorial phenotypes (SM Fig. 16). As not all biological attractors are of equal interest at all times (e.g., robust apoptotic attractors when studying other processes), *dynmod* can filter the list to visualize a user-specified subset (Fig. 6A).

c) As *dynmod* can phenotype arbitrary network states (or sequences to detect oscillations), it can automatically evaluate cell behavior in an arbitrary simulation sequence. To do this, *dynmod* compares the state of the network in each Boolean time-step to phenotypes of interest, and measures the fraction of time particular phenotypes are expressed, as well as the number of times cells undergo specific transitions (e.g., apoptosis or a full cell cycle).

d) As most other software, dynmod can collect statistics on large cell ensembles (independent simulation runs) in non-saturating environments and/or non-saturating perturbations (e.g., 10% Trail, 50% AMPK inhibition). While most software does this for network nodes only, *dynmod* can also detect complex phenotypes such as normal vs. erroneous cell cycle progression.

e) *Dynmod* allows us to set up simulations by specifying the initial environment and cell phenotype-combination (rather than each node's state, or an attractor ID), which allows us to use the same "*in silico* experimental protocol" as we iteratively improve our model (see *.vex* files described below).

f) Much like the *dmms* model file format, several other model formats include node and link metadata. That said, we designed *dmms* metadata fields to be plain text with LaTeX formatting tags, including in-line citations, and wrote a *dynmod* module that transform these metadata fields into a formatted, publication-ready supplementary table containing all biological justification and references used to build the model (SM Table 1).

b) Installing Haskell and dynmod

Note: This font, marks commands to copy-paste into command line or configuration file (e.g., .zshrc file on macOS).

- **macOS**

  i. Check that Terminal is running z-shell; if not change your shell with the command:

  chsh –s /bin/zsh

  ii. Install Xcode developer tools with: xcode-select –install

  iii. Install HomeBrew (simplifies installing libraries required by *dynmod*):

  /bin/bash -c "$(curl -fsSL https://raw.githubusercontent.com/Homebrew/install/HEAD/install.sh)"

  If you already have HomeBrew, run:

  brew update

  brew upgrade

  iv. Add HomeBrew's path to your .zshrc file by including:

  export PATH=/opt/homebrew/bin:$PATH

  export PATH=/opt/homebrew/opt/python/libexec/bin:$PATH

  v. Install the pdf-making packages *dynmod* needs by running:

  brew install cairo pkg-config pango

  iv. Install Haskell by running: curl -sSL https://get.haskellstack.org/ | sh

  v. Specify the path to Haskell and *dynmod* in your .zshrc file and include an update function:

```
# For Haskell Stack:
PATH=~/.local/bin:${PATH}

update_dynmod() {
   rm -rf dynmod
   git clone https://github.com/Ravasz-Regan-Group/dynmod
   cd dynmod
   stack install
}
```

  vi. Install and/or update *dynmod* by navigating to the pace you plan to keep the dynmod source code, then run: update_dynmod ; check your success by running dynmod . If this results in "command not found", you may need to quit and reload terminal. If the issues persist, there are likely errors in your .zshrc file. In this case, run the commands from the update_dynmod() function one at a time.

- **Windows:**

  i. Download "Haskell stack" for windows using Windows Installer from this address: https://docs.haskellstack.org/en/stable/

  ii. To check that stack was installed, write stack -- help into the command line (if the command is found, the output will contain useful command hints):

  iii. Update the MSYS2 package manager pacman, then install required packages:

  pacman: stack exec -- pacman -Syu

  git: stack exec -- pacman -S git

  cairo: stack exec -- pacman -S mingw-w64-x86_64-cairo

  pkg-config: stack exec -- pacman -S mingw-w64-x86_64-pkg-config

  pango: stack exec -- pacman -S mingw-w64-x86_64-pango

v. Specify file paths to these installations[1].

vi. Download *dynmod* from GitHub: stack exec -- git clone https://github.com/Ravasz-Regan-Group/dynmod

vii. Install dynmod:

· Close and restart the command prompt.

· Change directory to the new folder you downloaded dynmod to (cd yourfile_path)

stack install

· Test using the dynmod command. If everything is correctly installed, dynmod will generate a warning for a missing model file followed by use instructions.

c) Boolean model building in Dynamically Modular Specification (.dmms) format

Relevant SM Tables & Files:

- **SM Table 1**, included as *SM Table 1 - MiDAS_Cell_Cycle_Arrests_Apoptosis.pdf*: Large (107-page), formatted and referenced table describing the biological evidence behind each node, link and logic gate of our model, organized by regulatory module.
- **SM File 1**, included as *MiDAS_Cell_Cycle_Arrests_Apoptosis.sbml:* Model file in SBML-qual format used by other Boolean modeling software such as GinSim [PMID: 22144167] or The Cell Collective [PMID: 22871178]; also uploaded to BioModels (MODEL2312140001).
- **SM File 2**, included as *MiDAS_Cell_Cycle_Arrests_Apoptosis.dmms*: Model file in *.dmms* format, containing the model's modular organization (including module and node order), phenotype signatures for relevant regulatory switches or modules, all nodes with logic expression, node description, input link annotation and relevant citations used to auto-generate **SM Table 1**, as well as 2D node coordinates and colors used to generate **SM File 4**.
- **SM File 3**, included as *MiDAS_Cell_Cycle_Arrests_Apoptosis_Fine.booleannet*: Model file in BooleanNet format used by other Boolean modeling software such as BooleanNet [PMID: 19014577] (list of Boolean logic gates with no annotation).
- **SM File 4**, included as *MiDAS_Cell_Cycle_Arrests_Apoptosis.gml:* Visual network layout in *.gml* format used to generate **Fig. 2**, readable by yED (*https://www.yworks.com/products/yed*) or Cytoscape [PMID: 14597658].
- **SM File 5**, included as *Dynamically Modular Model Specification.plist:* Language module file for the BBEdit text editor (Mac OS X), allowing it fold blocks, mark keywords, and recognize comments in *.dmms* files. Upon installing BBEdit, place this file in /Users/*yourusername*/Library/Application Support/BBEdit/Language Modules, then associate this language specification to the *dmms* extension in BBEdit's Language preferences.
- **SM File 6**, included as *DMMS_VEX_Language_Notepad++.xml:* Language module file for the NotePad++ text editor (Windows), allowing it fold blocks, mark keywords, and recognize comments in *.dmms* and *.vex* files. Upon installing NotePad++, import this file from the Language/User Defined Language tab.

---

[1] An easy way to find the path to stack, use: stack uninstall .This will not uninstall stack; merely list all the things you would delete if you were to uninstall it; this includes the directory path.

· Copy the directory containing stack's tools. It should read: C:\Users\USERNAME\AppData\Local\Programs\stack\

· List the contents of this folder to find out the CPU architecture of your machine (e.g., x86_64-windows):

dir C:\Users\USERNAME\AppData\Local\Programs\stack\

· Add the folder name listed by the above command to the path to stack, then list its contents:

dir C:\Users\USERNAME\AppData\Local\Programs\stack\x86_64-windows\

· Add the name of the package manager directory starting with msys to the path, append mingw64\bin\, then list its contents yet again (DATE is the date of your current msys install, listed by the above command):

C:\Users\USERNAME\AppData\Local\Programs\stack\x86_64-windows\msys2-DATE\mingw64\bin\

· Open Settings and search for "environment". Select "edit environment variables for your account".

· Open this, select path, then edit and paste the path you built above into this section.

Our focus in building the *dmms* file structure and *dynmod* was to generate an easily human-editable, but also machine readable file format specific to discrete-state models (SBML-qual is a more general framework, but not friendly to direct editing without a Graphical User Interface). **SM File 2**, with the filename *MiDAS_Cell_Cycle_Arrests_Apoptosis.dmms (*do not rename as it must match internal model name) contains our model in *.dmms* format. This file keeps track of all model-relevant data and metadata, such as our model's hierarchically modular structure, phenotype profiles for regulatory switches, experimental data justifying each node, link and gate, and node visualization details. The top-level structure of this file is organized as follows:

```
Model{
ModelMetaData{ … }
ModelMapping{// organizes molecules of the embedded model into modules or regulatory switches
    Switch: Restriction_SW (Myc, CyclinD1, E2F1, CyclinE, p27Kip1, pRB, p21_B)
    ….
ModelMapping}
SwitchProfiles{ // specifies molecular activity/expression signatures of modules that determine the cell's phenotype / behavior
SwitchPhenotypes{
    SwitchName: Mitochondria
        Fragmented:0 *= (MFN1_2:0, Hyperfused_OM:0, Drp1:1, Unfused:1)
        Normal_Mito:1 *= (TCA_cycle:1, MFN1_2:0, Hyperfused_OM:0, MP_Low:0, AMPK:0, Drp1:0, Unfused:0, mROS:0,
NADp_m:1)
        MiDAS:2 *= (TCA_cycle:0, MFN1_2:1, Hyperfused_OM:1, MP_Low:1, AMPK:1, Drp1:0, Unfused:0, mROS:1, NADp_m:0)
SwitchPhenotypes}
SwitchProfiles}
ModelGraph{ // consists of a Node{ …} block for each regulatory switch; allows modeling the network at switch level}
Model{ // consists of a Node{ …} block for each molecule in the Boolean (or discrete-state) model
Node{
    NodeMetaData{…}
    NodeGate{
      DiscreteLogic{
            Myc *= GF or E2F1
      DiscreteLogic}
    NodeGate}
    InLink{ … }
Model} // closes the molecular model inside the outer one involving regulatory modules or switches
Model} // closes the switch-level model
CitationDictionary{ … } // references in BibTeX format, exported from a citation manager such as Zotero
```

For editing *.dmms* files on Mac OX / Windows, we recommend *BBEdit* / *Notepad++* with DMMS language modules included as **SM File 4** (copy to *~/Library/Application Support/BBEdit/ Language Modules*) and **SM File 5** (import under the *Language/User Defined Language* tab).

To parse and validate the *dmms* file using *dynmod*, run:

```
dynmod MiDAS_Cell_Cycle_Arrests_Apoptosis.dmms
```

Key export functions include:

-t to export the molecular-scale model to **.BooleanNet** format (simple list of logic gates; **SM File 3**):

```
dynmod -t MiDAS_Cell_Cycle_Arrests_Apoptosis.dmms
```

-g to export to **.gml** format (for network visualization; **SM File 4**):

dynmod **-t** MiDAS_Cell_Cycle_Arrests_Apoptosis.dmms

-u to update coordinates read from user-organized *.gml* and appended to metadata in *dmms* files (note that this requires NOT altering the network structure or hierarchy while organizing the *.gml* file):

dynmod **-u** MiDAS_Cell_Cycle_Arrests_Apoptosis.glm MiDAS_Cell_Cycle_Arrests_Apoptosis.dmms

-s to generate a formatted LaTeX document & references. A LaTeX editor then generates **SM Table S1**:

dynmod **-s** MiDAS_Cell_Cycle_Arrests_Apoptosis.dmms

To generate a SBML-qual format of the model (**SM File 1**, *MiDAS_Cell_Cycle_Arrests_Apoptosis.sbml*), install *bioLQM* [PMID: 30510517] (download the jar file associated to the latest release), and use Java 8 Runtime to run it. *BioLQM* can performs format conversions, including from a *.Booleannet* format (generated with *dynmod*) to SBML:

java -jar bioLQM-version.jar modelname.Booleannet modelname.sbml

d) Synchronous attractor detection with dynmod

Relevant SM Files:

- **SM File 7**, included as *MiDAS_Cell_Cycle_Arrests_Apoptosis_attr_gr20_10_2.0e-2_10.csv*: Table containing all attractors detected by *dynmod* across a grid of sampling runs with an increasing number of random seeds and time-course lengths to test for saturation ($N_{rnd} \in \{10, 20, 35, \ldots, 200\}$ random initial conditions in each unique environment, $N_{series} \in \{10, 20, 35, \ldots, 100\}$ noisy steps / initial condition, and $p_{noise} = 0.02$).
- **SM File 8**, included as *MiDAS_Cell_Cycle_Arrests_Apoptosis_attr_gr20_10_2.0e-2_10.xlsx*: Formatted Excel version of SM File 7, containing all synchronous attractors detected by *dynmod,* where ON/OFF states are marked yellow/blue, rows containing environmental input nodes are gray, and headers mark each attractor by their global phenotype.
- **SM File 9**, included as *Virtual Experiment.plist*: Language module file for the BBEdit text editor (Mac OS X), allowing it fold blocks, mark keywords, and recognize comments in *.vex* files. Upon installing BBEdit, place this file in /Users/*yourusername*/Library/Application Support/BBEdit/Language Modules, then associate this language specification to the *vex* extension in BBEdit's Language preferences.
- **SM File 10**, included as *MiDAS__Main_Figures.vex*: Experiment file specifying all simulation *dynmod* ran to generate main figures for the paper, with annotated comments on how to use the *.vex* file format. File can be used to reproduce all main time-course figures by running dynmod -e MiDAS__Main_Figures.vex MiDAS_Cell_Cycle_Arrests_Apoptosis.dmms.

Routine sampling. Dynmod has two sampling modes. The first, quicker mode involves a single attempt at finding attractors by running N noisy time-courses of length T with noise p from different random initial conditions for each unique combination of environmental inputs (e.g., N=200, T=20, p=0.02) [56,65,66]. In each noisy time-step, each node gives an erroneous output with probability p, allowing the dynamics to deviate from steepest descent. As *dynmod* does this, it also checks which synchronous attractor basin each state along the noisy trajectory belongs to. Once this is finished, *dymod* runs a synchronous time course from each attractor state by changing one environmental variable at a time. This increases its ability to detect attractors representing biological phenotypes that are robust in one environment (large attractor basin, easy to detect), and remain stable as the environment changes but their basin shrinks. All detected attractors are exported to a *.csv* file *dynmod* can read and reuse (instead of resampling). We use this mode as we iteratively improve our models, and we specify its parameters in the experiment (.vex) files we also use to run simulations. Details on setting up this sampling can be found at the top of **SM File 9**, *MiDAS__Main_Figures.vex.*

Testing the convergence of sampling. The second, slower sampling mode repeats the above procedure multiple times, with increasing N and T. This can test the convergence of the number of attractors detected with increasing N & T, while also collecting all unique attractors from these separate sampling runs. Here we used a 20 by 10 grid with N ∈ {10, 20, 30, . . , 200}, T ∈ {10, 20, 30, . . , 100}, and p = 0.02, and the command-line tag **-- grid**:

```
dynmod --grid '20 10 0.02 10' MiDAS_Cell_Cycle_Arrests_Apoptosis.dmms
```

(the last number in --grid '20 10 0.02 10' is the gap between increasing N and T values). The outputs of this command are a heat-map of the number of attractors in each independent sampling run along the grid (indicating that most attempts find 96 attractors; not shown), and the full attractor list collated into a single *.csv* (**SM File 7**). We use this mode when our models are largely finalized, to perform a final, extensive synchronous attractor search.

Update dependence of attractors. The fixed point attractors detected by synchronous update are solutions of the Boolean gate equations and thus stable regardless of update. In contrast, synchronous limit cycles do not always correspond to unique complex attractors, and there can be complex attractors with no synchronous limit cycle to match. To test whether *dynmod* detected all fixed points and probe whether its limit cycles are robust to update, we also ran an exhaustive asynchronous attractor search using AEON [63] (see *Suppl. Mat. 7* for comparison).

e) Attractor to cell phenotype mapping, visualization and evaluation of model attractors

Attractor to cell phenotype mapping. The idea that each attractor of a well-built biological network model represents a unique stable cell phenotype in a given environment is conceptually elegant, but difficult to achieve while building large models; especially in projects that focus on the context-dependent responses of large networks in 5 or more independent environments [56]. For models that involve irreversible apoptosis [45,56,66], this guarantees a minimum of 32 apoptotic attractors (doubled by each additional Boolean input). Thus, even a well-designed model with no non-biological attractor can have 50-100 or more attractors ([56] has ~ 480). Early stages of model-building often generate many more.

To help us with this problem, we designed *dynmod* in tandem with the *.dmms* model format to automatically evaluate the phenotype of each attractor in a principled, semi-automatic way. It does this by using the hierarchically modular structure of *dmms* files, paired with user-defined '*SwitchPhenotypes*' (*Suppl. Methods 1a*). These blocks allow the user to define and name specific signatures (sub-spaces) of regulatory modules of interest. For example, the Apoptosis label is associated with (Casp3:1, Casp9:1), one of two mutually exclusive signatures of the apoptotic switch (dead / alive). *Dynmod* uses this structured metadata to assign a global, combinatorial phenotype to each attractor, such as 'quiescent, alive, MiDAS'.

Visualizing attractors by external input and phenotype. Next, *dynmod* organizes all model attractors in a 1 to 5 dimensional environment-coordinate space (inputs above 5, if any, are selected and locked by user) and visualizes each attractor using a barcode of its modular phenotype (see **Fig. 6A**). With all attractors within an environment grouped and translated into cell behavior, iteratively evaluating whether a large model generates experimental documented states across multiple environments is faster and less error-prone than examining attractors one figure or table-column at a time. It is also a quick way to probe the model for biologically relevant multi-stability, such as the coexistence of healthy and MiDAS cells in the same environment (presumably due to their different history). In addition, *dynmod* can filter the list of visualized attractors by excluding/including a user-selected list of phenotypes (e.g., no apoptotic states), and can restrict the

environmental inputs to those of primary interest (e.g., not showing Trail = 1 once we have checked that it always forces apoptosis). Methods to do this are detailed below.

f) Running simulations

Relevant SM File:

- **SM File 9**, included as *Virtual Experiment.plist*: Language module file for the BBEdit text editor (Mac OS X), allowing it fold blocks, mark keywords, and recognize comments in *.vex* files. Upon installing BBEdit, place this file in /Users/*yourusername*/Library/Application Support/BBEdit/Language Modules, then associate this language specification to the *vex* extension in BBEdit's Language preferences.
- **SM File 10**, included as *MiDAS__Main_Figures.vex*: Experiment file specifying all simulation *dynmod* ran to generate main figures for the paper, with annotated comments on how to use the *.vex* file format. File can be used to reproduce all main time-course figures by running dynmod -e MiDAS__Main_Figures.vex MiDAS_Cell_Cycle_Arrests_Apoptosis.dmms.
- **SM File 11**, included as *MiDAS__SM_Figures.vex*: Experiment file specifying all simulation *dynmod* ran to generate supplemental figures of the paper, with annotated comments on how to use the *.vex* file format. File can be used to reproduce all supplemental time-courses via dynmod -e MiDAS__SM_Figures.vex MiDAS_Cell_Cycle_Arrests_Apoptosis.dmms.
- **SM File 12**, included as *MiDAS__Sizek_Results.vex*: Experiment file specifying all single time-course simulation by which *dynmod* ran reproduce previpsuyl published cell behaviors our model inherited from its predecessor in [PMID: 30875364]. File can be used to reproduce all supplemental time-courses via dynmod -e MiDAS__Sizek_Results.vex MiDAS_Cell_Cycle_Arrests_Apoptosis.dmms.
- *Jupyter notebook in Python* to reproduce main time-course figures with BooleanNet [59]: LINK

Setting up in silico experiments in .vex files. To use dynmod for simulations, we designed an "in silico experiments" file format (*.vex* extension). Much like dynmod, this human-editable but machine readable file has a hierarchical organization, with blocks that specify attractor detection or reuse, as well as a series of experiment types. For editing *.vex* files on Mac OX / Windows, we recommend BBEdit / Notepad++ with DMMS language modules included as **SM File 9** and **SM File 6** (same as *.dmms*). Detailed instructions on how to set up each block are included in the *.vex* files that reproduce all our results (main figure results in **SM File 10**; supplementary figure results in **SM File 11**; additional simulations mentioned in Table 2 but not shown in **SM File 12**). Key *.vex* command blocks include:

• ***Sampling.*** This block either instructs *dynmod* to perform a new sampling, or to use of previously generated attractors from a specified *.csv* file (these attractors are verified to assure they match the model). This block also provided options to only sample a subspace of the model's full environment-space to speed iterative model constitution, and expand iteratively with a read-and-sample option.

• ***InputSpaceDiagram.*** This block generates the attractor visualization by phenotype barcodes (specified in the model *.dmms* file under *SwitchProfiles*) and environment-combinations (see *1e* above).

• ***Initial conditions for all experiment blocks.*** Dynmod's attractor profiling allows us to set up simulations by specifying the initial environment along with a desired starting phenotype-combination, rather than each individual node's state. This setup is robust to model tweaks during development, and automatically sets up runs starting from each unique state of a limit cycle attractor, simulating the arrival of a signal at different stages along a cycle (for details, see comments explaining the precise setup in **SM Files 10-12**).

• ***Time course experiment types.***

— *Pulse1* block generates a synchronous time course with three intervals: a) initial cell state(s), b) response to a change in a single environmental signal of user-specified duration (including non-saturating signals such as *GF_High* = 0.6); and c) response to the reversal of the same signal. To

simulate non-saturating inputs, *dynmod* overrides their value with a stochastic ON/OFF sequence with a fixed probability, tuning the cell's average exposure. In case the initial state is a limit cycle, it runs multiple time courses to show the response to the environment change at all points along the cycle.

— *KDOE* block generates a synchronous time courses with two intervals: a) initial cell state(s), b) response the simultaneous partial or full knockdown/hyper-activation of a set of internal nodes. Partial knockdown/ hyper-activation of a node involves forcing it OFF/ON with a fixed probability in each time-step; otherwise allowing it to obey its Boolean rule [45,56,65,66] (justification / limitations of approach detailed in [65,66]). In case the initial state is a limit cycle, it runs multiple time courses to show the response to the environment change at all points along the cycle.

— *GeneralExperiment* block generates synchronous or asynchronous time courses from a subset of cell states in a given initial environment, exposed to an arbitrary sequence of manipulations defined in distinct time windows. Within each window the cell's external environment may be set to an arbitrary non-saturating value-combination (e.g., *GF_High* = 0.5 & *Glucose* = 0.3). In addition, a subset of internal nodes can be partially or fully knocked down / hyper-activated.

• ***Output from time course experiments.*** All types of time-course experiments run a single time-course by default, and show the time-dependent activity of each node (organized by modules) as a function of time (e.g., Fig. 3). Optional settings also include:

• averaging node activity in each tilmestep across an ensemble of cells (multiple simulations specified with a *SampeSize* parameter), resulting in an averaged time course (e.g., SM Fig. 18A)

• not exporting a node-level time course figure (*NodeTimeCourse*: False)

• exporting a time course at the switch phenotypes level, showing whether the model's state matching each phenotype at each time-point (can be averaged across an ensemble; e.g., SM Fig. 18B)

• averaging the activity of a user-specified subset of nodes within each distinct time-interval, resulting in a bar chart of activity for each interval (*AvgBarChartNodes;* e.g., SM Fig. 18C).

• ***Sampling the model's behavior with increasing input and/or knockout/hyper-activation*** *(coming to* dynmod *soon; SM Files 10-11, 15 offer ways to use time-course outputs to generate similar data).* In order to get a sense of how a large ensemble of cells behaves across a range of non-saturating environments and/or node knockdown/hyper-activation treatments, we designed a series of sampling runs that evaluate a cell ensemble across the desired range of conditions by calculating:

• the average time cells spend in any static phenotype of interest (e.g., MiDAS)

• how many times cells complete a biological cycle (e.g., cell cycle, normalized to the length of the wild-type cell cycle)

• how often cells complete a cycle with a biologically relevant error (e.g., resetting to G1 from G2; premature anaphase, skipped cytokinesis)

• and the average time cells take to undergo a particular, typically irreversible transition (e.g., apoptosis).

The intuitive way to set up the ensemble is to start with N_{sample} cells in the same initial environment and biological state (attractor), and simulate their dynamics with the desired environment / knockdown/ hyper-activation for T_{max} update steps. The problem with this is that if our cells undergo apoptosis during this time-course, then the data we get on other phenotypes following these events is no longer relevant to real cell behavior (e.g., a cell might die in MiDAS but after death its mitochondria are

fragmented, so the length of our simulation post apoptosis could heavily influence our pre-death results). To avoid this, we actually set up our ensemble as follows:

- We specify a STOP condition at which we stop tracking the dynamics of individual cells; the first time a simulated cell's state matches one any phenotype on the STOP list (e.g., `Apoptotic_SW:Apoptosis`, or `Apoptotic_SW:Apoptosis, Mitochondria:MiDAS`). Note: these STOP phenotypes may *not* be limit cycles.
- We specify the maximum simulation time for cells that do not reach the STOP condition (e.g., T_{max} = 250 update steps used here; corresponds to 10 wild-type cell cycle lengths).
- We specify a total sampled time across the ensemble, such as T_{total} = 500,000 update steps, rather than the size of the ensemble. This guarantees that N_{sample} is at least T_{total}/T_{max} = 2,000 cells, or more if they tend to reach the STOP condition faster (the rationale is that in this case we need a better sampling of their briefer pre-stop dynamics).
- In addition to the scanned environment and/or environment / knockdown/ hyper-activation (simulated for a range of values), we can also specify a background environment (e.g., scan the model's behavior across all *ROS_ext* values, but do so in 10% external pyruvate), and/or a background mutation (e.g., do the above scan in cells heterozygous for p53: 50% knockdown).

Using this shared setup, we ran a series of sampling simulations of increasing combinatorial complexity, including (note, we used our older code to do this; see [56]; *dynmod* update for scans coming to GitHub soon):

- 1D scan along a singe input (e.g., cell cycle rate as a function of *GF_High*)
- 1D scan with increasing knockdown/over-expression of one (or more) molecule(s), as shown on Fig. 4D (when more than one molecule is specified for the scan, their levels of knockdown move in lockstep; this works well to simulate broader-target inhibitors that target more than 1 molecule in the model).
- 2D scan across two inputs
- 2D scan across an input and a knockdown/over-expression
- 3D scan across three inputs
- 3D scan across two inputs and a knockdown/over-expression
- comparative 3D scan across three inputs, computing the difference between wild-type cells and a specific knockdown/over-expression (e.g., Fig. 6B)

Simulations with BooleanNet. For readers who wish to use BooleanNet [59] to reproduce our main findings, please see the Jupyter notebook in Python at *https://github.com/deriteidavid/midas_boolean_model*. This reproduces the synchronous time-course simulations included in our main figures (Figs. 3, 4B, 5).
