# Supplementary material for: Unlocking mitochondrial dysfunction-associated senescence (MiDAS) with NAD+ – A Boolean model of mitochondrial dynamics and cell cycle control
Source: Transl Oncol. 2024 Aug 19;49:102084. doi: 10.1016/j.tranon.2024.102084 (PMC11380032; doi:10.1016/j.tranon.2024.102084)
Supplement: Supplementary file 15 [file mmc15.pdf]

S1 Description & experimental support for the modules of MiDAS_Cell_Cycle_Arrests_Apoptosis_Fine.

Table S1a: Growth_Env module

Target Node	Node Gate<br>Node Type<br>Link Type	Node Description<br>Input Node	Link Description
GF	**GF** = **GF** or **GF_High**		
	Env	The *GF* node represents an extracellular environment with low levels of growth factors capable of sustaining survival signaling. Thus, the *GF* input node is self-sustaining in the absence of in silico perturbation.	
	← Env	GF	The *GF* input node is self-sustaining in the absence of in silico perturbation.
	← Env	GF_High	The *GF* node represents an extracellular environment with low levels of growth factors capable of sustaining survival signaling. Thus, this node is ON in high growth factor as well.
GF_High	**GF_High** = **GF_High**		
	Env	The GF_{High} node in our model represents an extracellular environment with saturating levels of growth factors; this input node is self-sustaining in the absence of *in silico* perturbation.	
	← Env	GF_High	The GF_{High} input node is self-sustaining in the absence of *in silico* perturbation.
Glucose	**Glucose** = **Glucose**		
	Env	The *Glucose* = OFF input node represents sufficent nutrient for quiescent cell survival aided by autophagy. Cell cycle entry, in contrast, requires *Glucose* = ON to reduce the AMP/ATP ratio and deactivate *AMPK* [1].	
	← Env	Glucose	The *Glucose* input node is self-sustaining in the absence of *in silico* perturbation.
Pyruvate_Ext	**Pyruvate_Ext** = **Pyruvate_Ext**		
	Env	The *Pyruvate_Ext* node represents saturating access to extracellular pyruvate.	
	← Env	Pyruvate_Ext	The *Pyruvate_Ext* input node is self-sustaining in the absence of *in silico* perturbation.

Table S1b: ROS module

Target Node	Node Gate<br>Node Type	Node Description

Table S1b: ROS module

	Link Type	Input Node	Link Description
ROS_Ext	**ROS_Ext = ROS_Ext**		
	Env	The *ROS_Ext* node in our model represents an extracellular environment with high levels of ROS; this input node is self-sustaining in the absence of *in silico* perturbation.	
	← Env	ROS_Ext	The *ROS_Ext* input node is self-sustaining in the absence of *in silico* perturbation.

Table S1c: GF_Basal_MAPK module

Target Node	Node Gate<br>Node Type<br>Link Type	Node Description<br>Input Node	Link Description
RTK	**RTK** = (**GF_High** or **GF**) and not **CAD**		
	Rec	The ON state of the *RTK* node in our model represents basal growth receptor activation (required to keep a normal cell alive). Thus it requires the absence of *CAD* and at least low growth levels of growth factors in the extracellular environment [2].	
	← Ligand	GF	The ON state of the *RTK* node in our model represents basal growth receptor activation by low / medium growth factor availability, encoded by the *GF* node (required to keep a normal cell alive).
	← Ligand	GF_High	Similarly, high growth factor availability also keeps *RTK* on.
	⊢ Per	CAD	Caspase-activated DNase (*CAD*) inhibition of receptor tyrosine kinases ensures that apoptotic cells no longer maintain even basal levels of growth signaling.
Shc	**Shc** = **RTK** and **GF_High**		
	Adap	*Shc* is ON when *RTK*s are activated by high levels of extracellular growth factors (capable of driving proliferation) [3].	
	← Compl	GF_High	The ON state of *Shc* in our model encode the change from basal *Shc* recruitment to weakly stimulated *RTKs* to the level of recruitment seen in high growth factor environments, capable of mediating *Ras* activation [3].
	← Compl	RTK	*Shc* proteins are adaptors that binds to phosphor-tyrosine motifs, facilitating their recruitment to activated receptors such as receptor tyrosine kinases *RTKs* [3].
Grb2	**Grb2** = **RTK** and **Shc**		
	Adap	*Grb2* is recruited to *RTKs* upon ligand binding and subsequent recruitment of *Shc* adaptors [4].	

Table S1c: GF_Basal_MAPK module

	← Compl	RTK	The SH2 domain of *Grb2* binds to a phosphotyrosine residue in the activated *RTK*, where it functions as an adaptor protein [4].
	← Compl	Shc	*Shc* proteins phosphorylated by tyrosine kinases represent binding sites for *Grb2*, aiding its recruitment to active RTKs [3].
SOS	**SOS** = **Grb2**		
	GEF	*RTK*-bound *Grb2* recruits *SOS*, a guanine nucleotide–exchange protein (GEF) that converts inactive *Ras* to its active GTP-bound form [4].	
	← Compl	Grb2	*Grb2* recruits *SOS* to activated *RTK*s [4].
Ras	**Ras** = **Grb2** and **SOS**		
	GTPa	*Ras* activation requires the GEF activity of *SOS* and the *RTK*-linked (active) adaptor protein *Grb2* [4].	
	← Compl	Grb2	*RTK*-bound *Grb2* is required to recruits *SOS*, the GEF responsible for converting inactive *Ras* to its GTP-bound active form [4].
	← GEF	SOS	*SOS* is a GEF that is recruited to activate *Ras* near ligand-bound, active *RTK*s [4].
RAF	**RAF** = **Ras** and not **Casp3**		
	K	*Raf* is active in response to *Ras* activity in the absence of *Caspase 3*. As active *Raf-1* is continuously dephosphorylated and bound by *14-3-3*, which translocates it to the cytoplasm from the plasma membrane (not modeled explicitly), ongoing *Ras* activity is necessary to keep it ON [5].	
	← P	Ras	Active *Ras* phosphorylates *Raf*, enhancing its kinase activity [5].
	⊣ Lysis	Casp3	*Raf-1* is cleaved and inhibited by *Caspase 3* [6].
MEK	**MEK** = **RAF**		
	K	*Raf* phosphorylates and activates the *MEK* kinase [5].	
	← P	RAF	*Raf* phosphorylates and activates the *MEK* kinase [5].
ERK	**ERK** = **MEK** and not **BIK**		
	K	The *ERK* kinase is active when phoisphotylated by *MEK* [5] and allowed to translocate to the nucleus in the absence of *BIK* [7].	
	← P	MEK	*MEK* phosphorylates and activates the *ERK* kinase [5].
	⊣ IBind	BIK	*BIK* binds to active, phosphorylated *ERK1/2* and suppresses its nuclear translocation [7].
mTORC2	**mTORC2** = (**PIP3** or not **S6K**) and not **Casp2**		

Table S1c: GF_Basal_MAPK module

	PC		Our model assumes that *mTORC2* is active in quiescent cells with basal levels of *PI3K* activity leading to basal *PIP3* generation [8]. Alternatively, the absence of high growth factor-stimulated *mTORC1* and *S6K1* can also increase *mTORC2* activity [9, 10]. In contrast, *Caspase-2* degrades a key *mTORC2* component and deactivates the complex [11].
	← PBind	PIP3	PtdIns(3,4,5)P3 (*PIP3*), interacts with the *mTORC2* component *Sin1* to release its inhibition on the *mTOR* kinase domain. Thus, *PIP3* is necessary for *mTORC2* activation [8].
	⊢ P	S6K	*Rictor*, a component of the *mTORC2 complex*, undergoes *S6K1*-mediated phosphorylation at T1135, dampening *mTORC2*-dependent phosphorylation of *Akt* [9, 10].
	⊢ Lysis	Casp2	*Caspase-2* degrades a key *mTORC2* component (*Rictor*) and inhibits downstream *Akt* activation [11].
PI3K	**PI3K** = **RTK** or **Ras**		
	K		In our model, basal *PI3K* activity can be maintained by active *RTK*s or active *Ras*, while high *PI3K* activity requires both (see below).
	← BLoc	RTK	Active *RTK*s recruit *PI3K* to the signaling complex they nucleate, where *PI3K* catalyzes the production of PtdIns(3,4,5)P3 (PIP3) [2].
	← Compl	Ras	*Ras* binds the catalytic subunit of *PI3K* and *Ras* knockdown / over expression decreases /increases the *PI3K*-dependent generation of PIP3 [12, 13].
PIP3	**PIP3** = **PI3K** or **PI3K_H**		
	Met		In our model, PIP3 is ON as a result of basal or high *PI3K* activity.
	← Cat	PI3K	Active *PI3K* recruited to the membrane catalyzes the production of membrane-bound PtdIns(3,4,5)P3 (PIP3) from PtdIns(4,5)P2 (PIP2) [2].
	← Cat	PI3K_H	Active *PI3K* recruited to the membrane catalyzes the production of membrane-bound PtdIns(3,4,5)P3 (PIP3) from PtdIns(4,5)P2 (PIP2) [2].
PDK1	**PDK1** = **PI3K** and **PIP3**		
	K		*PDK1* enzyme activation requires active (at least basal) *PI3K* and *PIP3* [14].
	← BLoc	PI3K	The *PDK1* kinase is recruited to the plasma membrane by *PIP3* at the sites of active *PI3K* activity [14].
	← BLoc	PIP3	The *PDK1* kinase is recruited to the plasma membrane by *PIP3* at the sites of active *PI3K* activity [14].
AKT_B	**AKT_B** = (**PDK1** or **mTORC2**) and **PIP3** and not **Casp3**		
	K		Basal *AKT1* activity in our model requires the absence of *Caspase 3*, the availability of at least basal levels of *PIP3*, and phosphorylation by *PDK1* or *mTORC2*. In contrast, full mitogen-stimulated *AKT1* activation requires phosphorylation by both (see *AKT_H*) [14].

Table S1c: GF_Basal_MAPK module

	← P	PDK1	Membrane-recruited *PDK1* phosphorylates *AKT1* at T308, a critical step in its activation [14].
	← P	mTORC2	Maximal activation of *AKT1* requires phosphorylation of S473 by *mTORC2* [14].
	← BLoc	PIP3	*PIP3* recruits *AKT1* to the plasma membrane and *PIP3* binding changes the conformation of *AKT1* such that it becomes accessible for T308 phosphorylation by *PDK1* [14].
	⊢ Lysis	Casp3	*AKT1* is cleaved and inhibited by *Caspase 3* [6].

Table S1d: GF_PI3K module

Target Node	Node Gate<br>Node Type<br>Link Type	Node Description<br>Input Node	Link Description
p110_H	**p110_H** = (**FoxO3** and not **Nedd4L**) or (**p110_H** and (**FoxO3** or not **Nedd4L**))		
	Prot	In order to capture the cyclic dynamics of *p110* protein expression, we make the assumption that high *p110* protein levels can be induced by *FoxO3* in the absence of the growth factor-activated *Nedd4L* ubiquitin ligase. Once present, high *p110* can be maintained by *FoxO3* transcription, or the absence of activated *Nedd4L*.	
	← TR	FoxO3	*FoxO3* is a direct inducer *p110α* (*PIK3CA*), the catalytic subunit of *PI3K* [15].
	⊢ Ubiq	Nedd4L	*p110α* (*PIK3CA*) is polyubiquitinated by the E3 ligase *Nedd4L*, leading to its proteasomal degradation. Both free *p110α* and the regulatory subunit-bound protein is subject to ubiquitination by *Nedd4L* [16].
	← Per	p110_H	Our model assumes that maintaining high *p110* levels is easier than driving the re-accumulation of the protein following its rapid destruction.
PI3K_H	**PI3K_H** = **p110_H** and **PI3K** and **RTK** and **Ras**		
	K	Full, peak-level activation of *PI3K* requires high levels of *p110* protein, basal *PI3K* activation, active *RTK*s, and active *Ras*. As the ON-state of *Ras* in our model represents strong *Ras* activation in the presence of proliferation-inducing (high) growth factors, *PI3K_H* activation can only occur in these conditions.	
	← Per	p110_H	High levels of *PI3K* activity in response to strong growth factor stimulation only occur in cells that express high levels of *p110* protein [17].
	← Per	PI3K	In our model, high *PI3K* activation is contingent on the ON-state of the basal *PI3K* node.

Table S1d: GF_PI3K module

	$\xleftarrow{\text{BLoc}}$	RTK	High levels of *PI3K* activation only occur at growth factor-bound *RTK*s, which recruit and activate *PI3K* at the plasma membrane [14].
	$\xleftarrow{\text{Compl}}$	Ras	*Ras* binds the catalytic subunit of *PI3K* and *Ras* knockdown/over-expression decreases/increases the *PI3K*-dependent generation of PIP3 [12, 13].
AKT_H	**AKT_H** = **AKT_B** and **p110_H** and **PI3K_H** and **PIP3** and **PDK1** and **mTORC2** and **Ras**		
	K		In contact to basal *AKT1*, high *AKT1* activity in our model requires basal *AKT1* (*AKT_B*), the ongoing presence of high *p110* protein levels along with active *PI3K_H* and *PIP3*. In addition this maximal *AKT1* activation requires phosphorylation by both *PDK1* and *mTORC2*, as well as active *Ras* [14].
	$\xleftarrow{\text{Per}}$	AKT_B	In our model, high *AKT1* activation is contingent on the ON-state of basal *AKT1* (*AKT_B*).
	$\xleftarrow{\text{P}}$	p110_H	Ongoing high *p110* availability and *PI3K_H* activity are required to induce maximal activation of *AKT_H* [14].
	$\xleftarrow{\text{P}}$	PI3K_H	Ongoing high *p110* availability and *PI3K_H* activity are required to induce maximal activation of *AKT_H* [14].
	$\xleftarrow{\text{BLoc}}$	PIP3	*PIP3* recruits *AKT1* to the plasma membrane and *PIP3* binding changes the conformation of *AKT1* such that it becomes accessible for T308 phosphorylation by *PDK1* [14].
	$\xleftarrow{\text{P}}$	PDK1	Membrane-recruited *PDK1* phosphorylates *AKT1* at T308, a critical step in its activation [14].
	$\xleftarrow{\text{P}}$	mTORC2	Maximal activation of *AKT1* requires phosphorylation of S473 by *mTORC2* [14].
	$\xleftarrow{\text{Compl}}$	Ras	*Ras* binding to the catalytic subunit of *PI3K* is required for its full potency in *PIP3* generation [12, 13]. Active *Ras* is thus required for inducing peak *AKT_H* activity.
FoxO3	**FoxO3** = (not(**AKT_H** and (**ERK** or **AKT_B** or **Plk1** or **Plk1_H** or **p53**)) and not(**Plk1** and **Plk1_H** and **ERK**)) or ((**AMPK** or **SIRT3**) and not(**AKT_H** and (**Plk1** or **Plk1_H** or **p53**)))		
	TF		In order to account for all the influences on *FoxO3* activity, we used the following logic. In the absence of basal or high *AKT1* as well as *ERK*, *FoxO3* remains active. In addition, *FoxO3* can overcome peak (*AKT_H*) activation only if no other inhibitor is present and *AKT_B* is OFF (indicating that *AKT1* levels are falling)[14], or if *AMPK* [18, 19] or *SIRT3* [20] activate *FoxO3* in the absence of *Plk1* and *P53* [21, 22]. Finally, the joint activity of *ERK* and *Plk1* can also block *FoxO3* [21, 23].
	$\xleftarrow{\text{PLoc}}$ ⊣	AKT_H	*AKT1* mediates the translocation of the *FoxO3* our of the nucleus through direct phosphorylation of three conserved residues. These events create a recognition site for *14-3-3* family proteins, which export and sequester *FoxO3* in the cytosol [14].

Table S1d: GF_PI3K module

	$\dashv$ PLoc	AKT_B	*AKT1* mediates the translocation of the *FoxO3* our of the nucleus through direct phosphorylation of three conserved residues. These events create a recognition site for *14-3-3* family proteins, which export and sequester *FoxO3* in the cytosol [14].
	$\dashv$ P	ERK	ERK downregulates *FoxO3* transcriptional activity by phosphorylating it at three Serines, inducing its *MDM2*-mediated ubiquitination and degradation [23].
	$\dashv$ PLoc	Plk1	*Plk1* binds *FoxO3*, induces its translocation to the cytosol, phosphorylates it and suppresses its activity through most of the the cell cycle, but most significantly during G2 and M [21].
	$\dashv$ PLoc	Plk1_H	*Plk1* binds *FoxO3*, induces its translocation to the cytosol, phosphorylates it and suppresses its activity through most of the the cell cycle, but most significantly during G2 and M [21].
	$\dashv$ Ind	p53	*p53*-induced increase in *MDM2*, an ubiquitin E3 ligase, promotes *FoxO1* and *FoxO3* ubiquitination and degradation in a manner dependent on *FoxO* phosphorylation at *AKT* sites [22, 24].
	$\leftarrow$ P	AMPK	*AMPK* phosphorylation of *FoxO3* at its C-terminal portion (its the transactivation domain) increases its transcriptional activity without altering its cytoplasmic-nuclear shuttling [18, 19].
	$\leftarrow$ Deacet	SIRT3	*SIRT3* deacetylates *FoxO3* at K271 and K290, which promotes *FoxO3* nuclear localization and increases transcription [20].
PLCgamma	**PLCgamma** = **RTK** and **Grb2** and **p110_H** and **PI3K_H** and **PIP3**		
	Enz		Peak activation of $PLC\gamma$ requires active an *RTK* receptor node bound by active *Grb2*, as well as high *PI3K* activity (including high *p110* availability and the presence of PIP3).
	$\leftarrow$ P	RTK	The SH2 domains of $PLC\gamma$ binds to active *RTK*s at tyrosine autophosphorylation sites, leading to tyrosine phosphorylation of $PLC\gamma$ and stimulation its enzymatic activity [25, 26].
	$\leftarrow$ BLoc	Grb2	*RTK* tyrosine autophosphorylation induces $PLC\gamma$ binding to the *Grb2* adaptor protein and likely aids the translocation of $PLC\gamma$ to the plasma membrane [27].
	$\leftarrow$ BLoc	p110_H	Membrane targeting of $PLC\gamma$ to growth receptor stimulation requires *PI3K* activity and *PIP3* generation near growth receptors [28]. Thus, peak $PLC\gamma$ activity in our model requires high *p110* protein expression [29].
	$\leftarrow$ BLoc	PI3K_H	In addition to high *p110* protein levels, high *PI3K* activation is also required to fully activate $PLC\gamma$ [28, 29].
	$\leftarrow$ BLoc	PIP3	Membrane targeting of $PLC\gamma$ to growth receptor stimulation is mediated by *PIP3* binding of $PLC\gamma$ [28, 29].

Table S1d: GF_PI3K module

IP3	**IP3** = **PLCgamma**		
	Met	Membrane-bound, active *PLCγ* is responsible for converting phosphatidylinositol(4,5)P2 (*PIP2*) to the second messenger inositol(1,4,5)P3 (*IP3*) responsible for triggering a sudden Ca^{2+} influx from the endoplasmic reticulum, along with *DAG* (diacylglycerol, another second messenger) [30].	
	← Cat	PLCgamma	Membrane-bound, active *PLCγ* is responsible for converting phosphatidylinositol(4,5)P2 (*PIP2*) to the second messenger inositol(1,4,5)P3 (*IP3*) responsible for triggering a sudden Ca^{2+} influx from the endoplasmic reticulum, along with *DAG* (diacylglycerol, another second messenger) [30].
Ca2p	**Ca2p** = **IP3**		
	Met	*IP3* travels from the cell membrane to the endoplasmic reticulum where it opens *IP3*-sensitive Ca^{2+} channels, releasing a sudden Ca^{2+} efflux from the ER into the cytosol [31].	
	← Loc	IP3	*IP3* travels from the cell membrane to the endoplasmic reticulum where it opens *IP3*-sensitive Ca^{2+} channels, releasing a sudden Ca^{2+} efflux from the ER into the cytosol [31].
Nedd4L	**Nedd4L** = **Ca2p** and **IP3**		
	UbL	Activation of *Nedd4L* requires both Ca^{2+} and *IP3* binding [32].	
	← Compl	IP3	In order to transition to its active form, the E3 ubiquitin ligase *Nedd4L* binds Ca^{2+} and inositol 1,4,5-trisphosphate (*IP3*) [32].
	← Compl	Ca2p	In order to transition to its active form, the E3 ubiquitin ligase *Nedd4L* binds Ca^{2+} and inositol 1,4,5-trisphosphate (*IP3*) [32].

Table S1e: GF_mTOR module

Target Node	Node Gate<br>Node Type<br>Link Type	Node Description<br>Input Node	Link Description
FoxO1	**FoxO1** = not(**AKT_H** or **Plk1** or **p53**) or (**AMPK** and **SIRT3** and not(**AKT_B** and **p53**))		
	TF	*FoxO1* is transcriptionally active in the absence of peak *AKT1* activation [14], *Plk1* activity [33], or *p53* activation [22]. Alternativelty, *AMPK* [34] and *SIRT3* [35] can activate *FoxO1* if there is no active *p53* (aided by at least basal *AKT*).	
	⊣ PLoc	AKT_H	*AKT1* mediates the translocation of *FoxO1* our of the nucleus through direct phosphorylation of three conserved residues. These events create a recognition site for *14-3-3* family proteins, which export and sequester *FoxO1* in the cytosol [14].

Table S1e: GF_mTOR module

	⊣ PLoc	AKT_B	*AKT1* mediates the translocation of *FoxO1* our of the nucleus through direct phosphorylation of three conserved residues. These events create a recognition site for *14-3-3* family proteins, which export and sequester *FoxO1* in the cytosol [14].
	⊣ PLoc	Plk1	*Plk1* interacts with and phosphorylates *FoxO1*, mainly at the G2/M phase of the cell cycle. *Plk1*-mediated phosphorylation leads to the impairment of *FoxO1*'s transcriptional activity in an *Akt*-independent manner. *Plk1*-induced *FoxO1* phosphorylation causes its nuclear exclusion [33].
	⊣ Ind	p53	*p53*-induced *MDM2*, an ubiquitin E3 ligase, promotes *FoxO1* and *FoxO3* ubiquitination and degradation in a manner dependent on *FoxO* phosphorylation at *AKT* sites [22].
	← PLoc	AMPK	*AMPK* induces *FoxO1* activity by phosphorylation at Ser383, Thr649, and Ser22; where Ser22 phosphorylation prevents binding to the *14-3-3* proteins (these inhibit DNA binding and mask *FoxO1*'s nuclear localization signal) [34].
	← Deacet	SIRT3	*SIRT3* binds to and deacetylates *FoxO1* and increases the transtiption of its antioxidant targets (catalase, MnSOD) [35].
MnSOD	**MnSOD** = **FoxO1** or **FoxO3** or **FoxM1** or (**ERK** and not **p27Kip1**) or **SIRT3**		
	Enz		Mitochondrial superoxide dismutase 2 (*SOD2*), also known as manganese-dependent superoxide dismutase or *MnSOD*, is induced by *FoxO* transcription factors, as well as *FoxM1* during cell cycle progression [36, 37, 38]. In addition, increased *ERK* in the absence of $p27^{Kip1}$ can also induce textitMnSOD [39].
	← TR	FoxO1	*FoxO1* is a direct transcriptional inducer of *MnSOD*, potentially dependent on the presence of the mechanosensitive *YAP* transcription factor (not explicitiy modeled, but assumed to be active in non-contact-inhibited cells we model) [37].
	← TR	FoxO3	*FoxO3* is a direct transcriptional inducer of *MnSOD* [36]. Note that *FoxO3* does not appear to interact with, or require *YAP* to induce *MnSOD* [37].
	← TR	FoxM1	*FoxM1* can substitute for *FoxOs* to keep *MnSOD* transctiption going during the cell cycle, when *FoxO* activity is blocked by *AKT* [38].
	← Ind	ERK	*ERK*-mediated induciton of *ATF1* upregulates *STAT3*, which in turn induces *MnSOD* expression; an effect observed in textitp27^{Kip1}-/- cells [39].
	⊣ TR	p27Kip1	Loss of $p27^{Kip1}$ upregulates the expression of *STAT3* in an *ERK*-dependent manner (via *ATF1*). *STAT3*, in turn, is a direct transcriptional inducer of *MnSOD* [39]. As a result, textitp27^{Kip1}-/- cells have elevated *MnSOD* levels [39].
	← Deacet	SIRT3	*SIRT3* deacetylates and activates *MnSOD* [40].
p21 _mRNA	**p21_mRNA** = (**FoxO1** and **FoxO3**) or ((**FoxO1** or **FoxO3**) and not **Myc**) or **p53**		

Table S1e: GF_mTOR module

	mRNA	Our model requires both *FoxOs* or *p53* to induce $p21^{Cip1}$ if *Myc* is active, but only one of the two *FoxOs* or *p53* if *Myc* is OFF. This is based on data showing that both *FoxO3* and *FoxO1* bind and induce the $p21^{Cip1}$ promoter, and that loss of *Myc* repression alone is not sufficient to induce $p21^{Cip1}$ [41].	
	← TR	FoxO1	$p21^{Cip1}$ is a direct transcriptional target of *FoxO1* [41].
	← TR	FoxO3	$p21^{Cip1}$ is a direct transcriptional target of *FoxO3* [41].
	⊢ TR	Myc	*Myc* is a direct transcriptional repressor of the $p21^{Cip1}$ promoter (it is recruited by the DNA-binding *Miz-1*) [42, 43].
	← TR	p53	$p21^{Cip1}$ is a direct transcriptional target of *p53* [44].
TSC2	**TSC2** = **AMPK** or **MP_Low** or not **AKT_H** or not(**AKT_B** or **ERK**)		
	Prot	Blocking *TSC2* requires ongoing mitogen stimulation through *AKT* and/or *ERK*, as well as the absence of active *AMPK* [45] and low $\Delta\Psi_M$ [46]. In our model, *TSC2* inhibition in the absence of an energy deficit requires high (peak) *AKT* activity, supported by either *ERK* or basal *AKT* (assuring that complete loss of *AKT* activity is not impending) [47].	
	← P	AMPK	*AMPK* phosphorylates *TSC2* to enhance its activity to block *mTORC1* in response to energy deprivation [45].
	← ComplProc	MP_Low	$\Delta\Psi_M$ lowered with OXPHOS uncouplers results in *TSC2* activation and *mTORC1* inhibition even in the absence of *AMPK* [46].
	⊢ P	AKT_H	*TSC2* is phosphorylated by *AKT1*, inhibiting it by dissociating *TSC2* from lysosomal membranes [48], where it stimulates GTP hydrolysis of the small GTPase *Rheb*, this inactivating it [47].
	⊢ P	AKT_B	*TSC2* is phosphorylated by *AKT1*, inhibiting it by dissociating *TSC2* from lysosomal membranes [48], where it stimulates GTP hydrolysis of the small GTPase *Rheb*, this inactivating it [47].
	⊢ P	ERK	*ERK* phosphorylates *TSC2* directly, causing dissociation of the complex and inhibition of its activity [49]. In addition, the *ERK* target *p90RSK* can also inactivate *TSC2* [50].
PRAS40	**PRAS40** = not **AKT_H** and not(**AKT_B** and **mTORC1**)		
	Prot	*PRAS40* is inhibited by peak *AKT1* activity aided by either basal *AKT1* (meaning *AKT_H* is on its way down), or ongoing *mTORC1* activation. Both *AKT1* and *mTORC1* phosphorylate *PRAS40*, leading to its dissociation from *mTORC1* [51].	
	⊢ P	AKT_H	*PRAS40* is an inhibitory component of the *mTORC1* complex. It is phosphorylated by *AKT*, triggering its dissociation from *mTORC1* and loss of *mTORC1* inhibition [52].
	⊢ P	AKT_B	*PRAS40* is phosphorylated by *AKT*, triggering its dissociation from *mTORC1* [52].

Table S1e: GF_mTOR module

	⊣ P	mTORC1	*PRAS40* is a substrate of the *mTORC1* kinase; its phosphorylation aids its dissociation from *mTORC1* and its sequestration by 14-3-3 proteins [53].
DAG	**DAG** = **PLCgamma**		
	Met	Membrane-bound, active *PLC*γ is responsible for converting phosphatidylinositol(4,5)P2 (*PIP2*) to the second messenger diacylglycerol (*DAG*), along with *IP3* [30].	
	← Cat	PLCgamma	Membrane-bound, active *PLC*γ is responsible for converting phosphatidylinositol(4,5)P2 (*PIP2*) to the second messenger diacylglycerol (*DAG*), along with *IP3* [30].
Rheb	**Rheb** = **DAG** and not **TSC2**		
	GTPa	*PKC* (and *DAG*)-dependent activation of *mTORC1* recruits *mTORC1* to the site of *Rheb* activity (to prenuclear lysosomes), while *AKT* and *ERK*-mediated *TSC2* inhibition guarantees that *Rheb* remains potent [54, 55, 56].	
	← Loc	DAG	The second messenger *DAG* activates both classical and novel *PKC*s. One of its targets, *PKC*η, is responsible for the translocation and accumulation of *mTORC1* to perinuclear lysosomes, where the majority of *Rheb* is anchored. Thus, *DAG* brings *Rheb* in proximity with its target, *mTORC1* [55].
	⊣ GAP	TSC2	*TSC2*, a key component of the heterotrimeric *TSC* complex, is a GTPase activating protein (GAP) that induces ATP hydrolysis and deactivation of the small GTPase *Rheb* [54].
mTORC1	**mTORC1** = ((not **AMPK** and not **PRAS40** and **Rheb**) or (**E2F1** and (not **AMPK** or **ERK**)) or (**CyclinB** and **Cdk1** and **GSK3**)) and not **Casp3**		
	PC	*mTORC1* is activated by mitogenic signals via *Rheb* in the absence of both active *PRAS40* and *AMPK*. Independently, *E2F1* can promote *mTORC1* activity as long as *AMPK* is absent, or *ERK* is active (*E2F1* can override *mTORC1* inhibition by *TSC2* [57]; we assume it requires growth signaling to do so.). Finally, the mitotic *Cyclin B* and its *Cdk1* kinase can also activate *mTORC1*, aided by *GSK3*.	
	⊣ P	AMPK	*AMPK* inhibits *mTORC1* by direct phosphorylation of the *mTORC1* member *Raptor*, as well as by activation of the *TSC1/2* pathway (also included in the model via its effects on *Rheb*) [58].
	⊣ IBind	PRAS40	*PRAS40* is an inhibitory component of the *mTORC1* complex, removed by phosphorylation by *AKT* or *mTORC1* itself [47].
	← Compl	Rheb	The *Rheb* small GTPase binds *mTORC1* directly and activates the complex [59].
	← Loc	E2F1	*E2F1* induces *mTORC1* activity by inducing *mTORC1* translocation to late endosomes. This effect does not require *AKT* and is not blocked by high levels of *TSC2* [57].

Table S1e: GF_mTOR module

	← P	ERK	*ERK1* and *ERK1* bind to the *mTORC1* component *Raptor* and phosphorylate it at Ser8, Ser696, and Ser863, modifications that promote *mTORC1* activity [60]. Here we assume that *ERK* can help *E2F1* maintain active *mTORC1* in the presence of *AMPK* (*E2F1* was shown to override *mTORC1* inhibition by *TSC2* [57]).
	← Ind	CyclinB	During mitosis, *mTORC1* is activated by the G2/M-specific phosphorylation of *Raptor*, a component of *mTORC1*, by *CyclinB* / *Cdk1* complexes, aided by *GSK3* [61].
	← Ind	Cdk1	During mitosis, *mTORC1* is activated by the G2/M-specific phosphorylation of *Raptor*, a component of *mTORC1*, by *CyclinB* / *Cdk1* complexes, aided by *GSK3* [61]. Here we assume that G2/M-specific phosphorylation of *Raptor* by *Cdk1* overrides direct inhibition by *AMPK*, keeping *mTORC1* active during mitosis.
	← Ind	GSK3	During mitosis, *mTORC1* is activated by the G2/M-specific phosphorylation of *Raptor*, a component of *mTORC1*, by *CyclinB* / *Cdk1* complexes, aided by *GSK3* [61].
	⊣ Lysis	Casp3	*Raptor*, a key component of the *mTORC1* complex, is cleaved and inhibited by *Caspase 3* [62].
S6K	**S6K** = **mTORC1** and not(**CyclinB** and **Cdk1**) and not **Casp3**		
	K	*S6K* is activated by *mTORC1* in the absence of *Caspase 3*.	
	← P	mTORC1	*mTORC1* phosphorylates and activates 40S ribosomal S6 kinases (*S6K*s) [63].
	← Ind	CyclinB	During mitosis, p70 *S6K* is a physiological substrate of Cdk1/Cyclin, keeping its mitotic activity low. This interaction may be responsible for supressed protein synthesis during mitosis [64].
	← Ind	Cdk1	During mitosis, p70 *S6K* is a physiological substrate of Cdk1/Cyclin, keeping its mitotic activity low. This interaction may be responsible for supressed protein synthesis during mitosis [64].
	⊣ Lysis	Casp3	*S6K* is cleaved and inhibited by *Caspase 3* [65].
eIF4E	**eIF4E** = **mTORC1** and not **Casp3**		
	Prot	*eIF4E* is activated by *mTORC1*-mediated *4EBP* repression in the absence of *Caspase 3*.	
	← Ind	mTORC1	*mTORC1* phosphorylates *4EBP*, triggering its dissociation from *eIF4F*, and thus promoting translation initiation [47].
	⊣ Lysis	Casp3	*eIF4E* is cleaved and inhibited by *Caspase 3* [66].
GSK3	**GSK3** = (not **AKT_H** or **U_Kinetochores**) and (**SIRT3** or not **AKT_B** or not(**S6K** and **ERK**))		

Table S1e: GF_mTOR module

	K		*GSK3β* activity can be completely blocked by peak *AKT* activity (*AKT_H*), or by the joint action of *S6K* and *ERK*. Peak *AKT*-mediated *GSK3β* inhibition is overriden during metaphase (marked by the presence of unattached kinetochores, or *U_Kinetochores* = ON), when active *AKT* localizes primarily to the centrosomes and spindle poles, leaving cytosolic *GSK3β* active. In addition, *SIRT3* can deacetylate and activate *GSK3β*, overriding the joint inhibitory effects of *S6K* and *ERK*.
	⊢ P	AKT_H	*AKT* blocks *GSK3β* kinase activity via an inhibitory phosphorylation on the amino terminus, which blocks the substrate accessibility of *GSK3β* [14].
	⊢ P	AKT_B	*AKT* blocks *GSK3β* kinase activity via an inhibitory phosphorylation on the amino terminus, which blocks the substrate accessibility of *GSK3β* [14]. We assume that loss of basal *AKT* activity overrides the joint effects of *S6K* and *ERK* on *GSK3* inhibition.
	← Loc	U_Kinetochores	During metaphase, active *AKT* and phosphorylated (inactive) *GSK3β* localize primarily to the centrosomes and spindle poles, leaving cytosoling *GSK3β* active [67].
	← Deacet	SIRT3	*SIRT3* activates *GSK3β* by deacetylating it at K15 [68].
	⊢ P	S6K	*GSK3β* is a direct phosphorylation target of *S6K1*, resulting in its inhibition [9].
	⊢ P	ERK	*ERK* binds and phosphorylates *GSK3β* at Thr-43, which primes it for subsequent phosphorylation by the *ERK* target *p90RSK* at Ser-9, which inactivates *GSK3β* [69].
Autophagy	**Autophagy** = (**FoxO1** or **FoxO3**) and **AMPK** and not **mTORC1** and not **BIM** and (**mROS** or **cROS** or **cROS_H**)		
	Proc		To account for increased autophagy triggered by low ATP/AMP, we assume that *AMPK* activation aided by *FoxO1/3* in the absence of *mTORC1* or *BIK*, can increase autophagy from its basal levels to a state that can regenerate sufficient ATP for survival. In addition to energy stress, this increase also requires an increase in internal ROS. Note that the current model does not include the processes that eventualy kills the cell in response to prolonged glucose withdrawal, only the metastable homeostasis before the cell permanenly runs out of energy.
	← ComplProc	FoxO3	*FoxO3* stimulates lysosomal proteolysis by inducing the expression of several autophagy-related genes, such as *LC3b*, *Gabarapl1*, and *PI3KIII* (required for autophagy), *Ulk2*, *Atg12l*, and *Beclin1*.
	← ComplProc	FoxO1	*FoxO1* increased *Rab7* expression, which mediates late autophagosome-lysosome fusion [70]. In addition to its transcriptional role, *FoxO1* is also required for autophagy in human cancer cell lines in response to oxidative stress or serum starvation, where it is acetylated and bound to *Atg7* (essential autophagy effector enzyme) [71].

Table S1e: GF_mTOR module

	$\leftarrow$ ComplProc	AMPK	During glucose starvation, *AMPK* directly activates *Ulk1* through phosphorylation of Ser-317 and Ser-777, which in turn promotes autophagy [72].
	$\vdash$ ComplProc	mTORC1	High *mTORC1* activity prevents *Ulk1* activation by phosphorylating *Ulk1* Ser-757, which disrupts its interaction with *AMPK* [72].
	$\vdash$ IBind	BIM	*BIM* inhibits autophagy by interacting with and changing the localization of *Beclin 1* [73].
	$\leftarrow$ ComplProc	mROS	ROS production, the main intrinsic source of which are mitochidria, aids autophagy (the exact mechanism of this is still debated) [74].
	$\leftarrow$ ComplProc	cROS	ROS production is induced immediately upon nutrient deprivation, where it aids autophagy (the exact mechanism of this is still debated) [74].
	$\leftarrow$ ComplProc	cROS_H	ROS production is induced immediately upon nutrient deprivation, where it aids autophagy (the exact mechanism of this is still debated) [74].
IKKa	**IKKa** = **AKT_H**		
	K		*IKKα* is a subunit of the *IKK* protein complex composed of two catalytic subunits, *IKKα* and *IKKβ*, and the regulatory protein *NEMO*. Activation of the transcription factor *NF-κB* is mediated by the IKK complex, which phosphorylates and degrades the inhibitory *IκB* proteins [75].
	$\leftarrow$ P	AKT_H	*IKKα* is phosphorylated by *AKT* at T23, and as subsequent *NF-κB* activation is induced when high *AKT* activity is observed, we model it via the *AKT_H* input [76].
NF_kB	**NF_kB** = **IKKa** and not **FoxO3** and not **p53**		
	TF		*NF-κB* is a transcription factor primarily known as a master regulator of inflammatory signaling and the immune system. It is activated via the destruction of its inhibitory binding partner *IκB*, which is phosphorylated by the *IKK* complex subsequently destroted [75]. *FoxO3* and *p53* both limit *NF-κB*-mediated transcription by trapping one of its subunits in the cytosol and competing for a limited pool of cofactors, respectively [77, 78].
	$\leftarrow$ Ind	IKKa	*IKKα*, part of the *IKK* complex, phosphorylates and degrades the inhibitory *IκB* proteins [75]; an action that can be independent of the *IKKβ* subunit [79].
	$\vdash$ IBind	FoxO3	*FoxO3* binds and traps *NF-κB*' *RelA* subunit in the cytosol [77].
	$\vdash$ Ind	p53	*p53*-mediated inhibition of *NF-κB* is likely the result of their competition for a limited pool of *p300/CBP complexes*, as they both require these transcriptional coactivators [78].

Table S1f: Warburg module

Target Node	Node Gate<br>Node Type<br>Link Type	Node Description<br>Input Node	Link Description
Glycolysis	**Glycolysis** = (**Glucose** and **AKT_B** and **NADp_c**) or **Glycolysis_H**		
	Proc	The *Glycolysis* node represents the metabolic process by which cells bring in glucose from the extracellular environment and convert it into pyruvate [80]. As a step of this pathway reduces NAD$^+$ [80], our model assumes that the *Glycolysis* node turns on when there is extracelluar glucose and the *NADp_c* node representing cytosolic NAD$^+$ is ON.	
	← ComplProc	Glucose	*Glycolysis* requires glucose uptake from the envrironment, which is converted to pyruvate in the cytosol [80].
	← Ind	AKT_B	*Akt* increases glucose uptake by promoting *Glut1* localization to the cell surface [81], as well as glucose phosphorylation by increasing cellular hexokinase (*HK2*) levels (this effect requires mTORC1 activity) [82].
	← Cat	NADp_c	NAD$^+$ is converted to NADH at the glyceraldehyde 3-phosphate dehydrogenase (GAPDH) step of glycolysis [80]. Inhibition of *NAMPT*, an enzyme essential for NAD$^+$ biosynthesis leads to attenuation of glycolysis [83].
	← Per	Glycolysis_H	To keep our Boolean model conistent with the use of two nodes for three qualitatively distinct levels of glycolysis, *Glycolysis_H* = ON keeps this basal glycolisis node ON as well.
Glycolysis_H	**Glycolysis_H** = (**HIF1** or **Myc**) and **Glycolysis** and **Glucose** and **NADp_c** and **ERK** and not **p53**		
	Proc	The *Glycolysis_H* node represents incrased glycolysis characteristic of proliferative metabolism, driven by increased glucose uptake and enhanced glycolytic enzyme expression downstream of *HIF1* (primarily under anaerobic conditions) or *Myc* (in proliferating cells under aerobic conditions); further aider by *ERK1/2*. In contrast, *p53* activation limits glycolysis.	
	← TR	HIF1	Hypoxia-induced factor-1 or *HIF-1α* is a transcriptional inducer of nealry every step of glycolysis, including *GLUT1* to facilitate increased glucose import [84].
	← TR	Myc	*Myc* is a transcriptional inducer of nealry every step of glycolysis, including *GLUT1/2/4* to facilitate increased glucose import [84].
	← Per	Glycolysis	We assume that basal *Glycolysis* must be active before the *Glycolysis_H* node can turn on.
	← ComplProc	Glucose	*Glycolysis* requires glucose uptake from the envrironment, which is converted to pyruvate in the cytosol [80].
	← Cat	NADp_c	NAD$^+$ is converted to NADH at the glyceraldehyde 3-phosphate dehydrogenase (GAPDH) step of glycolysis [80]. Inhibition of *NAMPT*, an enzyme essential for NAD$^+$ biosynthesis leads to attenuation of glycolysis [83].

Table S1f: Warburg module

	← Ind	ERK	*ERK1/2* phosphorylate *PKM2*, which aids the conversion of PKM2 from a tetramer to a monomer. In the nucleus this monomer leds acts as a protein kinase that phosphorylates histone H3 to boost the transcription of *c-Myc*, which then promotes glycolytic enzyme expression [85].
	⊣ Ind	p53	*p53* slows down glycolysis by multiple mechanisms, including the attenuation of *HIF-1*α transactivation (moderate *p53* activation), *HIF-1*α degradation (high *p53*) [86], and induction of the *TIGAR* enzyme, which lowers fructose 2,6-bisphosphate step of glycolysis [84].
NADp_c	**NADp_c** = not **Glycolysis_H** or **Fermentation** or **NADp_m**		
	Met		In mammalian cells intracellular NAD^+ levels typically fall between 0.2 and 0.5 mM [87]. NAD^+ is consumed by glycolysis but regenerated during pyruvate to lactate conversion (*Fermentation* node in our model). In addition, we assume that mitochodtial NAD^+ can replenish cytosolic NAD^+ via the malate-aspartate shuttle [88].
	⊣ Cat	Glycolysis_H	NAD^+ is converted to NADH during the glyceraldehyde 3-phosphate dehydrogenase (GAPDH) step of glycolysis [80], lowering cytosolic NAD^+.
	← Cat	Fermentation	When cells convert pyruvate into lactate rather than process it in the mitochodtia to drive the TCA cycle, the process uses NADH to regenerate NAD^+ [89, 90].
	← Loc	NADp_m	The malate–aspartate shuttle aids the translocaton of electrons produced during glycolysis across the mitochondrial inner membrane. Its net effect is to oxidize NADH in the cytosol to NAD+ (thus raising cytosolic NAD+ levels) at the expense of reducing mitochondrial NAD+ to NADH [88].
Pyruvate	**Pyruvate** = **Glycolysis** or **Glycolysis_H** or **Pyruvate_Ext**		
	Met		Intracellular pyruvate is the product of glycolysis [89]. In addition, extracellular pyruvate can enter the cytosol via monocarboxylate transporters [91].
	← ComplProc	Glycolysis	Intracellular pyruvate is the product of glycolysis [89].
	← ComplProc	Glycolysis_H	Intracellular pyruvate is the product of glycolysis [89].
	← Loc	Pyruvate_Ext	Extracellular pyruvate enters the cytosol via monocarboxylate transporters [91].
HIF1	**HIF1** = **mTORC1** and **eIF4E** and **S6K** and (**ERK** or not(**GSK3** and **FoxO3**)) and not **p53**		

Table S1f: Warburg module

	TF		Hypoxia-inducible factor 1, *HIF-1α*, is induced under normoxic conditions by growth-promoting signals to drive the Warburg effect in proliferating cells. First, *AKT*-mediated *mTORC1* and its downtream targets (*eIF4E*, *S6K*) increase both transcription and translation of *HIF-1α* [92]. Second, transcriptional activity of *HIF-1α* is boosted my MAPK signaling through *ERK*-mediated phosphorylation [93], while freed from *GSK3β*-mediated degradation and *FoxO3* binding by deactivation of these repressors downstream of *PI3K/AKT* [94, 95]. In contrast, *p53* represses *HIF-1α*-mediated transactivation [86].
	← Ind	mTORC1	*mTORC1* activation downtream of *AKT* increases *HIF-1α* protein expression [96] protein accumulation through enhanced *STAT3* dependent transcription [92].
	← TL	eIF4E	*mTORC1*-induced *eIF4E* activation increases *HIF-1α* protein translation [92].
	← TL	S6K	*mTORC1*-induced *S6K* activation increases *HIF-1α* protein translation [92].
	← P	ERK	*ERK*-mediated phosphorylation of *HIF-1α* regulates its physical interaction with *NPM1*, a histone chaperone and chromatin remodeler. This interaction is increases *HIF-1α* targeting to hypoxia target genes and their transcriptional activation [93].
	⊣ P	GSK3	*GSK3β* phosphorylates and destabolizes *HIF-1α*, independently of thye hypoxia sensor *VHL* [94].
	⊣ IBind	FoxO3	*FoxO3* interferes with and reduces *p300*-dependent *HIF-1α* transcription [95].
	⊣ Ind	p53	Moderate *p53* activation attenuates *HIF-1α* transactivation by competing for the shared *p300* co-activator [86].
Fermentation	**Fermentation** = **ERK** and ((**Glycolysis_H** and **Pyruvate**) or **Pyruvate_Ext**)		
	Proc		In our model *Fermentation* = ON represents high rates of pyruvate to lactate conversion. This requires *ERK*-mediated phosphorylatiom of *PKM2*, which decreases its enzymatic activity (required for estabolishing the metabolic fluxes of the Warbugr effect) and boosts the expression of glycolitic genes [97]. In additiom, *Fermentation* turns ON with increased glycolysis and intracellular pyruvate, or in cells with access to extracellular pyruvate.
	← Ind	ERK	*ERK*-mediated phosphorylatiom of *PKM2* converts this enzyme from a tetramer to a monomer, decreasing its catalytic activity (observed to promote increased pyruvate to lactate conversion) and allowing it to localize to the nucleus where it aids the transcription of *cyclin D1*, *c-Myc*, and glycolytic enzymes [97].
	← ComplProc	Glycolysis_H	Increased glycolysis coupled with low-activity *PKM2* drives the conversion of pyruvate to lactate [97].
	← ComplProc	Pyruvate	The *Fermentation* node represents the conversion of pyruvate to lactate.

Table S1f: Warburg module

	← ComplProc	Pyruvate _Ext	Extracellular pyruvate enters the cytosol via monocarboxylate transporters [91], where its excess can drive fermentation.

Table S1g: Mitochondria module

Target Node	Node Gate<br>Node Type<br>Link Type	Node Description<br>Input Node	Link Description
TCA_cycle	**TCA_cycle** = **Pyruvate** and (**NADp_m** or not **MP_Low**) and not(**Unfused** and **mROS**) and not **cROS_H**		
	Proc	The tricarboxylic acid (TCA) cycle, also known as the citric acid or Krebs (or Szent-Györgyi-Krebs) cycle, consists of a series of reactions driven by pyruvate-derived acetyl-CoA. In eukaryotic cells, the TCA cycle occurs in the matrix of mitochondria. This cycle feeds the Electron Transport Chain (ETC) by transfering electrons to NAD^+ carriers (thus using NAD^+ to generate NADPH). The ETC, in turn, raises the $\Delta\Psi_M$. In our model we ommited an explicit ETC node, and linked the TCA cycle to $\Delta\Psi_M$ (*TCA_Cycle* ⟶ *MP_High* and *TCA_Cycle* ⊣ *MP_Low*).	
	← ComplProc	Pyruvate	The TCA cycle is driven by pyruvate-derived acetyl-CoA.
	← ComplProc	NADp_m	Two steps of the TCA cycle require NAD^+ (mitochondrial NAD^+ is represented in the model by *NADp_m*).
	⊢ ComplProc	MP_Low	The TCA cycle removes electrons from acetyl-CoA and transfers them to carriers that bring them to the electron transport chain (ETC), which in turn generates the mitochondrial membrane potential. As the oxidized electron carriers required to continue to turn the cycle are regenerated by the ETC (e.g., NAD^+), we assume that the TCA cycle works normally if the $\Delta\Psi_M$ is not low, or the mitochondria still have NAD^+.
	⊢ ComplProc	Unfused	Inhibition of fusion, such as *Mfn2* knockdown, leads to fragmented mitochondria with reduced pyruvate, glucose and fatty acid oxidation, and reduces mitochondrial membrane potential [98]. Thus, we assume that unfused mitochondria negatively impact the TCA cycle. That said, experiments in cycling cells indicate that while the speed of the TCA cycle orcillates along with the cell cycle, the main change is an increased glucose-derived flux into TCA cycle in G1. Notably, the *short-term* mitochondrial fragmentation during metaphase does not significantly alter the TCA cycle [99]. To model this lack of disruption, we assume that only unfused mitochondria with high mitochodtial ROS (a consequence of fission triggered by stressors such as hyperglycemic challenge [100]) have significanly disrupted TCA cycles.

Table S1g: Mitochondria module

	⊢ ComplProc	mROS	Mitochondrial ROS can compromose the TCA cycle by blocking two key enzymes [101, 102]. First, the enzyme aconitase, which catalyzes the conversion of citrate to isocitrate, is vulnerable to reactive oxygen and reactive nitrogen species [103]. Second, α ketoglutarate dehydrogenase catalyzes conversion of α-ketoglutarate and coenzyme A to succinyl coA, converting NAD^+ to NADH in the process. This enzyme also has reduced activity in the presence of ROS [104].
	⊢ ComplProc	cROS_H	H_2O_2 exposure revealed that α ketoglutarate dehydrogenase (*KGDH*), succinate dehydrogenase (*SDH*), and aconitase were all inactivated by ROS [102].
MFN1_2	**MFN1_2** = (**PGC1** or **E2F1**) and not (**MP_Low** and **PINK1** and **Mitophagy_High**) and not **Drp1**		
	GTPa		The mitofusins *MFN1* and *MFN2* facilitate fusion of the outer mitochondrial membrane [105]. Their mRNA levels are increased by *PGC-1α* and/or *E2F1*. In contrast, low $\Delta\Psi_M$ on mitochondria subject to mitophagy triggers the activity of *PINK1*, a kinase that recruits the ubiquitin E3 ligase *Parkin* to target both *MFN*s [106, 107]. In our model, we assume that in the presence of low $\Delta\Psi_M$, high mitophagy and PINK1, *E2F1* or *PGC1* cannot accumulate sufficient *MFN1* and *MFN2* to upregulate them above their basal levels. Finally, *Drp1* recruits the fission protein *Fis1*, which binds to *Mfn1/2* and directly inhibits its GTPase activity [108].
	← TR	PGC1	*PGC-1α* is a direct transcriptional inducer of both *MFN1* [109] and *MFN2* [110, 111] by co-activating the estrogen-related receptor α.
	← TR	E2F1	*E2F1* is a direct transcriptional inducer of *MFN2* [112].
	⊢ ComplProc	MP_Low	Low $\Delta\Psi_M$ on mitochondria subject to mitophagy triggers the activity of the *PINK1* kinase, which recruits the ubiquitin E3 ligase *Parkin* to target both *MFN*s [106, 107].
	⊢ ComplProc	Mitophagy_High	Mitophagy triggers the activity of *PINK1* kinase, which recruits the ubiquitin E3 ligase *Parkin* to target both *MFN*s [107].
	⊢ Deg	PINK1	Active *PINK1* kinase recruits the ubiquitin E3 ligase *Parkin* to target both *MFN*s [107].
	⊢ Ind	Drp1	*Drp1* recruits the fission protein *Fis1*, which binds to *Mfn1*, *Mfn2*, and *OPA1* and inhibits their GTPase activity, blocking the fusion machinery [108].
Hyperfused_OM	**Hyperfused_OM** = **MFN1_2** and not(**Unfused** or **Drp1** or (**PINK1** and **Mitophagy_High**))		

Table S1g: Mitochondria module

	MSt		In mammalian cells, mitochondrial fusion is a two-step process in which outer mitochondrial membrane fusion is not always followed by inner membrane fusion [113] (the latter is required for boosting the $\Delta\Psi$ of the fused structure [114]). Outer membrane fusion is driven by mitofusins *MFN1/2* [113] and blocked by the fission factor *Drp1*, as well as *PINK1*-mediated strong mitophagy [107]. In our model, a hyperfused state is also mutually exclusive with the fragmented state, represented by the *Unfused* node.
	← Compl	MFN1_2	*MFN*s facilitate mitochondrial fusion [115]. Specifically *MFN1/2* facilitate outer mitochondrial membrane fusion, which can be uncoupled from inner membrane fusion in mammalian cells [113].
	⊢ Per	Unfused	As we model three distinct overall mitochondrial morphology states with two Boolean nodes we set *Unfused* = ON and *Hyperfused_OM* = ON to be mutually exclusive. Thus, a cell with *Unfused* (fragmented) must pass through a normal (*Unfused* = OFF and *Hyperfused_OM* = OFF) state before undergoing hyperfusion.
	⊢ IBind	Drp1	*Drp1* is a main driver of mitochondrial fission [116]. When recruited to mitochondria, *Drp1* dimers remodel the mitochondrial membrane, while *Drp1* multimers promote its GTPase activity and induce fission [117].
	⊢ ComplProc	PINK1	Low $\Delta\Psi$ on mitochondria undergoing mitophagy triggers the activity of the *PINK1* kinase, which recruits the ubiquitin E3 ligase *Parkin* to target both *MFN*s [107].
	⊢ Ind	Mitophagy_High	As *MFN1/2* ubiquitinated in a *PINK1/Parkin*-dependent manner upon induction of mitophagy [106], here we assume that high levels of mitophagy locally block low-$\Delta\Psi_M$ mitochondria from undergoing *MFN*-induced outer membrane fusion.
Hyperfused_IMOM	**Hyperfused_IMOM** = **Hyperfused_OM** and **MFN1_2** and not(**MP_Low** or **Unfused** or **Drp1**)		
	MSt		Fusion of the inner mitochondrial membrane requires outer membrane fusion maintained by *MFN1/2*, but blocked by active *Drp1* [116, 117] or low $\Delta\Psi_M$ [118]. In addition, we assume that cell-wide fragmentation of mitochodrtia needs to be resolved to a normal state before the transition to a connected hyperfused mitochondrial network.
	← ComplProc	Hyperfused_OM	Fusion of the outer mitochondrial membrane, maintained by *MFN1/2*, is required for *Opa1* cleavage and activation, and is thus a prerequisite for inner membrane fusion [118].
	← Compl	MFN1_2	Fusion of the outer mitochondrial membrane, maintained by *MFN1/2*, is required for *Opa1* cleavage and activation, and is thus a prerequisite for inner membrane fusion [118].

Table S1g: Mitochondria module

	$\vdash$ IBind	MP_Low	*Opa1* cleavage and activation requires OXPHOS-stimulated activation of *Yme1L*, which cleaves *Opa1* more efficiently under high OXPHOS conditions [118]. We model this by requiring normal or high $\Delta\Psi_M$ as a condition of inner membrane hyperfusion. In contrast, outter membrane fusion is insensitive to $\Delta\Psi_M$ and OXPHOS [118].
	$\vdash$ IBind	Drp1	*Drp1* is a main driver of mitochondrial fission [116, 117] and thus counteracts both inner and outer membrane fusion.
	$\vdash$ Per	Unfused	As we model three distinct overall mitochondrial morphology states with two Boolean nodes, we set *Unfused* = ON and *Hyperfused_ OM* = ON to be mutually exclusive. Thus, a cell with *Unfused* (fragmented) must pass through a normal (*Unfused* = OFF and *Hyperfused_ OM* = OFF) state before undergoing hyperfusion.
MP_High	**MP_High** = **TCA_cycle** and **Hyperfused_IMOM** and not(**MP_Low** or **Unfused** or **mROS** or **cROS** or **cROS_H** or **ROS_Ext**)		
	MP	The high $\Delta\Psi_M$ able to drive proliferation requires a funcioning TCA cycle / ETC, a hypefused network in which the inner membranes are also fused [119, 120], as well as the absence of excess mitochondrial, cytosolic, or external ROS [121].	
	$\leftarrow$ ComplProc	TCA_cycle	The TCA cycle feeds the Electron Transport Chain (ETC) by transfering electrons to NAD^+ carriers, and thus increases $\Delta\Psi_M$.
	$\leftarrow$ ComplProc	Hyperfused _IMOM	Mitochondrial inner membrane fusion raises the efficiency of OXPHOS and increases the network's $\Delta\Psi_M$ [119]. We assume that the high $\Delta\Psi_M$ required for *Cyclin E* accumulation and S-phase entry requires such hyperfusion [120].
	$\vdash$ Per	MP_Low	As we model three distinct overall $\Delta\Psi_M$ levels with two Boolean nodes, we set *MP_ High* = ON and *MP_ Low* = ON to be mutually exclusive. Thus, a cell with low $\Delta\Psi_M$ (*MP_ Low*) must pass through a state with normal $\Delta\Psi_M$ (*MP_ Low* = OFF and *MP_ High* = OFF) before achieving high $\Delta\Psi_M$.
	$\vdash$ ComplProc	Unfused	As mitochondrial inner membrane fusion raises the efficiency of OXPHOS and increases the network's $\Delta\Psi_M$ [119], we assume that a cell with fragmented overall mitochondrial morphology cannot have high $\Delta\Psi_M$.
	$\vdash$ ComplProc	mROS	Exposing mitochondria to excessive oxidative stress can push them past a threshold that triggers the opening of the mitochondrial permeability transition (MPT) pore and/or the inner membrane anion channel (IMAC). This results in the simultaneous collapse of the $\Delta\Psi_M$, and a transient increased mitochondrial ROS generation by the ETC. This mitochondria-derived ROS can be released into cytosol, where it can trigger "Reactive Oxygen Species (ROS)-induced ROS release" (RIRR) in neighboring mitochondria [121].

Table S1g: Mitochondria module

	$\vdash$ ComplProc	cROS	Excessive cytosolic ROS can push mitochondria past a threshold that triggers the opening of the MPT and/or the IMAC pore(s), which collapses the $\Delta\Psi_M$ [121].
	$\vdash$ ComplProc	cROS_H	Excessive cytosolic ROS can push mitochondria past a threshold that triggers the opening of the MPT and/or the IMAC pore(s), which collapses the $\Delta\Psi_M$ [121].
	$\vdash$ ComplProc	ROS_Ext	Excessive cytosolic ROS can push mitochondria past a threshold that triggers the opening of the MPT and/or the IMAC pore(s), which collapses the $\Delta\Psi_M$ [121].
MP_Low	**MP_Low** = **BAX** or **BAK** or **cROS_H** or (not **MP_High** and ((not **TCA_cycle** and **NADp_m**) or (**Unfused** and **mROS**) or (**Hyperfused_OM** and not **Mitophagy_High** and **mROS** and not(**MnSOD** and **SIRT3**)) or (**MP_Low** and **mROS** and not(**Mitophagy_High** or **PGC1**))))		
	MP		Low $\Delta\Psi_M$ can arise from a variety of processes: i) permeabilization of the outter membrane by *BAX/BAK* activation [122, 123] or by excessive cytosolic ROS [121], ii) lack of mitochondrial NAD$^+$ and no ongoing generation by the TCA cycle, iii) unfused ROS-producing mitochondria [124], iv) a hyperfused network barred from mitophagy [125] and damaged by ROS production, not mitigated by *MnSOD* [126] and *SIRT3* [127, 40], or v) mitochondria with low $\Delta\Psi_M$ and high ROS production, not rescued by *PGC-1*α-mediated biogenesis along with high mitophagic flux [128].
	$\leftarrow$ ComplProc	BAK	*BAK* translocation to the mitochondrial membrane can induce the loss of $\Delta\Psi_M$ [122].
	$\leftarrow$ ComplProc	BAX	*BAX* translocation to the mitochondrial membrane can induce the loss of $\Delta\Psi_M$[123].
	$\leftarrow$ ComplProc	cROS_H	Excessive cytosolic ROS can push mitochondria past a threshold that triggers the opening of the MPT and/or the IMAC pore(s), which collapses the $\Delta\Psi_M$ [121]. Over time, excessive ROS also damages mtDNA.
	$\vdash$ Per	MP_High	As we model three distinct overall $\Delta\Psi_M$ levels with two Boolean nodes, we set *MP_Low* = ON and *MP_High* = ON to be mutually exclusive. Thus, a cell with high $\Delta\Psi_M$ (*MP_High*) must pass through a state with normal $\Delta\Psi_M$ (*MP_Low* = OFF and *MP_High* = OFF) before crashing to a low $\Delta\Psi_M$.
	$\vdash$ ComplProc	TCA_cycle	The TCA cycle feeds the Electron Transport Chain (ETC) by transfering electrons to NAD$^+$. The ETC, in turn, raises $\Delta\Psi_M$.
	$\leftarrow$ ComplProc	NADp_m	As the ETC requires NADH to increase $\Delta\Psi_M$, high mitochondrial NAD$^+$ in the *absence* of ongoing TCA cycle flux is expected to crash $\Delta\Psi_M$.
	$\leftarrow$ ComplProc	Unfused	Cells with severely fragmented mitochondrial generally have low $\Delta\Psi_M$, though there is some heterogeneity among individual mitochondria [124].

Table S1g: Mitochondria module

	$\leftarrow$ ComplProc	mROS	Exposing mitochondria to excessive oxidative stress can push them past a threshold that triggers the opening of the mitochondrial permeability transition (MPT) pore and/or the inner membrane anion channel (IMAC). This results in the simultaneous collapse of the $\Delta\Psi_M$, and a transient increased mitochondrial ROS generation by the ETC.
	$\leftarrow$ ComplProc	Hyperfused _OM	As fusion of the inner membrane is needed to raise the efficiency of OXPHOS in hyperfused mitochodtia [119], we assume that outer membrane fusion in mitochondria that generate excess ROS and cannot detoxify it with the aid of both *MnSOD* and *SIRT3* become incapable of sustaining normal $\Delta\Psi_M$.
	$\vdash$ ComplProc	Mitophagy _High	High levels of mitophagy can remove damaged mitochondria and aid the reestablishment of $\Delta\Psi_M$ within its nominal range [125].
	$\vdash$ ComplProc	MnSOD	*MnSOD* detoxifies the superoxide anions ($O_2^{\cdot-}$) generated by the ETC by converting them to H_2O_2, which is further reduced to water by *Gpx-1*. Mitochondria from mice heterozygous for *MnSOD* show increased proton leak, inhibition of respiration, and early and rapid accumulation of mitochondrial oxidative damage [126], while complete loss of *MnSOD* is embryonic lethal [129].
	$\vdash$ ComplProc	SIRT3	*SIRT3* activation increases oxidative phosphorylation [127]. It deacetylates several critical enzymes involved the TCA cycle (*IDH2*) and OXPHOS (*SDHB*, *complex I*, *II* and *V*), boosting their activity [40].
	$\leftarrow$ Per	MP_Low	We assume that low $\Delta\Psi_M$ in mitochondria with excess mROS does not recover in the absence of high levels of mitophagy, or *PGC-1α*-mediated biogenesis.
	$\vdash$ ComplProc	PGC1	*PGC-1α* works together with *NRF1/2* and mitochondrial transcription factor A (*TFAM*) to increase mitochondrial biogenesis and boost mitochondrial energy metabolism [128].
ATP_Low	**ATP_Low** = not **MP_Low** or (**Glycolysis** and **Fermentation**) or (**Autophagy** and **PGC1**)		
	Met		The *ATP_Low* node in our model represents an ATP/ADP ratio required for avoiding necrotic cell death [130] (not modeled), but not sufficient to keep AMPK inactive. This requires ATP production by OXPHOS (i.e., at least normal $\Delta\Psi_M$), glycolysis coupled to fermentation, or autophagy coupled with *PGC-1α* activity.
	$\vdash$ ComplProc	MP_Low	We assume that mitochondria with cell-wide low $\Delta\Psi_M$ do not contribute to ATP production.
	$\leftarrow$ ComplProc	Glycolysis	In the absence of normal $\Delta\Psi_M$, glycolysis coupled with fermentation can support ATP production.
	$\leftarrow$ ComplProc	Fermentation	In the absence of normal $\Delta\Psi_M$, glycolysis coupled with fermentation can support ATP production.

Table S1g: Mitochondria module

	← ComplProc	Autophagy	The process of autophagy breaks down existing cytoplasmic components to support ATP production under starvation [131, 132].
	← ComplProc	PGC1	*PGC-1*α induces mitochondrial biogenesis [133], as well as mitochondrial fatty acid oxidation to boost ATP levels under fasting conditions [134].
ATP	**ATP** = **ATP_Low** and ((not **MP_Low**) or (**Glycolysis_H** and **Fermentation**))		
	Met	The *ATP* node represents normal ATP/ADP ratios that keep AMPK inactive. While at least low ATP (*ATP_Low* node) is a prerequisite, maintaining nominal ATP levels also requires normal $\Delta\Psi_M$, or high levels of glycolysis coupled to fermentation.	
	← Per	ATP_Low	As the *ATP* node represents normal ATP/ADP ratios, we designed the model suych that it cannot be ON while the node representing survival-level ATP (*ATP_Low*) is OFF.
	⊣ ComplProc	MP_Low	We assume that mitochondria with cell-wide low $\Delta\Psi_M$ do not contribute to ATP production.
	← ComplProc	Glycolysis_H	High levels of glycolysis coupled with fermentation can support normal ATP production.
	← ComplProc	Fermentation	High levels of glycolysis coupled with fermentation can support normal ATP production.
ATP_H	**ATP_H** = **ATP** and ((not **MP_Low** and **MP_High** and **Hyperfused_IMOM**) or (**Glycolysis_H** and **Fermentation** and **Pyruvate_Ext**))		
	Met	The *ATP_High* node represents the increased ATP/ADP ratios required to enter S-phase [120]. While at least normal ATP (*ATP* node) is a prerequisite, maintaining high ATP levels also requires high $\Delta\Psi_M$ maintained by a hyperfused network of mitochondria [120], or high levels of glycolysis coupled to fermentation supported by excess external pyruvate.	
	← Per	ATP	As the *ATP_High* node represents high ATP/ADP ratios, we designed the model suych that it cannot be ON while the node representing normal ATP levels (*ATP*) is OFF.
	⊣ ComplProc	MP_Low	We assume that mitochondria with cell-wide low $\Delta\Psi_M$ do not contribute to ATP production.
	← ComplProc	MP_High	Cell-wide high $\Delta\Psi_M$ can produce the high ATP levels required for S-phase entry [120].
	← ComplProc	Hyperfused_IMOM	A hyperfused mitochondrial state is required for the elevated ATP production leading to S-phase entry [120]. As inner membrane fusion does not occur at low $\Delta\Psi_M$ and helps raise $\Delta\Psi_M$ [119], we assume this hyperfusion invovles both membranes.
	← ComplProc	Glycolysis_H	Culturing cells with compromised mitochondria in excess pyruvate rescues cell cycle progression [135]. We assume that conversion of excess pyruvate to lactate can maintain the cytosolic NAD^+/NADH ratios required to support high levels of glycolysis, which in turn can raise ATP levels to promote S-phase entry even in teh absence of high $\Delta\Psi_M$.

Table S1g: Mitochondria module

	← ComplProc	Fermentation	The conversion of pyruvate to lactate regenerates cytosolic NAD^+, required to maintain high rates of glycolysis. When mitochondrial NAD^+ production (NADH use) is compromised by the lack of an active ETC, the cell's NADH shuttles saturate and fermentation is used to regenerate NAD^+ [136].
	← ComplProc	Pyruvate_Ext	Culturing cells with compromised mitochondria in excess pyruvate rescues cell cycle progression [135], presumably by allowing rapid NAD^+ generation to support high levels of glycolysis.
AMPK	**AMPK** = not(**ATP_H** or **MP_High**) and (not **ATP** or **mROS** or **cROS** or **cROS_H**)		
	K		The cellular energy sensor AMP-activated protein kinase (*AMPK*) is activated by AMP binding in cells with low ATP/AMP ratios, leading to its allosteric activation and Thr172 phosphorylation by *LKB1* kinase [137, 1]. Thus, we assume *AMPK* blocked by high ATP levels or high $\Delta\Psi_M$, while the lack of normal ATP levels activates it. In addition, mitochondrial and cellular ROS was also shown to activate *AMPK*, promoting its antioxidant functions[138].
	⊣ Ind	ATP_H	High ATP to AMP rations inhibit AMP binding to *AMPK*, blocking its allosteric activation and Thr172 phosphorylation [139].
	⊣ Ind	MP_High	High $\Delta\Psi_M$ can block *AMPK* localized to the mitochondrial outer membrane via a localized ATP boost [140].
	⊣ Ind	ATP	We assume that nominal ATP to AMP rations keep AMP binding to *AMPK* low, blocking its allosteric activation and Thr172 phosphorylation [139].
	← Loc	mROS	Mitochondrial ROS was shown to directly activate *AMPK*, triggering a *PGC-1*α-dependent antioxidant response to limit mitochondrial ROS [138].
	← Ind	cROS	Cells cultured in Trolox, an antioxidant that reduces cellular ROS levels displayed reduced AMPK activation (Thr172 phosphorylation) [138].
	← Ind	cROS_H	Cells cultured in Trolox, an antioxidant that reduces cellular ROS levels displayed reduced AMPK activation (Thr172 phosphorylation) [138].
PGC1	**PGC1** = **AMPK**		
	TF		Peroxisome proliferator-activated receptor-gamma coactivator *PGC-1*α is a transcription coactivator with key roles in the regulation of energy metabolism and antioxidant response [141]. In our model, activation of *PGC-1*α requires *AMPK*, or a joint boost from *mTORC1* [142] and *SIRT3* deacetylation [40]. (Note that we choose to omit *mTORC1*-mediated *PGC-1*α activation, as this would force our model to activate *MFN1/2* any time *mTORC1* was active, a response that does not match observed cell behavior. Overall, it appears that *mTORC1* balances the induction of mitochondrial biogenesis downstream of *PGC-1*α with increased fission, rendering the network more dynamic but not hyperfused.)
	← Ind	AMPK	Increased AMPK levels increase *PGC-1*α mRNA and protein levels [143, 144].

Table S1g: Mitochondria module

SIRT3	**SIRT3** = **NADp_m** and (**PGC1** or (**Hyperfused_IMOM** and not **MP_Low**))		
	Enz		The mitochondrial NAD-dependent deacetylase *SIRT3* helps maintain ATP levels by regulating mitochondrial electron transport [127], boosting antioxidant responses via deacetylaiton of *MnSOD*, *NRF2* and *PGC-1α* (which also promotes mitochondrial biogenesis) [40]. In our model, *SIRT3* activation requires mitochondrial NAD^+ and either *PGC-1α*-mediated transcription [145] or mitochondrial hyperfusion with at least nominal $\Delta\Psi_M$. (Note that we do not model the localized homeostatic role of *SIRT3*, where it its activity is locally boosted by the lowering of $\Delta\Psi_M$ in order cause rapid deacetylation of matrix proteins to recover membrane potential [146]. This effect, however, implicitly requires no drastic loss of mitochodtial NAD^+, and thus cannot be a lasting condition.)
	← Cat	NADp_m	*SIRT3* activity requires mitochondrial NAD^+ [127].
	← TR	PGC1	*PGC-1α* is a direct transcriptional inducer of *SIRT3* [145].
	← ComplProc	Hyperfused_IMOM	In the absence of a *PGC-1α*-mediated increase in *SIRT3* levels, we assume that a hyperfused mitochondrial network with strong ETC activity and at least normal $\Delta\Psi_M$ are required to keep mitochondrial NAD^+ levels high for sustained *SIRT3* activity.
	⊢ ComplProc	MP_Low	In the absence of a *PGC-1α*-mediated increase in *SIRT3* levels, we assume that a hyperfused mitochondrial network with strong ETC activity and at least normal $\Delta\Psi_M$ are required to keep mitochondrial NAD^+ levels high for sustained *SIRT3* activity.
PINK1	**PINK1** = **MP_Low** and (**NF_kB** or **FoxO1** or **FoxO3**)		
	K		PTEN-induced putative kinase 1 *PINK1* phosphorylates ubiquitin to activate the ubiquitin ligase *Parkin*, which builds ubiquitin chains on mitochondrial outer membrane proteins to initiate mitophagy [147]. *PINK1* is activated by mitochondrial membrane depolarization [148], while its transcriptional activation (for increased levels of mitophagy modeled here) is induced by *NF-κB* or *FoxO1/3* [46, 149].
	← Ind	MP_Low	*PINK1* is activated by mitochondrial membrane depolarization and stimulates Parkin E3 ligase activity by phosphorylating it at Ser65 [148].
	← TR	NF_kB	*NF-κB* is a direct transcriptional inducer of *PINK1*[150].
	← TR	FoxO1	*FoxO1* is a transcriptional inducer of *PINK1* [46].
	← TR	FoxO3	*FoxO3* is a transcriptional inducer of *PINK1* [149].
Mitophagy_High	**Mitophagy_High** = (**PINK1** or **Drp1**) and not **Hyperfused_OM** and (**AMPK** or **MP_Low**) and (**FoxO3** or **FoxO1** or not **mTORC1**) and not **Casp8**		

Table S1g: Mitochondria module

	Proc		In out model, high (above-basal) mitophagy requires either *PINK1/Parkin* [147] or *Drp1* activation [151], lack of cell-wide hyperfusion of the outer mitochondrial membrane, either *AMPK* [152] or low $\Delta\Psi_M$ [153], and transcriptional upregulation of autophagy/mitophagy components by *FoxO1/3* [154] or *mTORC1* inhibition [46]. Finally, *Caspase 8* blocks high mitophagy by inhibiting *Parkin* [155].
	← Ind	PINK1	*PINK1* phosphorylates ubiquitin to activate *Parkin*, which tags mitochondrial outer membrane proteins with ubiquitin chains to initiate mitophagy [147].
	← Ind	Drp1	In addition to *PINK1/Parkin*, mitophagy can also be initiated by *Drp1* [151].
	⊢ Ind	Hyperfused_OM	mitochondrial hyperfusion in the absence of *Drp1*-mediated fission protects mitochondria from autophagosomal degradation [156, 157].
	← Ind	AMPK	*AMPK* promotes mitophagy by enhancing mitochondrial fission (*MFF* phosphorylation) and autophagosomal engulfment (*TBK1* activation). This effect does not require *PINK1/Parkin* [152].
	← Ind	MP_Low	Loss of $\Delta\Psi_M$ rapidly recruits *Parkin* from the cytosol to mitochondria, boosting mitophagy [153].
	← Ind	FoxO1	*FoxO1/3* regulate both ubiquitin-dependent and receptor-mediated mitophagy by upregulating *PINK1* and *Bnip3/Bnip3L*, respectively. In addition, *FoxO1/3* induce genes involved in each stage of autophagosome formation, including initiation (e.g. *Ulk1/Ulk2*), nucleation (e.g. *Becn1*, *Atg14*, and *Pi3kIII*), elongation (e.g. *Map1lc3b*, *Gabarapl,Atg4/5/12*), and fusion (e.g. *Tfeb* and *Rab7*) [154].
	← Ind	FoxO3	*FoxO1/3* regulate both ubiquitin-dependent and receptor-mediated mitophagy, as well as each stage of autophagosome formation [154].
	⊢ Ind	mTORC1	Constitutively active *mTORC1* in *TSC2*-/- cells impairs mitophagy, while its inhibition reestablishes it [46].
	⊢ Ind	Casp8	*Caspase 8* cleaves and inhibits *Parkin* during apoptosis, a key player in the initiation of mitophagy [158]. Decreased *Parkin* levels block mitophagy and enhance apoptosis [155].
Drp1	**Drp1** = (**BIK** and (**BAK** or **BAX**)) or (not **p53** and ((**CyclinB** and **Cdk1** and not **Cdh1**) or (**AMPK** and not **MFN1_2**) or (**MP_Low** and **PINK1** and **Mitophagy_High**)))		
	GTPa		Dynamin-related protein 1 *Drp1* is required for mitochondrial fission [151]. *Drp1* is recruited to the mitochondrial outer membrane by several stimuli, including apoptosis (via *BIK*-mediated recruitment [159] and *BAK/BAX*-mediated retention [160]), *Cdk1/Cyclin B*-induced mitotic phosphorylation [161] counteracted by APC/C^{Cdh1} [162, 163], or low $\Delta\Psi_M$ [164] coupled to mitophagy [165, 166, 167] and aided by *AMPK* [168]. In contrast, *p53* inhibits *Drp1* activity [169].

Table S1g: Mitochondria module

← Ind	BIK	*BIK* activates the transmission of Ca(2+) to the mitochondria from the ER, which in turn recruits *Drp1* to the mitochondrial surface [159].
← Ind	BAK	*BAK* translocation to the mitochondria increases mitochondria-bound *Drp1* by sumoylation of *Drp1*, which subsequently accumulates on mitochondrial membranes after the completion of fission, but before the initiation of cytochrome C release [160].
← Loc	BAX	*BAX* translocation to the mitochondria increases mitochondria-bound *Drp1* by sumoylation of *Drp1*, which subsequently accumulates on mitochondrial membranes after the completion of fission, but before the initiation of cytochrome C release [160].
⊢ Ind	p53	*p53* increased inhibitory phosphorylation of *Drp1* at Ser637, an effect mediated by Protein kinase A but did not require increases *p21* [169].
← P	CyclinB	*Drp1* is phosphorylated on Ser585 by *Cdk1/Cyclin B* complexes on during mitosis, which is required for effective mitochondrial fragmentation during mitosis [161].
← P	Cdk1	*Drp1* is phosphorylated on Ser585 by *Cdk1/Cyclin B* complexes on during mitosis, which is required for effective mitochondrial fragmentation during mitosis [161].
⊢ Deg	Cdh1	At the end of mitosis, APC/C^{Cdh1} ubiquitylates *Drp1*, leading to its degradation [162, 163].
← Ind	AMPK	Under energy stress, *AMPK* phosphorylates *DRP1*'s mitochondrial outer-membrane receptor, fission factor *MFF*, aiding the translocation of *DRP1* from the cytosol to the mitochondrial membrane [163]. That said, the long-term indirect effects of sustained *AMPK* activation likely counteract the rapid fragmentation caused by this event, as *AMPK* activation was also linked to a shift in *Drp1* phosphorylation from Ser616, which accelerated its recruitment to the mitochondrial membrane, to Ser637, which hinders it [168].
← Ind	MP_Low	*Drp1* recruitment to damaged mitochondria is mediated by a $\Delta\Psi_M$ 'tipping point', below which mitochondria with functional *Drp1* undergo rapid fission [164].
← P	PINK1	*PINK1* directly phosphorylates *Drp1* on S616, which accelerated its recruitment to the mitochondrial membrane [165].
← Ind	Mitophagy_High	Mitophagy results in the translocation of mitochondrial E3 ubiquitin ligase *MITOL/March5* from depolarized mitochondria to peroxisomes [166]. This potentiates *Drp1* function, as *MITOL/March5* mediates *Drp1* clearance from the mitochondrial membrane via by outer mitochondrial membrane-associated degradation (OMMAD) [167].

Table S1g: Mitochondria module

	⊣ Ind	MFN1_2	We assume that in the absence of additional influences beyond *AMPK*-mediated *Drp1* activation, high levels of *MDN1/2* balance out increased *Drp1* activity.
Unfused	**Unfused** = not **Hyperfused_OM** and ((**Drp1** and not(**MFN1_2** and **Mitophagy_High**)) or **BAK** or **BAX**)		
	MSt		Our model captures overall (cell-wide) mitochondrial morphology in three stages. First, the fragmented state is represented by the *Unfused* = ON node state. Second, the "normal" morphology of a quiescent cell is represented by *Unfused* = OFF and *Hyperfused_OM* = OFF, while the third, hyperfused state is marked by *Hyperfused_OM* = ON. (We further distinguish between outer membrane vs. outer and inner membrane fusion).
	⊣ Per	Hyperfused _OM	As we model three distinct overall mitochondrial morphology states with two Boolean nodes, we set *Unfused* = ON and *Hyperfused_OM* = ON to be mutually exclusive. Thus, a cell with hyperfused mitochondria must pass through a normal (*Unfused* = OFF and *Hyperfused_OM* = OFF) state before fully fragmenting.
	← ComplProc	Drp1	*Drp1* is required for mitochondrial fission [151].
	⊣ ComplProc	MFN1_2	*MFN1/2* promote mitochondrial fusion, thus reducing the fraction of unfused mitochondria [113].
	⊣ Deg	Mitophagy _High	As mitophagy targets fragmented and damaged mitochondria, we assume that high levels of mitophagy act to reduce the fraction of unfused, damaged mitochondria.
	← Ind	BAK	During *BAK/BAX*-dependent apoptosis, the mitochondrial network undergoes extensive fragmentation even in *DRP1*-null cells [170]. Thus, we assume that *BAK/BAX* alone can drive excessive fragmentation.
	← Ind	BAX	During *BAK/BAX*-dependent apoptosis, the mitochondrial network undergoes extensive fragmentation even in *DRP1*-null cells [170]. Thus, we assume that *BAK/BAX* alone can drive excessive fragmentation.
mROS	**mROS** = **cROS_H** or (**Unfused** and (not(**MnSOD** or **SIRT3**) or **MP_Low** or **BAK** or **BAX**)) or (**MP_Low** and not(**PGC1** and (**Mitophagy_High** or **MnSOD** or **SIRT3**))) or (**Hyperfused_OM** and ((**MP_High** or **mROS**) and not(**MnSOD** and **SIRT3**)))		

Table S1g: Mitochondria module

Met	Mitochondria are the main site of cellular ROS production [171]. In our model, we assume that this production exceeds the normal parameters of healthy mitochondrial function (including healthy proliferation) if one of the following conditions hold: i) cytosolic ROS levels are very high [172], ii) the mitochondrial network is fragmented (cell-wide) and is either missing both *MnSOD* and *SIRT3*, or has undergone *BAK/BAX*-mediated mitochondrial outer membrane permeabilization (MOMP) [173, 174, 126, 40, 175, 176]; iii) $\Delta\Psi_M$ is low, not counteracted by ongoing high mitophagy coupled with mitochondrial biogenesis (requiring *PGC-1α* activity), or by increased antioxidant *MnSOD* or *SIRT3* activity [177, 178, 179, 180]; iv) the mitochondrial network is hyperfused, with either high $\Delta\Psi_M$ (higher ROS production by the ETC) [181] or dysnfunctionally low $\Delta\Psi_M$ [182, 177]; both in the absence of the joint mitigating effects of increased *MnSOD* and *SIRT3* activity.	
← ComplProc	cROS_H	High levels of cytosolic ROS (e.g. hydrogen peroxide) can damage the ETC by triggering a mitochondrial permeability transition, which leads to mitochondrial complex I deficiency. As a result, the damaged ETC produces more superoxide [172].
← ComplProc	Unfused	Mitochondrial fragmentation mediated by fission increases ROS production [173, 174].
⊢ Cat	MnSOD	*MnSOD* transforms the toxic superoxide generated by the mitochondrial ETC into hydrogen peroxide (further transformed to water by catalase) and diatomic oxygen [126], and thus it is required for lowering mitochondrial ROS.
⊢ Ind	SIRT3	*SIRT3* boosts mitochondrial antioxidant responses by deacetylating components of the ETC to reduce ROS generation [127], as well as indirectly via deacetylaiton of *MnSOD*, *NRF2* and *PGC-1α* (which also promotes mitochondrial biogenesis) [40].
← ComplProc	BAK	*SIRT3* Mitochondrial outer membrane permeabilization (MOMP) by oligomerization of *BAK/BAX* was shown to increase ROS (superoxide) release along with Cytochrome C [175, 176].
← ComplProc	BAX	*SIRT3* Mitochondrial outer membrane permeabilization (MOMP) by oligomerization of *BAK/BAX* was shown to increase ROS (superoxide) release along with Cytochrome C [175, 176].
← ComplProc	MP_Low	Mitochondrial dysfunction, or the inability to maintain normal $\Delta\Psi_M$, is associated with increased mitochondrial ROS production [182, 177]. (Note that *transient* $\Delta\Psi_M$ reduction can shift the electron transport chain (ETC) into a more oxidized state and can temporarily reduce ROS production, but sustained cell-wide low $\Delta\Psi_M$ appears to increase mROS [177].)
⊢ ComplProc	Mitophagy _High	As mitophagy targets damaged and dysfunctional mitochondria, high levels of mitophagy can lower overall mROS generation by selectively clearing the most prominent ROS producers [178, 179].

Table S1g: Mitochondria module

	$\dashv$ ComplProc	PGC1	*PGC-1α* is a master transcriptional regulator of mitochondrial biogenesis, oxidative phosphorylation and reactive oxygen species detoxification [180]. *PGC-1α* regulates the expression of mitochondrial antioxidant genes (*MnSOD*, catalase, peroxiredoxin 3 and 5, uncoupling protein 2, thioredoxin 2, and thioredoxin reductase) and lowers mROS production [128, 141].
	$\leftarrow$ ComplProc	Hyperfused_OM	Hyperfused mitochondria can produce excess ROS under two conditions. First, if $\Delta\Psi_M$ is very high (G1/S transition), the ETC produces excess ROS that must be counterbalanced by ROS scavenging enzymes (*MnSOD*, catalase, peroxiredoxins, thioredoxins, and thioredoxin reductases) [181]. Alternately, mROS is maintained if $\Delta\Psi_M$ is very low [182, 177], the mitochondrial network is already producing excess mROS, and the inner membrane cannot fuse to boost $\Delta\Psi_M$ [118].
	$\leftarrow$ ComplProc	MP_High	High $\Delta\Psi_M$ above a certain threshold ($\sim$ 140 mV) dramatically increases increase H_2O_2 production [181].
	$\leftarrow$ Per	mROS	mROS damages ETC components, leading to dysfunction and further mROS generation. Moreover, exposing mitochondria to excessive oxidative stress can push them past a threshold that triggers the opening of the mitochondrial permeability transition (MPT) pore and/or the inner membrane anion channel (IMAC). This results in the simultaneous collapse of the $\Delta\Psi_M$, and a transient increased mitochondrial ROS generation by the ETC. This mitochondria-derived ROS can be released into cytosol, where it can trigger "Reactive Oxygen Species (ROS)-induced ROS release" (RIRR) in neighboring mitochondria [121].
NADp_m	**NADp_m** = (not(**TCA_cycle** and **MP_Low**) and (**MP_High** or (**NADp_c** and not **mROS**))) or (**Pyruvate_Ext** and **NADp_c**)		
	Met		Mitochodtial NAD^+ is consumed by the TCA cycle and generated by the ETC [183]. Thus, mitochondrial NAD^+ in our model turns off if the TCA cycle is functional but the ETC is not (represented by *MP_Low* = ON). Along these lines, maintaining it requires no mROS damage to the ETC [184], and either high $\Delta\Psi_M$ [183] or normal cytosolic NAD^+. Alternatively, we assume that the presence of excess pyruvate can raise the cytosolic pool of NAD^+ to the point where the malate-aspartate shuttle slows to a halt [88], allowing mitochondria to recover their NAD^+ levels.
	$\dashv$ Ind	TCA_cycle	The TCA cycle consumes NAD^+ to generate NADH, which in turn carries eletrons to the ETC [183].
	$\dashv$ Ind	MP_Low	The ETC's Complex I regenerates mitochondrial NAD^+ as it pumps an electron into the intermembranespace to increase the $\Delta\Psi_M$ [183]. We assume that the ETC is not working effectively in cells with low $\Delta\Psi_M$.

Table S1g: Mitochondria module

← Loc	MP_High	The ETC's Complex I regenerates mitochondrial NAD^+ as it pumps an electron into the intermembranespace to increase the $\Delta\Psi_M$ [183]. Thus, high $\Delta\Psi_M$ generation is coupled with mitochondrial NAD^+ production.
← Loc	NADp_c	As the net effect of the malate-aspartate shuttle is to oxydise cytosolic NADH to NAD^+ at the expence of reducing mitochondrial NAD^+ to NADH, this shuttle runs one-way: it replenishes cytosolic NAD^+ from the mitochondrial pool. Here we assume that if the cytosolic levels are high, the shuttle works slowly and allows mitochondrial NAD^+ levels to build up by de novo production of NAD^+ [185].
⊢ Ind	mROS	mROS damages ETC compontents as well as mitochodrial DNA, blocking their ability to regenerate NAD^+ used up by the TCA cycle. Damage to Complex I in particular lowers mitochodrial NAD^+, which in turn blocks deacetlylation of *MnSOD* and further increases mROS [184].
← Ind	Pyruvate _Ext	We assume that the increased pyruvate to lactate conversion triggered by access to external pyruvate increases cytosolic NAD^+ levels, allowign the malate-aspartate shuttle to slow and help mitochondrial NAD^+ levels to build up by de novo production of NAD^+ [185].

Table S1h: Restriction_SW module

Target Node	Node Gate<br>Node Type<br>Link Type	Node Description<br>Input Node	Link Description
p21	**p21** = **p21_mRNA** and (not **CyclinE** or **p53**) and not **Casp3**		
	Prot	In this model, the *p21* node corresponds to nuclear p21 in cells with relatively high basal *p21* activity. *p21* activity and or localization can be lowered by loss of *FoxO* mediated transcription (see $p21_{mRNA}$ node) and via feedback from *Cyclin E/Cdk2* [186].	
	← TL	p21 _mRNA	*p21* protein activity requires the presence of *p21* transcription.
	← TR	p53	*p53*-mediated transcription of *p21* raises its levels sufficiently to override its repression by *Cdk2/Cyclin E*, resetting the switch to a high *p21*/low *Cdk2* state [187].
	⊢ Deg	CyclinE	*p21* and *Cyclin E/Cdk2* form a positive (double-negative) feedback loop in which *Cyclin E/Cdk2* activates the *SCF/Skp2* complex responsible for the degradation of *Cyclin E/Cdk2*-bound, phosphorylated *p21* [188]. *p21*, in turn, not only blocks *Cyclin E/Cdk2* activity, but it also inhibits *Cyclin D1*. Thus, *p21* interferes with the mitogen signal that turns on *Cyclin E* in the first place. In quiescent cells with high basal *p21* levels, this positive feedback renders cell cycle entry stochastic [186].

Table S1h: Restriction_SW module

	⊢ Lysis	Casp3	*Caspase 3* cleaves and deactivates *p21* [189].
pRB	**pRB** = (**p27Kip1** or not **CyclinE**) and not(**CyclinD1** or **CyclinA** or **Casp3**)		
	TF	*pRB* is active in the absence of *Caspase 3*, *Cyclin D1*, *Cyclin A*, and *Cyclin E*. In addition, *pRB* maintains its activity when active $p27^{Kip1}$ counteracts the effects of *Cyclin E* [190, 191, 192, 193].	
	← ComplProc	p27Kip1	Active $p27^{Kip1}$ can counteract the inhibitory effects of active *CyclinE/Cdk2* complexes [190].
	⊢ P	CyclinE	*Cyclin E/Cdk2* complexes bind and phosphorylate *RB*, inhibiting its activity [194, 192].
	⊢ P	CyclinD1	*Cyclin D1/Cdk4,6* complexes bind and phosphorylate *RB*, inhibiting its activity [195, 196, 192].
	⊢ P	CyclinA	*Cyclin A/Cdk1,2* complexes phosphorylate and deactivate *RB* [191].
	⊢ Lysis	Casp3	*Caspase 3* cleaves *RB*, generating fragments that do not associate with *E2F1*, rendering *RB* inactive [197].
p27Kip1	**p27Kip1** = not **CyclinD1** and ((not **CyclinE** and **FoxO3** and **FoxO1**) or (not **CyclinA** and (**FoxO3** or **FoxO1**)) or not(**CyclinE** or **CyclinA**) or **AMPK**) and (not(**Cdk1** and **CyclinB**) or **AMPK**) and not **Casp3**		
	Prot	Active $p27^{Kip1}$ is cleaved by *Caspase 3* and inhibited (sequestered) by *Cyclin D1/Cdk4,6* [190] or *Cyclin B/Cdk1* [198]. In addition, maintenance of $p27^{Kip1}$ requires one or both *FoxO* factors when sequestered by *Cyclin E/Cdk2* (one *FoxO* factor) or *Cyclin A/Cdk2* (both *FoxO* factors), but it cannot keep pace with the simultaneous activity of *Cyclin E/Cdk2* and *Cyclin A/Cdk2* [199] unless stabilized by *AMPK* [200].	
	⊢ IBind	CyclinD1	Active *Cyclin D/Cdk4,6* complexes competitively bind to $p27^{Kip1}$ and progressively inhibit its ability to keep *Cyclin-E/Cdk2* inactive, thereby inducing cdk2 activity and cell-cycle progression [190].
	⊢ Deg	CyclinE	Active *Cyclin-E/Cdk2* phosphorylate $p27^{Kip1}$ at threonine 187 (Thr187) [201], which marks it for degradation by the SCF^{SKP2} complex at the onset of S-phase [202]. (*Cyclin-E/Cdk2* complexes remain active in the presence of $p27^{Kip1}$ and promote its degradation when *Cyclin-A* is also active.)
	← TR	FoxO3	*FoxO* factors are direct inducers of $p27^{Kip1}$ expression [203].
	← TR	FoxO1	*FoxO* factors are direct inducers of $p27^{Kip1}$ expression [203].
	⊢ Deg	CyclinA	*Cyclin A/Cdk2* complexes bind and inactivate $p27^{Kip1}$ by sequestration, phosphorylate it, and promote its degradation [204].
	⊢ P	AMPK	*AMPK*-mediated $p27^{Kip1}$ phosphorylation at Thr-198 stabilizes $p27^{Kip1}$ [200].

Table S1h: Restriction_SW module

	⊢ PLoc	CyclinB	*Cyclin B/Cdk1* complexes phosphorylate $p27^{Kip1}$ [204], and although they do not promote its degradation, phosphorylated $p27^{Kip1}$ is exported from the nuclear compartment and looses its ability to inhibit *Cdk* activity [198].
	⊢ PLoc	Cdk1	*Cyclin B/Cdk1* complexes phosphorylate $p27^{Kip1}$ [204], and although they do not promote its degradation, phosphorylated $p27^{Kip1}$ is exported from the nuclear compartment and looses its ability to inhibit *Cdk* activity [198].
	⊢ Lysis	Casp3	*Caspase 3* cleaves $p27^{Kip1}$ [205]; the cleaved fragments can no longer associate with *Cdk2* / *Cyclin* complexes [206].
Myc	**Myc** = not **p53** and ((**ERK** and (**eIF4E** or not **GSK3**)) or (**E2F1** and not **pRB** and (**eIF4E** or **ERK** or not **GSK3**)))		
	TF		*Myc* activity is turned on by stabilization of the protein via *ERK* phosphorylation, aided by either an increase in translation initiated by *eIF4E* or loss of degradation-promoting phosphorylation by *GSK3β* [207]. Alternatively, increased transcription by *E2F1* can also promote *Myc* accumulation in the absence of active *pRB* [208], provided that the protein is stabilized by *ERK*, *eIF4E*, or the absence of *GSK3β*. *p53* blocks transcription of *c-Myc*, enforcing G1 arrest independently of *p21* [209].
	⊢ TR	p53	*p53* is a direct transcriptional repressor of the *c-Myc* promoter [209].
	← P	ERK	Ser-62 phosphorylation by *ERK* increases its half life, leading to *Myc* accumulation [207, 210].
	← TL	eIF4E	Increased translational initiation in the presence of activated *eIF4E* leads to an increase in *Myc* protein levels [211].
	⊢ P	GSK3	Thr-58 phosphorylation by *GSK-3* promotes *Myc* degradation [207, 212].
	⊢ TR	pRB	*E2F1*'s ability to induce *Myc* is blocked by active (hypo-phosphorylated *pRB*) [213].
	← TR	E2F1	*E2F1* binds and activates the *c-Myc* promoter [214, 215].
CyclinD1	**CyclinD1** = not(**ATR** or **ATM** or **p21_H** or (**p21** and **p53**)) and ((not **p21** and ((not **GSK3** and **eIF4E** and (**Myc** or **E2F1**)) or (**Myc** and **CyclinD1**) or (**Myc** and **E2F1**) or (**E2F1** and **CyclinD1**))) or ((not **pRB** and **E2F1**) and ((**Myc** and **CyclinD1**) or (**Myc** and (not **GSK3** or **eIF4E**)) or (**CyclinD1** and (not **GSK3** or **eIF4E**)))))		

Table S1h: Restriction_SW module

PC		Ongoing DNA synthesis keeps the *ATR* kinase active, which inhibits *Cyclin D1*. Similarly, double-strand DNA damage activates *ATM*, which also inhibits *Cyclin D1*. The precise regulatory logic of *Cyclin D1* as a function of transcriptional control by *Myc* and *E2F1*, combined with the regulation of its protein stability / activity by *GSK3β* / *p21* / *p53* is not known. Here, we assume that in the absence of *p21* (once *p21* levels drop due to growth factor signals and/or *Cdk2* activation), *Cyclin D1* can be activated by either *Myc* or *E2F1* in the absence of *GSK3β*. In the presence of *GSK3β*, we assume that *Cyclin D1* can be induced by the combined action of both *Myc* and *E2F1* [216], but sustained in an ON state by either. In the presence of basal (normal quiescent) levels of *p21*, we assume that *Cyclin D1* transcription requires *E2F1* unencumbered by *pRB*, as well as any two of the following: *Myc*, already active *Cyclin D1*, and no *GSK3β*.
← TL	eIF4E	Increased translational initiation in the presence of activated *eIF4E* leads to an increase in *CyclinD1* protein levels [217].
⊢ P	GSK3	*GSK-3β* phosphorylates *Cyclin D1* on Thr-286, promoting its ubiquitination and degradation [218].
⊢ IBind	p21	$p21^{Cip1}$ is a Cyclin Dependent kinase inhibitor which binds to and blocks the activity of *Cdk2*, *Cdk3*, *Cdk4* and *Cdk6* kinases [219] and thus inhibits *CyclinD1/Cdk4,6* [220].
⊢ TR	pRB	*E2F1*'s ability to induce *Cyclin D1* is blocked by active (hypophosphorylated) *RB* protein [221].
← TR	Myc	Extracellular growth signals activate the MAPK pathway, leading to transcriptional activation of *Cyclin D1* by *Myc* [222, 223]. *Myc* overexpression leads to rapid *Cyclin D1* induction and subsequent cell cycle entry [224], while its absence halves *Cyclin D1* levels [225]. In addition, *Myc* induces *Cdk4*, aiding the assembly of active *Cyclin D1 / Cdk4,6* complexes [225, 226].
← Per	CyclinD1	In order to take into account both production and stability of *Cyclin D1*, we assumed that the presence of active *CyclinD/Cdk2,4* complexes renders transcriptional maintenance of their levels easier.
← TR	E2F1	The *Cyclin D1* promoter is bound by *E2F* factors including *E2F1* [227], and *E2F1* overexpression can increase *Cyclin D1* (though its effects are context-dependent, as *E2F1* overexpression can also lead to apoptosis) [227]. Dominant negative *E2F1* overexpression results in a 2-3 fold decrease in *Cyclin D* expression and *Cyclin D/Cdk4,6* activity [228].
⊢ P	ATR	During replication, checkpoint kinases such as *ATR* (active during normal DNA synthesis) suppress *Cyclin D1* by promoting it's Thr286 phosphorylation, leading to its degradation [229].
⊢ P	ATM	ATM to promotes cyclin D1 Thr286 phosphorylation, leading to its degradation [229].

Table S1h: Restriction_SW module

	⊣ Ind	p53	*p53* represses *Cyclin D1* transcription by facilitating the recruitment of *HDAC1* to its promoter and thus promoting its deacetylation [230].
	⊣ IBind	p21_H	*p21^{Cip1}* is a Cyclin Dependent kinase inhibitor which binds to and blocks the activity of *Cdk2*, *Cdk3*, *Cdk4* and *Cdk6* kinases [219] and thus inhibits *CyclinD1/Cdk4,6* [220].
E2F1	**E2F1** = (**Myc** or **E2F1**) and not(**pRB** or **CyclinA** or **CAD**)		
	TF		In the absence of both *CyclinA* and *pRB*, *E2F1* transcription can be induced by *Myc* or maintained by active *E2F1*. *CAD* deactivates *E2F1* as it destroys the cell's DNA.
	← TR	Myc	*Myc* is required for growth-factor mediated induction of *E2F1* [231, 225]. It binds to and remodels the *E2F1* promoter, facilitating *E2F1* transcription [216]. In addition, *Myc* augments protein expression of *E2F1* [232]. Single-cell experiments show that *Myc* is a critical modulator of the amplitude of *E2F* activation [233].
	← TR	E2F1	*E2F1* binds to its own promoter and up regulates transcription (as long as *Cyclin D/E* activity blocks *RB-E2F1* binding) [234].
	⊣ TR	pRB	*RB* binds to *E2F/DP1* complexes and switches their DNA binding activity from activation to repression [221, 235].
	⊣ P	CyclinA	The phosphorylation of the *E2F1*-binding *DP-1* protein by *Cyclin A*, which binds directly to *E2F-1* (as well as *E2F-2,3*) downregulates *E2F1* transcriptional activity in S phase [236, 237, 238].
	⊣ Deg	CAD	This link from Caspase-activated DNase (*CAD*) to *E2F1* ensures that apoptotic cells settle into an *E2F1*-negative attractor regardless of their initial state. The rationale for this is that *E2F1* cannot maintain its activity if DNA is fragmented.
CyclinE	**CyclinE** = (**ATP_H** or **CyclinE**) and **ATP** and **E2F1** and **Pre_RC** and **Cdc6** and not(**p21_H** or **pRB** or **p27Kip1** or **CHK1** or **CHK2** or **Casp3**)		
	PC		In our model, the ON state of *Cyclin E* represents active *Cyclin E/Cdk2* complexes. Thus, its full activation requires high levels of ATP, transcription via *E2F1* not blocked by active *pRB*, binding to *Cdc6* and *pre-RC* complexes, and the absence of its inhibitors *p27^{Kip1}*, *CHK1*, *CHK2*, and *Caspase 3*.
	← Ind	ATP_H	The mechanistic link between normal-to high ATP production by hyperfused mitochondria is unclear, and may indeed be a largely indirect effect via AMPK/p53 activation [120]. That said, there is evidence that low ATP levels turn the balance between *p27^{Kip1}* and *Cdk2/Cyclin E* in *p27^{Kip1}*'s favor as a CDKI, rather than a *Cdk2* substrate [239]. Thus, we assume that the hyperfused network only allows active *Cyclin E* buildup if mitochondrial ATP production is high; an assumpiton supported by observations that forced hyperfusion without increased ATP prodiction did not force *Cyclin E* levels to remain elevated after S-phase completion [120].

Table S1h: Restriction_SW module

← Ind	ATP	As high ATP levels are required for *Cyclin E* buildup, we assume that at least nominal ATP levels remain required to maintain *Cyclin E* activity [120].
← Per	CyclinE	We assume that once *Cyclin E* is activated with the aid of high ATP levels, it can remain active to continue S-phase progression without ongoing high ATP output from mitochondria.
← TR	E2F1	*E2F1* is a potent transcriptional activator of *Cyclin E* [240].
← Compl	Pre_RC	At the G1/S transition, *Cyclin E* is loaded onto chromatin by *pre-RC* complexes (*Cdc6* and *Cdt1* binding), where it is required for *MCM2* loading, origin firing and the start of DNA synthesis [241]. In addition, activation of its partner *Cdk2* by *Cdc6* is contingent on this localization [242].
← Compl	Cdc6	Chromatin association and full activation of *Cyclin E/Cdk2* requires *Cdc6* [242].
⊢ IBind	p21_H	Above-basal *p21*$^{\mathrm{CIP1}}$ is a potent inhibitor of *Cyclin E/Cdk2* activity [243, 186].
⊢ TR	pRB	*Cyclin E* transcription by *E2F1* requires the absence of active, un-phosphorylated *RB* [238].
⊢ IBind	p27Kip1	*p27*^{Kip1} binds to and prevents the activation of *Cyclin E/Cdk2* complexes [190].
⊢ P	CHK1	*Chk1* activation during normal S-phase progression keeps *Cdk2* activity in a physiological range by binding to both *Cdk2* and *Cdc25A*, aiding the loss of *Cyclin E/Cdk2* activity [244].
⊢ Ind	CHK2	*CHK2* phosphorylates *Cdc25A* on serine 123, facilitating its destruction [245] and subsequently blocking *Cyclin E/Cdk2* activity [245].
⊢ Lysis	Casp3	*Caspase 3* cleaves and deactivates *Cyclin E*, which is then rapidly degraded [246].

Table S1i: Origin_Licensing module

Target Node	Node Gate<br>Node Type<br>Link Type	 Node Description<br>Input Node	 <br>Link Description
ORC	**ORC** = **E2F1** or (**Pre_RC** and **Cdt1** and **Cdc6**)		
	PC	*ORC* proteins can bind at origins of replication when transcribed by *E2F1* or as part of a fully assembled and licensed *Pre-RC* complex (including active *Cdc6* and *Cdt1*).	
	← TR	E2F1	Expression of the *ORC1* gene is regulated by *E2F1* [247].

Table S1i: Origin_Licensing module

	← Compl	Pre_RC	Licensed but not yet fired replication complexes (*Pre-RC*s containing *ORC*, *Cdc6*, *Cdt1* and inactive *MCM*s) remain stable at sites of replication origin until fired by the activation of the *MCM* helicase [248].
	← Compl	Cdc6	Availability of stable (unphosphorylated) *Cdc6* in the *Pre-RC* is necessary for the maintenance of licensed origins [248].
	← Compl	Cdt1	Active (unphosphorylated and not geminin-bound) *Cdt1* bound to the *Pre-RC* is necessary for the maintenance of licensed origins [248].
Cdc6	**Cdc6** = not(**f4N_DNA** and **CyclinA**) and (((**E2F1** and **ORC**) and not **Plk1** and not **CyclinA**) or (**Pre_RC** and **ORC** and **Cdc6** and **Cdt1**)) and not **Casp3**		
	Prot		In our model the *Cdc6* node represents nuclear, chromatin-bound *Cdc6*. Thus, the node is only active during the assembly of pre-replication complexes, or their ongoing presence during DNA replication. *Cdc6* is ON in the absence of *Caspase 3* or *CyclinA / Cdk2* phosphorylation of *Cdc6* in all origins required for the completion of DNA replication (thus, its inhibition by *Cyclin A* also requires 4N DNA). In addition, active *Cdc6* requires either transcription by *E2F1* and recruitment by origin-bound *ORC* proteins in the absence of mitotic *Plk1* or maintenance of *Pre-RCs* by the presence of all of its components.
	⊢ Ind	f4N_DNA	In our model, full deactivation of Cdc6 represents the firing of all ORCs as DNA replication is completed. Thus *Cyclin A*'s inhibitory action takes full effect once the cell reaches 4N DNA content [249].
	⊢ P	CyclinA	Phosphorylation of *CDC6* by *Cyclin A/Cdk2* during DNA replication leads to its re-localization to the cytoplasm [249].
	← TR	E2F1	Transcription of Cdc6 is directly induced by E2F1 [250].
	← Compl	ORC	*ORC* recruits *Cdc6* to origins of replication [248].
	⊢ P	Plk1	*Plk1* binds, phosphorylated and strongly recruits Cdc6 to the spindle pole during metaphase, then to the central spindle in anaphase, leading to its exclusion from chromosomes until telophase, when the majority of *Plk1* is degraded by APC/C^{Cdh1} [251].
	← Compl	Pre_RC	Licensed but not yet fired replication complexes (*Pre-RC*s containing *ORC*, *Cdc6*, *Cdt1* and inactive *MCM*s) remain stable and *Cdc6*-bound until fired by the activation of the *MCM* helicase [248].
	← Per	Cdc6	Stable (unphosphorylated) *Cdc6* in the *Pre-RC* is necessary for the maintenance of licensed origins [248].
	← Compl	Cdt1	Active (unphosphorylated and not geminin-bound) *Cdt1* bound to the *Pre-RC* is necessary for the maintenance of licensed origins [248].

Table S1i: Origin_Licensing module

	$\vdash$ Lysis	Casp3	*Caspase 3* cleaves and deactivates *Cdc6* [252].
Cdt1	**Cdt1** = not **geminin** and **ORC** and **Cdc6** and not(**CyclinE** and **CyclinA** and **Cdc25A**) and ((**Pre_RC** and (**E2F1** or **Myc**)) or (**E2F1** and (**Myc** or not **pRB**)))		
	Prot		Replication-origin bound *Cdt1* requires the absence of *geminin*, the presence of origin-bound *ORC* and *Cdc6*, and the absence of sustained *Cdk2* activity responsible for the firing of all origins during DNA synthesis (modeled as simultaneous *Cyclin E*, *Cyclin A* and *Cdc25A* activity). Bound into a licensed pre-replication complex (*Pre-RC*), *Cdt1* remains stable as long as it is transcribed by *E2F1* [253] or *Myc* [254] (this guarantees that *Pre-RC* complexes cannot persist indefinitely in the absence of de novo transcription). Alternatively, it can be turned on by *E2F1*, aided by *Myc* or the absence of *RB*, and *FoxO3* in cells with 4N DNA.
	$\vdash$ IBind	geminin	*Geminin* binds to *Cdt1* at pre-replication complexes, where it blocks *Cdt1* binding to DNA, sequestering it away from *Pre-RC*s [255].
	$\leftarrow$ Compl	ORC	*ORC*-bound origin of replication sites are the point of pre-replication complex assembly, where *Cdt1* is recruited by *ORC*-bound *Cdc6* [248].
	$\leftarrow$ Compl	Cdc6	*ORC*-bound *Cdc6* recruits *Cdt1* to *pre-RC* complexes [248].
	$\vdash$ P	CyclinE	Sustained *Cdk2* activity during S-phase (modeled as simultaneous *Cyclin E*, *Cyclin A* and *Cdc25A* activity) is responsible for the firing of all origins required to complete DNA synthesis; it also leads to the phosphorylation and proteasomal degradation of *Cdt1* [248].
	$\vdash$ P	CyclinA	Sustained *Cdk2* activity leads to phosphorylation and degradation of *Cdt1* [248].
	$\vdash$ P	Cdc25A	Sustained *Cdk2* activity leads to phosphorylation and degradation of *Cdt1* [248].
	$\leftarrow$ Compl	Pre_RC	Licensed but not yet fired replication complexes (*Pre-RC*) remain stable until fired during DNA replication [248].
	$\leftarrow$ TR	Myc	*Cdt1* is a direct transcriptional target of the *Myc-Max* complex [254], ensuring its availability for *Pre-RC* formation and maintenance.
	$\leftarrow$ TR	E2F1	*Cdt1* is a direct transcriptional target of *E2F1* [253], ensuring its availability for *Pre-RC* formation and maintenance.
	$\vdash$ TR	pRB	*E2F1*-mediated transcription of *Cdt1* is blocked by hypophosphorylated (active) *pRB* [253].
Pre_RC	**Pre_RC** = **ORC** and **Cdc6** and **Cdt1** and not(**Replication** and **f4N_DNA**)		
	PC		*Pre-RC* complexes assemble when *ORC*, *Cdc6*, and *Cdt1* are all bound to sites of replication origin along the DNA. The node denoting their licensing turns OFF at the moment of transition from ongoing *Replication* to *f4N_DNA* (it is blocked in the one time-point when both of these nodes are ON).

Table S1i: Origin_Licensing module

	← Compl	ORC	*Pre-RC* complexes assemble when *ORC*, *Cdc6*, and *Cdt1* are all bound to sites of replication origin along the DNA [248].
	← Compl	Cdc6	*Pre-RC* complexes assemble when *ORC*, *Cdc6*, and *Cdt1* are all bound to sites of replication origin along the DNA [248].
	← Compl	Cdt1	*Pre-RC* complexes assemble when *ORC*, *Cdc6*, and *Cdt1* are all bound to sites of replication origin along the DNA, leading to the recruitment of the *MCM* helicase [248].
	⊢ Unbind	Replication	*Pre-RC*s fire and fall apart during DNA replication [248].
	⊢ Ind	f4N_DNA	In our model the *Pre-RC* node turns OFF when *Replication* is completed, marked by the time-point when both *Replication* and *f4N_DNA* are ON.
geminin	**geminin** = **E2F1** and not(**pAPC** and **Cdc20**) and not **Cdh1**		
	Prot	*Geminin* is present when transcribed by *E2F1* and not targeted for degradation by APC/C^{Cdh1} or APC/C^{Cdc20} [256].	
	← TR	E2F1	*Geminin* is a direct transcriptional target of *E2F1* [253].
	⊢ Ubiq	pAPC	*Geminin* is a target of APC/C^{Cdc20} at the metaphase/anaphase transition [256].
	⊢ Ubiq	Cdc20	*Geminin* is a target of APC/C^{Cdc20} at the metaphase/anaphase transition [256].
	⊢ Ubiq	Cdh1	*Geminin* is a target of APC/C^{Cdh1} ubiquitin ligase [257].

Table S1j: Phase_SW module

Target Node	Node Gate<br>Node Type<br>Link Type	Node Description<br>Input Node	Link Description
CyclinA_mRNA	**CyclinA_mRNA** = ((**E2F1** and (not **pRB** or **Myc**)) or **FoxM1**) and not(**p53_4** or **CAD**)		
	mRNA	In non-apoptotic cells (no *CAD*), *Cyclin A* is transcribed by *E2F1* in the absence of active *RB* or by *FoxM1*. In addition, high and/or sustained *p53* activity, represented in our model by the *p53_4* node, can also repress *Cyclin A* transcription [258, 259].	
	← TR	E2F1	*Cyclin A* is transcriptionally activated by *E2F* factors [238].
	⊢ TR	pRB	Active *RB* blocks *E2F1*'s ability to transcribe *Cyclin A* [260].
	← TR	Myc	*Cyclin A* is transcriptionally activated in *Myc* overexpressing cells [261].

Table S1j: Phase_SW module

	← TR	FoxM1	Depletion of *FoxM1* results in reduced *Cyclin A2* expression (it is not clear whether *FoxM1* is a direct transcriptional inducer of *Cyclin A*) [262, 263, 264, 265].
	⊢ TR	p53_4	*p53* overexpression can repress *Cyclin A2*[259], an effect that likely requires high or sustained *p53* levels so that cells able to recover from G2 arrest without excessive damage do not loose their G2 cyclin profile and can continue to mitosis [258].
	⊢ Ind	CAD	This link from Caspase-activated DNase (*CAD*) to *Cyclin A* mRNA ensures that apoptotic cells settle into a G0-like attractor regardless of their initial state. The rationale for this is that no mRNA synthesis can be maintained if DNA is fragmented (we only use these links from *CAD* if needed).
Emi1	**Emi1** = (**E2F1** or not **pRB** or not **p21**) and not(**Plk1** and **CyclinB** and **Cdk1** and (**U_Kinetochores** or **A_Kinetochores**))		
	Prot		Our model allows the sustained presence of *Emi1* protein when it is either actively transcribed by *E2F1* [266, 267] or lacks joint inhibition by *pRB* [267] and *p21* [268]. Degradation of *Emi1* is mediated by *Plk1* and *CyclinB/Cdk1* complexes; initiation of this degradation requires at least temporary co-localization of *Emi1* with *Plk1* at mitotic spindle poles [269].
	← TR	E2F1	*Emi1* is a direct transactional target of *E2F1* [266, 267].
	⊢ TR	pRB	Active retinoblastoma protein can block *Emi1* transcription mediated by *E2F1* [267].
	⊢ Ind	p21	*p21* activation during DNA damage lead to a substantial decrease of *Emi1* levels, not observed in *p21*-null cells [268].
	⊢ P	Plk1	*Plk1* phosphorylates *Emi1* at mitotic spindle poles, stimulating its *βTrCP* binding and ubiquitination [269].
	⊢ Ind	CyclinB	*Cyclin B/Cdk1* enhances the ability of *Plk1* to mediate *Emi1* destruction [269].
	⊢ Ind	Cdk1	*Cyclin B/Cdk1* enhances the ability of *Plk1* to mediate *Emi1* destruction [269].
	⊢ Ind	U _Kinetochores	As *Plk1*-mediated phosphorylation of *Emi1* occurs at mitotic spindle poles, our model requires ongoing mitosis for this interaction [269].
	⊢ Ind	A _Kinetochores	*Plk1* phosphorylates *Emi1* at mitotic spindle poles, stimulating its *βTrCP* binding and ubiquitination [269].
FoxM1	**FoxM1** = (**Myc** and **CyclinE**) or (**CyclinA** and **Cdc25A** and **Cdc25B**) or (**Plk1** and **CyclinB** and **Cdk1**)		
	TF		In our model, *FoxM1* activity requires increased expression by *Myc* [270] and activating phosphorylation by *Cyclin E/Cdk2*. Alternatively, *FoxM1* activity can be sustained by potent *Cdk2* / *Cdk1* activity in G2 (supported by *Cdc25A* or *Cdc25B*), or a serial phosphorylation by *Cyclin B/Cdk1* and *Plk1* during mitosis.

Table S1j: Phase_SW module

	← TR	Myc	*FoxM1* is a direct transcriptional target of *c-Myc* [270].
	← P	CyclinE	*Cyclin E/Cdk2* complexes bind and phosphorylate FoxM1, potently inducing its transcriptional activity, which starts during S-phase [271].
	← P	CyclinA	In addition to *Cyclin E/Cdk2*, *Cyclin A/Cdk2* complexes can also keep *FoxM1* transcriptionally active by phosphorylating its autoinhibitory N-terminal region [272].
	← Compl	Cdc25A	Active *Cdc25A* binds to and enhances the transcriptional activity of *FoxM1*, potentually by bridging FoxM1 and active cyclin-*Cdk2* complexes [273].
	← Ind	Cdc25B	*Cdc25B* overexpression can increase *FoxM1*-dependent transcription, likely via aiding *Cdk1* activity [274].
	← P	Plk1	*Plk1* binds and phosphorylates *FoxM1*, which activates *FoxM1*-mediated transcription in early mitosis [275].
	← P	CyclinB	*FoxM1* binds *Plk1*, and phosphorylation of two key residues at this binding domain by *Cyclin B/Cdk1* primes it for *Plk1* binding [275].
	← P	Cdk1	*FoxM1* binds *Plk1*, and phosphorylation of two key residues at this binding domain by *Cyclin B/Cdk1* primes it for *Plk1* binding [275].
Cdc25A	**Cdc25A** = not **CHK2** and ((**FoxM1** and **E2F1** and not **pRB**) or (not **Cdh1** and (**FoxM1** or (**E2F1** and not **pRB**)))) and (not(**GSK3** or **CHK1**) or **CyclinE** or **CyclinA** or (**CyclinB** and **Cdk1**))		
	Ph		As the precise combinatorial regulation of *Cdc25A* throughput the cell cycle is unknown, our model assumes that accumulation of the *Cdc25A* protein requires transcriptional activation by both *E2F1* in the absence of *pRB*, and *FoxM1* to override destruction by APC/C^{Cdh1}. Alternatively, one of the two transcription factors can drive *Cdc25A* accumulation in the absence of APC/C^{Cdh1}. In addition, stabilization of *Cdc25A* either requires the absence of *GSK3β* and *CHK1* (both of which promote its degradation), or stabilization by *Cdk* activity.
	← TR	FoxM1	*FoxM1* is a direct transcriptional inducer of *Cdc25A* [273].
	← TR	E2F1	*E2F1* is a direct transcriptional inducer of *Cdc25A* [276].
	⊣ TR	pRB	Active (hypo-phosphorylated) *pRB* blocks *E2F1*'s ability to drive *Cdc25A* transcription [276, 277].
	⊣ Deg	Cdh1	The APC/C^{Cdh1} complex degrades *Cdc25A* at mitotic exit [278, 279].
	⊣ P	GSK3	*GSK3β* phosphorylates *Cdc25A*, promoting its proteolysis [280].

Table S1j: Phase_SW module

	⊣ P	CHK1	*CHK1* phosphorylates *Cdc25A*, promoting its proteolysis and inhibiting its interaction with *Cyclin A/Cdk1/2* and *Cyclin B/Cdk1* [281, 282].
	⊣ P	CHK2	*CHK2* phosphorylates *Cdc25A*, promoting its proteolysis and blocking its ability to keep *Cyclin A/Cdk1/2* active [245].
	← P	CyclinE	*Cdc25A* protein levels are stabilized during S-phase by *CyclinE/Cdk2*-dependent phosphorylation [283].
	← P	CyclinA	*Cdc25A* protein levels are stabilized during S and G2 by *Cdk2*-dependent phosphorylation. *Cdk2* first partners with *Cyclin E* [283], then continues to stabilize *Cdc25A* past the point of *Cyclin E* expression by partnering with *Cyclin A* [284].
	← P	CyclinB	During mitosis, *Cdc25A* is stabilizes by *Cyclin B/Cdk1* phosphorylation, which protects it from the proteasome [285].
	← P	Cdk1	During mitosis, *Cdc25A* is stabilizes by *Cyclin B/Cdk1* phosphorylation, which protects it from the proteasome [285].
CyclinA	**CyclinA** = **CyclinA_mRNA** and not **pAPC** and ((**Cdc25A** and (not **Cdh1** or **Emi1**)) or (**CyclinA** and ((not **Cdh1** and (**Emi1** or not **UbcH10**)) or (**Emi1** and not **UbcH10**))))		
	PC		*Cyclin A* activity requires transcription (*Cyclin A* mRNA) and the absence of degradation by phosphorylated (mitotic) *pAPC*. In addition, turning ON inactive *Cyclin A* requires activation of *Cdk2* by *Cdc25A* [286] and the absence / *Emi1*-mediated inhibition of APC/C^{Cdh1}. Once active, *Cyclin A* maintains its activity in the absence of overpowering influences driving its degradation. Namely, *Cyclin A* relies on either *Emi1* or the absence of *UbcH10* for its ability to keep inactive APC/C^{Cdh1} in check. To overpower active APC/C^{Cdh1}, *Cyclin A* requires both *Emi1* and no *UbcH10*. The precise combinatorial regulation of *Cyclin A* is not known; the above logic is consistent with *Cyclin A* activity pattern during cell cycle progression.
	← TL	CyclinA _mRNA	Sustained availability of *Cyclin A* requires ongoing translation from *CyclinA* mRNA.
	⊣ Deg	pAPC	*Cyclin A* is degraded by the APC/C^{Cdc20} in prometaphase (as soon as the APC/C components are phosphorylated by Cdk1) [287, 288], before the full activation of the complex at SAC passage [289]. In our model, this stage of mitotic APC/C^{Cdc20} activation is represented by *Cdk1*-phosphorylated *APC/C* (*pAPC*).
	← DP	Cdc25A	*Cdc25A* promotes active *Cyclin A/Cdk2* complex formation by removing inhibitory phosphorylation of *Cdk2* [286, 290].
	⊣ Deg	Cdh1	*Cyclin A* is degraded by APC/C^{Cdh1} in the presence of the *UbcH10* protein [291, 292, 199].
	← Compl	Emi1	*Emi1* binding to *Cdh1* is required to stabilize *Cyclin A* levels at the G1/S transition, allowing *Cyclin A/Cdk2* to block APC/C^{Cdh1} [293, 294, 295].
	← Per	CyclinA	We assume that once activated, *Cyclin A/Cdk2,1* complexes can sustain their activity until *Cyclin A* is degraded.

Table S1j: Phase_SW module

	⊢ Deg	UbcH10	*Cyclin A* degradation by APC/C^{Cdh1} requires *UbcH10* [292].
Wee1	**Wee1** = (**Replication** or **CHK1**) and not (**Cdk1** and **CyclinB**) and (**CHK1** or not (**Cdk1** and **CyclinA** and **Plk1**)) and not **Casp3**		
	K	*Wee1* is active during *Replication*, unless its activity is blocked by *CyclinA/Cdk1* OR *CyclinB/Cdk1* [296].	
	← ComplProc	Replication	To model the sensitivity of *Wee1* activation to ongoing DNA synthesis even in the absence of damage, our model turns on *Wee1* immediately upon the start of DNA replication and maintains it until both Replication and the checkpoint kinase *Chk1* is OFF [297]. In addition, *Wee1* activity has been implicated in maintaining normal replication fork procession, linking its activity directly to ongoing replication [298].
	← P	CHK1	During DNA replication *Wee1* is activated by the checkpoint kinase *CHK1* [297].
	⊢ P	CyclinB	*Cyclin B/Cdk1* is a strong inducer of *Wee1* phosphorylation and deactivation [296].
	⊢ P	Cdk1	The somatic *Wee1* protein is an order of magnitude more sensitive to *Cdk1* activity than *Cdc25C*. Thus, both *Cyclin A/Cdk1* and *Cyclin B/Cdk1* strongly induce *Wee1* phosphorylation and deactivation [296].
	⊢ P	CyclinA	*Cyclin A/Cdk1* is a strong inducer of *Wee1* phosphorylation and deactivation [296].
	⊢ P	Plk1	*Plk1* phosphorylation at S53 promotes *Wee1* degradation [299]. This event is primed by *Cdk1* phosphorylation of *Wee1* at S123 [299]. As the main partner of *Cdk1* in mitosis is *Cyclin B*, we assume that assistance from *Plk1* to block *Wee1* is more relevant when paired with *Cyclin A/Cdk1* complexes.
	⊢ Lysis	Casp3	*Caspase 3* cleaves and deactivates *Wee1* [300].
UbcH10	**UbcH10** = not **Cdh1** or (**UbcH10** and (**Cdc20** or **CyclinA** or **CyclinB**))		
	UbL	The ubiquitin-conjugating enzyme (E2) *UbcH10* is active in the absence of *Cdh1*. Alternatively, active *UbcH10* is maintained in the presence of *Cdh1* when some of its targets are present: *Cdc20* OR *CyclinA* OR *CyclinB* [292].	
	⊢ Deg	Cdh1	*UbcH10* is degraded by APC/C^{Cdh1}.
	← Per	UbcH10	Active *UbcH10* cannot be autoubiquitinated in the presence of APC/C^{Cdh1} substrates and thus remains active [292].
	← PBind	Cdc20	The presence of APC/C^{Cdh1} substrates, including *Cdc20*, inhibit the autoubiquitination of *UbcH10* but not its function, thus preserving APC activity [292].
	← PBind	CyclinA	The presence of APC/C^{Cdh1} substrates, including *Cyclin A*, inhibit the autoubiquitination of *UbcH10* but not its function, thus preserving APC activity [292].

Table S1j: Phase_SW module

	$\leftarrow$ PBind	CyclinB	The presence of APC/C^{Cdh1} substrates, including *CyclinB*, inhibit the autoubiquitination of *UbcH10* but not its function, thus preserving APC activity [292].
CyclinB	**CyclinB** = (**FoxM1** or (**FoxO3** and **CyclinB**)) and not(**Cdh1** or (**pAPC** and **Cdc20**)) and not **p53_4**		
	PC		*Cyclin B* node is ON when the concentration of *Cyclin B* proteins is high (does not represent the activity of *CyclinB/Cdk1* complexes). This occurs when *Cyclin B* is transcribed by *FoxM1*, maintained by *FoxO3* transcription, and not undergoing *APC*-mediated degradation by APC/C^{Cdc20} or APC/C^{Cdh1}. In addition, high and/or sustained *p53* activity, represented in our model by the *p53_4* node, can also repress *Cyclin B* transcription [258, 301].
	$\leftarrow$ TR	FoxO3	*FoxO3* is a direct transcriptional regulator of *Cyclin B*; its activation in G2 helps increase/maintain *Cyclin B* levels [302].
	$\leftarrow$ TR	FoxM1	*FoxM1* is a direct transcriptional regulator of *Cyclin B1* [265, 303].
	$\leftarrow$ Per	CyclinB	Here we assume that FoxO3 alone can only maintain, but not independently induce *Cyclin B1* expression.
	$\dashv$ Deg	pAPC	*Cyclin B* is degraded by APC/C^{Cdc20} [291].
	$\dashv$ Deg	Cdc20	*Cyclin B* is degraded by APC/C^{Cdc20} [291].
	$\dashv$ Deg	Cdh1	*Cyclin B* is degraded by APC/C^{Cdh1} [291].
	$\dashv$ TR	p53_4	*p53* can repress *Cyclin B* transcription [301], an effect that likely requires high or sustained *p53* levels so that cells able to recover from G2 arrest without excessive damage do not loose their G2 cyclin profile and can continue to mitosis [258].
Cdc25B	**Cdc25B** = **FoxM1** and **f4N_DNA**		
	Ph		*Cdc25B* activation requires transcription by *FoxM1*, centrosomal localization, and activation by *Aurora A* kinase on replicated centrosomes.
	$\leftarrow$ TR	FoxM1	*FoxM1* is an essential inducer or *Cdc25B* [304].
	$\leftarrow$ Loc	f4N_DNA	*Cdc25B* is localized at centrosomes, where it is activated by *Aurora A* kinase [305]. As *Aurora A* itself is only recruited to duplicated, centrosomes before their separation [306], *Cdc25B* activation requires duplicated centrosomes. As our model does not directly account for centrosome dynamics, we account for this by requiring the completion of S-phase (4N DNA).
Plk1	**Plk1** = not **Cdh1** and (**FoxM1** or **Plk1_H**) and ((**CyclinB** and **Cdk1**) or (**CyclinA** and **Cdc25A** and not **Wee1**))		

Table S1j: Phase_SW module

	K		*Plk1* activity requires the absence of APC/C^{Cdh1}, transcription by *FoxM1*, or high *Plk1* levels transcribed earlier by both *FoxM1* and *FoxO3* (see *Plk1_H* below) [302]. In addition, *Plk1* activation requires phosphorylation by either *CyclinB/Cdk1* during mitosis or *Cyclin A/Cdk2* (aided by lack of *Wee1* and *Cdc25A*) at the G2/M boundary [307].
	$\vdash$ Ubiq	Cdh1	The majority of *Plk1* is degraded in anaphase by the APC/C^{Cdh1} complex [308].
	$\leftarrow$ TR	FoxM1	*Plk1* is a direct transcriptional target of *FoxM1* [275].
	$\leftarrow$ Per	Plk1_H	Our model tracks the accumulauton of high-enough levels of *Plk1* to survive APC/C^{Cdh1} mediated destruction into telophase via the *Plk1_H* node. Its ON state represents strong prior *Plk1* activation. Thus, it sustains the *Plk1* node in the absence of *FoxM1*-mediated transcription until *Plk1_H* itself is lost as *Plk1* levels fall.
	$\leftarrow$ P	CyclinB	*Plk1* is activated by *Cyclin B/Cdk1* phosphorylation [309, 310, 311].
	$\leftarrow$ P	Cdk1	*Plk1* is activated by *Cyclin B/Cdk1* phosphorylation [309, 310, 311].
	$\leftarrow$ P	CyclinA	*Plk1* activation at the G2/M boundary, before *Cdk1/Cuclin B* complexes are activated, requires active *Cyclin A/Cdk* [307].
	$\leftarrow$ Ind	Cdc25A	As we do not include a separate *Cdk2* node in our model, strong *Cyclin A/Cdk2* activity requires ongoing dephosphorylation of *Cdk2* by *Cdc25A* [312].
	$\vdash$ Ind	Wee1	*Cyclin A*-mediated induction of *Plk1* is blocked by *Wee1* kinase, which specifically inhibits *Cdk2* activity [307].
Cdc25C	**Cdc25C** = **f4N_DNA** and **Plk1** and ((**Cdc25B** and not **CHK1** and (not **CHK2** or **Bipolar_Spindle**)) or (**CyclinB** and **Cdk1**))		
	Ph		In our model, *Cdc25C* is active in cells with replicated DNA (see *f4N_DNA* $\rightarrow$ *Cdc25C* link). Its activation is initiated by a small, initially cytoplasmic pool of *Cyclin B/Cdk1* activated by *Cdc25B* (not directly represented in our model) and further increased by *Cdc25B* itself, which translocates to the nucleus with the aid of *Plk1*. During mitosis, *Plk1* potentiates the ability of *Cyclin B/Cdk1* to maintain *Cdc25C* activity. In contrast, *CHK1/2* phosphorylate *Cdc25C*, leading to its nuclear exclusion and degradation [313, 314, 315, 316].
	$\leftarrow$ Ind	f4N_DNA	The nature and localization of the signals responsible for the onset and maintenance of *Cdc25C* activity require replicated DNA (*f4N_DNA*) [317, 318]. Namely, *Cdc25C* is initially activated by a small pool of *Cyclin B/Cdk1* (below the ON-threshold of *Cdk1* in our model) which starts our at the replicated centrosome. Moreover, the pool of mitotic *Cdc25C* co-localized with active *Chk1/Cyclin B* is found on condensed chromosomes, again requiring the presence of *f4N_DNA* [319].

Table S1j: Phase_SW module

	$\leftarrow$ P	Plk1	In addition, *Plk1* induces nuclear transport of *CDC25B*, where it contributes to the initiation of *Cdk1* activity [317]. During mitosis, *Plk1* helps maintain strong *Cdc25C* activation by phosphorylating it on the same site as *Cyclin B/Cdk1* [320], as indicated by the profound decrease of Cdc25C activity in Plk1-inhibited mitotic cells [310, 321].
	$\leftarrow$ Ind	Cdc25B	*CDC25B* starts the cascade leading to mitotic entry by activating a small centrosomal pool of *Cyclin B/Cdk1*, leading to their nuclear translocation where they trigger the activation of *Cdc25C* and eventually the larger nuclear *Cyclin B/Cdk1* pool [322, 323, 318].
	$\dashv$ P	CHK1	*CHK1* phosphorylates *Cdc25C*, leading to its nuclear exclusion, loss of access to its main target, *Cdk1* [319]. In addition, *CHK1* blocks the ability of *Cdc25B* to activate *Cdc25C* at the centrosomes by phosphorylating it and blocking its *Cdk1* activity [315, 316].
	$\dashv$ P	CHK2	*CHK2* phosphorylates *Cdc25C*, leading to its nuclear exclusion and degradation [313, 314].
	$\leftarrow$ Ind	Bipolar _Spindle	During mitosis, *CHK2* is localized to the centrosomes where it helps maintain SAC (not modeled). Thus, we assume that it cannot repress *Cdc25C* once a bipolar spindle has been assembled [324].
	$\leftarrow$ P	CyclinB	*Cyclin B/Cdk1* complexes are potent activators of *Cdc25C*, creating positive feedback that causes switch-like mitotic entry [325, 326].
	$\leftarrow$ P	Cdk1	*Cyclin B/Cdk1* complexes are potent activators of *Cdc25C*, creating positive feedback that causes switch-like mitotic entry [325, 326].
Cdk1	**Cdk1** = (**CyclinB** and **Cdc25C**) and (not(**CHK1** or **CHK2**) or (not **Wee1** and **Cdk1**))		
	K		Full *Cdk1* kinase activation requires its binding partner *Cyclin B* and the *Cdc25C* phosphatase, which maintains *Cdk1* in an active dephosphorylated state. *Cdk1* is inhibited by the checkpoint kinases *CHK1/2*, unless already full active and *Wee1* kinase is inhibited.
	$\leftarrow$ Compl	CyclinB	Full kinase activation of *Cdk1* in our model requires it to complex with *Cyclin B* [327].
	$\leftarrow$ DP	Cdc25C	*Cdk1* is subject to inhibitory phosphorylation by *Wee1* or *Myt1*, and its dephosphorylation is carried out by activated *Cdc25C* [325, 327, 326].
	$\dashv$ P	CHK1	In the absence of *CHK1* kinase, a small cytosolic (centrosomal) pool of *Cyclin B/Cdk1* can be activated by *Cdc25B*, the nuclear translocation of which can trigger a positive feedback loop that activates the full *Cdk1* pool (assuming nuclear *Wee1* is also inactive). Thus, *CHK1* can maintain the OFF state of inactive *Cdk1* [307].

Table S1j: Phase_SW module

	⊣ P	CHK2	The absence of *CHK2* activity is required to keep *Cyclin B/Cdk1* activate via *Cdc25B/C*-mediated dephosphorylation [328].
	⊣ P	Wee1	*Wee1* is a nuclear protein that ensures the completion of DNA replication prior to mitosis by blocking nuclear *Cdk1* activation [329].
	← Per	Cdk1	We assume that the presence of fully activated, nuclear *Cdk1* is able to overcome the effect of active *Wee1*, given that *Wee1* is very sensitive to *Cdk1*-mediated inhibitory phosphorylation [296].
pAPC	**pAPC** = (**CyclinB** and **Cdk1** and **Plk1**) or (**CyclinB** and **Cdk1** and **pAPC**) or (**pAPC** and **Cdc20**)		
	PC		In line with evidence that *Plk1* can aid full activation of *APC/C*, but *Cdk1* appears to be the more potent inducer, our model requires both *Cyclin B/Cdk1* and *Plk1* to activate *APC/C* from an OFF state, but only *Cdk1* activity to maintain it. In addition, ongoing phosphorylation of the functional APC/C^{Cdc20} complex is no longer required.
	← P	CyclinB	*CyclinB/Cdk1* activation triggers mitotic entry and promotes APC/C^{Cdc20} activity via APC/C subunit phosphorylation [330, 331].
	← P	Cdk1	*CyclinB/Cdk1* activation triggers mitotic entry and promotes APC/C^{Cdc20} activity via APC/C subunit phosphorylation [330, 331].
	← P	Plk1	In addition to *Cyclin B/Cdk1* phosphorylation, full activation of the APC/C^{Cdc20} complex also requires the kinase activity of *Plk1* [332].
	← Per	pAPC	Activated APC/C^{Cdc20} initiates the Metaphase / Anaphase transition by degrading *Cyclin B* and securin [256]. Once active, APC/C^{Cdc20} no longer requires sustained *CyclinB/Cdk1* or *Plk1* phosphorylation.
	← Compl	Cdc20	Once active, APC/C^{Cdc20} no longer requires sustained *CyclinB/Cdk1* or *Plk1* phosphorylation.
Cdc20	**Cdc20** = **pAPC** and not **Emi1** and not **Cdh1** and ((**Bipolar_Spindle** and not(**Mad2** and **Mps1**)) or (not **CyclinA** and not(**CyclinB** and **Cdk1**)))		
	Prot		In our model, APC/C^{Cdc20} complex formation is represented by the joint activity of *Cdc20* and phosphorylated *APC/C* (*pAPC*). *Cdc20* is thus ON in the presence of *pAPC* when both *Emi1* and *Cdh1* are absent (APC/C^{Cdh1} is represented by the *Cdh1* node, see below). In addition, *Cdc20* activity requires a bipolar spindle, and either the absence of *Mps1* and *Mad2* at unattached kinetochores, or the absence of *Cdc20* phosphorylation by *Cyclin B/Cdk1* or by *Cyclin A/Cdk2* complexes to potentiate the interaction between *Mad2* and *Cdc20*, and *pAPC* (present and phosphorylated) [333].
	← Compl	pAPC	*Cdc20* becomes active in early mitosis by binding to *APC/C*, an event that requires *Cyclin B/Cdk1*-mediated phosphorylation of several core *APC/C* subunits [279, 334].

Table S1j: Phase_SW module

	$\dashv$ IBind	Emi1	*Emi1* binds *Cdc20* and inhibits the ubiquitin ligase activity of APC/C^{Cdc20} [294].
	$\dashv$ Deg	Cdh1	APC/C^{Cdh1} complexes degrade *Cdc20*, leading to a complete switch from APC/C^{Cdc20} to APC/C^{Cdh1} during mitotic exit [279, 335].
	$\leftarrow$ Ind	Bipolar _Spindle	Completion of a bipolar spindle in which every chromosome is pulled in opposite directions by microtubule-attached kinetochodes is required for SAC passage and APC/C^{Cdc20} activation [336].
	$\dashv$ IBind	Mad2	Eukaryotic cells do not separate their replicated genome until they pass the Spindle Assembly Checkpoint (SAC). Namely, all their chromosomes need to be are aligned with respect to the metaphase plane and the two copies of each chromosome need to be attached to opposite poles of the mitotic spindle [331]. This physical alignment is monitored via *Mad2*: kinetochores that remain unattached to microtubules catalyze the sequestration of *Cdc20* and thus inhibit APC/C^{Cdc20} [337, 336].
	$\dashv$ IBind	Mps1	*Mps1* inhibits cytosolic *Cdc20*, as it aids *Cdc20* binding to *Mad2* and *BubR1* before kinetochores mature. Then it recruits factors that sustain the SAC to unattached kinetochores (*Mps1* inhibition in prometaphase removes all known SAC mediators from kinetochores) [338].
	$\dashv$ P	CyclinA	*Cyclin A/Cdk2* complexes phosphorylate *Cdc20* and inactivate the APC/C^{Cdc20} complex during S and G2 [339].
	$\dashv$ P	CyclinB	*Cyclin B* partners with *Cdk1* to keep *Cdc20* phosphorylated, increasing its interaction with *Mad2* rather than *APC/C* [340].
	$\dashv$ P	Cdk1	*Cdk1*-phosphorylated *Cdc20* interacts with *Mad2* rather than *APC/C*, resulting in a block on APC/C^{Cdc20} activation until completion of spindle assembly [333].
Cdh1	**Cdh1** = not(**CyclinB** and **Cdk1**) and not(**CyclinA** and (**Emi1** or **Cdc25A**))		
	PC		APC/C^{Cdh1} activity requires the absence of Cyclin Dependent kinase phosphorylation by *Cyclin B/Cdk1*, or *Cyclin A/Cdk2* aided by further inhibition of *Cdh1* by *Emi1*, or ongoing *Cdk2* activation by *Cdc25A* in the absence of *Emi1*.
	$\dashv$ P	CyclinB	*Cyclin B/Cdk1* phosphorylates *Cdh1* during mitosis, impairing its interaction with *APC/C* [279, 291].
	$\dashv$ P	Cdk1	*Cyclin B/Cdk1* phosphorylates *Cdh1* during mitosis, impairing its interaction with *APC/C* [279, 291].
	$\dashv$ P	CyclinA	Active *Cyclin A/Cdk1,2* complexes phosphorylate *Cdh1* during S, G2 and early mitosis, impairing its interaction with *APC/C* until late stages of mitosis when *Cdk1/2* activity falls [279, 291].

Table S1j: Phase_SW module

⊣ IBind	Emi1	*Emi1* blocks APC/C^{Cdh1} binding to its substrates [295], as well as its ability to add ubiquitin chains to them [341].
⊣ Ind	Cdc25A	As we do not include a separate *Cdk2* node in our model, strong *Cyclin A/Cdk2* activity capable of overriding *Cdh1* activity even in the presence of *Emi1* requires ongoing dephosphorylation of *Cdk2* by *Cdc25A* [325].

Table S1k: Cell_Cycle_Process module

Target Node	Node Gate<br>Node Type<br>Link Type	Node Description<br>Input Node	Link Description
Replication	**Replication** = **Pre_RC** and ((**E2F1** and **CyclinE** and **Cdc25A**) or (**Replication** and **CyclinA** and **Cdc25A** and (**E2F1** or not **f4N_DNA**))) and not **CAD**		
	Proc	The *Replication* node represents ongoing DNA synthesis. This requires a non-apoptotic cell, licensed pre-replication complexes (*Pre_RC*). The start of DNA synthesis requires *E2F1*-meditated transcription of the genes that help execute it, as well as the firing of the first round of replication origins by *Cyclin E/Cdk2*. Once ongoing, replication is sustained by *Cyclin A/Cdk2* and *Cdc25A*, aided by *E2F1* and terminated by completion of a full round of synthesis (*4N_DNA*).	
	← ComplProc	Pre_RC	Ongoing DNA replication requires licensed replication origins, which fire throughout DNA synthesis [342].
	← ComplProc	E2F1	In addition to *E2F1* target genes directly included in our model, *E2F1* transcribes an array of critical S-phase genes responsible for carrying out DNA synthesis (e.g, *POLA1*, *POLA2*, *MCM3*, *MCM5*, *MCM6*, *PCNA*, *TOP2A*, *RFC2*, *TK1*) [343, 344].
	← ComplProc	CyclinE	DNA replication is initiated by fully active *Cyclin E/Cdk2* [345].
	← ComplProc	Cdc25A	Active *Cdc25A* is required for onset as well as progression through S-phase [346, 347].
	← Per	Replication	Once ongoing, DNA synthesis continues in the presence of active *Cyclin A/Cdk2*, only ending when DNA content is doubled.
	← ComplProc	CyclinA	*Cyclin A/Cdk1* complexes regulate the origin firing program in mammalian cells and are required for the completion of DNA replication [347, 290].
	⊣ ComplProc	f4N_DNA	Complete duplication of a cell's DNA, represented in our model by *f4N_DNA* = ON, marks the end of active *Replication*.
	⊣ ComplProc	CAD	Caspase-activated DNase (*CAD*) destroys DNA, preventing ongoing replication.

Table S1k: Cell_Cycle_Process module

ATR — **ATR = Replication** or **cROS_H**

K — *ATR* accumulates at replication forks during unperturbed DNA synthesis [348], as well as at sites of single-strand DNA damage induced by oxidative stress [349, 350].

← Loc	Replication	*ATR* accumulates at replication forks during unperturbed DNA synthesis [348].
← Ind	cROS_H	Elevated cytosolic ROS activates the *ATR/CHK1* pathway (likely by sustaining DNA damage) [350].

CHK1 — **CHK1 = ATR** and **Glucose**

K — *ATR* kinase activates *CHK1* at replication forks, which not only blocks premature mitosis but also regulates the rate of origin firing by keeping *Cdc25* protein levels from increasing above their physiological range [348].

← P	ATR	*ATR* kinase activates *CHK1* at replication forks (by phosphorylation of serines 317 and 345), which not only blocks premature mitosis but also regulates the rate of origin firing by keeping *Cdc25* protein levels from increasing above their physiological range [351, 348].
← Ind	Glucose	Glucose deprivation induces ubiquitin-mediated *CHK1* degradation [352].

Nuclear_Membrane — **Nuclear_Membrane** = not(**CyclinB** and **Cdk1**) and not(**U_Kinetochores** or **A_Kinetochores**)

MSt — The nuclear membrane node represents a non-mitotic state where the cell's DNA is enclosed by the nuclear membrabe. Thus, this node is ON in the absence of *CyclinB/Cdk1*-mediated phosphorylations of the N-terminal head region of nuclear lamins, which form the meshwork structure near the inner nuclear membrane. This phosphorylations depolymerizes the lamin filament network and disolves the envelope [353]. Once disolved, the nuclear membrane does not form until the mitotic spindle is pulled apart in anaphase, and the chromosomes are concentrated near the two spindle poles. In our model this is represented by the deactivation of both unattached and attached kinetochore nodes.

⊢ P	CyclinB	*CyclinB/Cdk1* phosphorylate the N-terminal head region of nuclear lamins, causing their depolymerization and nuclear envelope breakdown [353].
⊢ P	Cdk1	*CyclinB/Cdk1* phosphorylate the N-terminal head region of nuclear lamins, causing their depolymerization and nuclear envelope breakdown [353].
⊢ ComplProc	U_Kinetochores	Nuclear envelope reformation requires the removal of the Chromosome Passanger Complex (CPC) and Aurora B kinase from anaphase chromosomes. This occurs in anaphase, when the CPC relocalizes to the midline of the cell to aid cleavage furrow assembly [354]. In our model, *U_Kinetochores* = ON signals metaphase before the SAC is cleared, at which point the CPC is attached to centromeres and kinetochores.

Table S1k: Cell_Cycle_Process module

	⊢ ComplProc	A _Kinetochores	Nuclear envelope reformation requires the removal of the Chromosome Passanger Complex (CPC) and Aurora B kinase from anaphase chromosomes. This occurs in anaphase, when the CPC relocalizes to the midline of the cell to aid cleavage furrow assembly [354]. In our model, *A_Kinetochores* = ON signals the end of metaphase and SAC clearance, at which point the CPC remains attached to centromeres and kinetochores.
f4N_DNA	**f4N_DNA** = ((**Replication** and ((**Pre_RC** and **CyclinA**) or **f4N_DNA**)) or (**f4N_DNA** and not **Cytokinesis**)) and not **CAD**		
	MSt	4N DNA content in our model is reached via the completion of *Replication* (via the firing of the last round of replication origins by *Cyclin A/Cdk* complexes) and maintained in non-apoptotic cells the absence of a contractile ring (marked by *Ect2*), driving cytokinesis.	
	← ComplProc	Replication	DNA content is doubled by the process of *Replication*.
	← ComplProc	Pre_RC	*Replication* can only complete DNA synthesis and produce double DNA content if the availability of licensed replication origins is not blocked [342].
	← ComplProc	CyclinA	*Cyclin A/Cdk1* complexes regulate the origin firing program in mammalian cells and are required for the completion of DNA replication [345, 290].
	⊢ Per	f4N_DNA	Once achieved, a cell's 4N DNA content is sustained up to the point of cytokinesis.
	⊢ ComplProc	Cytokinesis	Cytokinesis resets the daughter cell DNA content to 2N.
	⊢ Deg	CAD	Caspase-activated DNase (*CAD*) destroys DNA, preventing maintenance of a double DNA content.
U _Kinetochores	**U_Kinetochores** = **f4N_DNA** and not **Nuclear_Membrane** and not **Cdh1** and not **A_Kinetochores** and ((**CyclinB** and **Cdk1**) or **U_Kinetochores**)		
	MSt	The *U_Kinetochores* node in our model is on from the moment the nuclear envelope is dissolved in prometaphase and the mitotic spindle starts to form, until all kinetochores are properly attached. In addition to the presence of unattached kinetochores, *U_Kinetochores* = ON requires attached sister chromatics, the absence of APC/C^{Cdh1} activity. It is turned on my *Cyclin B/Cdk1* and remains on until the spindle is complete (or it is destroyed by APC/C^{Cdh1}).	
	← ComplProc	f4N_DNA	Metaphase requires replicated sister chromatids (*f4N_DNA*), held together by their kinetochores, face in opposing directions and can be attached to opposite poles of the mitotic spindle.
	⊢ Loc	Nuclear _Membrane	Dissolution of the nuclear membrane in prometaphase is required for unattached kinetochores to be accessible to microtublues and thus aid spindle formation [355].

Table S1k: Cell_Cycle_Process module

	⊢ ComplProc	Cdh1	Premature activation of APC/C^{Cdh1} destroys the incomplete spindle by triggering premature, aberrant anaphase. This occurs due to premature degradation of *APC/C* targets including *Securin* (responsible for keeping sister chromatids attached [356]), *Cyclin B*, *Cdc20*, and Aurora kinase A (*AURKA*) [357].
	⊢ ComplProc	A _Kinetochores	In our model, the transition from unattached to all attached kinetochores ($U\_Kinetochores \rightarrow A\_Kinetochores$) marks the completion of the mitotic spindle and Spindle Assembly Checkpoint (SAC) passage.
	← Per	U _Kinetochores	Once metaphase starts, the mitotic spindle remains incomplete as long as some of the kinetochores remain unattached.
	← ComplProc	CyclinB	The start of mitotic spindle assembly is initiated by active *Cyclin B/Cdk1* [358].
	← ComplProc	Cdk1	The start of mitotic spindle assembly is initiated by active *Cyclin B/Cdk1* [358].
Bipolar _Spindle	**Bipolar_Spindle** = (**CyclinB** and **Cdk1** and **Unfused**) or (**Bipolar_Spindle** and **f4N_DNA** and not **Cytokinesis** and (**Ect2** or **Plk1_H** or ((**U_Kinetochores** or **A_Kinetochores**) and not **Cdh1**)))		
	MSt		The bipolar spindle node in our model represents a mitotic spindle attached to two centrosomes, moved to opposing poles of the dividing cell [355]. Thus it formed in response to *CyclinB/Cdk1* activation, aided by mitochondrial fragmentation. Once formed, it is maintained as long as the cell has 4N DNA (sister chromatids), has not undergone cytokinesis, and it is either still in metaphase (*U_Kinetochores* or *A_Kinetochores* are ON, APC/C^{Cdh1} is OFF), or the cell is assembling a contractile ring (*Ect2* or *Plk1_H* are ON).
	← Ind	CyclinB	*CyclinB/Cdk1* activation orchestrates the reorganization of the cell's microtubule network to drive mitotic spindle formation [359].
	← Ind	Cdk1	*CyclinB/Cdk1* activation orchestrates the reorganization of the cell's microtubule network to drive mitotic spindle formation [359].
	← Ind	Unfused	Loss of *Drp1*-mediated mitochondrial fission induced mitochondrial aggregation around the microtubule organizing center (MTOC), centrosome over-duplication, severely misaligned metaphase chromosomes, and thus disruption of bipolar spindple formation [360, 120].
	⊢ Ind	Cdh1	APC/C^{Cdh1} activation leads to the separation of sister chromatids, and marks the start of anaphase [361].
	← Ind	f4N_DNA	Maintaining a bipolar spindle requires a cell with 4N DNA, either as attached sister chromatids or as separated chromosomes in telophase [355].
	← Per	Bipolar _Spindle	Once formed, the mitotic spindle persists until cytokinesis [355].

Table S1k: Cell_Cycle_Process module

	← Ind	U _Kinetochores	Once formed, a bipolar mitotic spindle persists until through metaphase and the SAC [355].
	← Ind	A _Kinetochores	Once formed, a bipolar mitotic spindle persists until through metaphase and the SAC [355].
	← Ind	Plk1_H	As the *Plk1_H* node represents the active *Plk1* pool briefly protected from destruction by APC/C^{Cdh1} [308] and responsible for recruiting *Ect2* to the central spindle, *Plk1_H* = ON marks ongoing anaphase before the cell assembles its contractile ring to trigger cytokinesis. Thus we use it as a logical condition for keeping the bipolar spindle node ON [355].
	← Ind	Ect2	As the *Ect2* node represents contractile ring assembly before cytokinesis, and as such we use it as a logical condition for keeping the bipolar spindle node ON [355].
	⊢ Ind	Cytokinesis	Cytokinesis separates the two poles of the bipolar spindple into two daughter cells, and thus turns the bipolar spindle node OFF [355].
Mps1	**Mps1** = **U_Kinetochores** or (**CyclinB** and **Cdk1**)		
	K	*Mps1* is a protein kinase critical to the spinde assembly checkpoint [362]. Once activated by *CyclinB/Cdk1* phosphorylation, *Mps1* inhibits cytosolic *Cdc20*, as it aids *Cdc20* binding to *Mad2* and *BubR1* before kinetochores mature. Then it recruits factors that sustain the SAC to unattached kinetochores (*Mps1* inhibition in prometaphase removes all known SAC mediators from kinetochores) [338].	
	← P	CyclinB	*CyclinB/Cdk1*-dependent phosphorylation of *Mps1* is required for its mitotic checkpoint activity [362].
	← P	Cdk1	*CyclinB/Cdk1*-dependent phosphorylation of *Mps1* is required for its mitotic checkpoint activity [362].
	← Loc	U _Kinetochores	*Mps1* aids SAC protein assembly at unattached kinetochores [338].
Mad2	**Mad2** = **U_Kinetochores** and **Mps1** and not **A_Kinetochores**		
	Prot	Our model represents the SAC via the *Mad2* kinetochore-binding protein. *Mad2* is recruited to unattached kinetochores by *Mps1* and remains active as long as the cell has at least one unattached kinetochore and it is responsible for keeping *Cdc20* sequestered from *APC/C*. By keeping *APC* at bay until the spindle is complete, *Mad2* is required for the proper timing of anaphase [336].	
	← Compl	U _Kinetochores	The *Mad2* SAC protein is active and potent in the presence of even a single unattached kinetochore [336].
	← Loc	Mps1	Sustained *Mps1* activity is required for maintaining *Mad1/C-Mad2* complexes [363] and other SAC proteins at unattached kinetochores [338].
	⊢ ComplProc	A _Kinetochores	*Mad2* is inhibited by SAC passage, marked by the completion of the spindle and proper attachment of all kinetochore [336].

Table S1k: Cell_Cycle_Process module

A _Kinetochores	**A_Kinetochores** = **f4N_DNA** and **Bipolar_Spindle** and **Plk1** and (**A_Kinetochores** or (**U_Kinetochores** and **CyclinB** and **Cdk1**)) and not **Cdh1** and not(**pAPC** and **Cdc20**)		
	MSt	The completed spindle, represented by the *A_Kinetochores* node, requires replicated and attached sister chromatids (*f4N_DNA*) and the absence of *APC/C* activity. It turns on when the process of spindle assembly (*U_Kinetochores*) is completed by active *Plk1* localized to unattached kinetochores in the presence of ongoing *Cyclin B/Cdk1* activity, and it remains on until anaphase (*APC/C* activation).	
	← ComplProc	f4N_DNA	Completion of the mitotic spindle requires replicated and attached sister chromatids (*f4N_DNA*).
	← ComplProc	Bipolar _Spindle	Bipolar spindle formation is required for proper attachment and tension on all kinetochores [355].
	← ComplProc	Plk1	*Plk1* activity at unattached kinetochores is required for promoting their attachment [364]. In its absence, kinetochores remain unattached and cells eventually undergo mitotic catastrophe and apoptosis [365].
	← Per	A _Kinetochores	Once assembled, separation of the mitotic spindle requires *APC/C* activity to promote the destruction of sister chromatid cohesion [356].
	← ComplProc	U _Kinetochores	The mitotic spindle is assembled gradually, as the number of unattached kinetochores gradually decreased by the formation of microtubule attachments.
	← ComplProc	CyclinB	Ongoing *Cyclin B/Cdk1* at unattached kinetochores is necessary to keep *Plk1* active and allow the completion of mitosis [366].
	← ComplProc	Cdk1	Ongoing *Cyclin B/Cdk1* at unattached kinetochores is necessary to keep *Plk1* active and allow the completion of mitosis [366].
	⊣ Deg	Cdh1	APC/C^{Cdh1} destroys the spindle by triggering anaphase via the degradation of *APC/C* targets, including *Securin* [357].
	⊣ Deg	pAPC	During normal mitosis, the completed spindle is pulled apart in response to APC/C^{Cdc20}-mediated degradation of *Securin*, which normally blocks *Separate* from severing the *Cohesin* rings keeping sister chromatids attached [356].
	⊣ Deg	Cdc20	During normal mitosis, the completed spindle is pulled apart in response to APC/C^{Cdc20}-mediated degradation of *Securin*, which normally blocks *Separate* from severing the *Cohesin* rings keeping sister chromatids attached [356].
Plk1_H	**Plk1_H** = **Plk1** and **FoxM1** and (**Plk1_H** or **FoxO3** or **FoxO1**)		
	K	The ON state of *Plk1_H* encodes the short-lived memory of a sufficiently large active *Plk1* pool to temporarily survive *Plk1* destruction by APC/C^{Cdh1} [308], recruit *Ect2* to the central spindle, and thus aid the completion of cytokinesis [367]. Thus, *Plk1_H* requires ongoing *Plk1* activation and transcription by *FoxM1*, and either induction by *FoxO3* or *FoxO1*, or prior accumulation.	

Table S1k: Cell_Cycle_Process module

	$\leftarrow$ Per	Plk1	Active mitotic *Plk1* is a prerequisite for the accumulation of the larger active *Plk1* pool denoted by *Plk1_H*.
	$\leftarrow$ TR	FoxM1	*Plk1* is a direct transcriptional target of *FoxM1*; loss of *FoxM1* severely reduces *Plk1* protein levels [264, 275].
	$\leftarrow$ Per	Plk1_H	Once accumulated, we assume that the *Plk1_H* pool of active *Plk1* remains stable in the absence of *FoxO*-mediated transcription. This is supported by negative feedback regulation of *FoxO* proteins by *Plk1* [21], indicating that ongoing high FoxO activity is likely not required for the maintenance of *Plk1_H*.
	$\leftarrow$ TR	FoxO3	*Plk1* is a direct transcriptional target of *FoxO3*, but *Plk1* appears to be sufficiently induced in the absence of *FoxO* preteens to aid its G2/M and mitotic functions. In contrast, accumulation if a large enough *Plk1* pool to briefly outlast APC/C^{Cdh1} activation (modeled by the *Plk1_H* node), requires *FoxO* activity in G2 [302].
	$\leftarrow$ TR	FoxO1	In addition to *FoxO3*, *FoxO1* also binds the *Plk1* promoter, potentially aiding its accumulation during G2 [368].
Ect2	**Ect2** = **f4N_DNA** and **Plk1_H** and **Cdh1** and not **U_Kinetochores** and not **A_Kinetochores** and **Bipolar_Spindle**		
	GEF	*Ect2* activaiton at the spindle midzone represents the step of cytokinesis in our model. Thus, *Ect2* requires *f4N_DNA*, high *Plk1* activity, as well as *Cdh1* for the assembly of a normal spindle midzone. Finally, *Ect2* cannot be recruited to the mid zone before anaphase is completed.	
	$\leftarrow$ Ind	f4N_DNA	Formation of a spindle midzone, where *Ect2* accumulates in preparation of cytokinesis requires recently separated sister chromatids (4N DNA content).
	$\leftarrow$ Ind	Plk1_H	*Plk1* activity in telophase (*Plk1_H*) is required for the recruitment of *Ect2* to the central spindle [308, 369].
	$\leftarrow$ Ind	Cdh1	APC/C^{Cdh1}-mediated destruction of Aurora kinase is required for the assembly of a robust spindle midzone at anaphase and for the normal timing of cytokinesis [370].
	$\dashv$ ComplProc	U _Kinetochores	Formation of a spindle midzone requires the separation of sister chromatids; thus it cannot occur before anaphase.
	$\dashv$ ComplProc	A _Kinetochores	Formation of a spindle midzone requires the separation of sister chromatids; thus it cannot occur before anaphase.
	$\leftarrow$ Loc	Bipolar _Spindle	The midzone of the bipolar spindle serves as source of signals that specify the location of contractile ring assembly and constriction, including recruitment of the *Ect2* RhoA GEF [371].
Cytokinesis	**Cytokinesis** = **Ect2** and **Bipolar_Spindle**		

Table S1k: Cell_Cycle_Process module

Target Node	Node Gate / Node Type / Link Type	Input Node	Description
	Proc		In contrast to our previous model in [372] where *Ect2* recruitment to the central spindle marked the start of cytokinesis, in [373] we introduced a separate *Cytokinesis* node to mark cytokinesis and the subsequent resetting of daughter cell DNA content to 2N by a separate node. In addition to *Ect2* recruitment, completion of cytokinesis also requires a bipolar spindle [371].
	← Ind	Ect2	At the start of cytokinesis, the *Ect2* RhoGEF is recruited to the central spindle [367]. *Ect2* aids the accumulation of GTP-bound *RhoA* [374] and the formation of the contractile ring. In our model, *Ect2* recruitment to the central spindle marks the start of cytokinesis.
	← Loc	Bipolar _Spindle	The midzone of the bipolar spindle specifes the location of contractile ring assembly and constriction [371].

Table S1l: DNA_Damage_Signaling module

Target Node	Node Gate / Node Type / Link Type	Node Description / Input Node	Link Description
cROS	**cROS** = not **Glucose** or **ROS_Ext** or **cROS_H**		
	Met	The *cROS* node represents a moderate rise in cytosolic ROS. This may be the result of glucose starvation [375] or external ROS exposure. The node is also ON by default if the cyrosolic levels are high (*cROS_H* = ON).	
	⊢ ComplProc	Glucose	Glucose starvation blocks glycolysis and the pentose phosphate pathway, resulting in oxidative stress due to enhanced ROS production (e.g. H_2O_2) [375].
	← Loc	ROS_Ext	External ROS molecules such as the nonpolar H_2O_2 can cross the cell membrane and raise cytosolic ROS levels.
	← Per	cROS_H	As the *cROS_H* node represents a higher cytosolic ROS burden with qualitatively different downstream consequences from the *cROS* node, *cROS_H* = ON is sufficient to keep the *cROS* node ON.
cROS_H	**cROS_H** = **cROS** and (**ROS_Ext** or (**mROS** and not **Glucose** and not **PGC1**))		
	Met	The *cROS_H* node represents high cytosolic ROS, above a threshold where its consequences are qualitatively distinct from those of the moderate *cROS* node (i.e., DNA damage leading to *ATM/ATR* activation and sustained high *p53* levels). In addition to moderate *cROS*, this requires external ROS or the combined effects of glucose starvation [375], mitochodrial ROS generation and a lack of *PGC-1α*-driven antioxidant response [180].	
	← Per	cROS	*cROS_H* = ON is a step up from moderate ROS levels and thus requires *cROS* = ON.
	← Loc	ROS_Ext	External ROS molecules such as the nonpolar H_2O_2 can cross the cell membrane and raise cytosolic ROS levels.

Table S1l: DNA_Damage_Signaling module

	⊣ ComplProc	Glucose	Glucose starvation blocks glycolysis and the pentose phosphate pathway, resulting in oxidative stress due to enhanced ROS production (e.g. H_2O_2) [375].
	← Loc	mROS	As mitochondria are the primary sources of intracellular ROS, increased mROS production can raise cytosolic levels as well.
	⊣ ComplProc	PGC1	*PGC-1α* increases the expression of mitochondrial antioxidant genes such as *MnSOD*, catalase, peroxiredoxins, *UCP2*, thioredoxin 2, thioredoxin reductase [180].
ATM	**ATM** = **cROS_H** or **CAD**		
	K	Ataxia-telangiectasia mutated, or ATM serine/threonine kinase, is recruited to and activated by DNA double-strand breaks [349]. In addition, oxidative stress can directly activate *ATM* [376].	
	← Cat	cROS_H	Elevated cytosolic ROS directly activte *ATM* by creating active, disulfide-linked dimers [376]. In addition, double-strand damage caused by ROS can also activate ATM indirectly [349].
	← Ind	CAD	*CAD*-induced DNA fragmentation during apoptosis activates ATM [377].
CHK2	**CHK2** = **ATM**		
	K	*ATM* kinase activates *CHK2* at DNA double strand breaks [351, 349].	
	← P	ATM	*ATM* kinase activates *CHK2* at DNA double-strand breaks, which blocks cell cycle progression [351, 349].
p53	**p53** = (**Nuclear_Membrane** and (((**AMPK** or (**ATR** and **CHK1**) or (**ATM** and **CHK2**))and(not **Mdm2**ornot **NF_kB**or**cROS**or(**mROS**andnot **SIRT3**)))))or**p53_2**or **p53_3** or **p53_4**		
	TF	*p53* is stabilized and its protein levels increase in response to stress. Here we use four Boolean nodes to track the sustained rise of *p53* and/or sustained oscillations that slowly boost/decrease mRNA expression in a subset of targets (others respond to the early rise of p53). We choose 4 steps because the time required for 4 consecutive pulses is a good qualitative match to the time it takes for *p53* to block *Cyclin B* expression during prolonged G2 arrest, *p21* mRNA expression saturates after 2-3 pulses [378, 258, 379]. The *p53* node represents the first level. Its activation requires a nuclear membrane (mitotic *p53* is sequestered to centrosomes) [380], phoshorylation by *AMPK* [381], *ATR/CHK1* or *ATM/CHK2* [351, 382], and either a lack of *Mdm2*-mediated degradation [383, 384, 385, 351], competition for co-activators with *NF-κB* [78], cytosolic ROS [386], or mitochondrial ROS in the absence of *SIRT3* [387]. In addition, the *p53* node is ON by default if any of the three nodes marking higher/prologed activation are also ON.	
	← Loc	Nuclear _Membrane	During mitosis, *p53* is recruited to and kept at centrosomes (a localization process aided by basal *ATM*-mediated Ser-15 phosphorylation), where it is dephosphorylated and kept inactive to allow cell cycle progression in cells with normal mitotic spindles [380]. Thus, we assume that the lack of a nuclear membrane keeps *p53* transcription OFF.

Table S1l: DNA_Damage_Signaling module

$\leftarrow$ P	AMPK	*AMPK* induces Ser-15 phosphorylation of *p53* to initiate an *AMPK*-dependent cell-cycle arrest [381].
$\leftarrow$ P	ATR	*ATR* induces Ser-15 phosphorylation of *p53* in response to single-strand DNA damage, and activates it with the aid of *CHK1* [351].
$\leftarrow$ P	CHK1	*CHK1/2* phosphorylate *p53* at Ser-20 [388] to prevent its ubiquitination by the ubiquitin E3 ligase *Mdm2* and subsequent proteosomal degradation [382].
$\leftarrow$ P	ATM	*ATR* induces Ser-15 phosphorylation of *p53* in response to double-strand DNA breaks, and activates it with the aid of *CHK2* [351].
$\leftarrow$ P	CHK2	*CHK1/2* phosphorylate *p53* at Ser-20 [388] to prevent its ubiquitination by the ubiquitin E3 ligase *Mdm2* and subsequent proteosomal degradation [382].
$\vdash$ Deg	Mdm2	*Mdm2*, induced by *p53* [389], is part of a negative feedback responsible of shutting down *p53* accumulation when damage signals turn off. *Mdm2* poly-ubiquitinates *p53*, targeting it for degradation [383, 384, 385, 351].
$\vdash$ BLoc	NF_kB	*NF-κB*-mediated inhibition of *p53* is likely the result of their competition for a limited pool of *p300/CBP complexes*, as they both require these transcriptional coactivators [78].
$\leftarrow$ Ind	cROS	ROS activate *p38 MAPK* (in addition to *ATM*), which in turn phosphorylates *p53* at Ser15, 33, 37 and 46, leading to its activation under increased ROS [386].
$\leftarrow$ Ind	mROS	ROS activate *p38 MAPK* (in addition to *ATM*), which in turn phosphorylates *p53* at Ser15, 33, 37 and 46, leading to its activation under increased ROS [386].
$\vdash$ Deacet	SIRT3	*SIRT3* deacetylates the mitochodrial pool of *p53*, reducing its transcriptional activity (*p53* localized to the nucleus as well as the mitocniodria when its levels rise above basal) [387].
$\leftarrow$ Per	p53_2	We assume that the transcriptional influence of *p53*, either from the accumulation of its active (Ser15-phosphorylated and acetylated) form or from repeated pulses of *p53* oscillation (in response to double-strand breaks; not modeled here), accumulates and dissapears stepwise from level 1 (*p53* node representing accumulation rought equivalent to a single pulse) to level 4 (*p53_4* node, transcritional activity on par with the effect of 4 consecutive pulses). Thus, the *p53* node is kept ON by default when nodes representing higher activity levels are ON.
$\leftarrow$ Per	p53_3	The *p53* node is kept ON by default when all nodes representing higher activity levels are ON (see *p53_2* in-link for details).
$\leftarrow$ Per	p53_4	The *p53* node is kept ON by default when all nodes representing higher activity levels are ON (see *p53_2* in-link for details).

Table S1l: DNA_Damage_Signaling module

p53_2	**p53_2** = ((**p53** or **p53_2**) and ((**AMPK** or (**ATR** and **CHK1**) or (**ATM** and **CHK2**)) and **cROS_H** and **Nuclear_Membrane**)) or **p53_3** or **p53_4**		
	TF		The *p53_2* node represents the second level/pulse of *p53* activation. It's accumulation and maintenance requires *p53* or *p53_2*, a nuclear membrane [380], phoshorylation by *AMPK* [381], *ATR/CHK1* or *ATM/CHK2* [351, 382], and high cytosolic ROS [386]. In addition, the *p53_2* node is ON by default if any of the two nodes marking higher/prologed activation are also ON.
	← Per	p53	We assume that the transcriptional influence of *p53*, either from the accumulation of its active (Ser15-phosphorylated and acetylated) form or from repeated pulses of *p53* oscillation, accumulates and dissapears stepwise from level 1 (*p53* node representing accumulation rought equivalent to a single pulse) to level 4 (*p53_4* node, transcritional activity on par with the effect of 4 consecutive pulses). Thus, the *p53_2* node turns ON/OFF when nodes representing the lower/higher activity levels are also ON/OFF.
	← Per	p53_2	The *p53_2* node can be maintained ON by ongoing activating signals.
	← Loc	Nuclear _Membrane	During mitosis, *p53* is recruited to and kept at centrosomes (a localization process aided by basal *ATM*-mediated Ser-15 phosphorylation), where it is dephosphorylated and kept inactive to allow cell cycle progression in cells with normal mitotic spindles [380]. Thus, we assume that the lack of a nuclear membrane keeps *p53* transcription OFF.
	← P	AMPK	*AMPK* induces Ser-15 phosphorylation of *p53* to initiate an *AMPK*-dependent cell-cycle arrest [381].
	← P	ATR	*ATR* induces Ser-15 phosphorylation of *p53* in response to single-strand DNA damage, and activates it with the aid of *CHK1* [351].
	← P	CHK1	*CHK1/2* phosphorylate *p53* at Ser-20 [388] to prevent its ubiquitination by the ubiquitin E3 ligase *Mdm2* and subsequent proteosomal degradation [382].
	← P	ATM	*ATR* induces Ser-15 phosphorylation of *p53* in response to double-strand DNA breaks, and activates it with the aid of *CHK2* [351].
	← P	CHK2	*CHK1/2* phosphorylate *p53* at Ser-20 [388] to prevent its ubiquitination by the ubiquitin E3 ligase *Mdm2* and subsequent proteosomal degradation [382].
	← Ind	cROS_H	ROS activate *p38 MAPK* (in addition to *ATM*), which in turn phosphorylates *p53* at Ser15, 33, 37 and 46, leading to its activation under increased ROS [386].
	← Per	p53_3	The *p53_2* node turns ON/OFF when all nodes representing the lower/higher activity levels are also ON/OFF.
	← Per	p53_4	The *p53_2* node turns ON/OFF when all nodes representing the lower/higher activity levels are also ON/OFF.

Table S1l: DNA_Damage_Signaling module

p53_3	**p53_3** = (((**p53_2** and **p53**) or **p53_3**) and ((**AMPK** or (**ATR** and **CHK1**) or (**ATM** and **CHK2**)) and **cROS_H** and **Nuclear_Membrane**)) or **p53_4**		
	TF		The *p53_3* node represents the third level/pulse of *p53* activation. It's accumulation and maintenance requires *p53* / *p53_2* or *p53_2*, a nuclear membrane [380], phoshorylation by *AMPK* [381], *ATR/CHK1* or *ATM/CHK2* [351, 382], and high cytosolic ROS [386]. In addition, the *p53_3* node is ON by default if *p53_4* is also ON.
	← Per	p53	We assume that the transcriptional influence of *p53*, either from the accumulation of its active (Ser15-phosphorylated and acetylated) form or from repeated pulses of *p53* oscillation, accumulates and dissapears stepwise from level 1 (*p53* node representing accumulation rought equivalent to a single pulse) to level 4 (*p53_4* node, transcritional activity on par with the effect of 4 consecutive pulses). Thus, the *p53_3* node turns ON/OFF when nodes representing the lower/higher activity levels are also ON/OFF.
	← Per	p53_2	The *p53_3* node turns ON/OFF when all nodes representing the lower/higher activity levels are also ON/OFF.
	← Per	p53_3	The *p53_3* node can be maintained ON by ongoing activating signals.
	← Loc	Nuclear _Membrane	During mitosis, *p53* is recruited to and kept at centrosomes (a localization process aided by basal *ATM*-mediated Ser-15 phosphorylation), where it is dephosphorylated and kept inactive to allow cell cycle progression in cells with normal mitotic spindles [380]. Thus, we assume that the lack of a nuclear membrane keeps *p53* transcription OFF.
	← P	AMPK	*AMPK* induces Ser-15 phosphorylation of *p53* to initiate an *AMPK*-dependent cell-cycle arrest [381].
	← P	ATR	*ATR* induces Ser-15 phosphorylation of *p53* in response to single-strand DNA damage, and activates it with the aid of *CHK1* [351].
	← P	CHK1	*CHK1/2* phosphorylate *p53* at Ser-20 [388] to prevent its ubiquitination by the ubiquitin E3 ligase *Mdm2* and subsequent proteosomal degradation [382].
	← P	ATM	*ATR* induces Ser-15 phosphorylation of *p53* in response to double-strand DNA breaks, and activates it with the aid of *CHK2* [351].
	← P	CHK2	*CHK1/2* phosphorylate *p53* at Ser-20 [388] to prevent its ubiquitination by the ubiquitin E3 ligase *Mdm2* and subsequent proteosomal degradation [382].
	← Ind	cROS_H	ROS activate *p38 MAPK* (in addition to *ATM*), which in turn phosphorylates *p53* at Ser15, 33, 37 and 46, leading to its activation under increased ROS [386].
	← Per	p53_4	The *p53_3* node turns ON/OFF when all nodes representing the lower/higher activity levels are also ON/OFF.

Table S1l: DNA_Damage_Signaling module

p53_4	**p53_4** = ((**p53** and **p53_2** and **p53_3**) or **p53_4**) and ((**AMPK** or (**ATR** and **CHK1**) or (**ATM** and **CHK2**)) and **cROS_H** and **Nuclear_Membrane**)		
	TF	The *p53_4* node represents the fourth and highest level/pulse of *p53* activation. It's accumulation and maintenance requires *p53* / *p53_2* / *p53_3* or *p53_4*, a nuclear membrane [380], phoshorylation by *AMPK* [381], *ATR/CHK1* or *ATM/CHK2* [351, 382], and high cytosolic ROS [386].	
	← Per	p53	We assume that the transcriptional influence of *p53*, either from the accumulation of its active (Ser15-phosphorylated and acetylated) form or from repeated pulses of *p53* oscillation, accumulates and dissapears stepwise from level 1 (*p53* node representing accumulation rought equivalent to a single pulse) to level 4 (*p53_4* node, transcritional activity on par with the effect of 4 consecutive pulses). Thus, the *p53_4* node turns ON/OFF when nodes representing the lower/higher activity levels are also ON/OFF.
	← Per	p53_2	The *p53_4* node turns ON/OFF when all nodes representing the lower/higher activity levels are also ON/OFF.
	← Per	p53_3	The *p53_4* node turns ON/OFF when all nodes representing the lower/higher activity levels are also ON/OFF.
	← Per	p53_4	The *p53_4* node can be maintained ON by ongoing activating signals.
	← Loc	Nuclear _Membrane	During mitosis, *p53* is recruited to and kept at centrosomes (a localization process aided by basal *ATM*-mediated Ser-15 phosphorylation), where it is dephosphorylated and kept inactive to allow cell cycle progression in cells with normal mitotic spindles [380]. Thus, we assume that the lack of a nuclear membrane keeps *p53* transcription OFF.
	← P	AMPK	*AMPK* induces Ser-15 phosphorylation of *p53* to initiate an *AMPK*-dependent cell-cycle arrest [381].
	← P	ATR	*ATR* induces Ser-15 phosphorylation of *p53* in response to single-strand DNA damage, and activates it with the aid of *CHK1* [351].
	← P	CHK1	*CHK1/2* phosphorylate *p53* at Ser-20 [388] to prevent its ubiquitination by the ubiquitin E3 ligase *Mdm2* and subsequent proteosomal degradation [382].
	← P	ATM	*ATR* induces Ser-15 phosphorylation of *p53* in response to double-strand DNA breaks, and activates it with the aid of *CHK2* [351].
	← P	CHK2	*CHK1/2* phosphorylate *p53* at Ser-20 [388] to prevent its ubiquitination by the ubiquitin E3 ligase *Mdm2* and subsequent proteosomal degradation [382].
	← Ind	cROS_H	ROS activate *p38 MAPK* (in addition to *ATM*), which in turn phosphorylates *p53* at Ser15, 33, 37 and 46, leading to its activation under increased ROS [386].

Table S1l: DNA_Damage_Signaling module

Mdm2	**Mdm2** = not(**ATM** or **ATR**) and (**AKT_H** or **p53** or **p53_2** or **p53_3** or **p53_4**) and not(**Casp2** or **Casp3**)		
	UbL		The *Mdm2* E3 ubiquitin-protein ligase is a key inhibitor of *p53* accumulation [385, 384]. It's activity is inhibited by *ATM/ATR*-mediated phosphorylation [351], while its levels are increased by *AKT* phosphorylation (stabilization) or *p53*-mediated transcription. Finally, *Caspase2/3* cleave and deactivate *Mdm2*.
	⊢ P	ATM	*ATM* phosphorylates *Mdm2* at Ser-395, leading to its deactivation as well as rapid degradation [351].
	⊢ P	ATR	*ATR* phosphorylates *Mdm2* at Ser-407, inhibiting its ability to bind and block *p53* accumulation [351].
	← P	AKT_H	*AKT* phosphorylates *Mdm2* at Ser-183, inreasing its nuclear stability and decreasing *p53* levels [390].
	← TR	p53	*p53* is a direct transcriptional inducer of *Mdm2*[383]. As its levels rise rapidly within the frist few hours of *p53* activation (see SM data in [378]), we model it as induced by any of the four *p53* nodes.
	← TR	p53_2	*p53* is a direct transcriptional inducer of *Mdm2*[383].
	← TR	p53_3	*p53* is a direct transcriptional inducer of *Mdm2*[383].
	← TR	p53_4	*p53* is a direct transcriptional inducer of *Mdm2*[383].
	⊢ Lysis	Casp2	*Caspase 2* cleaves *Mdm2* at Asp-367, removing its C-terminal RING domain required for *p53* ubiquitination [391].
	⊢ Lysis	Casp3	*Caspase 2* cleaves and deactivates *Mdm2* [392].
p21_H	**p21_H** = **p21_mRNA** and **p21** and **p53_3** and not **Casp3**		
	CDKI		The *p21_H* node represents high, cell cycle arresting and senescence-promoting levels of *p21* accumulation in response to damage. Thus, this node requires *p21* mRNA expression, at least basal *p21*, and sustained increase / pulsing of *p53* (node *p53_3*) [393], as well as the absence of *Caspase 3*-mediated cleavage [189].
	← TL	p21 _mRNA	Increased mRNA levels are required to raise *p21* protein levels [378].
	← Per	p21	At least basal *p21* protein activity is required before the high *p21* node turns ON.
	← TR	p53_3	*p21* mRNA levels increase with consecutive *p53* pulses, reaching saturation after 3-4 pulses on average [393]. Thus we chose to link our *p21_H* node to *p53_3*.
	⊢ Lysis	Casp3	*Caspase 3* cleaves and deactivates *p21* [189].

Table S1m: TRAIL module

Target Node	Node Gate		
	Node Type	Node Description	
	Link Type	Input Node	Link Description
Trail	**Trail** = **Trail**		
	Env	The *Trail* node represents environmental availability of the *Trail* protein outside the cell.	
	← Env	Trail	The *Trail* input node remains on/off if set ON/OFF in the absence of *in silico* perturbation.

Table S1n: Apoptotic_SW module

Target Node	Node Gate		
	Node Type	Node Description	
	Link Type	Input Node	Link Description
DR4_5	**DR4_5** = **Trail**		
	Rec	The *DR4* and *DR5* death receptors, represented by the *DR4_5* node, are activated by extracellular *Trail* [394].	
	← Ligand	Trail	*DR4* and *DR5* death receptors are activated by extracellular *Trail* [394].
Casp8	**Casp8** = **DR4_5** or **Casp3**		
	PTase	*Pro-Caspase 8* may be cleaved independently by *DISC* (not directly reprsented; an adaptor protein for *DR4_5*) or *Caspase 3*.	
	← Compl	DR4_5	*Trail*-bound (active) *DR4* and *DR5* receptors trigger the assembly of the pro-apoptotic death-inducing signaling complex (*DISC*), which binds a cluster of *pro-Caspase 8* proteins and initiates their cleavage into active *Caspase 8* [395].
	← Ind	Casp3	*Caspase 3* indirectly activates *Caspase 8* by cleaving *Caspase 6* [396], which, in turn, cleaves *Caspase 8* [397].
Casp2	**Casp2** = (**U_Kinetochores** and **Mad2** and not(**CyclinB** and **Cdk1** and **Bipolar_Spindle**)) or **Casp3**		
	PTase	*Pro-caspase 2* is cleaved and activated by *Caspase 3*, or by failed cytokinesis marked by the presence of unattached kinetochores, an active SAC, and the absence of active *Cyclin B/Cdk1* complexes alongside a properly formed bipoar spindle to phosphorylate and inhibit *Caspase 2*.	

Table S1n: Apoptotic_SW module

	← Ind	U _Kinetochores	Although the precise molecular mechanism by which *Caspase 2* is activated during prolonged or stalled mitosis is unclear, its activation platform, the *PIDDosome*, has been localized to unattached kinetochores [398]. Even though a checkpoint protein keeps the *PIDDosome* unresponsive to DNA damage signals, the loss of protective *Cyclin B/Cdk1* phosphorylation only leads to *Caspase 2* activation in the presence of a partially assembled mitotic spindle, and requires active SAC.
	← Ind	Mad2	A functional spindle assembly checkpoint is required for mitotic cell death upon prolonged mitotic arrest [399] or spindle damage [400].
	⊣ P	CyclinB	*Cyclin B1/Cdk1* phosphorylate *caspase-2* at Ser 340, preventing its activation [401].
	⊣ P	Cdk1	*Cyclin B1/Cdk1* phosphorylate *caspase-2* at Ser 340, preventing its activation [401].
	⊣ P	Bipolar _Spindle	Abberant mitotic spindle formation leads to *Caspase 2* activation followed by mitotic catastrophe [402, 403].
	← Lysis	Casp3	*Caspase 2* is a target of *Caspase 3*, as its inhibition severely limits *Caspase 2* cleavage during apoptosis [404, 405].
MCL_1	**MCL_1** = not **Casp3** and not **Casp2** and (not **GSK3** or (**AKT_B** and (**ERK** or not **E2F1**))) and not(**Cdk1** and **CyclinB** and **U_Kinetochores**)		
	Prot		*Caspase 3* or *2*-mediated destruction of *MCL-1* must be absent for *MCL-1* to be ON. Avoiding degradation via textitGSK3 requires the *GSK3*-weakenjng presence of basal *AKT* activity (*AKT_B*) [406] and either *ERK*-mediated stabilization, or the absence of its repressor *E2F1*. Finally, during mitotic arrest (*U_Kinetochores*), *MCL-1* is deactivated by *Cyclin B/Cdk1* phosphorylation, which shields it from the *PPA2*-mediated dephosphorylation of its degradation-targeting sites [407].
	⊣ Lysis	Casp3	*Caspase 3* cleaves and deactivated *MCL-1* [408].
	⊣ Lysis	Casp2	*Caspase 2* activation destabilizes the *MCL-1* protein [409].
	⊣ P	GSK3	*MCL-1* is phosphorylated by *GSK3*, leading to ubiquitinylation and degradation of Phosphorylation [406].
	← Ind	AKT_B	In order to account for the loss of *MCL-1* in the complete absence of growth factors versus its presence in low growth factor environments, we required basal *AKT* to modulate the strength of *GSK3* inhibition [406].
	← P	ERK	*ERK* phosphorylates *MCL-1*, promoting its interaction with *Pin1*, which stabilizes it [410, 411].
	⊣ TR	E2F1	*E2F1* is a direct transcriptional repressor of *MCL-1* [412].
	⊣ P	CyclinB	In cells arrested in mitosis, phosphorylation by *Cyclin B/Cdk1* on T92 initiates *MCL-1* degradation [413].

Table S1n: Apoptotic_SW module

	⊢ P	Cdk1	Phosphorylation by *Cyclin B/Cdk1* in cells arrested in mitosis initiates *MCL-1* degradation [413].
	⊢ Ind	U _Kinetochores	During prolonged mitotic arrest (*U_kinetochores*), *MCL-1* levels drop steadily due to phosphorylation by *JNK*, *p38* and/or *CKII* and its subsequent degradation by the E3 ubiquitin ligase *SCF* (*FBW7*) [407].
BCLXL	**BCLXL** = not **Casp3** and (**BCL2** or not **BAD**) and (not **U_Kinetochores** or (**Plk1** and (not(**CyclinB** and **Cdk1**) or (**BCL2** and **MCL_1**) or **Bipolar_Spindle**)) or ((**BCL2** and **MCL_1**) and (not(**CyclinB** and **Cdk1**))))		
	Prot		*Bcl-xL* activity requires the absence of *Caspase 3*. In addition, *BAD* can block *Bcl-xL* when *BCL2* is also OFF. Finally, mitotic *BCL-xL* can be inhibited by *Cdk1* activity if either *BCL2*, *MCL-1*, or *Plk1* are OFF, or bipolar spindle formation is compromised. In the absence of *Plk1*, loss of either *BCL2* or *MCL-1* can result in *Bcl-xL* inhibition (even without *Cdk1* phosphorylation), as we assume its targets are no longer competitively bound by its family members.
	← Ind	Plk1	In addition to other effects of prolonged mitotic arrest on *BCL-2* proteins, *Plk1* inhibition synergistically enhances the inhibitory phosphorylation of *BCL-2* and *BCL-xL*, as well as downregulation of *MCL-1* [414].
	⊢ P	CyclinB	During normal mitosis, *Cyclin B/Cdk1* only transiently phosphorylates part of the *BCL-xL* pool. Prolonged mitosis, however, results in high levels of *BCL-xL* (and *Bcl-2*) phosphorylation, priming the system for *Caspase 2*-mediated apoptosis [415, 416].
	⊢ P	Cdk1	During normal mitosis, *Cyclin B/Cdk1* only transiently phosphorylates part of the *BCL-xL* pool. Prolonged mitosis, however, results in high levels of *BCL-xL* (and *Bcl-2*) phosphorylation, priming the system for *Caspase 2*-mediated apoptosis [415, 416].
	← Ind	Bipolar _Spindle	Prolonged mitosis, without proper bipolar spindle formation leading to SAC passage, is required for the accumulation of *BCL-xL* phosphorylation, which weaken its interaction with *Bax* [417].
	⊢ Ind	U _Kinetochores	Prolonged mitosis is required for the accumulation of *BCL-xL* phosphorylation, weakening its interaction with *Bax* [417].
	← Ind	MCL_1	*MCL-1* competes with *BCL-xL* for *BAK* binding; the presence of *MCL-1* can keep part of the *BCL-xL* pool active [418].
	← Ind	BCL2	*BCL2* competes with *BCL-xL* for *BAD* binding. Although *BCL-xL* is a stronger binding partner, we assume that *BAD* alone cannot fully block *BCL-xL* in the presence of *BCL2* [419].
	⊢ IBind	BAD	*Bad* can bind *BCL-xL* and displace it from *BAX*, thus deactivating it [419].

Table S1n: Apoptotic_SW module

	⊢ Lysis	Casp3	*BCL2* is cleaved and deactivated by *Caspase 3* [420].
BCL2	**BCL2** = not(**Casp3** or **BAD** or **BIM** or **BIK** or (**BID** and **HIF1**)) and ((not **U_Kinetochores** or (**MCL_1** and **BCLXL**)) or (**Plk1** and (**BCLXL** or **MCL_1** or not(**Cdk1** and **CyclinB**))))		
	Prot	While the precise combinatorial logic governing *BCL2* activity is not clear from literature, we modeled *BCL2* as ON in the absence of *Caspase 3*, *BAD*, *BIM* or *BIK*. This choice makes *BCL2* the most sensitive of the three family members to activation of its three inhibitors. In addition, mitotic *BCL2* is blocked by *Cdk1* if both *BCL-xL* and *MCL-1* are OFF. In the absence of *Plk1*, loss of either *BCL2* or *MCL-1* can result in *BCL-2* inhibition (even without *Cdk1* phosphorylation), as we assume its targets are no longer competitively bound by its family members. In order to account for the apoptosis-sensitising role of *HIF-1*α, we assume that truncated *BID* can also repress *BCL2* when *HIF-1*α activation ups the ratio of *BAX* to *BCL2* [421].	
	⊢ Lysis	Casp3	*BCL2* is cleaved and deactivated by *Caspase 3* [420].
	⊢ IBind	BAD	*BCL2* competes with *BCL-xL* for *BAD* binding. *BAD* displaces *BCL2* from its inhibitory binding of *Bax/Bak*. Although *BCL-xL* is a stronger binding partner, we assume that *BAD* alone cannot fully block *BCL-xL* in the presence of *BCL2* [419].
	⊢ IBind	BIM	*BIM* binds *BCL2* and they mutually inhibit each other's ability to activate further targets [422].
	⊢ IBind	BIK	*BIK* binds *BCL2* and they mutually inhibit each other's activity [423].
	⊢ IBind	BID	*BID* binds *BCL2* and they mutually inhibit each other's ability to activate further targets [424]. We assume that *tBID* can repress *BCL2* when *HIF-1*α is active, as it increased the ratio of *BAX* to *BCL2* proteins [421].
	⊢ Ind	HIF1	*HIF-1*α was shown to increase the ratio of *Bax* to *BCL2* proteins, as well as upregulate the levels of extrinsic death signaing proteins (including *Trail* and *DR4/5*) while down-regulating *c-FLIP* [421]. Thus, we assume that *HIF-1*α can boost *Trail* signaling to overpower *BCL2* even in the presence of potent survival signals.
	⊢ Ind	U _Kinetochores	Prolonged mitosis is required for the accumulation of *BCL2* phosphorylation [415, 416, 420].
	← Ind	MCL_1	*MCL-1* competes with *BCL-xL* for binding most of their apoptotic partners, including *BIK*, *BIM*, *BID*, *BAX* and *BAK*.
	← Ind	BCLXL	*BCL2* competes with *BCL-xL* for binding most of their apoptotic partners, including *BIK*, *BIM*, *BID*, *BAX* and *BAK*.

Table S1n: Apoptotic_SW module

	← Ind	Plk1	In addition to other effects of prolonged mitotic arrest on *BCL2* proteins, *Plk1* inhibition synergistically enhances the inhibitory phosphorylation of *BCL2* and *BCL-xL*, as well as downregulation of *MCL-1* [414].
	⊢ P	CyclinB	*Cyclin B/Cdk1* phosphorylates *BCL2* (and *BCL-xL*) during mitosis [415, 416, 420].
	⊢ P	Cdk1	Prolonged mitosis results in high levels of *BCL-xL* and *BCL2* phosphorylation, priming the system for *Caspase 2*-mediated apoptosis [415, 416, 420].
BAD	**BAD** = **Casp3** or not(**AKT_H** or **AKT_B** or **ERK** or **S6K**) or (**Casp8** and not(**AKT_B** and **ERK** and **S6K**) and not(**AKT_H** and (**AKT_B** or **ERK**)))		
	Prot		*BAD* in our model is ON when cleaved by *Caspase 3*, or in the complete absence of survival signals (*AKT*, *ERK* or *S6K*). Alternatively, *BAD* can be cleaved and activated by *Caspase 8* in the absence of strong survival signaling. We modeled this inhibitory survival signal as either the combined activity of *ERK*, *S6K* and (at least) basal *AKT*, or high *AKT* in the joint presence of ERK and basal *AKT* (indicating that *AKT_H* will not drop by the next time-step).
	← Lysis	Casp3	*Caspase 3* cleaves *BAD*, generating a more potently apoptotic fragment [425].
	⊢ P	AKT_H	*Akt* phosphorylates *BAD* at Ser-136, inducing its sequestration away from the mitochondrial membrane where its BCL-2 family targets are located (e.g., *BCL2*, *BCL-xL*) [426].
	⊢ P	AKT_B	*Akt* phosphorylates *BAD* at Ser-136, inducing its sequestration away from the mitochondrial membrane where its BCL-2 family targets are located (e.g., *BCL2*, *BCL-xL*) [426].
	⊢ P	ERK	*ERK* phosphorylates *BAD* at Ser-112, inducing its sequestration away from the mitochondrial membrane where its BCL-2 family targets are located (e.g., *BCL-2*, *BCL-xL*)[427].
	⊢ P	S6K	*S6K1* phosphorylates *BAD* at Ser-155, directly blocking its binding to *BCL-xL* [428].
	← Lysis	Casp8	*Caspase 8* is also able to cleave *BAD*, generating a more potently apoptotic fragment [425]. In addition, *TRAIL*-mediated apoptosis results in *BAD* cleavage by a Caspase upstream of MOMP, creating a potent apoptotic inducer before full *Caspase 3* activation [429].
BIK	**BIK** = not(**MCL_1** or **BCLXL** or **BCL2**)		
	Prot		*BIK* is free to activate its target, *BAX*, only when it is not sequestered by any of the three BCL-2 family proteins in our model [418].
	⊢ IBind	MCL_1	*MCL-1* binds *BIK*; they mutually inhibit each other [430].
	⊢ IBind	BCLXL	*BCL-xL* binds *BIK*; they mutually inhibit each other [431].

Table S1n: Apoptotic_SW module

	⊣ IBind	BCL2	*BCL2* binds *BIK*; they mutually inhibit each other [423].
BIM	**BIM** = **FoxO3** and **GSK3** and not(**ERK** or **MCL_1** or **BCLXL** or **BCL2**)		
	Prot	*BIM*'s pro-apoptotic activity requires expression driven by *FoxO3* and aided by *GSK3* and the absence of all three inhibitory *BCL2* family proteins.	
	← TR	FoxO3	*FoxO3* is a transcriptional activator of *BIM* [432].
	← Ind	GSK3	*GSK3* kinase is likely required for the *AP1*-dependent expression of *BIM* [433].
	⊣ Ind	ERK	The *MEK/ERK* pathway represses *BIM* protein levels, likely via transcriptional repression [434].
	⊣ IBind	MCL_1	*MCL-1* binds *BIM* and inhibits its apoptotic activity [435].
	⊣ IBind	BCLXL	*BCL-xL* binds *BIM* and inhibits its apoptotic activity [422].
	⊣ IBind	BCL2	*BLC2* binds *BIM* and inhibits its apoptotic activity [422].
BID	**BID** = **Casp8** or (**Casp2** and (not(**BCL2** or **BCLXL** or **MCL_1**) or **ATM**))		
	Prot	*BID* is truncated in response to *Caspase 8* activation. In addition, *Caspase 2* can also promote *BID* activation once all three pro-apoptotic *BCL2* family proteins are blocked.	
	← Lysis	Casp8	In response to *TRAIL* (or *FAS* ligand), the initiator *Caspase 8* cleaves *BID* to its active truncated form [436, 437, 438].
	← Lysis	Casp2	*Caspase 2* cleaves *BID* to its active truncated form [439].
	⊣ IBind	BCL2	All three anti-apoptotic BCL2 proteins (*BCL2*, *BCL-xL* and *MCL-1*) sequesters *BID* into stable complexes, preventing them from activating *BAX* or *BAK* [440].
	⊣ IBind	BCLXL	All three anti-apoptotic BCL2 proteins (*BCL2*, *BCL-xL* and *MCL-1*) sequesters *BID* into stable complexes, preventing them from activating *BAX* or *BAK* [440].
	⊣ IBind	MCL_1	All three anti-apoptotic BCL2 proteins (*BCL2*, *BCL-xL* and *MCL-1*) sequesters *BID* into stable complexes, preventing them from activating *BAX* or *BAK* [440].
	← P	ATM	*ATM* phosphorylates BID during mitosis, priming it for activation during mitotic catastrophe [441, 442]. Here we assume that this primed form of BID is more potentlty cleaved by *Caspase 2*, while it was shown not to alter its cleavage by *Caspase 8* [441].
BAK	**BAK** = (**BID** and (**BIM** or **BIK** or not(**BCL2** and **BCLXL** and **MCL_1**))) or ((**BIM** or **BIK**) and not(**BCLXL** or **MCL_1**))		

Table S1n: Apoptotic_SW module

	Prot	Given that *BAK* is preferentially activated by *BID* compared to *BIM* [443] and that it is less responsive to sequestration by *BCL2* than the other two anti-apoptotic *BCL2* family proteins [444, 445], *BAK* in our model turns on when stimulated by *BID* if one or more *BCL2* family proteins are absent, or if *BIM* or *BIK* are also present. In contrast, *BIM* or *BIK* only activate *BAK* if *BCL-xL* and *MCL-1* are absent (*BCL-2* alone cannot block them).	
	$\leftarrow$ Compl	BID	Activated (truncated) *BID* binds to mitochondrial *BAK*, resulting in its activation and oligomerization in the mitochondrial membrane, followed by *cytochrome c* release [446].
	$\leftarrow$ Compl	BIM	*BAK* is preferentially activated by *BID* compared to *BIM*, but *BIM* can also promote BAK oligomerization [443].
	$\leftarrow$ Compl	BIK	*BIK* can aid the activation of both *BAK* and *BAX* by triggering *BAK* oligomerization on the ER membrane and promoting a Ca^{2+} efflux required for the fragmentation of hyper fused mitochondrial tubules, aiding *BAK* and *BAX* activation [447].
	$\vdash$ IBind	BCL2	*BCL2* can also bind *BAK* to prevent its oligomerization, but it does so less potently than the other two BCL-2 family members [444, 445, 448].
	$\vdash$ IBind	BCLXL	*BCL-xL* binds *BAK* and prevent its oligomerization in the mitochondrial membrane [449, 444, 445].
	$\vdash$ IBind	MCL_1	*MCL-1* binds *BAK* and prevent its oligomerization in the mitochondrial membrane [444, 445].
BAX	**BAX** = (**BIM** and (**BID** or **BIK** or not(**BCL2** and **BCLXL** and **MCL_1**))) or ((**BID** or **BIK**) and not(**BCL2** or **BCLXL**))		
	Prot	In contrast to *BAK*, *BAX* is preferentially activated by *BIM* compared to *BID* [443] and it is less responsive to sequestration by *MCL-1* than the other two anti-apoptotic BCL2 family proteins [444, 445]. *BAX* in our model turns on when stimulated by *BIM* if one or more *BCL2* family proteins are absent, or if *BID* or *BIK* are also present. In contrast, *BID* or *BIK* only activate *BAK* if *BCL2* and *BCL-xL* are both absent (*MCL-1* alone cannot block them).	
	$\leftarrow$ Compl	BIM	Activated *BIM* binds to mitochondrial *BAX*, resulting in its allosteric activation and oligomerization in the mitochondrial membrane, leading to *cytochrome c* release [443].
	$\leftarrow$ Compl	BID	*BAX* is preferentially activated by *BIM* compared to *BID*, but *BID* can also promote *BAK* oligomerization [443].
	$\leftarrow$ Compl	BIK	*BIK* can aid the activation of both *BAK* and *BAX* by triggering *BAK* oligomerization on the ER membrane and promoting a Ca^{2+} efflux required for the fragmentation of hyper fused mitochondrial tubules, aiding *BAK* and *BAX* activation [447].
	$\vdash$ IBind	BCL2	*BCL2* binds *BAX* and prevent its oligomerization in the mitochondrial membrane [444, 445].
	$\vdash$ IBind	BCLXL	*BCL-xL* binds *BAX* and prevent its oligomerization in the mitochondrial membrane [444, 445].

Table S1n: Apoptotic_SW module

	⊣ IBind	MCL_1	*MCL-1* can also bind *BAK* to prevent its oligomerization, but it does so less potently than the other two BCL-2 family members [444, 445, 450].
Cyto_C	**Cyto_C** = **BAX** or **BAK** or (not **Mps1** and **Drp1** and **Unfused** and not **Mitophagy_High**)		
	Prot	*Cytochrome C* release from mitochondria requires the oligomerization of either *BAK* or *BAX* [451]. In addition, *Drp1*-mediated mitochondrial fragmentation can aid cytochrome C release [452, 453], as long as the SAC protein *Mps1* [454] or high levels of mitophagy [455] do not counteract it.	
	← Loc	BAK	*BAK* oligomerization at the mitochondrial membrane triggers MOMP, which results in the release of *cytochrome C* from mitochondria [451].
	← Loc	BAX	*BAX* oligomerization at the mitochondrial membrane triggers MOMP, which results in the release of *cytochrome C* from mitochondria [456, 457].
	⊣ Ind	Mps1	During mitosis, fragmented mitochodria are blocked from leaking cytochrome C and killing the cell by mitochodrial recruitment of the SAC protein *Mps1* to the voltage-dependent anion channel 1 (*VDAC1*) [454].
	⊣ Ind	Drp1	*Drp1* null cells show blunted and delayed cytochrome C release, due to a lack of *Drp1*-dependent remodelling and opening of cristae, where the majority of cytochrome c resides [453].
	← ComplProc	Unfused	Mitochondrial fission, which leads to loss of OPA1 function, opens the cristae junctions and releases cytochrome C [452].
	⊣ ComplProc	Mitophagy_High	High levels of mitophagy can engulf mitochondria debris and cytochrome C, and blunt the apoptosis signal [455].
SMAC	**SMAC** = **BAX** or **BAK**		
	Prot	*SMAC/Diablo* release from mitochondria requires the oligomerization of either *BAK* or *BAX* [451, 458].	
	← Loc	BAK	*BAK* oligomerization at the mitochondrial membrane triggers MOMP, which results in the release of *SMAC/Diablo* from mitochondria [451, 458].
	← Loc	BAX	*BAX* oligomerization at the mitochondrial membrane triggers MOMP, which results in the release of *SMAC/Diablo* from mitochondria [451, 458].
IAPs	**IAPs** = **AKT_H** or not **SMAC**		
	Prot	Inhibitor of Apoptosis Proteins (*IAP*s) are active in the absence of *SMAC* inhibition, or following *AKT_H* mediated upregulation (this protection from *SMAC* requires peak or oncogenic *AKT* activity).	
	← Ind	AKT_H	*cIAP-2* and *XIAP* are both transcriptionally up-regulated in response to strong *PI3K/AKT1* activation [459].
	⊣ IBind	SMAC	*SMAC/Diablo* binds tightly to *IAP* proteins and blocks their ability to inhibit *Caspase 3* [460].

Table S1n: Apoptotic_SW module

Casp9	**Casp9** = (not **IAPs** and **Cyto_C**) or **Casp3**		
	PTase	*Procaspase 9* is cleaved into active *Caspase 9* by *Caspase 3*, or by the apoptosome (which relies on *cytochrome C* for its assembly) in the absence of *IAP* proteins.	
	⊢ IBind	IAPs	*XIAP*, *cIAP1* and *cIAP2* inhibit the *cytochrome c*-induced activation of *procaspase-9* [461].
	← Compl	Cyto_C	*Cytochrome c* binds to *APAF-1* proteins, promoting their assembly into the apoptosome, a platform for *procaspase 9* binding and cleavage into its active form [462].
	← Lysis	Casp3	*Procaspase 9* is a direct cleavage target of *Caspase 3* [405].
Casp3	**Casp3** = (**Casp9** and **Casp8**) or (**Casp3** and (**Casp9** or **Casp8**)) or (not **IAPs** and (**Casp9** or **Casp8** or **Casp3**))		
	PTase	Activation of *Caspase 3* requires proteolytic cleavage of *procaspase-3* by initiator caspases such as *Caspase 9* or *Caspase 8*. In our model, cooperation of two of the three caspases (*Casp9*, *Casp8*, *Casp3*) is required in the presence of *IAP*s, which inhibit the proteolytic activity of *Caspase 3* by bind tightly to its active site. In the absence of *IAP*s, either of the three caspases can cleave and activate *Caspase 3*.	
	← Lysis	Casp9	Active *Caspase 9* cleaves *procaspase 3* [463].
	← Lysis	Casp8	*Caspase 8* can cleave *Caspase 3* [464], but full *Caspase 3* activation also requires MOMP (potentially due to a need for *IAP* inhibition) [465].
	← Per	Casp3	Once activated, *Caspase 3* helps sustain its own activation by cleaving *procaspase 8* and *6*. *Caspase 6*, in turn, generates additional active *caspase 8* and *9*. Together they all sustains a continuing active pool of Caspase 3.
	⊢ IBind	IAPs	*IAP*s bind tightly to the active site of *Caspase 3*, keeping its activity in check [461, 466].

Table S1o: DNA_Fragmentation module

Target Node	Node Gate<br>Node Type<br>Link Type	Node Description<br>Input Node	Link Description
CAD	**CAD** = **Casp3** and **Casp9**		

Table S1o: DNA_Fragmentation module

DNase	Caspase-activated DNase (*CAD*) is activated when its inhibition is released via the cleavage of *ICAD* (inhibitor of caspase-activated DNase). While *Capsase 3* and *7* (a direct target of *Caspase 9*) can inhibit *ICAD* [467], in our model they are both required, as *CAD* = ON is represents terminal, irreversible apoptotic commitment, which is fully locked in when both *Caspase 3* and *9* are on.	
$\leftarrow$ Ind	Casp3	*Caspase 3* relives *CAD* inhibition by cleaving its inhibitor *ICAD* [467].
$\leftarrow$ Ind	Casp9	In addition of *Caspase 3*, *CAD* inhibition can also be relieved by *ICAD* cleavage by *Caspase 7*, which is a direct target of *Caspase 9* [467].

Table S2a: Key to Node Type Symbols

Symbol	Node Type	Description
Cell	Cell	Node type used when a node's state is used to represent discrete phenotypes of an entire cell; appropriate for multi models.
DM	DM_Switch	Multi-stable regulatory module (Dynamically Modular Switch) in a higher-level model layer.
Conn	Connector	Grouping of nodes that are either mono-stable, or do not form a switch-like circuit that controls discrete phenotype transitions. For example, signaling input layers without feedback, or multi-step links between distinct switches can be represented as connector nodes in coarse-grained (switch-level) models.
Env	Environment	Nodes or modules that represent the extracellular environment of a single cell or cell collective. These are self-sustaining nodes or node groups that maintain their initial states and receive no feedback from the rest of the network (they act as inputs).
Proc	Process	Nodes or modules that stand in for complex cellular processes not modeled in detail (e.g., DNA replication or the process of aligning chromosomes at the metaphase plane during mitosis).
MSt	Macro_Structure	Nodes or modules that represent the state of large, complex cellular structures such as DNA content, cytoskeletal features, junctions or mitochondria.
Met	Metabolite	Regulatory node representing a metabolite (not protein, gene product or complex structure).
mRNA	MRNA	mRNA.
miR	MicroRNA	microRNA.
PC	Protein_Complex	Protein complex represented by a single node or via a key member of the complex.
Rec	Receptor	Cell surface receptor protein or complex.
Adap	Adaptor_Protein	Protein that helps scaffold a signaling complex or other large assembly of proteins.
Secr	Secreted_Protein	Protein secreted into the extracellular environment, such that the state of the node tagged with this type represents the availability fo this protein outside the cell.
TF	TF_Protein	Transcription factor.
K	Kinase	Kinase (enzyme that catalyzes the phosphorylation of its target).
Ph	Phosphatase	Phosphatase (enzyme that catalyzes the removal of phosphorylation from its target).
UbL	Ubiquitin_Ligase	Ubiquitin ligase (protein that recruits an ubiquitin-conjugating enzyme that has been loaded with ubiquitin to a target protein and assists or directly catalyzes the transfer of ubiquitin from the ubiquitin-conjugating enzyme to the target).
PTase	Protease	Protease (enzyme that catalyzes the breakdown of proteins into smaller fragments).
DNase	DNase	Deoxyribonuclease (DNase, for short); endonuclease that catalyzes the hydrolytic cleavage of the DNA backbone.

Table S2a: Key to Node Type Symbols

CAM	CAM	Cell adhesion proteins located on the cell surface.
CDK	CDK	Cyclin-dependent kinase.
CDKI	CDKI	Cyclin-dependent kinase inhibitor.
GEF	GEF	Guanine nucleotide exchange factor.
GAP	GAP	GTPase-activating protein (also called GTPase-accelerating protein).
GTPa	GTPase	GTPase enzymes that hydrolyze ATP to ADP.
Enz	Enzyme	Enzyme that does not fit the more specific enzyme categories listed above.
Prot	Protein	Regulatory protein that does not fit any of the more specific classifications listed above.
MP	Membrane_Potential	A relative measure of membrane potential across a biological membrane, generally indicating whether this potential is within the normal range, or abnormally low / high in a way that affects other regulatory processes.
lncRNA	LncRNA	Long intervening noncoding RNA
SLig	Cell_Surgace_Ligand	Membrane-bound signaling molecule that serves as a ligand to receptors on neighboring cells.

Table S2b: Key to Link Type Symbols

Symbol	Link Type	Description
Env	Enforced_Env	This link type represents self-loops on Environment nodes, which guarantee that these nodes maintain their initial state throughout a time-course simulation unless they are explicitly altered by the simulation's settings.
Ind	Indirect	Regulatory influence that does not involve direct binding, processing, or enzyme activity.
ComplProc	Complex_Process	Regulatory influence that is not modeled in detail, but involves more than one molecule or a macrostructure. For example, the physical need for kinetochores on replicated sister chromatids for the assembly of certain protein complexes can be represented as a link from the node representing kinetochores to the regulatory proteins, with a Complex_Process link type.
Per	Persistence	This link type represents self-loops that alter the ability of a node to stay in a particular state depending on its own current state. For example, if transcription of a protein is easier to maintain than to induce de novo, this may be encoded by a logic gate that includes the node itself and creates a self-loop. The link type of this loop is "Persistence".
TR	Transcription	Action of a transcription factor to alter the expression of the target node (mRNA or protein). Link type should be used for induction as well as repression (the link effect contains this information).

Table S2b: Key to Link Type Symbols

TL	Translation	Regulatory influence that controls the translation of mRNA into protein; should be used for induction as well as repression of translation.
Ligand	Ligand_Binding	Binding of extracellular ligand to its receptor.
Compl	Complex_Formation	Binding even that leads to a regulatory protein complex.
IBind	Inhibitory_Binding	Binding even that represses the target node's level or activity.
Loc	Localization	Regulatory influence that alters the localization of a molecule.
BLoc	Binding_Localization	Binding even that alters the localization of a molecule.
PBind	Protective_Binding	Binding even that increases / protects the target node's activity.
Unbind	Unbinding	A regulatory influence that causes the target node to be released from a protein complex and change its activity (increase or decrease) as a result.
P	Phosphorylation	Phosphorylation.
DP	Dephosphorylation	Dehosphorylation.
PLoc	Phosphorylation_Localization	Phosphorylation resulting in altered protein localization.
Ubiq	Ubiquitination	Ubiquitination, usually leading to protein degradation.
Deg	Degradation	Regulatory influence leading to the degradation of the target molecule (more general than Ubiquitination; the latter link type should be used when appropriate).
GEF	GEF_Activity	Action of a Guanine nucleotide exchange factor (GEF) leading to GTP loading onto (and usually the activation of) a GTPase.
GAP	GAP_Activity	Actions of a GTPase-activating protein (GAP) leading to the hydrolysis of GTP by (and usually de-activation of) a GTPase.
Lysis	Proteolysis	Protein cleavage.
Cat	Catalysis	Increasing the rate of metabolite production by an enzyme.
Epi	Epigenetic	Process that alters gene expression via modifying chromatin condensation or altering DNA methylation.
TrConf	Transcription_Conflict	Transcription from the same DNA locus using an alternate start site or proceeding in another direction, leading to a conflict between two transcriptional events.
Secr	Secretion	Secretion or shedding of a protein or other regulatory molecule to the extracellular environment.
RNAi	RNAi	This process represents inhibitory binding of cytoplasmic mRNAs by RISC-bound microRNAs that block translation and/or enhance mRNA degradation.
Acet	Acetylation	Acetylation
Deacet	Deacetylation	Deacetylation
-OH	Hydroxylation	Hydroxylation

Table S2c: Key to Link Effect Symbols

Table S2c: Key to Link Effect Symbols

Symbol	Link Effect	Description
$\leftarrow$	Activation	Link in which the input node aids the expression, activity, persistence or localization of the target such that the target is easier to turn/keep in an ON state. It can be used for multi-level nodes as long as these levels represent increasing intervals of activity.
$\vdash$	Repression	Link in which the input node hinders the expression, activity, persistence or localization of the target such that the target is easier to turn/keep in an OFF state. It can be used for multi-level nodes as long as these levels represent increasing intervals of activity.
$\bullet\!-$	Context_Dependent	Link acting on a node in such a way that it activates it under certain conditions (e.g., when another input is OFF), but represses it when this condition is not met. The XOR gate is a good example of a context-dependent link effect.
$\perp$	Inapt	This link-type refers to connections between complex regulatory switches (rather than molecules), where categorizing the effect of an input as Activation or Repression, or even context-depended activation or repression does not apply. This is usually the case with multi-state switches, where the 3 or more phenotypes represented by the discrete states of these switches do not have a meaningful ordering. Thus, stating that this switch is "activated" by another one is not appropriate.

References

[1] Sébastien Herzig and Reuben J. Shaw. AMPK: Guardian of metabolism and mitochondrial homeostasis. *Nature Reviews. Molecular Cell Biology*, 19(2):121–135, February 2018.

[2] Mark A Lemmon and Joseph Schlessinger. Cell signaling by receptor tyrosine kinases. *Cell*, 141(7):1117–1134, June 2010.

[3] Samrein B. M. Ahmed and Sally A. Prigent. Insights into the Shc Family of Adaptor Proteins. *Journal of Molecular Signaling*, 12:2, May 2017.

[4] A Uzman. Molecular Cell Biology (4th edition) Harvey Lodish, Arnold Berk, S. Lawrence Zipursky, Paul Matsudaira, David Baltimore and James Darnell; Freeman & Co., New York, NY, 2000, 1084 pp., list price 102.25, ISBN 0-7167-3136-3. *Biochemistry and Molecular Biology Education*, 29(3):126–128, 2001.

[5] F Chang, L S Steelman, J T Lee, J G Shelton, P M Navolanic, W L Blalock, R A Franklin, and J A McCubrey. Signal transduction mediated by the Ras/Raf/MEK/ERK pathway from cytokine receptors to transcription factors: Potential targeting for therapeutic intervention. *Leukemia*, 17(7):1263–1293, July 2003.

[6] C Widmann, S Gibson, and G L Johnson. Caspase-dependent cleavage of signaling proteins during apoptosis. A turn-off mechanism for anti-apoptotic signals. *J Biol Chem*, 273(12):7141–7147, March 1998.

[7] Yohannes A Mebratu, Burton F Dickey, Chris Evans, and Yohannes Tesfaigzi. The BH3-only protein Bik/Blk/Nbk inhibits nuclear translocation of activated ERK1/2 to mediate IFNgamma-induced cell death. *J Cell Biol*, 183(3):429–439, November 2008.

[8] Pengda Liu, Wenjian Gan, Y Rebecca Chin, Kohei Ogura, Jianping Guo, Jinfang Zhang, Bin Wang, John Blenis, Lewis C Cantley, Alex Toker, Bing Su, and Wenyi Wei. PtdIns(3,4,5)P3-Dependent Activation of the mTORC2 Kinase Complex. *Cancer discovery*, 5(11):1194–1209, November 2015.

[9] Hui H Zhang, Alex I Lipovsky, Christian C Dibble, Mustafa Sahin, and Brendan D Manning. S6K1 regulates GSK3 under conditions of mTOR-dependent feedback inhibition of Akt. *Mol Cell*, 24(2):185–197, October 2006.

[10] Christian C Dibble, John M Asara, and Brendan D Manning. Characterization of Rictor phosphorylation sites reveals direct regulation of mTOR complex 2 by S6K1. *Molecular and cellular biology*, 29(21):5657–5670, November 2009.

[11] Zhi-Xiang Xu, Ji-Wei Tan, Haifei Xu, Cassandra J. Hill, Olga Ostrovskaya, Kirill A. Martemyanov, and Baoji Xu. Caspase-2 promotes AMPA receptor internalization and cognitive flexibility via mTORC2-AKT-GSK3β signaling. *Nature Communications*, 10(1):3622, August 2019.

[12] P Rodriguez-Viciana, P H Warne, R Dhand, B Vanhaesebroeck, I Gout, M J Fry, M D Waterfield, and J Downward. Phosphatidylinositol-3-OH kinase as a direct target of Ras. *Nature*, 370(6490):527–532, August 1994.

[13] Surbhi Gupta, Antoine R Ramjaun, Paula Haiko, Yihua Wang, Patricia H Warne, Barbara Nicke, Emma Nye, Gordon Stamp, Kari Alitalo, and Julian Downward. Binding of Ras to phosphoinositide 3-kinase p110alpha is required for Ras-driven tumorigenesis in mice. *Cell*, 129(5):957–968, June 2007.

[14] Brendan D Manning and Alex Toker. AKT/PKB Signaling: Navigating the Network. *Cell*, 169(3):381–405, April 2017.

[15] Rosaline C-Y Hui, Ana R Gomes, Demetra Constantinidou, Joana R Costa, Christina T Karadedou, Silvia Fernández de Mattos, Matthias P Wymann, Jan J Brosens, Almut Schulze, and Eric W-F Lam. The forkhead transcription factor FOXO3a increases phosphoinositide-3 kinase/Akt activity in drug-resistant leukemic cells through induction of PIK3CA expression. *Molecular and cellular biology*, 28(19):5886–5898, October 2008.

[16] Zixi Wang, Tingting Dang, Tingting Liu, She Chen, Lin Li, Song Huang, and Min Fang. NEDD4L Protein Catalyzes Ubiquitination of PIK3CA Protein and Regulates PI3K-AKT Signaling. *Journal of Biological Chemistry*, 291(33):17467–17477, August 2016.

[17] Tina L Yuan, Gerburg Wulf, Laura Burga, and Lewis C Cantley. Cell-to-Cell Variability in PI3K Protein Level Regulates PI3K-AKT Pathway Activity in Cell Populations. *Current biology : CB*, 21(3):173–183, February 2011.

[18] Eric L. Greer, Philip R. Oskoui, Max R. Banko, Jay M. Maniar, Melanie P. Gygi, Steven P. Gygi, and Anne Brunet. The energy sensor AMP-activated protein kinase directly regulates the mammalian FOXO3 transcription factor. *The Journal of Biological Chemistry*, 282(41):30107–30119, October 2007.

[19] Xianwang Wang, Shujuan Hu, and Lei Liu. Phosphorylation and acetylation modifications of FOXO3a: Independently or synergistically? *Oncology Letters*, 13(5):2867–2872, May 2017.

[20] Anne H. H. Tseng, Shyan-Shu Shieh, and Danny Ling Wang. SIRT3 deacetylates FOXO3 to protect mitochondria against oxidative damage. *Free Radical Biology & Medicine*, 63:222–234, October 2013.

[21] Octavian Bucur, Andreea Lucia Stancu, Maria Sinziana Muraru, Armelle Melet, Stefana Maria Petrescu, and Roya Khosravi-Far. PLK1 is a binding partner and a negative regulator of FOXO3 tumor suppressor. *Discoveries (Craiova, Romania)*, 2(2):e16, April 2014.

[22] Wei Fu, Qiuping Ma, Lei Chen, Pengfei Li, Mu Zhang, Sivapriya Ramamoorthy, Zafar Nawaz, Tsukasa Shimojima, Hengbin Wang, Yonghua Yang, Zheng Shen, Yingtao Zhang, Xiaohong Zhang, Santo V. Nicosia, Yanping Zhang, Jack W. Pledger, Jiandong Chen, and Wenlong Bai. MDM2 acts downstream of p53 as an E3 ligase to promote FOXO ubiquitination and degradation. *The Journal of Biological Chemistry*, 284(21):13987–14000, May 2009.

[23] Jer-Yen Yang, Cong S Zong, Weiya Xia, Hirohito Yamaguchi, Qingqing Ding, Xiaoming Xie, Jing-Yu Lang, Chien-Chen Lai, Chun-Ju Chang, Wei-Chien Huang, Hsin Huang, Hsu-Ping Kuo, Dung-Fang Lee, Long-Yuan Li, Huang-Chun Lien, Xiaoyun Cheng, King-Jen Chang, Chwan-Deng Hsiao, Fuu-Jen Tsai, Chang-Hai Tsai, Aysegul A Sahin, William J Muller, Gordon B Mills, Dihua Yu, Gabriel N Hortobagyi, and Mien-Chie Hung. ERK promotes tumorigenesis by inhibiting FOXO3a via MDM2-mediated degradation. *Nat Cell Biol*, 10(2):138–148, February 2008.

[24] Yasuo Miyaguchi, Keita Tsuchiya, and Kazuichi Sakamoto. P53 negatively regulates the transcriptional activity of FOXO3a under oxidative stress. *Cell Biology International*, 33(8):853–860, August 2009.

[25] S Cockcroft and G M Thomas. Inositol-lipid-specific phospholipase C isoenzymes and their differential regulation by receptors. *The Biochemical journal*, 288 (Pt 1)(Pt 1):1–14, November 1992.

[26] H K Kim, J W Kim, A Zilberstein, B Margolis, J G Kim, J Schlessinger, and S G Rhee. PDGF stimulation of inositol phospholipid hydrolysis requires PLC-gamma 1 phosphorylation on tyrosine residues 783 and 1254. *Cell*, 65(3):435–441, May 1991.

[27] W Zhang, R P Trible, M Zhu, S K Liu, C J McGlade, and L E Samelson. Association of Grb2, Gads, and phospholipase C-gamma 1 with phosphorylated LAT tyrosine residues. Effect of LAT tyrosine mutations on T cell angigen receptor-mediated signaling. *J Biol Chem*, 275(30):23355–23361, July 2000.

[28] M Falasca, S K Logan, V P Lehto, G Baccante, M A Lemmon, and J Schlessinger. Activation of phospholipase C gamma by PI 3-kinase-induced PH domain-mediated membrane targeting. *EMBO J*, 17(2):414–422, January 1998.

[29] L E Rameh, S G Rhee, K Spokes, A Kazlauskas, L C Cantley, and L G Cantley. Phosphoinositide 3-kinase regulates phospholipase Cgamma-mediated calcium signaling. *J Biol Chem*, 273(37):23750–23757, September 1998.

[30] Aurelie Gresset, John Sondek, and T Kendall Harden. The phospholipase C isozymes and their regulation. *Sub-cellular biochemistry*, 58(Chapter 3):61–94, 2012.

[31] R H Michell, C J Kirk, L M Jones, C P Downes, and J A Creba. The stimulation of inositol lipid metabolism that accompanies calcium mobilization in stimulated cells: Defined characteristics and unanswered questions. *Philosophical transactions of the Royal Society of London Series B, Biological sciences*, 296(1080):123–138, December 1981.

[32] Albert Escobedo, Tiago Gomes, Eric Aragón, Pau Martín-Malpartida, Lidia Ruiz, and Maria J Macias. Structural basis of the activation and degradation mechanisms of the E3 ubiquitin ligase Nedd4L. *Structure (London, England : 1993)*, 22(10):1446–1457, October 2014.

[33] Chengfu Yuan, Lei Wang, Liang Zhou, and Zheng Fu. The function of FOXO1 in the late phases of the cell cycle is suppressed by PLK1-mediated phosphorylation. *Cell Cycle*, 13(5):807–819, 2014.

[34] Maria Saline, Lukas Badertscher, Madita Wolter, Roxanne Lau, Anders Gunnarsson, Tomas Jacso, Tyrrell Norris, Christian Ottmann, and Arjan Snijder. AMPK and AKT protein kinases hierarchically phosphorylate the N-terminus of the FOXO1 transcription factor, modulating interactions with 14-3-3 proteins. *The Journal of Biological Chemistry*, 294(35):13106–13116, August 2019.

[35] Bin Zhang, Shaoyuan Cui, Xueyuan Bai, Li Zhuo, Xuefeng Sun, Quan Hong, Bo Fu, Jianzhong Wang, Xiangmei Chen, and Guangyan Cai. SIRT3 overexpression antagonizes high glucose accelerated cellular senescence in human diploid fibroblasts via the SIRT3-FOXO1 signaling pathway. *Age (Dordrecht, Netherlands)*, 35(6):2237–2253, December 2013.

[36] Geert J. P. L. Kops, Tobias B. Dansen, Paulien E. Polderman, Ingrid Saarloos, Karel W. A. Wirtz, Paul J. Coffer, Ting-T. Huang, Johannes L. Bos, René H. Medema, and Boudewijn M. T. Burgering. Forkhead transcription factor FOXO3a protects quiescent cells from oxidative stress. *Nature*, 419(6904):316–321, September 2002.

[37] Dan Shao, Peiyong Zhai, Dominic P. Del Re, Sebastiano Sciarretta, Norikazu Yabuta, Hiroshi Nojima, Dae-Sik Lim, Duojia Pan, and Junichi Sadoshima. A functional interaction between Hippo-YAP signalling and FoxO1 mediates the oxidative stress response. *Nature Communications*, 5:3315, 2014.

[38] Yoshinori Imai, Akiko Takahashi, Aki Hanyu, Satoshi Hori, Seidai Sato, Kazuhito Naka, Atsushi Hirao, Naoko Ohtani, and Eiji Hara. Crosstalk between the Rb pathway and AKT signaling forms a quiescence-senescence switch. *Cell Reports*, 7(1):194–207, April 2014.

[39] Dongyun Zhang, Yulei Wang, Yuguang Liang, Min Zhang, Jinlong Wei, Xiao Zheng, Fei Li, Yan Meng, Nina Wu Zhu, Jingxia Li, Xue-Ru Wu, and Chuanshu Huang. Loss of p27 upregulates MnSOD in a STAT3-dependent manner, disrupts intracellular redox activity and enhances cell migration. *Journal of Cell Science*, 127(Pt 13):2920–2933, July 2014.

[40] Jingxin Liu, Dan Li, Tian Zhang, Qiang Tong, Richard Dequan Ye, and Ligen Lin. SIRT3 protects hepatocytes from oxidative injury by enhancing ROS scavenging and mitochondrial integrity. *Cell Death & Disease*, 8(10):e3158, October 2017.

[41] Joan Seoane, Hong-Van Le, Lijian Shen, Stewart A. Anderson, and Joan Massagué. Integration of Smad and forkhead pathways in the control of neuroepithelial and glioblastoma cell proliferation. *Cell*, 117(2):211–223, April 2004.

[42] P. Staller, K. Peukert, A. Kiermaier, J. Seoane, J. Lukas, H. Karsunky, T. Möröy, J. Bartek, J. Massagué, F. Hänel, and M. Eilers. Repression of p15INK4b expression by Myc through association with Miz-1. *Nature Cell Biology*, 3(4):392–399, April 2001.

[43] Joan Seoane, Hong-Van Le, and Joan Massagué. Myc suppression of the p21(Cip1) Cdk inhibitor influences the outcome of the p53 response to DNA damage. *Nature*, 419(6908):729–734, October 2002.

[44] W. S. el-Deiry, T. Tokino, V. E. Velculescu, D. B. Levy, R. Parsons, J. M. Trent, D. Lin, W. E. Mercer, K. W. Kinzler, and B. Vogelstein. WAF1, a potential mediator of p53 tumor suppression. *Cell*, 75(4):817–825, November 1993.

[45] Ken Inoki, Tianqing Zhu, and Kun-Liang Guan. TSC2 mediates cellular energy response to control cell growth and survival. *Cell*, 115(5):577–590, November 2003.

[46] Alberto Bartolomé, Ana García-Aguilar, Shun-Ichiro Asahara, Yoshiaki Kido, Carlos Guillén, Utpal B. Pajvani, and Manuel Benito. MTORC1 Regulates both General Autophagy and Mitophagy Induction after Oxidative Phosphorylation Uncoupling. *Molecular and Cellular Biology*, 37(23):e00441–17, December 2017.

[47] Robert A. Saxton and David M. Sabatini. mTOR Signaling in Growth, Metabolism, and Disease. *Cell*, 169(2):361–371, April 2017.

[48] Ken Inoki, Yong Li, Tianquan Zhu, Jun Wu, and Kun-Liang Guan. TSC2 is phosphorylated and inhibited by Akt and suppresses mTOR signalling. *Nature Cell Biology*, 4(9):648–657, September 2002.

[49] Li Ma, Zhenbang Chen, Hediye Erdjument-Bromage, Paul Tempst, and Pier Paolo Pandolfi. Phosphorylation and functional inactivation of TSC2 by Erk implications for tuberous sclerosis and cancer pathogenesis. *Cell*, 121(2):179–193, April 2005.

[50] Philippe P. Roux, Bryan A. Ballif, Rana Anjum, Steven P. Gygi, and John Blenis. Tumor-promoting phorbol esters and activated Ras inactivate the tuberous sclerosis tumor suppressor complex via p90 ribosomal S6 kinase. *Proceedings of the National Academy of Sciences of the United States of America*, 101(37):13489–13494, September 2004.

[51] Claudia Wiza, Emmani B. M. Nascimento, and D. Margriet Ouwens. Role of PRAS40 in Akt and mTOR signaling in health and disease. *American Journal of Physiology. Endocrinology and Metabolism*, 302(12):E1453–1460, June 2012.

[52] Emilie Vander Haar, Seong-Il Lee, Sricharan Bandhakavi, Timothy J. Griffin, and Do-Hyung Kim. Insulin signalling to mTOR mediated by the Akt/PKB substrate PRAS40. *Nature Cell Biology*, 9(3):316–323, March 2007.

[53] Bruno D. Fonseca, Ewan M. Smith, Vivian H.-Y. Lee, Carol MacKintosh, and Christopher G. Proud. PRAS40 is a target for mammalian target of rapamycin complex 1 and is required for signaling downstream of this complex. *The Journal of Biological Chemistry*, 282(34):24514–24524, August 2007.

[54] Ken Inoki, Yong Li, Tian Xu, and Kun-Liang Guan. Rheb GTPase is a direct target of TSC2 GAP activity and regulates mTOR signaling. *Genes & Development*, 17(15):1829–1834, August 2003.

[55] Mengling Liu, Christopher J. Clarke, Mohamed F. Salama, Yeon Ja Choi, Lina M. Obeid, and Yusuf A. Hannun. Co-ordinated activation of classical and novel PKC isoforms is required for PMA-induced mTORC1 activation. *PloS One*, 12(9):e0184818, 2017.

[56] Constantinos Demetriades, Monika Plescher, and Aurelio A. Teleman. Lysosomal recruitment of TSC2 is a universal response to cellular stress. *Nature Communications*, 7:10662, February 2016.

[57] Sebastian Real, Nathalie Meo-Evoli, Lilia Espada, and Albert Tauler. E2F1 regulates cellular growth by mTORC1 signaling. *PloS One*, 6(1):e16163, January 2011.

[58] Dana M. Gwinn, David B. Shackelford, Daniel F. Egan, Maria M. Mihaylova, Annabelle Mery, Debbie S. Vasquez, Benjamin E. Turk, and Reuben J. Shaw. AMPK Phosphorylation of Raptor Mediates a Metabolic Checkpoint. *Molecular Cell*, 30(2):214–226, April 2008.

[59] Xiaomeng Long, Yenshou Lin, Sara Ortiz-Vega, Kazuyoshi Yonezawa, and Joseph Avruch. Rheb binds and regulates the mTOR kinase. *Current biology: CB*, 15(8):702–713, April 2005.

[60] Audrey Carriere, Yves Romeo, Hugo A. Acosta-Jaquez, Julie Moreau, Eric Bonneil, Pierre Thibault, Diane C. Fingar, and Philippe P. Roux. ERK1/2 phosphorylate Raptor to promote Ras-dependent activation of mTOR complex 1 (mTORC1). *The Journal of Biological Chemistry*, 286(1):567–577, January 2011.

[61] Francisco Ramírez-Valle, Michelle L. Badura, Steve Braunstein, Manisha Narasimhan, and Robert J. Schneider. Mitotic raptor promotes mTORC1 activity, G(2)/M cell cycle progression, and internal ribosome entry site-mediated mRNA translation. *Molecular and Cellular Biology*, 30(13):3151–3164, July 2010.

[62] R. Martin, C. Desponds, R. O. Eren, M. Quadroni, M. Thome, and N. Fasel. Caspase-mediated cleavage of raptor participates in the inactivation of mTORC1 during cell death. *Cell Death Discovery*, 2:16024, 2016.

[63] Xiaoju Max Ma and John Blenis. Molecular mechanisms of mTOR-mediated translational control. *Nature Reviews. Molecular Cell Biology*, 10(5):307–318, May 2009.

[64] P. J. Papst, H. Sugiyama, M. Nagasawa, J. J. Lucas, J. L. Maller, and N. Terada. Cdc2-cyclin B phosphorylates p70 S6 kinase on Ser411 at mitosis. *The Journal of Biological Chemistry*, 273(24):15077–15084, June 1998.

[65] Rohini Dhar, Shalini D. Persaud, Joe R. Mireles, and Alakananda Basu. Proteolytic cleavage of p70 ribosomal S6 kinase by caspase-3 during DNA damage-induced apoptosis. *Biochemistry*, 48(7):1474–1480, February 2009.

[66] M. Bushell, L. McKendrick, R. U. Jänicke, M. J. Clemens, and S. J. Morley. Caspase-3 is necessary and sufficient for cleavage of protein synthesis eukaryotic initiation factor 4G during apoptosis. *FEBS letters*, 451(3):332–336, May 1999.

[67] James G. Wakefield, David J. Stephens, and Jeremy M. Tavaré. A role for glycogen synthase kinase-3 in mitotic spindle dynamics and chromosome alignment. *Journal of Cell Science*, 116(4):637–646, February 2003.

[68] Nagalingam R. Sundaresan, Samik Bindu, Vinodkumar B. Pillai, Sadhana Samant, Yong Pan, Jing-Yi Huang, Madhu Gupta, Raghu S. Nagalingam, Donald Wolfgeher, Eric Verdin, and Mahesh P. Gupta. SIRT3 Blocks Aging-Associated Tissue Fibrosis in Mice by Deacetylating and Activating Glycogen Synthase Kinase 3β. *Molecular and Cellular Biology*, 36(5):678–692, March 2016.

[69] Qingqing Ding, Weiya Xia, Jaw-Ching Liu, Jer-Yen Yang, Dung-Fang Lee, Jiahong Xia, Geoffrey Bartholomeusz, Yan Li, Yong Pan, Zheng Li, Ralf C. Bargou, Jun Qin, Chien-Chen Lai, Fuu-Jen Tsai, Chang-Hai Tsai, and Mien-Chie Hung. Erk associates with and primes GSK-3beta for its inactivation resulting in upregulation of beta-catenin. *Molecular Cell*, 19(2):159–170, July 2005.

[70] Nirmala Hariharan, Yasuhiro Maejima, Jun Nakae, Jihye Paik, Ronald A. Depinho, and Junichi Sadoshima. Deacetylation of FoxO by Sirt1 Plays an Essential Role in Mediating Starvation-Induced Autophagy in Cardiac Myocytes. *Circulation Research*, 107(12):1470–1482, December 2010.

[71] Ying Zhao, Jing Yang, Wenjuan Liao, Xiangyu Liu, Hui Zhang, Shan Wang, Donglai Wang, Jingnan Feng, Li Yu, and Wei-Guo Zhu. Cytosolic FoxO1 is essential for the induction of autophagy and tumour suppressor activity. *Nature Cell Biology*, 12(7):665–675, July 2010.

[72] Joungmok Kim, Mondira Kundu, Benoit Viollet, and Kun-Liang Guan. AMPK and mTOR regulate autophagy through direct phosphorylation of Ulk1. *Nature Cell Biology*, 13(2):132–141, February 2011.

[73] Shouqing Luo, Moises Garcia-Arencibia, Rui Zhao, Claudia Puri, Pearl P. C. Toh, Oana Sadiq, and David C. Rubinsztein. Bim inhibits autophagy by recruiting Beclin 1 to microtubules. *Molecular Cell*, 47(3):359–370, August 2012.

[74] G. Filomeni, D. De Zio, and F. Cecconi. Oxidative stress and autophagy: The clash between damage and metabolic needs. *Cell Death and Differentiation*, 22(3):377–388, March 2015.

[75] Simon Mitchell, Jesse Vargas, and Alexander Hoffmann. Signaling via the NFκB system. *Wiley Interdisciplinary Reviews. Systems Biology and Medicine*, 8(3):227–241, May 2016.

[76] Dong Bai, Lynn Ueno, and Peter K. Vogt. Akt-mediated regulation of NFκB and the essentialness of NFκB for the oncogenicity of PI3K and Akt. *International journal of cancer. Journal international du cancer*, 125(12):2863–2870, December 2009.

[77] Matthew G. Thompson, Michelle Larson, Amy Vidrine, Kelly Barrios, Flor Navarro, Kaitlyn Meyers, Patricia Simms, Kushal Prajapati, Lennox Chitsike, Lance M. Hellman, Brian M. Baker, and Stephanie K. Watkins. FOXO3-NF-κB RelA Protein Complexes Reduce Proinflammatory Cell Signaling and Function. *Journal of Immunology (Baltimore, Md.: 1950)*, 195(12):5637–5647, December 2015.

[78] Gill A. Webster and Neil D. Perkins. Transcriptional Cross Talk between NF-κB and p53. *Molecular and Cellular Biology*, 19(5):3485–3495, May 1999.

[79] A. Israel. The IKK Complex, a Central Regulator of NF- B Activation. *Cold Spring Harbor Perspectives in Biology*, 2(3):a000158–a000158, March 2010.

[80] Jeremy Berg, John L Tymoczko, and Lubert Stryer. *Biochemistry*. Macmillan, 2007.

[81] Jeffrey C. Rathmell, Casey J. Fox, David R. Plas, Peter S. Hammerman, Ryan M. Cinalli, and Craig B. Thompson. Akt-Directed Glucose Metabolism Can Prevent Bax Conformation Change and Promote Growth Factor-Independent Survival. *Molecular and Cellular Biology*, 23(20):7315–7328, October 2003.

[82] D. J. Roberts and S. Miyamoto. Hexokinase II integrates energy metabolism and cellular protection: Akting on mitochondria and TORCing to autophagy. *Cell Death and Differentiation*, 22(2):248–257, February 2015.

[83] Bo Tan, Sucai Dong, Robert L. Shepard, Lisa Kays, Kenneth D. Roth, Sandaruwan Geeganage, Ming-Shang Kuo, and Genshi Zhao. Inhibition of Nicotinamide Phosphoribosyltransferase (NAMPT), an Enzyme Essential for NAD+ Biosynthesis, Leads to Altered Carbohydrate Metabolism in Cancer Cells. *The Journal of Biological Chemistry*, 290(25):15812–15824, June 2015.

[84] S. J. Yeung, J. Pan, and M.-H. Lee. Roles of p53, MYC and HIF-1 in regulating glycolysis - the seventh hallmark of cancer. *Cellular and molecular life sciences: CMLS*, 65(24):3981–3999, December 2008.

[85] Salvatore Papa, Pui Man Choy, and Concetta Bubici. The ERK and JNK pathways in the regulation of metabolic reprogramming. *Oncogene*, 38(13):2223–2240, March 2019.

[86] Tobias Schmid, Jie Zhou, Roman Köhl, and Bernhard Brüne. P300 relieves p53-evoked transcriptional repression of hypoxia-inducible factor-1 (HIF-1). *The Biochemical Journal*, 380(Pt 1):289–295, May 2004.

[87] Sarika Srivastava. Emerging therapeutic roles for NAD(+) metabolism in mitochondrial and age-related disorders. *Clinical and Translational Medicine*, 5(1):25, December 2016.

[88] Piet Borst. The malate-aspartate shuttle (Borst cycle): How it started and developed into a major metabolic pathway. *IUBMB life*, 72(11):2241–2259, November 2020.

[89] Bruce Alberts, Alexander Johnson, Julian Lewis, David Morgan, Martin Raff, Keith Roberts, and Peter Walter. *Molecular Biology of the Cell*. W.W. Norton & Company, 6 edition, August 2017.

[90] Alba Luengo, Zhaoqi Li, Dan Y. Gui, Lucas B. Sullivan, Maria Zagorulya, Brian T. Do, Raphael Ferreira, Adi Naamati, Ahmed Ali, Caroline A. Lewis, Craig J. Thomas, Stefani Spranger, Nicholas J. Matheson, and Matthew G. Vander Heiden. Increased demand for NAD+ relative to ATP drives aerobic glycolysis. *Molecular Cell*, 81(4):691–707.e6, February 2021.

[91] Andrew P. Halestrap and Nigel T. Price. The proton-linked monocarboxylate transporter (MCT) family: Structure, function and regulation. *Biochemical Journal*, 343(2):281–299, October 1999.

[92] K. M. Dodd, J. Yang, M. H. Shen, J. R. Sampson, and A. R. Tee. mTORC1 drives HIF-1α and VEGF-A signalling via multiple mechanisms involving 4E-BP1, S6K1 and STAT3. *Oncogene*, 34(17):2239–2250, April 2015.

[93] Kreon Koukoulas, Antonis Giakountis, Angeliki Karagiota, Martina Samiotaki, George Panayotou, George Simos, and Ilias Mylonis. ERK signaling controls productive HIF-1 binding to chromatin and cancer cell adaptation to hypoxia through HIF-1α interaction with NPM1. *Molecular Oncology*, 15(12):3468–3489, December 2021.

[94] Daniela Flügel, Agnes Görlach, Carine Michiels, and Thomas Kietzmann. Glycogen synthase kinase 3 phosphorylates hypoxia-inducible factor 1alpha and mediates its destabilization in a VHL-independent manner. *Molecular and Cellular Biology*, 27(9):3253–3265, May 2007.

[95] Brooke M. Emerling, Frank Weinberg, Juinn-Lin Liu, Tak W. Mak, and Navdeep S. Chandel. PTEN regulates p300-dependent hypoxia-inducible factor 1 transcriptional activity through Forkhead transcription factor 3a (FOXO3a). *Proceedings of the National Academy of Sciences of the United States of America*, 105(7):2622–2627, February 2008.

[96] E. Laughner, P. Taghavi, K. Chiles, P. C. Mahon, and G. L. Semenza. HER2 (neu) signaling increases the rate of hypoxia-inducible factor 1alpha (HIF-1alpha) synthesis: Novel mechanism for HIF-1-mediated vascular endothelial growth factor expression. *Molecular and Cellular Biology*, 21(12):3995–4004, June 2001.

[97] Weiwei Yang and Zhimin Lu. Nuclear PKM2 regulates the Warburg effect. *Cell Cycle (Georgetown, Tex.)*, 12(19):3154–3158, October 2013.

[98] Sara Pich, Daniel Bach, Paz Briones, Marc Liesa, Marta Camps, Xavier Testar, Manuel Palacín, and Antonio Zorzano. The Charcot-Marie-Tooth type 2A gene product, Mfn2, up-regulates fuel oxidation through expression of OXPHOS system. *Human Molecular Genetics*, 14(11):1405–1415, June 2005.

[99] Eunyong Ahn, Praveen Kumar, Dzmitry Mukha, Amit Tzur, and Tomer Shlomi. Temporal fluxomics reveals oscillations in TCA cycle flux throughout the mammalian cell cycle. *Molecular Systems Biology*, 13(11):953, November 2017.

[100] Martin Picard, Orian S. Shirihai, Benoit J. Gentil, and Yan Burelle. Mitochondrial morphology transitions and functions: Implications for retrograde signaling? *American Journal of Physiology-Regulatory, Integrative and Comparative Physiology*, 304(6):R393–R406, March 2013.

[101] Vikas Bhardwaj and Jun He. Reactive Oxygen Species, Metabolic Plasticity, and Drug Resistance in Cancer. *International Journal of Molecular Sciences*, 21(10):3412, May 2020.

[102] Amy C. Nulton-Persson and Luke I. Szweda. Modulation of Mitochondrial Function by Hydrogen Peroxide. *Journal of Biological Chemistry*, 276(26):23357–23361, June 2001.

[103] David Cantu, Jerome Schaack, and Manisha Patel. Oxidative inactivation of mitochondrial aconitase results in iron and H2O2-mediated neurotoxicity in rat primary mesencephalic cultures. *PloS One*, 4(9):e7095, September 2009.

[104] Milana A. B. Applegate, Kenneth M. Humphries, and Luke I. Szweda. Reversible inhibition of alpha-ketoglutarate dehydrogenase by hydrogen peroxide: Glutathionylation and protection of lipoic acid. *Biochemistry*, 47(1):473–478, January 2008.

[105] Song Gao and Junjie Hu. Mitochondrial Fusion: The Machineries In and Out. *Trends in Cell Biology*, 31(1):62–74, January 2021.

[106] Matthew E. Gegg, J. Mark Cooper, Kai-Yin Chau, Manuel Rojo, Anthony H. V. Schapira, and Jan-Willem Taanman. Mitofusin 1 and mitofusin 2 are ubiquitinated in a PINK1/parkin-dependent manner upon induction of mitophagy. *Human Molecular Genetics*, 19(24):4861–4870, December 2010.

[107] Seok Min Jin and Richard J. Youle. PINK1- and Parkin-mediated mitophagy at a glance. *Journal of Cell Science*, 125(Pt 4):795–799, February 2012.

[108] Rong Yu, Shao-Bo Jin, Urban Lendahl, Monica Nistér, and Jian Zhao. Human Fis1 regulates mitochondrial dynamics through inhibition of the fusion machinery. *The EMBO Journal*, 38(8), April 2019.

[109] Ola J. Martin, Ling Lai, Mangala M. Soundarapandian, Teresa C. Leone, Antonio Zorzano, Mark P. Keller, Alan D. Attie, Deborah M. Muoio, and Daniel P. Kelly. A role for peroxisome proliferator-activated receptor γ coactivator-1 in the control of mitochondrial dynamics during postnatal cardiac growth. *Circulation Research*, 114(4):626–636, February 2014.

[110] Romain Cartoni, Bertrand Léger, M. Benjamin Hock, Manu Praz, Antoinette Crettenand, Sara Pich, Jean-Luc Ziltener, François Luthi, Olivier Dériaz, Antonio Zorzano, Charles Gobelet, Anastasia Kralli, and Aaron P. Russell. Mitofusins 1/2 and ERRalpha expression are increased in human skeletal muscle after physical exercise. *The Journal of Physiology*, 567(Pt 1):349–358, August 2005.

[111] Marc Liesa, Bárbara Borda-d'Agua, Gema Medina-Gómez, Christopher J. Lelliott, José Carlos Paz, Manuel Rojo, Manuel Palacín, Antonio Vidal-Puig, and Antonio Zorzano. Mitochondrial fusion is increased by the nuclear coactivator PGC-1beta. *PloS One*, 3(10):e3613, 2008.

[112] Sudha Bucha, Debashis Mukhopadhyay, and Nitai Pada Bhattacharyya. E2F1 activates MFN2 expression by binding to the promoter and decreases mitochondrial fission and mitophagy in HeLa cells. *The FEBS journal*, 286(22):4525–4541, November 2019.

[113] Zhiyin Song, Mariam Ghochani, J. Michael McCaffery, Terrence G. Frey, and David C. Chan. Mitofusins and OPA1 mediate sequential steps in mitochondrial membrane fusion. *Molecular Biology of the Cell*, 20(15):3525–3532, August 2009.

[114] Benedikt Westermann. Bioenergetic role of mitochondrial fusion and fission. *Biochimica Et Biophysica Acta*, 1817(10):1833–1838, October 2012.

[115] Erik E. Griffin, Scott A. Detmer, and David C. Chan. Molecular mechanism of mitochondrial membrane fusion. *Biochimica et Biophysica Acta (BBA) - Molecular Cell Research*, 1763(5-6):482–489, May 2006.

[116] Chenxia Hu, Yong Huang, and Lanjuan Li. Drp1-Dependent Mitochondrial Fission Plays Critical Roles in Physiological and Pathological Progresses in Mammals. *International Journal of Molecular Sciences*, 18(1):144, January 2017.

[117] Patrick J. Macdonald, Natalia Stepanyants, Niharika Mehrotra, Jason A. Mears, Xin Qi, Hiromi Sesaki, and Rajesh Ramachandran. A dimeric equilibrium intermediate nucleates Drp1 reassembly on mitochondrial membranes for fission. *Molecular Biology of the Cell*, 25(12):1905–1915, June 2014.

[118] Prashant Mishra, Valerio Carelli, Giovanni Manfredi, and David C. Chan. Proteolytic cleavage of Opa1 stimulates mitochondrial inner membrane fusion and couples fusion to oxidative phosphorylation. *Cell Metabolism*, 19(4):630–641, April 2014.

[119] Cong-Hui Yao, Rencheng Wang, Yahui Wang, Che-Pei Kung, Jason D. Weber, and Gary J. Patti. Mitochondrial fusion supports increased oxidative phosphorylation during cell proliferation. *eLife*, 8:e41351, January 2019.

[120] Kasturi Mitra, Christian Wunder, Badrinath Roysam, Gang Lin, and Jennifer Lippincott-Schwartz. A hyperfused mitochondrial state achieved at G1-S regulates cyclin E buildup and entry into S phase. *Proceedings of the National Academy of Sciences of the United States of America*, 106(29):11960–11965, July 2009.

[121] Dmitry B. Zorov, Magdalena Juhaszova, and Steven J. Sollott. Mitochondrial ROS-induced ROS release: An update and review. *Biochimica Et Biophysica Acta*, 1757(5-6):509–517, 2006.

[122] Hongce Chen, Beini Sun, Han Sun, Lingjun Xu, Guihao Wu, Zhuang Tu, Xuecheng Cheng, Xuhong Fan, Zihao Mai, Qiling Tang, Xiaoping Wang, and Tongsheng Chen. Bak instead of Bax plays a key role in metformin-induced apoptosis s in HCT116 cells. *Cell Death Discovery*, 7(1):363, November 2021.

[123] J. Xiang, D. T. Chao, and S. J. Korsmeyer. BAX-induced cell death may not require interleukin 1 beta-converting enzyme-like proteases. *Proceedings of the National Academy of Sciences of the United States of America*, 93(25):14559–14563, December 1996.

[124] Hsiuchen Chen, Anne Chomyn, and David C. Chan. Disruption of fusion results in mitochondrial heterogeneity and dysfunction. *The Journal of Biological Chemistry*, 280(28):26185–26192, July 2005.

[125] Wen-Xing Ding and Xiao-Ming Yin. Mitophagy: Mechanisms, pathophysiological roles, and analysis. *Biological Chemistry*, 393(7):547–564, July 2012.

[126] J. E. Kokoszka, P. Coskun, L. A. Esposito, and D. C. Wallace. Increased mitochondrial oxidative stress in the Sod2 (+/-) mouse results in the age-related decline of mitochondrial function culminating in increased apoptosis. *Proceedings of the National Academy of Sciences of the United States of America*, 98(5):2278–2283, February 2001.

[127] Bong-Hyun Ahn, Hyun-Seok Kim, Shiwei Song, In Hye Lee, Jie Liu, Athanassios Vassilopoulos, Chu-Xia Deng, and Toren Finkel. A role for the mitochondrial deacetylase Sirt3 in regulating energy homeostasis. *Proceedings of the National Academy of Sciences of the United States of America*, 105(38):14447–14452, September 2008.

[128] Lei Chen, Yuan Qin, Bilin Liu, Meng Gao, Anqi Li, Xue Li, and Guohua Gong. PGC-1α-Mediated Mitochondrial Quality Control: Molecular Mechanisms and Implications for Heart Failure. *Frontiers in Cell and Developmental Biology*, 10:871357, 2022.

[129] Y. Li, T. T. Huang, E. J. Carlson, S. Melov, P. C. Ursell, J. L. Olson, L. J. Noble, M. P. Yoshimura, C. Berger, P. H. Chan, D. C. Wallace, and C. J. Epstein. Dilated cardiomyopathy and neonatal lethality in mutant mice lacking manganese superoxide dismutase. *Nature Genetics*, 11(4):376–381, December 1995.

[130] Y. Eguchi, S. Shimizu, and Y. Tsujimoto. Intracellular ATP levels determine cell death fate by apoptosis or necrosis. *Cancer Research*, 57(10):1835–1840, May 1997.

[131] Julian J. Lum, Ralph J. DeBerardinis, and Craig B. Thompson. Autophagy in metazoans: Cell survival in the land of plenty. *Nature Reviews. Molecular Cell Biology*, 6(6):439–448, June 2005.

[132] Ivan Dikic and Zvulun Elazar. Mechanism and medical implications of mammalian autophagy. *Nature Reviews. Molecular Cell Biology*, 19(6):349–364, June 2018.

[133] J. J. Lehman, P. M. Barger, A. Kovacs, J. E. Saffitz, D. M. Medeiros, and D. P. Kelly. Peroxisome proliferator-activated receptor gamma coactivator-1 promotes cardiac mitochondrial biogenesis. *The Journal of Clinical Investigation*, 106(7):847–856, October 2000.

[134] Zachary Gerhart-Hines, Joseph T. Rodgers, Olivia Bare, Carles Lerin, Seung-Hee Kim, Raul Mostoslavsky, Frederick W. Alt, Zhidan Wu, and Pere Puigserver. Metabolic control of muscle mitochondrial function and fatty acid oxidation through SIRT1/PGC-1alpha. *The EMBO journal*, 26(7):1913–1923, April 2007.

[135] Christopher D. Wiley, Michael C. Velarde, Pacome Lecot, Su Liu, Ethan A. Sarnoski, Adam Freund, Kotaro Shirakawa, Hyung W. Lim, Sonnet S. Davis, Arvind Ramanathan, Akos A. Gerencser, Eric Verdin, and Judith Campisi. Mitochondrial Dysfunction Induces Senescence with a Distinct Secretory Phenotype. *Cell Metabolism*, 23(2):303–314, February 2016.

[136] Yahui Wang, Ethan Stancliffe, Ronald Fowle-Grider, Rencheng Wang, Cheng Wang, Michaela Schwaiger-Haber, Leah P. Shriver, and Gary J. Patti. Saturation of the mitochondrial NADH shuttles drives aerobic glycolysis in proliferating cells. *Molecular Cell*, 82(17):3270–3283.e9, September 2022.

[137] B. Chaube and M. K. Bhat. AMPK, a key regulator of metabolic/energy homeostasis and mitochondrial biogenesis in cancer cells. *Cell Death & Disease*, 7(1):e2044, January 2016.

[138] Rebecca C. Rabinovitch, Bozena Samborska, Brandon Faubert, Eric H. Ma, Simon-Pierre Gravel, Sylvia Andrzejewski, Thomas C. Raissi, Arnim Pause, Julie St.-Pierre, and Russell G. Jones. AMPK Maintains Cellular Metabolic Homeostasis through Regulation of Mitochondrial Reactive Oxygen Species. *Cell Reports*, 21(1):1–9, October 2017.

[139] Fiona A. Ross, Thomas E. Jensen, and D. Grahame Hardie. Differential regulation by AMP and ADP of AMPK complexes containing different γ subunit isoforms. *The Biochemical Journal*, 473(2):189–199, January 2016.

[140] Joshua C. Drake, Rebecca J. Wilson, Rhianna C. Laker, Yuntian Guan, Hannah R. Spaulding, Anna S. Nichenko, Wenqing Shen, Huayu Shang, Maya V. Dorn, Kian Huang, Mei Zhang, Aloka B. Bandara, Matthew H. Brisendine, Jennifer A. Kashatus, Poonam R. Sharma, Alexander Young, Jitendra Gautam, Ruofan Cao, Horst Wallrabe, Paul A. Chang, Michael Wong, Eric M. Desjardins, Simon A. Hawley, George J. Christ, David F. Kashatus, Clint L. Miller, Matthew J. Wolf, Ammasi Periasamy, Gregory R. Steinberg, D. Grahame Hardie, and Zhen Yan. Mitochondria-localized AMPK responds to local energetics and contributes to exercise and energetic stress-induced mitophagy. *Proceedings of the National Academy of Sciences of the United States of America*, 118(37):e2025932118, September 2021.

[141] Huiyun Liang and Walter F. Ward. PGC-1alpha: A key regulator of energy metabolism. *Advances in Physiology Education*, 30(4):145–151, December 2006.

[142] John T. Cunningham, Joseph T. Rodgers, Daniel H. Arlow, Francisca Vazquez, Vamsi K. Mootha, and Pere Puigserver. mTOR controls mitochondrial oxidative function through a YY1-PGC-1alpha transcriptional complex. *Nature*, 450(7170):736–740, November 2007.

[143] Masataka Suwa, Hiroshi Nakano, and Shuzo Kumagai. Effects of chronic AICAR treatment on fiber composition, enzyme activity, UCP3, and PGC-1 in rat muscles. *Journal of Applied Physiology (Bethesda, Md.: 1985)*, 95(3):960–968, September 2003.

[144] Shin Terada, Masahide Goto, Miyuki Kato, Kentaro Kawanaka, Teruhiko Shimokawa, and Izumi Tabata. Effects of low-intensity prolonged exercise on PGC-1 mRNA expression in rat epitrochlearis muscle. *Biochemical and Biophysical Research Communications*, 296(2):350–354, August 2002.

[145] Xingxing Kong, Rui Wang, Yuan Xue, Xiaojun Liu, Huabing Zhang, Yong Chen, Fude Fang, and Yongsheng Chang. Sirtuin 3, a new target of PGC-1alpha, plays an important role in the suppression of ROS and mitochondrial biogenesis. *PloS One*, 5(7):e11707, July 2010.

[146] Wen Yang, Koji Nagasawa, Christian Münch, Yingjie Xu, Kyle Satterstrom, Seungmin Jeong, Sebastian D. Hayes, Mark P. Jedrychowski, F. Sejal Vyas, Elma Zaganjor, Virginia Guarani, Alison E. Ringel, Steven P. Gygi, J. Wade Harper, and Marcia C. Haigis. Mitochondrial Sirtuin Network Reveals Dynamic SIRT3-Dependent Deacetylation in Response to Membrane Depolarization. *Cell*, 167(4):985–1000.e21, November 2016.

[147] Michael Lazarou, Danielle A. Sliter, Lesley A. Kane, Shireen A. Sarraf, Chunxin Wang, Jonathon L. Burman, Dionisia P. Sideris, Adam I. Fogel, and Richard J. Youle. The ubiquitin kinase PINK1 recruits autophagy receptors to induce mitophagy. *Nature*, 524(7565):309–314, August 2015.

[148] Chandana Kondapalli, Agne Kazlauskaite, Ning Zhang, Helen I. Woodroof, David G. Campbell, Robert Gourlay, Lynn Burchell, Helen Walden, Thomas J. Macartney, Maria Deak, Axel Knebel, Dario R. Alessi, and Miratul M. K. Muqit. PINK1 is activated by mitochondrial membrane potential depolarization and stimulates Parkin E3 ligase activity by phosphorylating Serine 65. *Open Biology*, 2(5):120080, May 2012.

[149] Yang Mei, Yiru Zhang, Kazuo Yamamoto, Wei Xie, Tak W. Mak, and Han You. FOXO3a-dependent regulation of Pink1 (Park6) mediates survival signaling in response to cytokine deprivation. *Proceedings of the National Academy of Sciences of the United States of America*, 106(13):5153–5158, March 2009.

[150] Xiaoling Duan, Jade Tong, Qin Xu, Yili Wu, Fang Cai, Tingyu Li, and Weihong Song. Upregulation of human PINK1 gene expression by NFκB signalling. *Molecular Brain*, 7:57, August 2014.

[151] Mingming Tong, Daniela Zablocki, and Junichi Sadoshima. The role of Drp1 in mitophagy and cell death in the heart. *Journal of Molecular and Cellular Cardiology*, 142:138–145, May 2020.

[152] Alex P. Seabright, Nicholas H. F. Fine, Jonathan P. Barlow, Samuel O. Lord, Ibrahim Musa, Alexander Gray, Jack A. Bryant, Manuel Banzhaf, Gareth G. Lavery, D. Grahame Hardie, David J. Hodson, Andrew Philp, and Yu-Chiang Lai. AMPK activation induces mitophagy and promotes mitochondrial fission while activating TBK1 in a PINK1-Parkin independent manner. *The FASEB Journal*, 34(5):6284–6301, May 2020.

[153] Dieter A. Kubli and Åsa B. Gustafsson. Mitochondria and mitophagy: The yin and yang of cell death control. *Circulation Research*, 111(9):1208–1221, October 2012.

[154] Zhiyong Cheng. FoxO transcription factors in mitochondrial homeostasis. *The Biochemical Journal*, 479(4):525–536, February 2022.

[155] Tao Lan, Yu-chen Zheng, Ning-dao Li, Xiao-sheng Chen, Zhe Shen, and Bin Yan. CRISPR/dCas9-Mediated Parkin Inhibition Impairs Mitophagy and Aggravates Apoptosis of Rat Nucleus Pulposus Cells Under Oxidative Stress. *Frontiers in Molecular Biosciences*, 8:674632, April 2021.

[156] Angelika S. Rambold, Brenda Kostelecky, Natalie Elia, and Jennifer Lippincott-Schwartz. Tubular network formation protects mitochondria from autophagosomal degradation during nutrient starvation. *Proceedings of the National Academy of Sciences of the United States of America*, 108(25):10190–10195, June 2011.

[157] Angelika S. Rambold, Brenda Kostelecky, and Jennifer Lippincott-Schwartz. Together we are stronger: Fusion protects mitochondria from autophagosomal degradation. *Autophagy*, 7(12):1568–1569, December 2011.

[158] Søren Kahns, Michael Kalai, Lene Diness Jakobsen, Brian F.C. Clark, Peter Vandenabeele, and Poul Henning Jensen. Caspase-1 and Caspase-8 Cleave and Inactivate Cellular Parkin. *Journal of Biological Chemistry*, 278(26):23376–23380, June 2003.

[159] Marc Germain, Jaigi P. Mathai, Heidi M. McBride, and Gordon C. Shore. Endoplasmic reticulum BIK initiates DRP1-regulated remodelling of mitochondrial cristae during apoptosis. *The EMBO journal*, 24(8):1546–1556, April 2005.

[160] Sylwia Wasiak, Rodolfo Zunino, and Heidi M. McBride. Bax/Bak promote sumoylation of DRP1 and its stable association with mitochondria during apoptotic cell death. *The Journal of Cell Biology*, 177(3):439–450, May 2007.

[161] Naoko Taguchi, Naotada Ishihara, Akihiro Jofuku, Toshihiko Oka, and Katsuyoshi Mihara. Mitotic phosphorylation of dynamin-related GTPase Drp1 participates in mitochondrial fission. *The Journal of Biological Chemistry*, 282(15):11521–11529, April 2007.

[162] Sarah R. Horn, Michael J. Thomenius, Erika Segear Johnson, Christopher D. Freel, Judy Q. Wu, Jonathan L. Coloff, Chih-Sheng Yang, Wanli Tang, Jie An, Olga R. Ilkayeva, Jeffrey C. Rathmell, Christopher B. Newgard, and Sally Kornbluth. Regulation of mitochondrial morphology by APC/CCdh1-mediated control of Drp1 stability. *Molecular Biology of the Cell*, 22(8):1207–1216, April 2011.

[163] Erin Quan Toyama, Sébastien Herzig, Julien Courchet, Tommy L. Lewis, Oliver C. Losón, Kristina Hellberg, Nathan P. Young, Hsiuchen Chen, Franck Polleux, David C. Chan, and Reuben J. Shaw. Metabolism. AMP-activated protein kinase mediates mitochondrial fission in response to energy stress. *Science (New York, N.Y.)*, 351(6270):275–281, January 2016.

[164] Edith Jones, Norma Gaytan, Iraselia Garcia, Alan Herrera, Manuel Ramos, Divya Agarwala, Maahrose Rana, Wendy Innis-Whitehouse, Erin Schuenzel, and Robert Gilkerson. A threshold of transmembrane potential is required for mitochondrial dynamic balance mediated by DRP1 and OMA1. *Cellular and molecular life sciences: CMLS*, 74(7):1347–1363, April 2017.

[165] Hailong Han, Jieqiong Tan, Ruoxi Wang, Huida Wan, Yaohui He, Xinxiang Yan, Jifeng Guo, Qingtao Gao, Jie Li, Shuai Shang, Fang Chen, Runyi Tian, Wen Liu, Lujian Liao, Beisha Tang, and Zhuohua Zhang. PINK1 phosphorylates Drp1S616 to regulate mitophagy-independent mitochondrial dynamics. *EMBO reports*, 21(8):e48686, August 2020.

[166] Fumika Koyano, Koji Yamano, Hidetaka Kosako, Yoko Kimura, Mayumi Kimura, Yukio Fujiki, Keiji Tanaka, and Noriyuki Matsuda. Parkin-mediated ubiquitylation redistributes MITOL/March5 from mitochondria to peroxisomes. *EMBO reports*, 20(12):e47728, December 2019.

[167] Takeshi Tokuyama, Hideki Uosaki, Ayumu Sugiura, Gen Nishitai, Keisuke Takeda, Shun Nagashima, Isshin Shiiba, Naoki Ito, Taku Amo, Satoshi Mohri, Akiyuki Nishimura, Motohiro Nishida, Ayumu Konno, Hirokazu Hirai, Satoshi Ishido, Takahiro Yoshizawa, Takayuki Shindo, Shingo Takada, Shintaro Kinugawa, Ryoko Inatome, and Shigeru Yanagi. Protective roles of MITOL against myocardial senescence and ischemic injury partly via Drp1 regulation. *iScience*, 25(7):104582, July 2022.

[168] Jingxia Du, Hongchao Li, Jingjing Song, Tingting Wang, Yibo Dong, An Zhan, Yan Li, and Gaofeng Liang. AMPK Activation Alleviates Myocardial Ischemia-Reperfusion Injury by Regulating Drp1-Mediated Mitochondrial Dynamics. *Frontiers in Pharmacology*, 13:862204, 2022.

[169] Young Yeon Kim, Jee-Hyun Um, Jeong-Hyun Yoon, Da-Ye Lee, Yoon Jung Lee, Dong Hyun Kim, Joo-In Park, and Jeanho Yun. P53 regulates mitochondrial dynamics by inhibiting Drp1 translocation into mitochondria during cellular senescence. *FASEB journal: official publication of the Federation of American Societies for Experimental Biology*, 34(2):2451–2464, February 2020.

[170] Rong Yu, Urban Lendahl, Monica Nistér, and Jian Zhao. Regulation of Mammalian Mitochondrial Dynamics: Opportunities and Challenges. *Frontiers in Endocrinology*, 11:374, 2020.

[171] Michael P. Murphy. How mitochondria produce reactive oxygen species. *The Biochemical Journal*, 417(1):1–13, January 2009.

[172] Melinda T. Coughlan, David R. Thorburn, Sally A. Penfold, Adrienne Laskowski, Brooke E. Harcourt, Karly C. Sourris, Adeline L. Y. Tan, Kei Fukami, Vicki Thallas-Bonke, Peter P. Nawroth, Michael Brownlee, Angelika Bierhaus, Mark E. Cooper, and Josephine M. Forbes. RAGE-induced cytosolic ROS promote mitochondrial superoxide generation in diabetes. *Journal of the American Society of Nephrology: JASN*, 20(4):742–752, April 2009.

[173] Tianzheng Yu, James L. Robotham, and Yisang Yoon. Increased production of reactive oxygen species in hyperglycemic conditions requires dynamic change of mitochondrial morphology. *Proceedings of the National Academy of Sciences of the United States of America*, 103(8):2653–2658, February 2006.

[174] Jan Ježek, Katrina F. Cooper, and Randy Strich. The Impact of Mitochondrial Fission-Stimulated ROS Production on Pro-Apoptotic Chemotherapy. *Biology*, 10(1):33, January 2021.

[175] Cecilia Garcia-Perez, Soumya Sinha Roy, Shamim Naghdi, Xuena Lin, Erika Davies, and György Hajnóczky. Bid-induced mitochondrial membrane permeabilization waves propagated by local reactive oxygen species (ROS) signaling. *Proceedings of the National Academy of Sciences of the United States of America*, 109(12):4497–4502, March 2012.

[176] Meng-Liang Lin and Shih-Shun Chen. Activation of Casein Kinase II by Gallic Acid Induces BIK-BAX/BAK-Mediated ER Ca++-ROS-Dependent Apoptosis of Human Oral Cancer Cells. *Frontiers in Physiology*, 8:761, 2017.

[177] Satomi Miwa, Sonu Kashyap, Eduardo Chini, and Thomas von Zglinicki. Mitochondrial dysfunction in cell senescence and aging. *The Journal of Clinical Investigation*, 132(13):e158447, July 2022.

[178] Junghwan Lee, Seong-Ahn Lee, Sang-Hun Son, Ji-Ae Choi, Tam Doan Nguyen, Jaewhan Kim, Doyi Son, and Chang-Hwa Song. Impaired mitophagy induces antimicrobial responses in macrophages infected with Mycobacterium tuberculosis. *Cell & Bioscience*, 13(1):158, August 2023.

[179] Tanveer Ahmad, Isaac K. Sundar, Chad A. Lerner, Janice Gerloff, Ana M. Tormos, Hongwei Yao, and Irfan Rahman. Impaired mitophagy leads to cigarette smoke stress-induced cellular senescence: Implications for chronic obstructive pulmonary disease. *FASEB journal: official publication of the Federation of American Societies for Experimental Biology*, 29(7):2912–2929, July 2015.

[180] Sergio Rius-Pérez, Isabel Torres-Cuevas, Iván Millán, Ángel L. Ortega, and Salvador Pérez. PGC-1α, Inflammation, and Oxidative Stress: An Integrative View in Metabolism. *Oxidative Medicine and Cellular Longevity*, 2020:1452696, 2020.

[181] S. S. Korshunov, V. P. Skulachev, and A. A. Starkov. High protonic potential actuates a mechanism of production of reactive oxygen species in mitochondria. *FEBS letters*, 416(1):15–18, October 1997.

[182] João F Passos, Glyn Nelson, Chunfang Wang, Torsten Richter, Cedric Simillion, Carole J Proctor, Satomi Miwa, Sharon Olijslagers, Jennifer Hallinan, Anil Wipat, Gabriele Saretzki, Karl Lenhard Rudolph, Tom B L Kirkwood, and Thomas von Zglinicki. Feedback between p21 and reactive oxygen production is necessary for cell senescence. *Molecular Systems Biology*, 6(1):347, January 2010.

[183] Inmaculada Martínez-Reyes and Navdeep S. Chandel. Mitochondrial TCA cycle metabolites control physiology and disease. *Nature Communications*, 11(1):102, January 2020.

[184] Jean Bastin, Marine Sroussi, Ivan Nemazanyy, Pierre Laurent-Puig, Sophie Mouillet-Richard, and Fatima Djouadi. Downregulation of mitochondrial complex I induces ROS production in colorectal cancer subtypes that differently controls migration. *Journal of Translational Medicine*, 21(1):522, August 2023.

[185] Magali R. VanLinden, Christian Dölle, Ina K. N. Pettersen, Veronika A. Kulikova, Marc Niere, Gennaro Agrimi, Sissel E. Dyrstad, Ferdinando Palmieri, Andrey A. Nikiforov, Karl Johan Tronstad, and Mathias Ziegler. Subcellular Distribution of NAD+ between Cytosol and Mitochondria Determines the Metabolic Profile of Human Cells. *The Journal of Biological Chemistry*, 290(46):27644–27659, November 2015.

[186] K. Wesley Overton, Sabrina L. Spencer, William L. Noderer, Tobias Meyer, and Clifford L. Wang. Basal p21 controls population heterogeneity in cycling and quiescent cell cycle states. *Proceedings of the National Academy of Sciences of the United States of America*, 111(41):E4386–4393, October 2014.

[187] Hasan Zalzali, Bilal Nasr, Mohamad Harajly, Hussein Basma, Farah Ghamloush, Sandra Ghayad, Noël Ghanem, Gerard I Evan, and Raya Saab. CDK2 Transcriptional Repression Is an Essential Effector in p53-Dependent Cellular Senescence—Implications for Therapeutic Intervention. *Molecular Cancer Research*, 13(1):29–40, January 2015.

[188] Zhimin Lu and Tony Hunter. Ubiquitylation and proteasomal degradation of the p21(Cip1), p27(Kip1) and p57(Kip2) CDK inhibitors. *Cell Cycle (Georgetown, Tex.)*, 9(12):2342–2352, June 2010.

[189] J. L. Gervais, P. Seth, and H. Zhang. Cleavage of CDK inhibitor p21(Cip1/Waf1) by caspases is an early event during DNA damage-induced apoptosis. *The Journal of Biological Chemistry*, 273(30):19207–19212, July 1998.

[190] Olivier Coqueret. New roles for p21 and p27 cell-cycle inhibitors: A function for each cell compartment? *Trends in cell biology*, 13(2):65–70, 2003.

[191] J William Harbour, Robin X Luo, Angeline Dei Santi, Antonio A Postigo, and Douglas C Dean. Cdk phosphorylation triggers sequential intramolecular interactions that progressively block Rb functions as cells move through G1. *Cell*, 98(6):859–869, 1999.

[192] Bela Novak and John J Tyson. A model for restriction point control of the mammalian cell cycle. *Journal of Theoretical Biology*, 230(4):563–579, October 2004.

[193] Yoichi Taya. RB kinases and RB-binding proteins: New points of view. *Trends in biochemical sciences*, 22(1):14–17, 1997.

[194] Philip W Hinds, Sibylle Mittnacht, Vjekoslav Dulic, Andrew Arnold, Steven I Reed, and Robert A Weinberg. Regulation of retinoblastoma protein functions by ectopic expression of human cyclins. *Cell*, 70(6):993–1006, 1992.

[195] JY Kato, Hitoshi Matsushime, Scott W Hiebert, Mark E Ewen, and Charles J Sherr. Direct binding of cyclin D to the retinoblastoma gene product (pRb) and pRb phosphorylation by the cyclin D-dependent kinase CDK4. *Genes and Development*, 7:331–331, 1993.

[196] Mark E Ewen, Hayla K Sluss, Charles J Sherr, Hitoshi Matsushime, Jun-ya Kato, and David M Livingston. Functional interactions of the retinoblastoma protein with mammalian D-type cyclins. *Cell*, 73(3):487–497, 1993.

[197] C. L. Fattman, S. M. Delach, Q. P. Dou, and D. E. Johnson. Sequential two-step cleavage of the retinoblastoma protein by caspase-3/-7 during etoposide-induced apoptosis. *Oncogene*, 20(23):2918–2926, May 2001.

[198] Noriko Ishida, Taichi Hara, Takumi Kamura, Minoru Yoshida, Keiko Nakayama, and Keiichi I Nakayama. Phosphorylation of p27 Kip1 on serine 10 is required for its binding to CRM1 and nuclear export. *Journal of Biological Chemistry*, 277(17):14355–14358, 2002.

[199] A. Faure, A. Naldi, C. Chaouiya, and D. Thieffry. Dynamical analysis of a generic Boolean model for the control of the mammalian cell cycle. *Bioinformatics*, 22(14):e124–e131, July 2006.

[200] Jiyong Liang, Shan H. Shao, Zhi-Xiang Xu, Bryan Hennessy, Zhiyong Ding, Michelle Larrea, Seiji Kondo, Dan J. Dumont, Jordan U. Gutterman, Cheryl L. Walker, Joyce M. Slingerland, and Gordon B. Mills. The energy sensing LKB1-AMPK pathway regulates p27(kip1) phosphorylation mediating the decision to enter autophagy or apoptosis. *Nature Cell Biology*, 9(2):218–224, February 2007.

[201] D Müller, C Bouchard, B Rudolph, P Steiner, I Stuckmann, R Saffrich, W Ansorge, W Huttner, and M Eilers. Cdk2-dependent phosphorylation of p27 facilitates its Myc-induced release from cyclin E/cdk2 complexes. *Oncogene*, 15(21):2561–2576, November 1997.

[202] Robert J Sheaff, Mark Groudine, Matthew Gordon, James M Roberts, and Bruce E Clurman. Cyclin E-CDK2 is a regulator of p27Kip1. *Genes & development*, 11(11):1464–1478, 1997.

[203] R. H. Medema, G. J. Kops, J. L. Bos, and B. M. Burgering. AFX-like Forkhead transcription factors mediate cell-cycle regulation by Ras and PKB through p27kip1. *Nature*, 404(6779):782–787, April 2000.

[204] Alessia Montagnoli, Francesca Fiore, Esther Eytan, Andrea C Carrano, Giulio F Draetta, Avram Hershko, and Michele Pagano. Ubiquitination of p27 is regulated by Cdk-dependent phosphorylation and trimeric complex formation. *Genes & development*, 13(9):1181–1189, 1999.

[205] B. Eymin, O. Sordet, N. Droin, B. Munsch, M. Haugg, M. Van de Craen, P. Vandenabeele, and E. Solary. Caspase-induced proteolysis of the cyclin-dependent kinase inhibitor p27Kip1 mediates its anti-apoptotic activity. *Oncogene*, 18(34):4839–4847, August 1999.

[206] B. Levkau, H. Koyama, E. W. Raines, B. E. Clurman, B. Herren, K. Orth, J. M. Roberts, and R. Ross. Cleavage of p21Cip1/Waf1 and p27Kip1 mediates apoptosis in endothelial cells through activation of Cdk2: Role of a caspase cascade. *Molecular Cell*, 1(4):553–563, March 1998.

[207] R. Sears, F. Nuckolls, E. Haura, Y. Taya, K. Tamai, and J. R. Nevins. Multiple Ras-dependent phosphorylation pathways regulate Myc protein stability. *Genes & Development*, 14(19):2501–2514, October 2000.

[208] MF Roussel, JN Davis, JL Cleveland, J Ghysdael, and SW Hiebert. Dual control of myc expression through a single DNA binding site targeted by ets family proteins and E2F-1. *Oncogene*, 9(2):405–415, 1994.

[209] Jenny S. L. Ho, Weili Ma, Daniel Y. L. Mao, and Samuel Benchimol. P53-Dependent transcriptional repression of c-myc is required for G1 cell cycle arrest. *Molecular and Cellular Biology*, 25(17):7423–7431, September 2005.

[210] B. Lutterbach and S. R. Hann. Hierarchical phosphorylation at N-terminal transformation-sensitive sites in c-Myc protein is regulated by mitogens and in mitosis. *Molecular and Cellular Biology*, 14(8):5510–5522, August 1994.

[211] Chen-Ju Lin, Abba Malina, and Jerry Pelletier. C-Myc and eIF4F constitute a feedforward loop that regulates cell growth: Implications for anticancer therapy. *Cancer Research*, 69(19):7491–7494, October 2009.

[212] Markus Welcker, Amir Orian, Jianping Jin, Jonathan E. Grim, Jonathan A. Grim, J. Wade Harper, Robert N. Eisenman, and Bruce E. Clurman. The Fbw7 tumor suppressor regulates glycogen synthase kinase 3 phosphorylation-dependent c-Myc protein degradation. *Proceedings of the National Academy of Sciences of the United States of America*, 101(24):9085–9090, June 2004.

[213] E. Batsché, M. Lipp, and C. Cremisi. Transcriptional repression and activation in the same cell type of the human c-MYC promoter by the retinoblastoma gene protein: Antagonisation of both effects by SV40 T antigen. *Oncogene*, 9(8):2235–2243, August 1994.

[214] F Oswald, H Lovec, T Möröy, and M Lipp. E2F-dependent regulation of human MYC: Trans-activation by cyclins D1 and A overrides tumour suppressor protein functions. *Oncogene*, 9(7):2029–2036, July 1994.

[215] K Thalmeier, H Synovzik, R Mertz, EL Winnacker, and M Lipp. Nuclear factor E2F mediates basic transcription and trans-activation by E1a of the human MYC promoter. *Genes & development*, 3(4):527–536, 1989.

[216] JY Leung, GL Ehmann, PH Giangrande, and JR Nevins. A role for Myc in facilitating transcription activation by E2F1. *Oncogene*, 27(30):4172–4179, 2008.

[217] A. Asli Hizli, Adrian R. Black, Marybeth A. Pysz, and Jennifer D. Black. Protein kinase C alpha signaling inhibits cyclin D1 translation in intestinal epithelial cells. *The Journal of Biological Chemistry*, 281(21):14596–14603, May 2006.

[218] J. A. Diehl, M. Cheng, M. F. Roussel, and C. J. Sherr. Glycogen synthase kinase-3beta regulates cyclin D1 proteolysis and subcellular localization. *Genes & Development*, 12(22):3499–3511, November 1998.

[219] J Wade Harper, Stephen J Elledge, Khandan Keyomarsi, Brian Dynlacht, Li-Huei Tsai, Pumin Zhang, Steven Dobrowolski, Connell Bai, Lisa Connell-Crowley, and Eric Swindell. Inhibition of cyclin-dependent kinases by p21. *Molecular biology of the cell*, 6(4):387–400, 1995.

[220] Yue Xiong, Gregory J Hannon, Hui Zhang, David Casso, Ryuji Kobayashi, and David Beach. P21 is a universal inhibitor of cyclin kinases. *nature*, 366(6456):701–704, 1993.

[221] Srikumar P Chellappan, Scott Hiebert, Maria Mudryj, Jonathan M Horowitz, and Joseph R Nevins. The E2F transcription factor is a cellular target for the RB protein. *Cell*, 65(6):1053–1061, 1991.

[222] Masahiro Hitomi and Dennis W Stacey. Cyclin D1 production in cycling cells depends on ras in a cell-cycle-specific manner. *Current biology*, 9(19):1075–S2, 1999.

[223] Huseyin Aktas, Hong Cai, and Geoffrey M Cooper. Ras links growth factor signaling to the cell cycle machinery via regulation of cyclin D1 and the Cdk inhibitor p27KIP1. *Molecular and cellular biology*, 17(7):3850–3857, 1997.

[224] Jasmine I Daksis, Richard Y Lu, Linda M Facchini, Wilson W Marhin, and LJ Penn. Myc induces cyclin D1 expression in the absence of de novo protein synthesis and links mitogen-stimulated signal transduction to the cell cycle. *Oncogene*, 9(12):3635–3645, 1994.

[225] MK Mateyak, AJ Obaya, and JM Sedivy. C-Myc regulates cyclin D-Cdk4 and-Cdk6 activity but affects cell cycle progression at multiple independent points. *Molecular and cellular biology*, 19(7):4672–4683, 1999.

[226] Itaru Matsumura, Hirokazu Tanaka, and Yuzuru Kanakura. E2F1 and c-Myc in cell growth and death. *Cell Cycle*, 2(4):332–335, 2003.

[227] Zhi-yi Guo, Xiao-hui Hao, Fei-Fei Tan, Xin Pei, Li-Mei Shang, Xue-lian Jiang, and Fang Yang. The elements of human cyclin D1 promoter and regulation involved. *Clinical epigenetics*, 2(2):63–76, 2011.

[228] J Fan and J R Bertino. Functional roles of E2F in cell cycle regulation. *Oncogene*, 14(10):1191–1200, March 1997.

[229] Masahiro Hitomi, Ke Yang, Andrew W. Stacey, and Dennis W. Stacey. Phosphorylation of cyclin D1 regulated by ATM or ATR controls cell cycle progression. *Molecular and Cellular Biology*, 28(17):5478–5493, September 2008.

[230] Sonia Rocha, Anthea M. Martin, David W. Meek, and Neil D. Perkins. P53 represses cyclin D1 transcription through down regulation of Bcl-3 and inducing increased association of the p52 NF-kappaB subunit with histone deacetylase 1. *Molecular and Cellular Biology*, 23(13):4713–4727, July 2003.

[231] Gustavo Leone, James DeGregori, Rosalie Sears, Laszlo Jakoi, and Joseph R Nevins. Myc and Ras collaborate in inducing accumulation of active cyclin E/Cdk2 and E2F. *Nature*, 387(6631):422–426, 1997.

[232] Hirokazu Tanaka, Itaru Matsumura, Sachiko Ezoe, Yusuke Satoh, Toshiyuki Sakamaki, Chris Albanese, Takashi Machii, Richard G Pestell, and Yuzuru Kanakura. E2F1 and c-Myc potentiate apoptosis through inhibition of NF-κB activity that facilitates MnSOD-mediated ROS elimination. *Molecular cell*, 9(5):1017–1029, 2002.

[233] Peng Dong, Manoj V Maddali, Jaydeep K Srimani, François Thélot, Joseph R Nevins, Bernard Mathey-Prevot, and Lingchong You. Division of labour between Myc and G1 cyclins in cell cycle commitment and pace control. *Nature Communications*, 5:4750, 2014.

[234] David G Johnson, Kiyoshi Ohtani, and Joseph R Nevins. Autoregulatory control of E2F1 expression in response to positive and negative regulators of cell cycle progression. *Genes & Development*, 8(13):1514–1525, 1994.

[235] S J Weintraub, C A Prater, and D C Dean. Retinoblastoma protein switches the E2F site from positive to negative element. *Nature*, 358(6383):259–261, July 1992.

[236] W Krek, M E Ewen, S Shirodkar, Z Arany, W G Kaelin, and D M Livingston. Negative regulation of the growth-promoting transcription factor E2F-1 by a stably bound cyclin A-dependent protein kinase. *Cell*, 78(1):161–172, July 1994.

[237] M Xu, K A Sheppard, C Y Peng, A S Yee, and H Piwnica-Worms. Cyclin A/CDK2 binds directly to E2F-1 and inhibits the DNA-binding activity of E2F-1/DP-1 by phosphorylation. *Molecular and cellular biology*, 14(12):8420–8431, December 1994.

[238] Kristian Helin. Regulation of cell proliferation by the E2F transcription factors. *Current opinion in genetics & development*, 8(1):28–35, 1998.

[239] Robert J Sheaff, Mark Groudine, Matthew Gordon, James M Roberts, and Bruce E Clurman. Cyclin E-CDK2 is a regulator of p27Kip1. *Genes & development*, 11(11):1464–1478, 1997.

[240] Kiyoshi Ohtani, James Degregori, and JOSEPH R Nevins. Regulation of the cyclin E gene by transcription factor E2F1. *Proceedings of the National Academy of Sciences*, 92(26):12146–12150, 1995.

[241] Yan Geng, Young-Mi Lee, Markus Welcker, Jherek Swanger, Agnieszka Zagozdzon, Joel D. Winer, James M. Roberts, Philipp Kaldis, Bruce E. Clurman, and Piotr Sicinski. Kinase-independent function of cyclin E. *Molecular Cell*, 25(1):127–139, January 2007.

[242] Cara L Lunn, John C Chrivia, and Joseph J Baldassare. Activation of Cdk2/Cyclin E complexes is dependent on the origin of replication licensing factor Cdc6 in mammalian cells. *Cell Cycle*, 9(22):4533–4541, November 2010.

[243] J. W. Harper, G. R. Adami, N. Wei, K. Keyomarsi, and S. J. Elledge. The p21 Cdk-interacting protein Cip1 is a potent inhibitor of G1 cyclin-dependent kinases. *Cell*, 75(4):805–816, November 1993.

[244] Claus Storgaard Sørensen, Randi G Syljuåsen, Jacob Falck, Tine Schroeder, Lars Rönnstrand, Kum Kum Khanna, Bin-Bing Zhou, Jiri Bartek, and Jiri Lukas. Chk1 regulates the S phase checkpoint by coupling the physiological turnover and ionizing radiation-induced accelerated proteolysis of Cdc25A. *Cancer cell*, 3(3):247–258, March 2003.

[245] J. Falck, N. Mailand, R. G. Syljuåsen, J. Bartek, and J. Lukas. The ATM-Chk2-Cdc25A checkpoint pathway guards against radioresistant DNA synthesis. *Nature*, 410(6830):842–847, April 2001.

[246] Suparna Mazumder, Bendi Gong, Quan Chen, Judith A. Drazba, Jeffrey C. Buchsbaum, and Alexandru Almasan. Proteolytic cleavage of cyclin E leads to inactivation of associated kinase activity and amplification of apoptosis in hematopoietic cells. *Molecular and Cellular Biology*, 22(7):2398–2409, April 2002.

[247] K. Ohtani, J. DeGregori, G. Leone, D. R. Herendeen, T. J. Kelly, and J. R. Nevins. Expression of the HsOrc1 gene, a human ORC1 homolog, is regulated by cell proliferation via the E2F transcription factor. *Molecular and Cellular Biology*, 16(12):6977–6984, December 1996.

[248] Melvin L. DePamphilis, J. Julian Blow, Soma Ghosh, Tapas Saha, Kohji Noguchi, and Alex Vassilev. Regulating the licensing of DNA replication origins in metazoa. *Current Opinion in Cell Biology*, 18(3):231–239, June 2006.

[249] B. O. Petersen, J. Lukas, C. S. Sørensen, J. Bartek, and K. Helin. Phosphorylation of mammalian CDC6 by cyclin A/CDK2 regulates its subcellular localization. *The EMBO journal*, 18(2):396–410, January 1999.

[250] Z. Yan, J. DeGregori, R. Shohet, G. Leone, B. Stillman, J. R. Nevins, and R. S. Williams. Cdc6 is regulated by E2F and is essential for DNA replication in mammalian cells. *Proceedings of the National Academy of Sciences of the United States of America*, 95(7):3603–3608, March 1998.

[251] Hyungshin Yim and Raymond L. Erikson. Cell division cycle 6, a mitotic substrate of polo-like kinase 1, regulates chromosomal segregation mediated by cyclin-dependent kinase 1 and separase. *Proceedings of the National Academy of Sciences of the United States of America*, 107(46):19742–19747, November 2010.

[252] Cristina Pelizon, Fabrizio d'Adda di Fagagna, Lorena Farrace, and Ronald A. Laskey. Human replication protein Cdc6 is selectively cleaved by caspase 3 during apoptosis. *EMBO reports*, 3(8):780–784, August 2002.

[253] Kenichi Yoshida and Ituro Inoue. Regulation of Geminin and Cdt1 expression by E2F transcription factors. *Oncogene*, 23(21):3802–3812, May 2004.

[254] Taras Valovka, Manuela Schönfeld, Philipp Raffeiner, Kathrin Breuker, Theresia Dunzendorfer-Matt, Markus Hartl, and Klaus Bister. Transcriptional control of DNA replication licensing by Myc. *Scientific Reports*, 3:3444, December 2013.

[255] Ken-ichiro Yanagi, Takeshi Mizuno, Zhiying You, and Fumio Hanaoka. Mouse geminin inhibits not only Cdt1-MCM6 interactions but also a novel intrinsic Cdt1 DNA binding activity. *The Journal of Biological Chemistry*, 277(43):40871–40880, October 2002.

[256] Linda Clijsters, Janneke Ogink, and Rob Wolthuis. The spindle checkpoint, APC/C(Cdc20), and APC/C(Cdh1) play distinct roles in connecting mitosis to S phase. *J Cell Biol*, 201(7):1013–1026, June 2013.

[257] Irene García-Higuera, Eusebio Manchado, Pierre Dubus, Marta Cañamero, Juan Méndez, Sergio Moreno, and Marcos Malumbres. Genomic stability and tumour suppression by the APC/C cofactor Cdh1. *Nature Cell Biology*, 10(7):802–811, July 2008.

[258] Jared E. Toettcher, Alexander Loewer, Gerard J. Ostheimer, Michael B. Yaffe, Bruce Tidor, and Galit Lahav. Distinct mechanisms act in concert to mediate cell cycle arrest. *Proceedings of the National Academy of Sciences of the United States of America*, 106(3):785–790, January 2009.

[259] Kevin B. Spurgers, David L. Gold, Kevin R. Coombes, Nicole L. Bohnenstiehl, Brian Mullins, Raymond E. Meyn, Christopher J. Logothetis, and Timothy J. McDonnell. Identification of cell cycle regulatory genes as principal targets of p53-mediated transcriptional repression. *The Journal of Biological Chemistry*, 281(35):25134–25142, September 2006.

[260] K. E. Knudsen, A. F. Fribourg, M. W. Strobeck, J. M. Blanchard, and E. S. Knudsen. Cyclin A is a functional target of retinoblastoma tumor suppressor protein-mediated cell cycle arrest. *The Journal of Biological Chemistry*, 274(39):27632–27641, September 1999.

[261] Lucía García-Gutiérrez, Gabriel Bretones, Ester Molina, Ignacio Arechaga, Catherine Symonds, Juan C. Acosta, Rosa Blanco, Adrián Fernández, Leticia Alonso, Piotr Sicinski, Mariano Barbacid, David Santamaría, and Javier León. Myc stimulates cell cycle progression through the activation of Cdk1 and phosphorylation of p27. *Scientific Reports*, 9(1):18693, December 2019.

[262] Il-Man Kim, Timothy Ackerson, Sneha Ramakrishna, Maria Tretiakova, I.-Ching Wang, Tanya V. Kalin, Michael L. Major, Galina A. Gusarova, Helena M. Yoder, Robert H. Costa, and Vladimir V. Kalinichenko. The Forkhead Box m1 transcription factor stimulates the proliferation of tumor cells during development of lung cancer. *Cancer Research*, 66(4):2153–2161, February 2006.

[263] Tanya V. Kalin, I.-Ching Wang, Timothy J. Ackerson, Michael L. Major, Carol J. Detrisac, Vladimir V. Kalinichenko, Alexander Lyubimov, and Robert H. Costa. Increased levels of the FoxM1 transcription factor accelerate development and progression of prostate carcinomas in both TRAMP and LADY transgenic mice. *Cancer Research*, 66(3):1712–1720, February 2006.

[264] Jamila Laoukili, Matthijs R. H. Kooistra, Alexandra Brás, Jos Kauw, Ron M. Kerkhoven, Ashby Morrison, Hans Clevers, and René H. Medema. FoxM1 is required for execution of the mitotic programme and chromosome stability. *Nature Cell Biology*, 7(2):126–136, February 2005.

[265] Mónica Alvarez-Fernández, Vincentius A. Halim, Lenno Krenning, Melinda Aprelia, Shabaz Mohammed, Albert J. Heck, and René H. Medema. Recovery from a DNA-damage-induced G2 arrest requires Cdk-dependent activation of FoxM1. *EMBO reports*, 11(6):452–458, June 2010.

[266] Moe Tategu, Hiroki Nakagawa, Kaori Sasaki, Rieko Yamauchi, Sota Sekimachi, Yuka Suita, Naoko Watanabe, and Kenichi Yoshid. Transcriptional regulation of human polo-like kinases and early mitotic inhibitor. *Journal of Genetics and Genomics = Yi Chuan Xue Bao*, 35(4):215–224, April 2008.

[267] Jerry Y. Hsu, Julie D. R. Reimann, Claus S. Sørensen, Jiri Lukas, and Peter K. Jackson. E2F-dependent accumulation of hEmi1 regulates S phase entry by inhibiting APC(Cdh1). *Nature Cell Biology*, 4(5):358–366, May 2002.

[268] Jinho Lee, Jin Ah Kim, Valerie Barbier, Arun Fotedar, and Rati Fotedar. DNA damage triggers p21WAF1-dependent Emi1 down-regulation that maintains G2 arrest. *Molecular Biology of the Cell*, 20(7):1891–1902, April 2009.

[269] David V. Hansen, Alexander V. Loktev, Kenneth H. Ban, and Peter K. Jackson. Plk1 regulates activation of the anaphase promoting complex by phosphorylating and triggering SCFbetaTrCP-dependent destruction of the APC Inhibitor Emi1. *Molecular Biology of the Cell*, 15(12):5623–5634, December 2004.

[270] Huafeng Pan, Yudi Zhu, Wei Wei, Siliang Shao, and Xin Rui. Transcription factor FoxM1 is the downstream target of c-Myc and contributes to the development of prostate cancer. *World Journal of Surgical Oncology*, 16(1):59, March 2018.

[271] Juliane M. Lüscher-Firzlaff, Richard Lilischkis, and Bernhard Lüscher. Regulation of the transcription factor FOXM1c by Cyclin E/CDK2. *FEBS letters*, 580(7):1716–1722, March 2006.

[272] Jamila Laoukili, Monica Alvarez, Lars A. T. Meijer, Marie Stahl, Shabaz Mohammed, Livio Kleij, Albert J. R. Heck, and René H. Medema. Activation of FoxM1 during G2 requires cyclin A/Cdk-dependent relief of autorepression by the FoxM1 N-terminal domain. *Molecular and Cellular Biology*, 28(9):3076–3087, May 2008.

[273] Con Sullivan, Youhong Liu, Jingjing Shen, Adam Curtis, Christina Newman, Janet M. Hock, and Xiong Li. Novel interactions between FOXM1 and CDC25A regulate the cell cycle. *PloS One*, 7(12):e51277, 2012.

[274] Michael L. Major, Rita Lepe, and Robert H. Costa. Forkhead box M1B transcriptional activity requires binding of Cdk-cyclin complexes for phosphorylation-dependent recruitment of p300/CBP coactivators. *Molecular and Cellular Biology*, 24(7):2649–2661, April 2004.

[275] Zheng Fu, Liviu Malureanu, Jun Huang, Wei Wang, Hao Li, Jan M. van Deursen, Donald J. Tindall, and Junjie Chen. Plk1-dependent phosphorylation of FoxM1 regulates a transcriptional programme required for mitotic progression. *Nature Cell Biology*, 10(9):1076–1082, September 2008.

[276] E. Vigo, H. Müller, E. Prosperini, G. Hateboer, P. Cartwright, M. C. Moroni, and K. Helin. CDC25A phosphatase is a target of E2F and is required for efficient E2F-induced S phase. *Molecular and Cellular Biology*, 19(9):6379–6395, September 1999.

[277] L. Wu, E. C. Goodwin, L. K. Naeger, E. Vigo, K. Galaktionov, K. Helin, and D. DiMaio. E2F-Rb complexes assemble and inhibit cdc25A transcription in cervical carcinoma cells following repression of human papillomavirus oncogene expression. *Molecular and Cellular Biology*, 20(19):7059–7067, October 2000.

[278] Maddalena Donzelli, Massimo Squatrito, Dvora Ganoth, Avram Hershko, Michele Pagano, and Giulio F Draetta. Dual mode of degradation of Cdc25 A phosphatase. *The EMBO journal*, 21(18):4875–4884, 2002.

[279] Eusebio Manchado, Manuel Eguren, and Marcos Malumbres. The anaphase-promoting complex/cyclosome (APC/C): Cell-cycle-dependent and -independent functions. *Biochem Soc Trans*, 38(Pt 1):65–71, February 2010.

[280] Tiebang Kang, Yongkun Wei, Yuchi Honaker, Hiroshi Yamaguchi, Ettore Appella, Mien-Chie Hung, and Helen Piwnica-Worms. GSK-3 beta targets Cdc25A for ubiquitin-mediated proteolysis, and GSK-3 beta inactivation correlates with Cdc25A overproduction in human cancers. *Cancer Cell*, 13(1):36–47, January 2008.

[281] Mei-Shya Chen, Christine E. Ryan, and Helen Piwnica-Worms. Chk1 kinase negatively regulates mitotic function of Cdc25A phosphatase through 14-3-3 binding. *Molecular and Cellular Biology*, 23(21):7488–7497, November 2003.

[282] Yong-Sheng Tu, Xiao-Long Kang, Jia-Guo Zhou, Xiao-Fei Lv, Yong-Bo Tang, and Yong-Yuan Guan. Involvement of Chk1-Cdc25A-cyclin A/CDK2 pathway in simvastatin induced S-phase cell cycle arrest and apoptosis in multiple myeloma cells. *European Journal of Pharmacology*, 670(2-3):356–364, November 2011.

[283] I Hoffmann, G Draetta, and E Karsenti. Activation of the phosphatase activity of human cdc25A by a cdk2-cyclin E dependent phosphorylation at the G1/S transition. *EMBO J*, 13(18):4302–4310, September 1994.

[284] Laurent Mazzolini, Anaïs Broban, Carine Froment, Odile Burlet-Schiltz, Arnaud Besson, Stéphane Manenti, and Christine Dozier. Phosphorylation of CDC25A on SER283 in late S/G2 by CDK/cyclin complexes accelerates mitotic entry. *Cell Cycle (Georgetown, Tex.)*, 15(20):2742–2752, October 2016.

[285] Niels Mailand, Alexandre V. Podtelejnikov, Anja Groth, Matthias Mann, Jiri Bartek, and Jiri Lukas. Regulation of G(2)/M events by Cdc25A through phosphorylation-dependent modulation of its stability. *The EMBO journal*, 21(21):5911–5920, November 2002.

[286] Ida Blomberg and Ingrid Hoffmann. Ectopic expression of Cdc25A accelerates the G1/S transition and leads to premature activation of cyclin E-and cyclin A-dependent kinases. *Molecular and cellular biology*, 19(9):6183–6194, 1999.

[287] S. Geley, E. Kramer, C. Gieffers, J. Gannon, J. M. Peters, and T. Hunt. Anaphase-promoting complex/cyclosome-dependent proteolysis of human cyclin A starts at the beginning of mitosis and is not subject to the spindle assembly checkpoint. *The Journal of Cell Biology*, 153(1):137–148, April 2001.

[288] N. den Elzen and J. Pines. Cyclin A is destroyed in prometaphase and can delay chromosome alignment and anaphase. *The Journal of Cell Biology*, 153(1):121–136, April 2001.

[289] Barbara Di Fiore and Jonathon Pines. How cyclin A destruction escapes the spindle assembly checkpoint. *The Journal of Cell Biology*, 190(4):501–509, August 2010.

[290] Yuko Katsuno, Ayumi Suzuki, Kazuto Sugimura, Katsuzumi Okumura, Doaa H Zineldeen, Midori Shimada, Hiroyuki Niida, Takeshi Mizuno, Fumio Hanaoka, and Makoto Nakanishi. Cyclin A-Cdk1 regulates the origin firing program in mammalian cells. *Proceedings of the National Academy of Sciences*, 106(9):3184–3189, March 2009.

[291] J W Harper. The anaphase-promoting complex: It's not just for mitosis any more. *Genes & Development*, 16(17):2179–2206, September 2002.

[292] Michael Rape and Marc W. Kirschner. Autonomous regulation of the anaphase-promoting complex couples mitosis to S-phase entry. *Nature*, 432(7017):588–595, December 2004.

[293] Yuichi J. Machida and Anindya Dutta. The APC/C inhibitor, Emi1, is essential for prevention of rereplication. *Genes & Development*, 21(2):184–194, January 2007.

[294] J. D. Reimann, E. Freed, J. Y. Hsu, E. R. Kramer, J. M. Peters, and P. K. Jackson. Emi1 is a mitotic regulator that interacts with Cdc20 and inhibits the anaphase promoting complex. *Cell*, 105(5):645–655, June 2001.

[295] J. D. Reimann, B. E. Gardner, F. Margottin-Goguet, and P. K. Jackson. Emi1 regulates the anaphase-promoting complex by a different mechanism than Mad2 proteins. *Genes & Development*, 15(24):3278–3285, December 2001.

[296] Richard W Deibler and Marc W. Kirschner. Quantitative reconstitution of mitotic CDK1 activation in somatic cell extracts. *Mol Cell*, 37(6):753–767, March 2010.

[297] Joon Lee, Akiko Kumagai, and William G Dunphy. Positive regulation of Wee1 by Chk1 and 14-3-3 proteins. *Molecular biology of the cell*, 12(3):551–563, 2001.

[298] Raquel Domínguez-Kelly, Yusé Martín, Stephane Koundrioukoff, Marvin E. Tanenbaum, Veronique A. J. Smits, René H. Medema, Michelle Debatisse, and Raimundo Freire. Wee1 controls genomic stability during replication by regulating the Mus81-Eme1 endonuclease. *The Journal of Cell Biology*, 194(4):567–579, August 2011.

[299] Nobumoto Watanabe, Harumi Arai, Jun-Ichi Iwasaki, Masaaki Shiina, Kazuhiro Ogata, Tony Hunter, and Hiroyuki Osada. Cyclin-dependent kinase (CDK) phosphorylation destabilizes somatic Wee1 via multiple pathways. *Proceedings of the National Academy of Sciences of the United States of America*, 102(33):11663–11668, August 2005.

[300] B. B. Zhou, H. Li, J. Yuan, and M. W. Kirschner. Caspase-dependent activation of cyclin-dependent kinases during Fas-induced apoptosis in Jurkat cells. *Proceedings of the National Academy of Sciences of the United States of America*, 95(12):6785–6790, June 1998.

[301] Robert Lipski, Daniel J. Lippincott, Brittany C. Durden, Anne R. Kaplan, Hilary E. Keiser, Jung-Ho Park, and Aime A. Levesque. P53 Dimers associate with a head-to-tail response element to repress cyclin B transcription. *PloS One*, 7(8):e42615, 2012.

[302] B. Alvarez, C. Martínez-A, B. M. Burgering, and A. C. Carrera. Forkhead transcription factors contribute to execution of the mitotic programme in mammals. *Nature*, 413(6857):744–747, October 2001.

[303] T. W. Leung, S. S. Lin, A. C. Tsang, C. S. Tong, J. C. Ching, W. Y. Leung, R. Gimlich, G. G. Wong, and K. M. Yao. Over-expression of FoxM1 stimulates cyclin B1 expression. *FEBS letters*, 507(1):59–66, October 2001.

[304] I.-Ching Wang, Yi-Ju Chen, Douglas Hughes, Vladimir Petrovic, Michael L. Major, Hyung Jung Park, Yongjun Tan, Timothy Ackerson, and Robert H. Costa. Forkhead box M1 regulates the transcriptional network of genes essential for mitotic progression and genes encoding the SCF (Skp2-Cks1) ubiquitin ligase. *Molecular and Cellular Biology*, 25(24):10875–10894, December 2005.

[305] Stéphanie Dutertre, Martine Cazales, Muriel Quaranta, Carine Froment, Valerie Trabut, Christine Dozier, Gladys Mirey, Jean-Pierre Bouché, Nathalie Theis-Febvre, Estelle Schmitt, Bernard Monsarrat, Claude Prigent, and Bernard Ducommun. Phosphorylation of CDC25B by Aurora-A at the centrosome contributes to the G2-M transition. *Journal of Cell Science*, 117(Pt 12):2523–2531, May 2004.

[306] Stéphanie Dutertre, Simon Descamps, and Claude Prigent. On the role of aurora-A in centrosome function. *Oncogene*, 21(40):6175–6183, September 2002.

[307] Lilia Gheghiani, Damarys Loew, Bérangère Lombard, Jörg Mansfeld, and Olivier Gavet. PLK1 Activation in Late G2 Sets Up Commitment to Mitosis. *Cell Reports*, 19(10):2060–2073, June 2017.

[308] Catherine Lindon and Jonathon Pines. Ordered proteolysis in anaphase inactivates Plk1 to contribute to proper mitotic exit in human cells. *The Journal of Cell Biology*, 164(2):233–241, January 2004.

[309] S. Kotani, S. Tugendreich, M. Fujii, P. M. Jorgensen, N. Watanabe, C. Hoog, P. Hieter, and K. Todokoro. PKA and MPF-activated polo-like kinase regulate anaphase-promoting complex activity and mitosis progression. *Molecular Cell*, 1(3):371–380, February 1998.

[310] Y. W. Qian, E. Erikson, C. Li, and J. L. Maller. Activated polo-like kinase Plx1 is required at multiple points during mitosis in Xenopus laevis. *Molecular and Cellular Biology*, 18(7):4262–4271, July 1998.

[311] Yann Thomas, Luca Cirillo, Costanza Panbianco, Lisa Martino, Nicolas Tavernier, Françoise Schwager, Lucie Van Hove, Nicolas Joly, Anna Santamaria, Lionel Pintard, and Monica Gotta. Cdk1 Phosphorylates SPAT-1/Bora to Promote Plk1 Activation in C. elegans and Human Cells. *Cell Reports*, 15(3):510–518, April 2016.

[312] Oleg Timofeev, Onur Cizmecioglu, Entan Hu, Thomas Orlik, and Ingrid Hoffmann. Human Cdc25A phosphatase has a non-redundant function in G2 phase by activating Cyclin A-dependent kinases. *FEBS letters*, 583(4):841–847, February 2009.

[313] Kai Liu, Minying Zheng, Rui Lu, Jiaxing Du, Qi Zhao, Zugui Li, Yuwei Li, and Shiwu Zhang. The role of CDC25C in cell cycle regulation and clinical cancer therapy: A systematic review. *Cancer Cell International*, 20:213, 2020.

[314] Maria Thanasoula, Jose Miguel Escandell, Natsuko Suwaki, and Madalena Tarsounas. ATM/ATR checkpoint activation downregulates CDC25C to prevent mitotic entry with uncapped telomeres. *The EMBO journal*, 31(16):3398–3410, August 2012.

[315] Estelle Schmitt, Rose Boutros, Carine Froment, Bernard Monsarrat, Bernard Ducommun, and Christine Dozier. CHK1 phosphorylates CDC25B during the cell cycle in the absence of DNA damage. *Journal of Cell Science*, 119(Pt 20):4269–4275, October 2006.

[316] Alwin Krämer, Niels Mailand, Claudia Lukas, Randi G. Syljuåsen, Christopher J. Wilkinson, Erich A. Nigg, Jiri Bartek, and Jiri Lukas. Centrosome-associated Chk1 prevents premature activation of cyclin-B-Cdk1 kinase. *Nature Cell Biology*, 6(9):884–891, September 2004.

[317] Valerie Lobjois, Denis Jullien, Jean-Pierre Bouché, and Bernard Ducommun. The polo-like kinase 1 regulates CDC25B-dependent mitosis entry. *Biochimica Et Biophysica Acta*, 1793(3):462–468, March 2009.

[318] Arne Lindqvist, Helena Källström, Andreas Lundgren, Emad Barsoum, and Christina Karlsson Rosenthal. Cdc25B cooperates with Cdc25A to induce mitosis but has a unique role in activating cyclin B1-Cdk1 at the centrosome. *The Journal of Cell Biology*, 171(1):35–45, October 2005.

[319] A. Lopez-Girona, B. Furnari, O. Mondesert, and P. Russell. Nuclear localization of Cdc25 is regulated by DNA damage and a 14-3-3 protein. *Nature*, 397(6715):172–175, January 1999.

[320] B. Ouyang, W. Li, H. Pan, J. Meadows, I. Hoffmann, and W. Dai. The physical association and phosphorylation of Cdc25C protein phosphatase by Prk. *Oncogene*, 18(44):6029–6036, October 1999.

[321] J. P. Cogswell, C. E. Brown, J. E. Bisi, and S. D. Neill. Dominant-negative polo-like kinase 1 induces mitotic catastrophe independent of cdc25C function. *Cell Growth & Differentiation: The Molecular Biology Journal of the American Association for Cancer Research*, 11(12):615–623, December 2000.

[322] C. P. De Souza, K. A. Ellem, and B. G. Gabrielli. Centrosomal and cytoplasmic Cdc2/cyclin B1 activation precedes nuclear mitotic events. *Experimental Cell Research*, 257(1):11–21, May 2000.

[323] Mark Jackman, Catherine Lindon, Erich A. Nigg, and Jonathon Pines. Active cyclin B1-Cdk1 first appears on centrosomes in prophase. *Nature Cell Biology*, 5(2):143–148, February 2003.

[324] Z. Shang, L. Yu, Y.-F. Lin, S. Matsunaga, C.-Y. Shen, and B. P. C. Chen. DNA-PKcs activates the Chk2-Brca1 pathway during mitosis to ensure chromosomal stability. *Oncogenesis*, 3(2):e85, February 2014.

[325] I Hoffmann, PR Clarke, M Jessus Marcote, E Karsenti, and G Draetta. Phosphorylation and activation of human cdc25-C by cdc2–cyclin B and its involvement in the self-amplification of MPF at mitosis. *The EMBO journal*, 12(1):53, 1993.

[326] A Karaskou, X Cayla, O Haccard, C Jessus, and R Ozon. MPF amplification in Xenopus oocyte extracts depends on a two-step activation of cdc25 phosphatase. *Experimental Cell Research*, 244(2):491–500, November 1998.

[327] MR Jackman and JN Pines. Cyclins and the G2/M transition. *Cancer surveys*, 29:47–73, 1996.

[328] Ruopeng Zhang, Li Zhu, Lirong Zhang, Anli Xu, Zhengwei Li, Yijuan Xu, Pei He, Maoqing Wu, Fengxiang Wei, and Chenhong Wang. PTEN enhances G2/M arrest in etoposide-treated MCF-7 cells through activation of the ATM pathway. *Oncology Reports*, 35(5):2707–2714, May 2016.

[329] R Heald, M McLoughlin, and F McKeon. Human wee1 maintains mitotic timing by protecting the nucleus from cytoplasmically activated Cdc2 kinase. *Cell*, 74(3):463–474, August 1993.

[330] Adam D Rudner and Andrew W Murray. Phosphorylation by Cdc28 activates the Cdc20-dependent activity of the anaphase-promoting complex. *The Journal of cell biology*, 149(7):1377–1390, 2000.

[331] Xinxian Qiao, Liyong Zhang, Armin M Gamper, Takeo Fujita, and Yong Wan. APC/C-Cdh1: From cell cycle to cellular differentiation and genomic integrity. *Cell Cycle*, 9(19):3904–3912, October 2010.

[332] Amnon Golan, Yana Yudkovsky, and Avram Hershko. The cyclin-ubiquitin ligase activity of cyclosome/APC is jointly activated by protein kinases Cdk1-cyclin B and Plk. *The Journal of Biological Chemistry*, 277(18):15552–15557, May 2002.

[333] Vincenzo D'Angiolella, Cecilia Mari, Donatella Nocera, Linda Rametti, and Domenico Grieco. The spindle checkpoint requires cyclin-dependent kinase activity. *Genes & Development*, 17(20):2520–2525, October 2003.

[334] Brian R Thornton and David P Toczyski. Precise destruction: An emerging picture of the APC. *Genes & development*, 20(22):3069–3078, 2006.

[335] Jan-Michael Peters. The anaphase promoting complex/cyclosome: A machine designed to destroy. *Nature reviews Molecular cell biology*, 7(9):644–656, 2006.

[336] Luigi Nezi and Andrea Musacchio. Sister chromatid tension and the spindle assembly checkpoint. *Current opinion in cell biology*, 21(6):785–795, December 2009.

[337] S K Reddy, M Rape, W A Margansky, and M W Kirschner. Ubiquitination by the anaphase-promoting complex drives spindle checkpoint inactivation. *Nature*, 446(7138):921–925, April 2007.

[338] John Maciejowski, Kelly A. George, Marie-Emilie Terret, Chao Zhang, Kevan M. Shokat, and Prasad V. Jallepalli. Mps1 directs the assembly of Cdc20 inhibitory complexes during interphase and mitosis to control M phase timing and spindle checkpoint signaling. *The Journal of Cell Biology*, 190(1):89–100, July 2010.

[339] Jamin B. Hein and Jakob Nilsson. Interphase APC/C-Cdc20 inhibition by cyclin A2-Cdk2 ensures efficient mitotic entry. *Nature Communications*, 7:10975, March 2016.

[340] Speranta Avram, Maria Mernea, Dan Florin Mihailescu, Corina Duda Seiman, Daniel Duda Seiman, and Mihai Viorel Putz. Mitotic checkpoint proteins Mad1 and Mad2 - structural and functional relationship with implication in genetic diseases. *Current Computer-Aided Drug Design*, 10(2):168–181, 2014.

[341] Weiping Wang and Marc W. Kirschner. Emi1 preferentially inhibits ubiquitin chain elongation by the anaphase-promoting complex. *Nature Cell Biology*, 15(7):797–806, July 2013.

[342] Michalis Fragkos, Olivier Ganier, Philippe Coulombe, and Marcel Méchali. DNA replication origin activation in space and time. *Nature Reviews Molecular Cell Biology*, 16(6):360–374, June 2015.

[343] Bing Ren, Hieu Cam, Yasuhiko Takahashi, Thomas Volkert, Jolyon Terragni, Richard A. Young, and Brian David Dynlacht. E2F integrates cell cycle progression with DNA repair, replication, and G(2)/M checkpoints. *Genes & Development*, 16(2):245–256, January 2002.

[344] Hugh Cam and Brian David Dynlacht. Emerging roles for E2F: Beyond the G1/S transition and DNA replication. *Cancer Cell*, 3(4):311–316, April 2003.

[345] Dawn Coverley, Heike Laman, and Ronald A Laskey. Distinct roles for cyclins E and A during DNA replication complex assembly and activation. *Nature Cell Biology*, 4(7):523–528, 2002.

[346] Niels Mailand, Jacob Falck, Claudia Lukas, Randi G Syljuåsen, Markus Welcker, Jiri Bartek, and Jiri Lukas. Rapid destruction of human Cdc25A in response to DNA damage. *Science*, 288(5470):1425–1429, 2000.

[347] Maddalena Donzelli and Giulio F Draetta. Regulating mammalian checkpoints through Cdc25 inactivation. *EMBO reports*, 4(7):671–677, July 2003.

[348] Eva Petermann, Apolinar Maya-Mendoza, George Zachos, David A F Gillespie, Dean A Jackson, and Keith W Caldecott. Chk1 requirement for high global rates of replication fork progression during normal vertebrate S phase. *Molecular and Cellular Biology*, 26(8):3319–3326, April 2006.

[349] Upadhyayula Sai Srinivas, Bryce W. Q. Tan, Balamurugan A. Vellayappan, and Anand D. Jeyasekharan. ROS and the DNA damage response in cancer. *Redox Biology*, 25:101084, July 2019.

[350] Yunxiao Meng, Chi-Wei Chen, Mingo M. H. Yung, Wei Sun, Jing Sun, Zhuqing Li, Jing Li, Zongzhu Li, Wei Zhou, Stephanie S. Liu, Annie N. Y. Cheung, Hextan Y. S. Ngan, John C. Braisted, Yan Kai, Weiqun Peng, Alexandros Tzatsos, Yiliang Li, Zhijun Dai, Wei Zheng, David W. Chan, and Wenge Zhu. DUOXA1-mediated ROS production promotes cisplatin resistance by activating ATR-Chk1 pathway in ovarian cancer. *Cancer Letters*, 428:104–116, August 2018.

[351] Eric Batchelor, Alexander Loewer, Caroline Mock, and Galit Lahav. Stimulus-dependent dynamics of p53 in single cells. *Molecular Systems Biology*, 7(1):488, January 2011.

[352] Ae Jeong Kim, Hyun-Ju Kim, Hye Jin Jee, Naree Song, Minjee Kim, Yoe-Sik Bae, Jay H. Chung, and Jeanho Yun. Glucose deprivation is associated with Chk1 degradation through the ubiquitin-proteasome pathway and effective checkpoint response to replication blocks. *Biochimica Et Biophysica Acta*, 1813(6):1230–1238, June 2011.

[353] Soyeon Jeong, Jinsook Ahn, Inseong Jo, So-Mi Kang, Bum-Joon Park, Hyun-Soo Cho, Yong-Hak Kim, and Nam-Chul Ha. Cyclin-dependent kinase 1 depolymerizes nuclear lamin filaments by disrupting the head-to-tail interaction of the lamin central rod domain. *The Journal of Biological Chemistry*, 298(9):102256, September 2022.

[354] Mayumi Kitagawa and Sang Hyun Lee. The chromosomal passenger complex (CPC) as a key orchestrator of orderly mitotic exit and cytokinesis. *Frontiers in Cell and Developmental Biology*, 3:14, 2015.

[355] Evanthia Pangou and Izabela Sumara. The Multifaceted Regulation of Mitochondrial Dynamics During Mitosis. *Frontiers in Cell and Developmental Biology*, 9:767221, 2021.

[356] F. Uhlmann, F. Lottspeich, and K. Nasmyth. Sister-chromatid separation at anaphase onset is promoted by cleavage of the cohesin subunit Scc1. *Nature*, 400(6739):37–42, July 1999.

[357] Tamar Listovsky and Julian E. Sale. Sequestration of CDH1 by MAD2L2 prevents premature APC/C activation prior to anaphase onset. *The Journal of Cell Biology*, 203(1):87–100, October 2013.

[358] Irma Sánchez and Brian David Dynlacht. New insights into cyclins, CDKs, and cell cycle control. *Seminars in Cell & Developmental Biology*, 16(3):311–321, June 2005.

[359] Angela Flavia Serpico, Francesco Febbraro, Caterina Pisauro, and Domenico Grieco. Compartmentalized control of Cdk1 drives mitotic spindle assembly. *Cell Reports*, 38(4):110305, January 2022.

[360] Wei Qian, Serah Choi, Gregory A. Gibson, Simon C. Watkins, Christopher J. Bakkenist, and Bennett Van Houten. Mitochondrial hyperfusion induced by loss of the fission protein Drp1 causes ATM-dependent G2/M arrest and aneuploidy through DNA replication stress. *Journal of Cell Science*, 125(Pt 23):5745–5757, December 2012.

[361] Xinxian Qiao, Liyong Zhang, Armin M Gamper, Takeo Fujita, and Yong Wan. APC/C-Cdh1: From cell cycle to cellular differentiation and genomic integrity. *Cell Cycle*, 9(19):3904–3912, October 2010.

[362] Violeta Morin, Susana Prieto, Sabrina Melines, Sonia Hem, Michel Rossignol, Thierry Lorca, Julien Espeut, Nathalie Morin, and Ariane Abrieu. CDK-dependent potentiation of MPS1 kinase activity is essential to the mitotic checkpoint. *Current biology: CB*, 22(4):289–295, February 2012.

[363] Aaron R. Tipton, Wenbin Ji, Brianne Sturt-Gillespie, Michael E. Bekier, Kexi Wang, William R. Taylor, and Song-Tao Liu. Monopolar spindle 1 (MPS1) kinase promotes production of closed MAD2 (C-MAD2) conformer and assembly of the mitotic checkpoint complex. *The Journal of Biological Chemistry*, 288(49):35149–35158, December 2013.

[364] Mark Petronczki, Péter Lénárt, and Jan-Michael Peters. Polo on the Rise-from Mitotic Entry to Cytokinesis with Plk1. *Developmental Cell*, 14(5):646–659, May 2008.

[365] Travis L Schmit, Weixiong Zhong, Vijayasaradhi Setaluri, Vladimir S Spiegelman, and Nihal Ahmad. Targeted depletion of Polo-like kinase (Plk) 1 through lentiviral shRNA or a small-molecule inhibitor causes mitotic catastrophe and induction of apoptosis in human melanoma cells. *The Journal of investigative dermatology*, 129(12):2843–2853, December 2009.

[366] Andrea Vecchione, Gustavo Baldassarre, Hideshi Ishii, Milena S. Nicoloso, Barbara Belletti, Fabio Petrocca, Nicola Zanesi, Louise Y. Y. Fong, Sabrina Battista, Daniela Guarnieri, Raffaele Baffa, Hansjuerg Alder, John L. Farber, Peter J. Donovan, and Carlo M. Croce. Fez1/Lzts1 absence impairs Cdk1/Cdc25C interaction during mitosis and predisposes mice to cancer development. *Cancer Cell*, 11(3):275–289, March 2007.

[367] Mark Petronczki, Michael Glotzer, Norbert Kraut, and Jan-Michael Peters. Polo-like kinase 1 triggers the initiation of cytokinesis in human cells by promoting recruitment of the RhoGEF Ect2 to the central spindle. *Developmental Cell*, 12(5):713–725, May 2007.

[368] Zengqiang Yuan, Esther B E Becker, Paola Merlo, Tomoko Yamada, Sara DiBacco, Yoshiyuki Konishi, Erik M Schaefer, and Azad Bonni. Activation of FOXO1 by Cdk1 in cycling cells and postmitotic neurons. *Science (New York, NY)*, 319(5870):1665–1668, March 2008.

[369] Benjamin A. Wolfe, Tohru Takaki, Mark Petronczki, and Michael Glotzer. Polo-like kinase 1 directs assembly of the HsCyk-4 RhoGAP/Ect2 RhoGEF complex to initiate cleavage furrow formation. *PLoS biology*, 7(5):e1000110, May 2009.

[370] Suzanne Floyd, Jonathon Pines, and Catherine Lindon. APC/C Cdh1 targets aurora kinase to control reorganization of the mitotic spindle at anaphase. *Current biology: CB*, 18(21):1649–1658, November 2008.

[371] Patricia Wadsworth. The multifunctional spindle midzone in vertebrate cells at a glance. *Journal of Cell Science*, 134(10):jcs250001, May 2021.

[372] Herbert Sizek, Andrew Hamel, Dávid Deritei, Sarah Campbell, and Erzsébet Ravasz Regan. Boolean model of growth signaling, cell cycle and apoptosis predicts the molecular mechanism of aberrant cell cycle progression driven by hyperactive PI3K. *PLoS computational biology*, 15(3):e1006402, March 2019.

[373] Eric Guberman, Hikmet Sherief, and Erzsébet Ravasz Regan. Boolean model of anchorage dependence and contact inhibition points to coordinated inhibition but semi-independent induction of proliferation and migration. *Computational and Structural Biotechnology Journal*, 18:2145–2165, 2020.

[374] Mark E. Burkard, Catherine L. Randall, Stéphane Larochelle, Chao Zhang, Kevan M. Shokat, Robert P. Fisher, and Prasad V. Jallepalli. Chemical genetics reveals the requirement for Polo-like kinase 1 activity in positioning RhoA and triggering cytokinesis in human cells. *Proceedings of the National Academy of Sciences of the United States of America*, 104(11):4383–4388, March 2007.

[375] Yi Ren and Han-Ming Shen. Critical role of AMPK in redox regulation under glucose starvation. *Redox Biology*, 25:101154, July 2019.

[376] Zhi Guo, Sergei Kozlov, Martin F. Lavin, Maria D. Person, and Tanya T. Paull. ATM activation by oxidative stress. *Science (New York, N.Y.)*, 330(6003):517–521, October 2010.

[377] Brian D. Larsen and Claus S. Sørensen. The caspase-activated DNase: Apoptosis and beyond. *The FEBS journal*, 284(8):1160–1170, April 2017.

[378] Antonina Hafner, Jacob Stewart-Ornstein, Jeremy E. Purvis, William C. Forrester, Martha L. Bulyk, and Galit Lahav. P53 pulses lead to distinct patterns of gene expression albeit similar DNA-binding dynamics. *Nature Structural & Molecular Biology*, 24(10):840–847, October 2017.

[379] José Reyes, Jia-Yun Chen, Jacob Stewart-Ornstein, Kyle W. Karhohs, Caroline S. Mock, and Galit Lahav. Fluctuations in p53 Signaling Allow Escape from Cell-Cycle Arrest. *Molecular Cell*, 71(4):581–591.e5, August 2018.

[380] A. Tritarelli, E. Oricchio, M. Ciciarello, R. Mangiacasale, A. Palena, P. Lavia, S. Soddu, and E. Cundari. P53 localization at centrosomes during mitosis and postmitotic checkpoint are ATM-dependent and require serine 15 phosphorylation. *Molecular Biology of the Cell*, 15(8):3751–3757, August 2004.

[381] Russell G. Jones, David R. Plas, Sara Kubek, Monica Buzzai, James Mu, Yang Xu, Morris J. Birnbaum, and Craig B. Thompson. AMP-activated protein kinase induces a p53-dependent metabolic checkpoint. *Molecular Cell*, 18(3):283–293, April 2005.

[382] N. H. Chehab, A. Malikzay, M. Appel, and T. D. Halazonetis. Chk2/hCds1 functions as a DNA damage checkpoint in G(1) by stabilizing p53. *Genes & Development*, 14(3):278–288, February 2000.

[383] X. Wu, J. H. Bayle, D. Olson, and A. J. Levine. The p53-mdm-2 autoregulatory feedback loop. *Genes & Development*, 7(7A):1126–1132, July 1993.

[384] Y. Haupt, R. Maya, A. Kazaz, and M. Oren. Mdm2 promotes the rapid degradation of p53. *Nature*, 387(6630):296–299, May 1997.

[385] M. H. Kubbutat, S. N. Jones, and K. H. Vousden. Regulation of p53 stability by Mdm2. *Nature*, 387(6630):299–303, May 1997.

[386] Tao Shi, Daan M. K. van Soest, Paulien E. Polderman, Boudewijn M. T. Burgering, and Tobias B. Dansen. DNA damage and oxidant stress activate p53 through differential upstream signaling pathways. *Free Radical Biology & Medicine*, 172:298–311, August 2021.

[387] SiDe Li, Michaela Banck, Shiraz Mujtaba, Ming-Ming Zhou, Mary M. Sugrue, and Martin J. Walsh. P53-induced growth arrest is regulated by the mitochondrial SirT3 deacetylase. *PloS One*, 5(5):e10486, May 2010.

[388] S. Y. Shieh, J. Ahn, K. Tamai, Y. Taya, and C. Prives. The human homologs of checkpoint kinases Chk1 and Cds1 (Chk2) phosphorylate p53 at multiple DNA damage-inducible sites. *Genes & Development*, 14(3):289–300, February 2000.

[389] Y. Barak, T. Juven, R. Haffner, and M. Oren. Mdm2 expression is induced by wild type p53 activity. *The EMBO journal*, 12(2):461–468, February 1993.

[390] Loretah Chibaya, Baktiar Karim, Hong Zhang, and Stephen N. Jones. Mdm2 phosphorylation by Akt regulates the p53 response to oxidative stress to promote cell proliferation and tumorigenesis. *Proceedings of the National Academy of Sciences of the United States of America*, 118(4):e2003193118, January 2021.

[391] Trudy G. Oliver, Etienne Meylan, Gregory P. Chang, Wen Xue, James R. Burke, Timothy J. Humpton, Diana Hubbard, Arjun Bhutkar, and Tyler Jacks. Caspase-2-mediated cleavage of Mdm2 creates a p53-induced positive feedback loop. *Molecular Cell*, 43(1):57–71, July 2011.

[392] Koh Katsuda, Masafumi Kataoka, Futoshi Uno, Takayoshi Murakami, Tadashi Kondo, Jack A. Roth, Noriaki Tanaka, and Toshiyoshi Fujiwara. Activation of caspase-3 and cleavage of Rb are associated with p16-mediated apoptosis in human non-small cell lung cancer cells. *Oncogene*, 21(13):2108–2113, March 2002.

[393] Alexander Loewer, Eric Batchelor, Giorgio Gaglia, and Galit Lahav. Basal dynamics of p53 reveal transcriptionally attenuated pulses in cycling cells. *Cell*, 142(1):89–100, July 2010.

[394] Maria Eugenia Guicciardi and Gregory J Gores. Life and death by death receptors. *The FASEB Journal*, 23(6):1625–1637, June 2009.

[395] Bodvaël Pennarun, Annemieke Meijer, Elisabeth G. E. de Vries, Jan H. Kleibeuker, Frank Kruyt, and Steven de Jong. Playing the DISC: Turning on TRAIL death receptor-mediated apoptosis in cancer. *Biochimica Et Biophysica Acta*, 1805(2):123–140, April 2010.

[396] E. A. Slee, M. T. Harte, R. M. Kluck, B. B. Wolf, C. A. Casiano, D. D. Newmeyer, H. G. Wang, J. C. Reed, D. W. Nicholson, E. S. Alnemri, D. R. Green, and S. J. Martin. Ordering the cytochrome c-initiated caspase cascade: Hierarchical activation of caspases-2, -3, -6, -7, -8, and -10 in a caspase-9-dependent manner. *The Journal of Cell Biology*, 144(2):281–292, January 1999.

[397] V. Cowling and J. Downward. Caspase-6 is the direct activator of caspase-8 in the cytochrome c-induced apoptosis pathway: Absolute requirement for removal of caspase-6 prodomain. *Cell Death and Differentiation*, 9(10):1046–1056, October 2002.

[398] Richa B. Shah, Ruth Thompson, and Samuel Sidi. A mitosis-sensing caspase activation platform? New insights into the PIDDosome. *Molecular & Cellular Oncology*, 3(3):e1059921, May 2016.

[399] Celia Vogel, Anne Kienitz, Rolf Müller, and Holger Bastians. The mitotic spindle checkpoint is a critical determinant for topoisomerase-based chemotherapy. *The Journal of Biological Chemistry*, 280(6):4025–4028, February 2005.

[400] Akira Masuda, Ken Maeno, Taku Nakagawa, Hiroko Saito, and Takashi Takahashi. Association between mitotic spindle checkpoint impairment and susceptibility to the induction of apoptosis by anti-microtubule agents in human lung cancers. *The American Journal of Pathology*, 163(3):1109–1116, September 2003.

[401] Joshua L Andersen, Carrie E Johnson, Christopher D Freel, Amanda B Parrish, Jennifer L Day, Marisa R Buchakjian, Leta K Nutt, J Will Thompson, M Arthur Moseley, and Sally Kornbluth. Restraint of apoptosis during mitosis through interdomain phosphorylation of caspase-2. *The EMBO Journal*, 28(20):3216–3227, October 2009.

[402] Ilio Vitale, Gwenola Manic, Maria Castedo, and Guido Kroemer. Caspase 2 in mitotic catastrophe: The terminator of aneuploid and tetraploid cells. *Molecular & Cellular Oncology*, 4(3):e1299274, 2017.

[403] M. Kimura, T. Yoshioka, M. Saio, Y. Banno, H. Nagaoka, and Y. Okano. Mitotic catastrophe and cell death induced by depletion of centrosomal proteins. *Cell Death & Disease*, 4(4):e603, April 2013.

[404] H. Li, L. Bergeron, V. Cryns, M. S. Pasternack, H. Zhu, L. Shi, A. Greenberg, and J. Yuan. Activation of caspase-2 in apoptosis. *The Journal of Biological Chemistry*, 272(34):21010–21017, August 1997.

[405] E. A. Slee, C. Adrain, and S. J. Martin. Executioner caspase-3, -6, and -7 perform distinct, non-redundant roles during the demolition phase of apoptosis. *The Journal of Biological Chemistry*, 276(10):7320–7326, March 2001.

[406] Ulrich Maurer, Céline Charvet, Allan S Wagman, Emmanuel Dejardin, and Douglas R Green. Glycogen synthase kinase-3 regulates mitochondrial outer membrane permeabilization and apoptosis by destabilization of MCL-1. *Mol Cell*, 21(6):749–760, March 2006.

[407] Ingrid E. Wertz, Saritha Kusam, Cynthia Lam, Toru Okamoto, Wendy Sandoval, Daniel J. Anderson, Elizabeth Helgason, James A. Ernst, Mike Eby, Jinfeng Liu, Lisa D. Belmont, Josh S. Kaminker, Karen M. O'Rourke, Kanan Pujara, Pawan Bir Kohli, Adam R. Johnson, Mark L. Chiu, Jennie R. Lill, Peter K. Jackson, Wayne J. Fairbrother, Somasekar Seshagiri, Mary J. C. Ludlam, Kevin G. Leong, Erin C. Dueber, Heather Maecker, David C. S. Huang, and Vishva M. Dixit. Sensitivity to antitubulin chemotherapeutics is regulated by MCL1 and FBW7. *Nature*, 471(7336):110–114, March 2011.

[408] Eun-Sil Sung, Kyung-Jin Park, Hye-Ji Choi, Chul-Ho Kim, and Yong-Sung Kim. The proteasome inhibitor MG132 potentiates TRAIL receptor agonist-induced apoptosis by stabilizing tBid and Bik in human head and neck squamous cell carcinoma cells. *Experimental Cell Research*, 318(13):1564–1576, August 2012.

[409] Chiou-Feng Lin, Cheng-Chieh Tsai, Wei-Ching Huang, Yu-Chih Wang, Po-Chun Tseng, Tsung-Ting Tsai, and Chia-Ling Chen. Glycogen Synthase Kinase-3β and Caspase-2 Mediate Ceramide- and Etoposide-Induced Apoptosis by Regulating the Lysosomal-Mitochondrial Axis. *PloS One*, 11(1):e0145460, 2016.

[410] Qingqing Ding, Longfei Huo, Jer-Yen Yang, Weiya Xia, Yongkun Wei, Yong Liao, Chun-Ju Chang, Yan Yang, Chien-Chen Lai, Dung-Fang Lee, Chia-Jui Yen, Yun-Ju Rita Chen, Jung-Mao Hsu, Hsu-Ping Kuo, Chun-Yi Lin, Fuu-Jen Tsai, Long-Yuan Li, Chang-Hai Tsai, and Mien-Chie Hung. Down-regulation of myeloid cell leukemia-1 through inhibiting Erk/Pin 1 pathway by sorafenib facilitates chemosensitization in breast cancer. *Cancer Research*, 68(15):6109–6117, August 2008.

[411] K. J. Townsend, J. L. Trusty, M. A. Traupman, A. Eastman, and R. W. Craig. Expression of the antiapoptotic MCL1 gene product is regulated by a mitogen activated protein kinase-mediated pathway triggered through microtubule disruption and protein kinase C. *Oncogene*, 17(10):1223–1234, September 1998.

[412] Rhonda Croxton, Yihong Ma, Lanxi Song, Eric B Haura, and W Douglas Cress. Direct repression of the Mcl-1 promoter by E2F1. *Oncogene*, 21(9):1359–1369, February 2002.

[413] Margaret E. Harley, Lindsey A. Allan, Helen S. Sanderson, and Paul R. Clarke. Phosphorylation of Mcl-1 by CDK1-cyclin B1 initiates its Cdc20-dependent destruction during mitotic arrest. *The EMBO journal*, 29(14):2407–2420, July 2010.

[414] Lilly Magdalena Weiß, Manuela Hugle, Sarah Romero, and Simone Fulda. Synergistic induction of apoptosis by a polo-like kinase 1 inhibitor and microtubule-interfering drugs in Ewing sarcoma cells. *International Journal of Cancer*, 138(2):497–506, January 2016.

[415] David T. Terrano, Meenakshi Upreti, and Timothy C. Chambers. Cyclin-dependent kinase 1-mediated Bcl-xL/Bcl-2 phosphorylation acts as a functional link coupling mitotic arrest and apoptosis. *Molecular and Cellular Biology*, 30(3):640–656, February 2010.

[416] Lingli Zhou, Xiaoling Cai, Xueyao Han, Naihan Xu, and Donald C. Chang. CDK1 switches mitotic arrest to apoptosis by phosphorylating Bcl-2/Bax family proteins during treatment with microtubule interfering agents. *Cell Biology International*, 38(6):737–746, June 2014.

[417] N. Bah, L. Maillet, J. Ryan, S. Dubreil, F. Gautier, A. Letai, P. Juin, and S. Barillé-Nion. Bcl-xL controls a switch between cell death modes during mitotic arrest. *Cell Death & Disease*, 5:e1291, June 2014.

[418] Céline Gélinas and Eileen White. BH3-only proteins in control: Specificity regulates MCL-1 and BAK-mediated apoptosis. *Genes & Development*, 19(11):1263–1268, June 2005.

[419] E. Yang, J. Zha, J. Jockel, L. H. Boise, C. B. Thompson, and S. J. Korsmeyer. Bad, a heterodimeric partner for Bcl-XL and Bcl-2, displaces Bax and promotes cell death. *Cell*, 80(2):285–291, January 1995.

[420] D. G. Kirsch, A. Doseff, B. N. Chau, D. S. Lim, N. C. de Souza-Pinto, R. Hansford, M. B. Kastan, Y. A. Lazebnik, and J. M. Hardwick. Caspase-3-dependent cleavage of Bcl-2 promotes release of cytochrome c. *The Journal of Biological Chemistry*, 274(30):21155–21161, July 1999.

[421] Xinrui Zhao, Lidong Liu, Rui Li, Xuan Wei, Wenqing Luan, Peishu Liu, and Jing Zhao. Hypoxia-Inducible Factor 1-α (HIF-1α) Induces Apoptosis of Human Uterosacral Ligament Fibroblasts Through the Death Receptor and Mitochondrial Pathways. *Medical Science Monitor: International Medical Journal of Experimental and Clinical Research*, 24:8722–8733, December 2018.

[422] L. O'Connor, A. Strasser, L. A. O'Reilly, G. Hausmann, J. M. Adams, S. Cory, and D. C. Huang. Bim: A novel member of the Bcl-2 family that promotes apoptosis. *The EMBO journal*, 17(2):384–395, January 1998.

[423] B. Elangovan and G. Chinnadurai. Functional dissection of the pro-apoptotic protein Bik. Heterodimerization with anti-apoptosis proteins is insufficient for induction of cell death. *The Journal of Biological Chemistry*, 272(39):24494–24498, September 1997.

[424] X. Luo, I. Budihardjo, H. Zou, C. Slaughter, and X. Wang. Bid, a Bcl2 interacting protein, mediates cytochrome c release from mitochondria in response to activation of cell surface death receptors. *Cell*, 94(4):481–490, August 1998.

[425] F. Condorelli, P. Salomoni, S. Cotteret, V. Cesi, S. M. Srinivasula, E. S. Alnemri, and B. Calabretta. Caspase cleavage enhances the apoptosis-inducing effects of BAD. *Molecular and Cellular Biology*, 21(9):3025–3036, May 2001.

[426] S R Datta, H Dudek, X Tao, S Masters, H Fu, Y Gotoh, and M E Greenberg. Akt phosphorylation of BAD couples survival signals to the cell-intrinsic death machinery. *Cell*, 91(2):231–241, October 1997.

[427] X. Fang, S. Yu, A. Eder, M. Mao, R. C. Bast, D. Boyd, and G. B. Mills. Regulation of BAD phosphorylation at serine 112 by the Ras-mitogen-activated protein kinase pathway. *Oncogene*, 18(48):6635–6640, November 1999.

[428] Y. Tan, M. R. Demeter, H. Ruan, and M. J. Comb. BAD Ser-155 phosphorylation regulates BAD/Bcl-XL interaction and cell survival. *The Journal of Biological Chemistry*, 275(33):25865–25869, August 2000.

[429] Agshin F. Taghiyev, Natalya V. Guseva, Hisashi Harada, C. Michael Knudson, Oskar W. Rokhlin, and Michael B. Cohen. Overexpression of BAD potentiates sensitivity to tumor necrosis factor-related apoptosis-inducing ligand treatment in the prostatic carcinoma cell line LNCaP. *Molecular cancer research: MCR*, 1(7):500–507, May 2003.

[430] Bernhard Gillissen, Frank Essmann, Philipp G. Hemmati, Antje Richter, Anja Richter, Ilker Oztop, Govindaswamy Chinnadurai, Bernd Dörken, and Peter T. Daniel. Mcl-1 determines the Bax dependency of Nbk/Bik-induced apoptosis. *The Journal of Cell Biology*, 179(4):701–715, November 2007.

[431] J. M. Boyd, G. J. Gallo, B. Elangovan, A. B. Houghton, S. Malstrom, B. J. Avery, R. G. Ebb, T. Subramanian, T. Chittenden, and R. J. Lutz. Bik, a novel death-inducing protein shares a distinct sequence motif with Bcl-2 family proteins and interacts with viral and cellular survival-promoting proteins. *Oncogene*, 11(9):1921–1928, November 1995.

[432] P. F. Dijkers, R. H. Medema, J. W. Lammers, L. Koenderman, and P. J. Coffer. Expression of the pro-apoptotic Bcl-2 family member Bim is regulated by the forkhead transcription factor FKHR-L1. *Current biology: CB*, 10(19):1201–1204, October 2000.

[433] Vesa Hongisto, Nina Smeds, Stephan Brecht, Thomas Herdegen, Michael J. Courtney, and Eleanor T. Coffey. Lithium blocks the c-Jun stress response and protects neurons via its action on glycogen synthase kinase 3. *Molecular and Cellular Biology*, 23(17):6027–6036, September 2003.

[434] Rosie Hughes, Jonathan Gilley, Mark Kristiansen, and Jonathan Ham. The MEK-ERK pathway negatively regulates bim expression through the 3' UTR in sympathetic neurons. *BMC neuroscience*, 12:69, July 2011.

[435] Patricia Gomez-Bougie, Régis Bataille, and Martine Amiot. The imbalance between Bim and Mcl-1 expression controls the survival of human myeloma cells. *European Journal of Immunology*, 34(11):3156–3164, November 2004.

[436] H. Yamada, S. Tada-Oikawa, A. Uchida, and S. Kawanishi. TRAIL causes cleavage of bid by caspase-8 and loss of mitochondrial membrane potential resulting in apoptosis in BJAB cells. *Biochemical and Biophysical Research Communications*, 265(1):130–133, November 1999.

[437] Kai Huang, Jingjing Zhang, Katelyn L. O'Neill, Channabasavaiah B. Gurumurthy, Rolen M. Quadros, Yaping Tu, and Xu Luo. Cleavage by Caspase 8 and Mitochondrial Membrane Association Activate the BH3-only Protein Bid during TRAIL-induced Apoptosis. *The Journal of Biological Chemistry*, 291(22):11843–11851, May 2016.

[438] H Li, H Zhu, C J Xu, and J Yuan. Cleavage of BID by caspase 8 mediates the mitochondrial damage in the Fas pathway of apoptosis. *Cell*, 94(4):491–501, August 1998.

[439] John-Paul Upton, Kathryn Austgen, Mari Nishino, Kristen M. Coakley, Andrew Hagen, Dan Han, Feroz R. Papa, and Scott A. Oakes. Caspase-2 cleavage of BID is a critical apoptotic signal downstream of endoplasmic reticulum stress. *Molecular and Cellular Biology*, 28(12):3943–3951, June 2008.

[440] Hyungjin Kim, Mubina Rafiuddin-Shah, Ho-Chou Tu, John R. Jeffers, Gerard P. Zambetti, James J.-D. Hsieh, and Emily H.-Y. Cheng. Hierarchical regulation of mitochondrion-dependent apoptosis by BCL-2 subfamilies. *Nature Cell Biology*, 8(12):1348–1358, December 2006.

[441] Pengbo Wang, Jennefer Lindsay, Thomas W. Owens, Ewa J. Mularczyk, Stacey Warwood, Fiona Foster, Charles H. Streuli, Keith Brennan, and Andrew P. Gilmore. Phosphorylation of the proapoptotic BH3-only protein bid primes mitochondria for apoptosis during mitotic arrest. *Cell Reports*, 7(3):661–671, May 2014.

[442] Sandra S. Zinkel, Kristen E. Hurov, Christy Ong, Farvardean M. Abtahi, Atan Gross, and Stanley J. Korsmeyer. A role for proapoptotic BID in the DNA-damage response. *Cell*, 122(4):579–591, August 2005.

[443] Kristopher A. Sarosiek, Xiaoke Chi, John A. Bachman, Joshua J. Sims, Joan Montero, Luv Patel, Annabelle Flanagan, David W. Andrews, Peter Sorger, and Anthony Letai. BID preferentially activates BAK while BIM preferentially activates BAX, affecting chemotherapy response. *Molecular Cell*, 51(6):751–765, September 2013.

[444] Dayong Zhai, Chaofang Jin, Ziwei Huang, Arnold C. Satterthwait, and John C. Reed. Differential regulation of Bax and Bak by anti-apoptotic Bcl-2 family proteins Bcl-B and Mcl-1. *The Journal of Biological Chemistry*, 283(15):9580–9586, April 2008.

[445] Simon N. Willis, Lin Chen, Grant Dewson, Andrew Wei, Edwina Naik, Jamie I. Fletcher, Jerry M. Adams, and David C. S. Huang. Proapoptotic Bak is sequestered by Mcl-1 and Bcl-xL, but not Bcl-2, until displaced by BH3-only proteins. *Genes & Development*, 19(11):1294–1305, June 2005.

[446] M. C. Wei, T. Lindsten, V. K. Mootha, S. Weiler, A. Gross, M. Ashiya, C. B. Thompson, and S. J. Korsmeyer. tBID, a membrane-targeted death ligand, oligomerizes BAK to release cytochrome c. *Genes & Development*, 14(16):2060–2071, August 2000.

[447] Jaigi P. Mathai, Marc Germain, and Gordon C. Shore. BH3-only BIK regulates BAX,BAK-dependent release of Ca2+ from endoplasmic reticulum stores and mitochondrial apoptosis during stress-induced cell death. *The Journal of Biological Chemistry*, 280(25):23829–23836, June 2005.

[448] Haiming Dai, X. Wei Meng, Sun-Hee Lee, Paula A. Schneider, and Scott H. Kaufmann. Context-dependent Bcl-2/Bak interactions regulate lymphoid cell apoptosis. *The Journal of Biological Chemistry*, 284(27):18311–18322, July 2009.

[449] Erinna F. Lee, Stephanie Grabow, Stephane Chappaz, Grant Dewson, Colin Hockings, Ruth M. Kluck, Marlyse A. Debrincat, Daniel H. Gray, Matthew T. Witkowski, Marco Evangelista, Anne Pettikiriarachchi, Philippe Bouillet, Rachael M. Lane, Peter E. Czabotar, Peter M. Colman, Brian J. Smith, Benjamin T. Kile, and W. Douglas Fairlie. Physiological restraint of Bak by Bcl-xL is essential for cell survival. *Genes & Development*, 30(10):1240–1250, May 2016.

[450] Marc Germain, Jocelyn Milburn, and Vincent Duronio. MCL-1 inhibits BAX in the absence of MCL-1/BAX Interaction. *The Journal of Biological Chemistry*, 283(10):6384–6392, March 2008.

[451] M. C. Wei, W. X. Zong, E. H. Cheng, T. Lindsten, V. Panoutsakopoulou, A. J. Ross, K. A. Roth, G. R. MacGregor, C. B. Thompson, and S. J. Korsmeyer. Proapoptotic BAX and BAK: A requisite gateway to mitochondrial dysfunction and death. *Science (New York, N.Y.)*, 292(5517):727–730, April 2001.

[452] J.-C. Martinou and R. J. Youle. Which came first, the cytochrome c release or the mitochondrial fission? *Cell Death and Differentiation*, 13(8):1291–1295, August 2006.

[453] Björn Oettinghaus, Donato D'Alonzo, Elisa Barbieri, Lisa Michelle Restelli, Claudia Savoia, Maria Licci, Markus Tolnay, Stephan Frank, and Luca Scorrano. DRP1-dependent apoptotic mitochondrial fission occurs independently of BAX, BAK and APAF1 to amplify cell death by BID and oxidative stress. *Biochimica Et Biophysica Acta*, 1857(8):1267–1276, August 2016.

[454] X. Zhang, Y. Ling, Y. Guo, Y. Bai, X. Shi, F. Gong, P. Tan, Y. Zhang, C. Wei, X. He, A. Ramirez, X. Liu, C. Cao, H. Zhong, Q. Xu, and R. Z. Ma. Mps1 kinase regulates tumor cell viability via its novel role in mitochondria. *Cell Death & Disease*, 7(7):e2292, July 2016.

[455] Hao Zhou, Pingjun Zhu, Jun Guo, Nan Hu, Shuyi Wang, Dandan Li, Shunying Hu, Jun Ren, Feng Cao, and Yundai Chen. Ripk3 induces mitochondrial apoptosis via inhibition of FUNDC1 mitophagy in cardiac IR injury. *Redox Biology*, 13:498–507, October 2017.

[456] Z. N. Oltvai, C. L. Milliman, and S. J. Korsmeyer. Bcl-2 heterodimerizes in vivo with a conserved homolog, Bax, that accelerates programmed cell death. *Cell*, 74(4):609–619, August 1993.

[457] S. Manon, B. Chaudhuri, and M. Guérin. Release of cytochrome c and decrease of cytochrome c oxidase in Bax-expressing yeast cells, and prevention of these effects by coexpression of Bcl-xL. *FEBS letters*, 415(1):29–32, September 1997.

[458] Liying Zhou and Donald C. Chang. Dynamics and structure of the Bax-Bak complex responsible for releasing mitochondrial proteins during apoptosis. *Journal of Cell Science*, 121(Pt 13):2186–2196, July 2008.

[459] Ping Hu, Zhang Han, Anthony D. Couvillon, and John H. Exton. Critical role of endogenous Akt/IAPs and MEK1/ERK pathways in counteracting endoplasmic reticulum stress-induced cell death. *The Journal of Biological Chemistry*, 279(47):49420–49429, November 2004.

[460] C. Du, M. Fang, Y. Li, L. Li, and X. Wang. Smac, a mitochondrial protein that promotes cytochrome c-dependent caspase activation by eliminating IAP inhibition. *Cell*, 102(1):33–42, July 2000.

[461] Q. L. Deveraux, N. Roy, H. R. Stennicke, T. Van Arsdale, Q. Zhou, S. M. Srinivasula, E. S. Alnemri, G. S. Salvesen, and J. C. Reed. IAPs block apoptotic events induced by caspase-8 and cytochrome c by direct inhibition of distinct caspases. *The EMBO journal*, 17(8):2215–2223, April 1998.

[462] S. M. Srinivasula, M. Ahmad, T. Fernandes-Alnemri, and E. S. Alnemri. Autoactivation of procaspase-9 by Apaf-1-mediated oligomerization. *Molecular Cell*, 1(7):949–957, June 1998.

[463] P. Li, D. Nijhawan, I. Budihardjo, S. M. Srinivasula, M. Ahmad, E. S. Alnemri, and X. Wang. Cytochrome c and dATP-dependent formation of Apaf-1/caspase-9 complex initiates an apoptotic protease cascade. *Cell*, 91(4):479–489, November 1997.

[464] H. R. Stennicke, J. M. Jürgensmeier, H. Shin, Q. Deveraux, B. B. Wolf, X. Yang, Q. Zhou, H. M. Ellerby, L. M. Ellerby, D. Bredesen, D. R. Green, J. C. Reed, C. J. Froelich, and G. S. Salvesen. Pro-caspase-3 is a major physiologic target of caspase-8. *The Journal of Biological Chemistry*, 273(42):27084–27090, October 1998.

[465] Sabrina L. Spencer, Suzanne Gaudet, John G. Albeck, John M. Burke, and Peter K. Sorger. Non-genetic origins of cell-to-cell variability in TRAIL-induced apoptosis. *Nature*, 459(7245):428–432, May 2009.

[466] S. J. Riedl, M. Renatus, R. Schwarzenbacher, Q. Zhou, C. Sun, S. W. Fesik, R. C. Liddington, and G. S. Salvesen. Structural basis for the inhibition of caspase-3 by XIAP. *Cell*, 104(5):791–800, March 2001.

[467] B. B. Wolf, M. Schuler, F. Echeverri, and D. R. Green. Caspase-3 is the primary activator of apoptotic DNA fragmentation via DNA fragmentation factor-45/inhibitor of caspase-activated DNase inactivation. *The Journal of Biological Chemistry*, 274(43):30651–30656, October 1999.
