# Supplementary material for: Unlocking mitochondrial dysfunction-associated senescence (MiDAS) with NAD+ – A Boolean model of mitochondrial dynamics and cell cycle control
Source: Transl Oncol. 2024 Aug 19;49:102084. doi: 10.1016/j.tranon.2024.102084 (PMC11380032; doi:10.1016/j.tranon.2024.102084)
Supplement: Supplementary file 16 [file mmc16.pdf]

2. Results, section 2 - Cell Cycle-Dependence of Mitochondrial Morphology

a) Model reproduces experimentally observed mitochondrial dynamics during the cell cycle

<u>Relevant SM Tables and Figures:</u>

- **SM Table 2.** Model reproduces experimentally observed mitochondrial dynamics during cell cycle progression.
- **SM Figure 1.** Full version of Fig. 3: Model reproduces cell cycle-linked mitochondrial dynamics (hyperfusion at G1/S, fission in mitosis) and G1 arrest in response to glucose withdrawal.

SM Table 2. Mitochondrial dynamics during cell cycle progression

**Model behavior**<br>✤ new here / * inherited from [45] / ✲ prediction		**Figure**	**Experimentally observed cell behavior**
	• Stimulation with low mitogens (*GF* = ON but GF_{High} = OFF) leads to quiescent cells (**Fig 3A**, *left*).<br><br>• Stimulation with GF_{High} = ON leads to continuous cell cycle progression (**Fig 3A**,*middle*).	**Fig 3A**<br><br>**SM Fig 1**	– An increasing fraction of cells enter the cell cycle at increasing *EGF* or serum concentrations, reaching ~100% at 20 ng/mL *EGF* / 5% serum in MCF10A cells [78]; similar results were reported for rat embryonic fibroblasts [79].<br>– Mouse fetal fibroblasts display wide heterogeneity in the timing of the G1 / S transition regardless of the level or duration *of IGF-I, EGF, PDGF-AA*, or *PDGF-BB* treatment [80].
	• Mitogen washout experiments (GF_{High} = ON to GF_{High} = OFF) show that a cell can pre-commit to another division cycle before finishing its current mitosis and execute a full cell cycle in the absence of mitogenic stimulation (**Fig 3A**, *right*).	**Fig 3A**<br><br>**SM Fig 1**	– Rapidly dividing mammalian cells (MCF10A, Swiss3T3) can pre-commit to a division cycle before finishing their current one; they often execute a full cell cycle after mitogen withdrawal, such that their last exposure to mitogens occurs sometime during the previous G2 phase [81].
	✤ Stimulation with GF_{High} = ON leads to cyclic mitochondrial dynamics during cell cycle progression (**Fig 3A**, *middle*), involving:<br>✤ *E2F1*-induced hyperfusion of the mitochondrial network at the G1/S transition, leading to increased $\Delta\Psi_M$ and ATP generation, which helps activate *Cyclin E*<br>✲ Strong ETC activity in a healthy hyper- fused network increases SIRT3 activity to block runaway ROS production.<br>✤ Resetting of the mitochondrial network to its basal state in G2 (similar to G0) due to *E2F1*inhibition by *Cyclin A/Cdk2.*<br>✤ *Cyclin B/Cdk1*-mediated activation of the fission protein *Drp1*, resulting in an unfused (fragmented) state with relatively normal $\Delta\Psi_M$, which aids bipolar mitotic spindle formation and SAC passage.	**Fig 3A**<br><br>**SM Fig 1**	– *E2F1* is a direct transcriptional inducer of *MFN2,* which in turn increases mitochondrial fusion [28].<br>– Mitochondrial inner membrane fusion raises the efficiency of OXPHOS and increases the network's $\Delta\Psi_M$ [27].<br>– A high $\Delta\Psi_M$ mediated by hyper fusion and resulting in increased ATP production in late G1 are required for *Cyclin E* accumulation and S-phase entry [28].<br>– <u>*Indirect support*</u>*:* SIRT3 is necessary for healthy proliferation [17].<br>– During mitosis, *Drp1* is phosphorylated and activated by *Cdk1/Cyclin B*, resulting in mitochondrial fragmentation and proper bipolar spindle formation [28,31,82].<br>– TCA cycle metabolite levels oscillate during cell cycle progression to support high $\Delta\Psi_M$ at the G1/S boundary, but do not crash in mitosis [83].

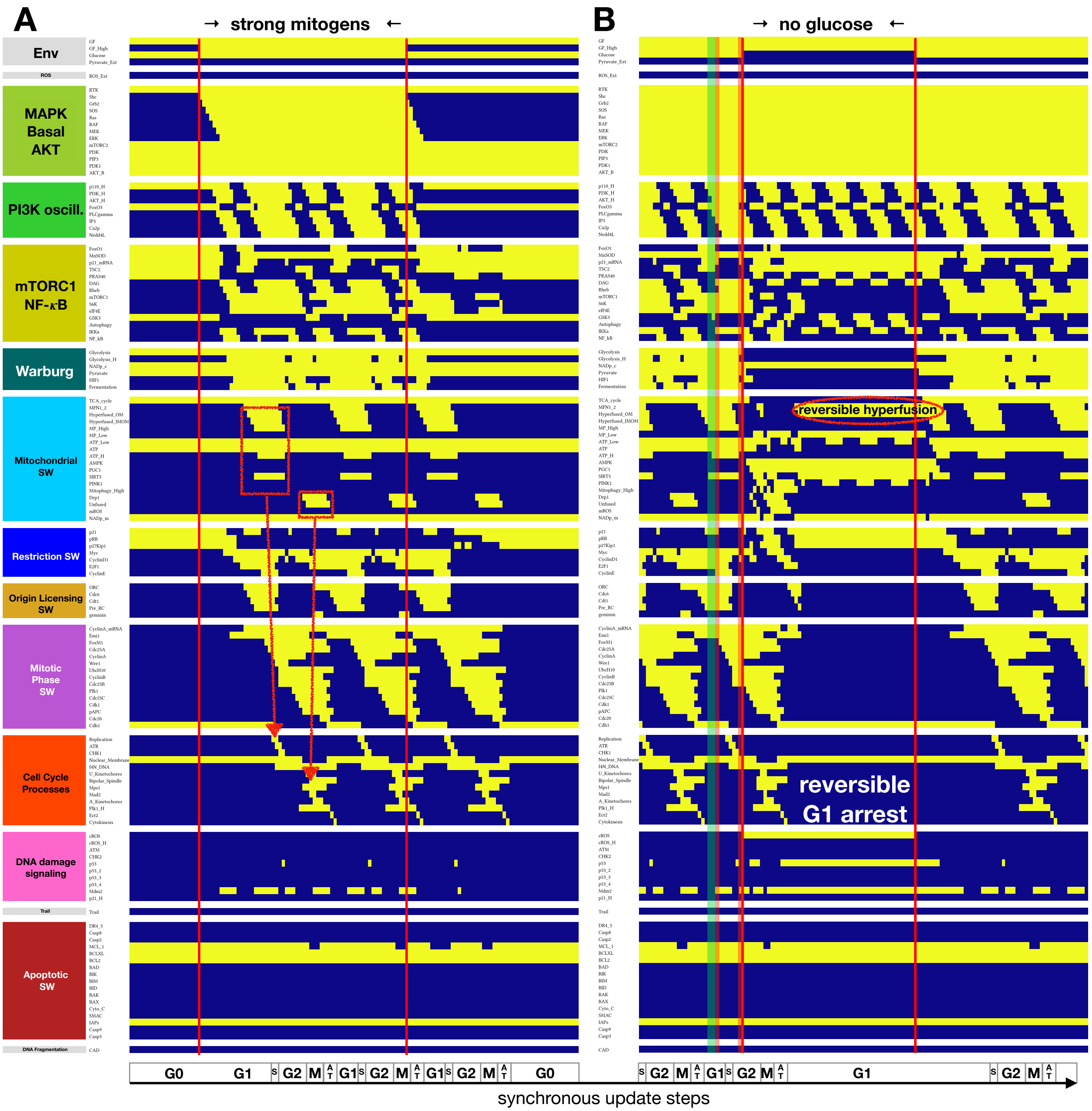

SM Figure 1. Full version of Fig. 3: Model reproduces cell cycle-linked mitochondrial dynamics (hyperfusion at G1/S, fission in mitosis) and G1 arrest in response to glucose withdrawal. A) Dynamics of regulatory molecule expression/activity during exposure of a quiescent cell to a saturating mitogenic signal for 60 update steps (full network). **B)** Dynamics of regulatory molecule expression/activity in a dividing cell responding to glucose withdrawal in early G1 for 50 update steps (full network). *X-axis:* time-steps; *y-axis:* nodes organized by regulatory modules; *yellow/dark blue:* ON/OFF; *vertical red lines:* start/end of signal; *red boxes with arrows:* morphological changes to mitochondria linked to cell cycle processes; *red oval*: reversibly hyperfused mitochondrial network; *shaded green box:* window of glucose withdrawal resulting in endoreduplicaiton (**SM Fig. 6A**); *shaded red bands:* windows of glucose withdrawal resulting in apoptosis (**SM Fig. 6B-C**).

b) *Model reproduces experimentally observed mitochondrial dynamics under cell cycle perturbations*

Relevant SM Tables and Figures:

- **SM Table 3.** Model reproduces experimentally observed mitochondrial dynamics under perturbations of cell cycle progression, accompanied by mitochondrial morphology change, and/or perturbations to mitochondrial fusion/fission proteins.
- **SM Figure 2.** Model reproduces the cell cycle phase-dependent effects of *Plk1* knockout and showcases difference between mitotic and apoptotic mitochondrial fragmentation.
- **SM Figure 3.** Model reproduces the cell cycle arrest induced by MFN1/2 knockdown or forced lowering of $\Delta\Psi_M$.
- **SM Figure 4.** Model reproduces loss of mitotic mitochondrial fragmentation and spindle assembly defects induced by Drp1 knockdown and/or forced mitochondrial hyperfusion.
- **SM Figure 5.** Model reproduces cell cycle arrest and apoptosis in cells with extreme mitochondrial hyperfusion.

SM Table 3. Perturbations of cell cycle progression accompanied by mitochondrial morphology change

	**Model behavior**<br>✤ new here / * inherited from [45] / ✲ prediction	**Figure**	**Experimentally observed cell behavior**
	• Cycling cells experiencing *Plk1* knockdown at different points along the cell cycle, leading to:<br>— G2 arrest when *Plk1* is lost before the G2/M transition<br>— mitotic catastrophe when *Plk1* is lost in early metaphase<br>— aneuploidy when *Plk1* is lost in late metaphase<br>— no cytokinesis followed by endoreduplication when *Plk1* is lost after SAC passage.	**SM Fig. 2**	– *Plk1* activation by *Cyclin A/Cdk2* at the G2/M boundary is required for mitotic entry [84].<br>– *Plk1* is required for the formation and maintenance of microtubule-kinetochore attachments [85,86].<br>– Aneuploidy and genome duplication have been documented in *Plk1*-inhibited cells [87].<br>– Due to *Plk1*'s role in driving contractile ring assembly, *Plk1* knockdown in telophase blocks cytokinesis [88].
	• Saturating *Trail* exposure kills quiescent as well as cycling cells.<br>• Non-saturating *Trail* leads to fractional killing<br>• Cells with prolonged metaphase are the most sensitive to *Trail*- mediated apoptosis.	virtual exp. in **SM File 10**	– Saturating *Trail* can kill ~100% of cells (immortalized human T lymphocytes [89], MCF10A [90], pancreatic cancer [91], glioblastoma & colon cancer cell lines [92]).<br>– *Trail* is synergistic with microtubule-targeting chemotherapy agents that trap cells in metaphase and delay SAC [92].
	✤ *Caspase 2*-mediated *BAK/BAX* activation during mitotic catastrophe activates *Drp1* to induce mitochondrial fragmentation, lowers $\Delta\Psi_M$, and increases mitochondrial ROS.<br>✤ In contrast to fragmentation in mitosis, apoptotic fragmented mitochondria aid *Cytochrome C* release and apoptosis.	**SM Fig. 2B**	– Mitotic catastrophe in IR-treated cells requires *Drp1*-induced mitochondrial fission [95].
	✤ Cycling cells experiencing *MFN1/2* knockdown show G1 arrest.<br>✤ Cycling cells experiencing forced low $\Delta\Psi_M$ (forced *MP_low* = ON) starting in late S-phase results in G1 arrest with no *Cyclin E* as well as increased *p21* and *p53*.	**SM Fig. 3**	– Cells infected with *Mfn2* shRNA show inhibited cell proliferation [96].<br>– Dividing cells treated with an oxidative phosphorylation uncoupler undergo G1 arrest, show a lack of *Cyclin E* accumulation and increased *p21* expression [28].<br>– Reducing $\Delta\Psi_M$ with FCCP results in G1–S arrest [28].

	✤ Cycling cells experiencing partial (60%) *Drp1* knockdown cannot sustain mitochondrial fragmentation during mitosis, resulting in problems with spindle assembly and prolonged SAC arrest.<br>✲ Cycling cells experiencing full *Drp1* knockdown arrest at the SAC long enough to undergo apoptosis.<br>✤ Cycling cells experiencing a brief period of *Drp1* knockdown in G2 do not arrest their cell cycle.<br>✤✲ Cycling cells experiencing prolonged *Drp1* knockdown arrest at the SAC and eventually undergo apoptosis.<br>✲ Cycling cells experiencing full *Drp1* knockdown undergo MiDAS.<br>✤ Cycling cells experiencing 50% forced outer membrane hyperfusion show prolonged SAC arrest due to spindle assembly delays and stochastically undergo apoptosis.	**SM Fig. 4 SM Fig. 5**	– Cells expressing dominant negative mutant *Drp1K38A* (DRP1m) fail to undergo mitochondrial fission during mitosis [31].<br>– *Indirect support*: high concentrations of mdivi-1 (mitochondrial division inhibitor) induce apoptosis [97].<br>– Cells overexpressing dominant negative *DRP1m* in a *brief* window before cells undergo mitosis experience no cell cycle inhibition [28].<br>– Perpetually hyperfused mitochondria generated by *prolonged DRP1m* expression significantly decreases proliferation [28].<br>– *Indirect support*: *Drp1* knockdown leads to mitochondrial dysfunction in muscle cells [97,98] and senescence in endothelial cells [99].<br>– Cells treated with a mitochondrial division inhibitor (mdivi-1) to induce hyperfusion have severely misaligned metaphase chromosomes [28].<br>– High concentrations of mdivi-1 not only block proliferation but also induce apoptosis [97].
	✤ *Trail*-induced apoptosis leads to *BAK/BAX*-mediated *Drp1* activation, mitochondrial fragmentation, and *Cytochrome C* release, along with the loss $\Delta\Psi_M$ and increased mROS.<br>✲ Partial Mitochondrial Outer Membrane Permeabilization (MOMP) via initially sub-lethal *Trail* can disrupt mitochondrial dynamics in G2/M, leading to mitotic catastrophe (discussed in *Results - 3*).<br><br>***Note:*** as most of our apoptosis results involve the intrinsic pathway, we chose not to include other extrinsic apoptotic signaling pathways such as FasL, TNF-α, or CD95-driven apoptosis. Given the similarities between FasL and Trail signaling, we hypothesize that our Trail results can be generalized to FasL signaling. In contrast, TNFR1 and CD95 also trigger pro-survival and pro-inflammatory signaling that may alter the fate of the cell and could alter the path to apoptosis and/or MiDAS (or trigger it by an alternate mechanism). The biological mechanisms needed to model this are not well explored in the experimental literature, and beyond our current scope.	**SM Fig. 10B**	– *Trail* induces *Drp1*-dependent mitochondrial fission by inducing Ser616 phosphorylation of *Drp1*, $\Delta\Psi_M$ loss, mROS, and cytochrome C release [94].<br>– *Indirect support*: non-lethal MOMP that only occurs in a small subset of mitochondria (minority MOMP) was documented in senescence, though it is unclear if it could be causal [94].

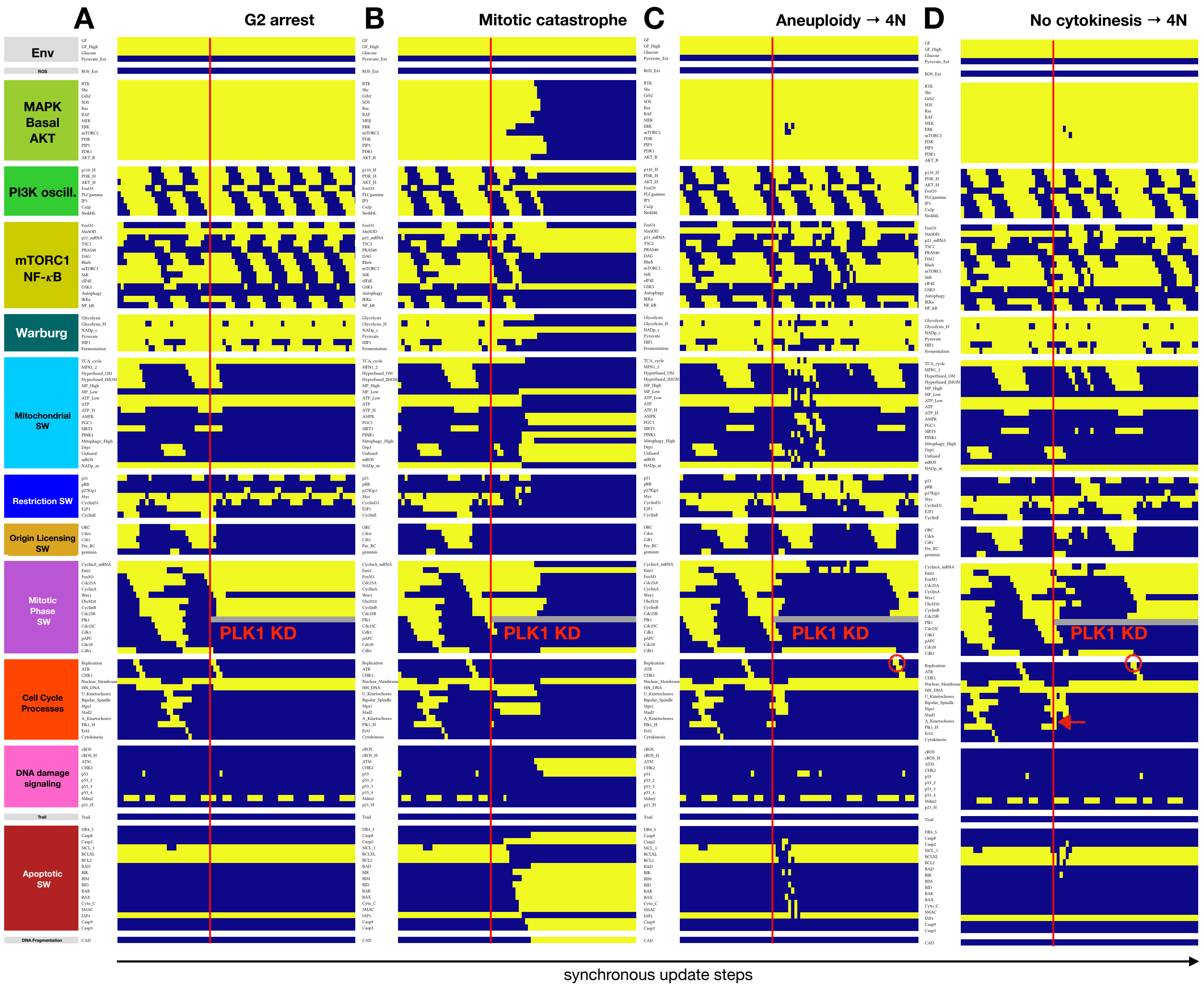

SM Figure 2. Model reproduces the cell cycle phase-dependent effects of *Plk1* knockout and showcases difference between mitotic and apoptotic mitochondrial fragmentation. A-D) Dynamics of regulatory molecule expression/ activity during full *Plk1* knockdown during cell cycle progression in (A) G2, leading to G2 arrest; *red box:* mitotic mitochondrial fission with normal $\Delta\Psi_M$; (B) prophase/early metaphase, leading to mitotic catastrophe and apoptosis; *red box:* apoptotic mitochondrial fission with low $\Delta\Psi_M$ and ROS production; (C) later in metaphase where *Cyclin A* loss results in APC^{Cdh1} activation before apoptosis can set in, but also before the spindle is complete, leading to aneuploidy and endoreduplication; *red circle:* S-phase with 4N DNA content. (D) in anaphase past the SAC, leading to no cytokinesis and subsequent endoreduplication; *red circle:* S-phase with 4N DNA content; *red arrow:* node marking the completion of kinetochore attachment. *X-axis:* time-steps; *y-axis:* nodes organized in regulatory modules; *yellow/dark blue:* ON/OFF; *gray:* forced *Plk1* OFF state; *vertical red line:* start of *Plk1* knockdown.

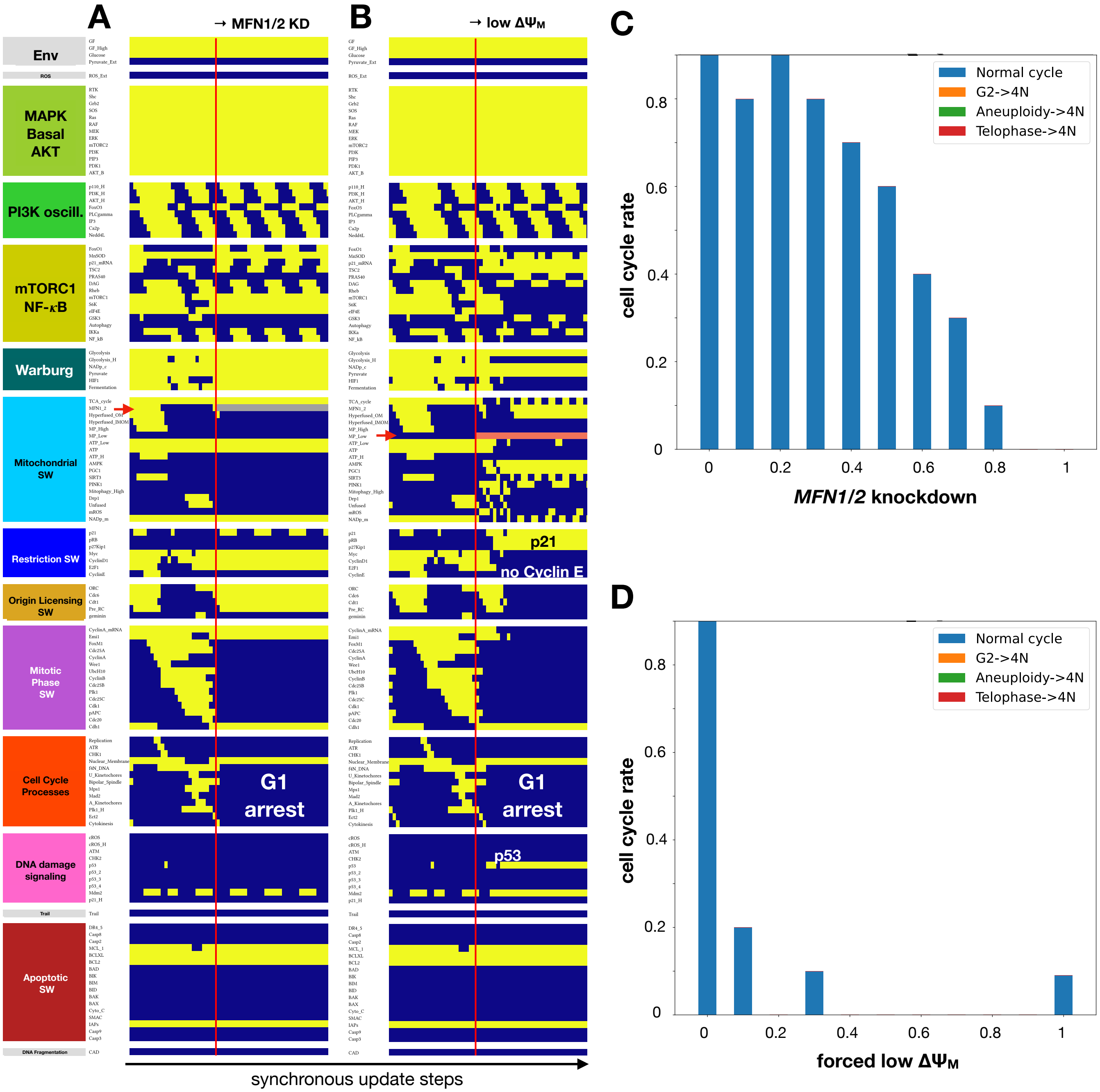

SM Figure 3. Model reproduces the cell cycle arrest induced by MFN1/2 knockdown or forced lowering of $\Delta\Psi_M$. **A)** Dynamics of regulatory molecule expression/activity during full *MFN1/2* knockdown in early G1, reproducing observations in [PMID: 25781899] that cells infected with *Mfn2* shRNA show inhibited cell proliferation; **B)** Dynamics of regulatory molecule expression/activity when $\Delta\Psi_M$ is forced to remain low starting before the previous cytokinesis, reproducing observations in [PMID: 19617534] that cells treated with the oxidative phosphorylation uncoupler FCCP arrest in G1, do not accumulate Cyclin E, and increase their *p21* expression. *X-axis:* time-steps; *y-axis:* nodes organized in regulatory modules; *yellow/dark blue:* ON/OFF; *gray/pink:* forced OFF/ON state; *vertical red line:* start of perturbation; *red arrows:* point of full *MFN1/2* knockdown, representing an inability to increase beyond its basal levels (A) and point of enforced low $\Delta\Psi_M$ (B); *white/black labels:* relevant outcomes. **C-D)** Response of cells dividing in 95% saturating growth signals to increasing *MFN1/2* knockdown (C) or increasingly low $\Delta\Psi_M$ (D). *Top/bottom:* rate of normal cell cycle completion (*blue*) vs. G2 → G1 reset (*orange,* not observed), aberrant mitosis (*green*, not observed), or failed cytokinesis followed by genome duplication (*red*, not observed), relative to the wild-type cell cycle length (25 time-steps), shown as stacked bar charts. *Initial state for sampling:* cycling cell in high glucose and no external pyruvate, ROS, or *Trail; sample size:* ≥ 2000 cells; *stop at:* apoptosis or MiDAS; *maximum length of single-cell tracks*: 250 steps (10 wild-type cell cycle lengths); *total sampled live cell time:* 500,000 steps; *update:* synchronous.

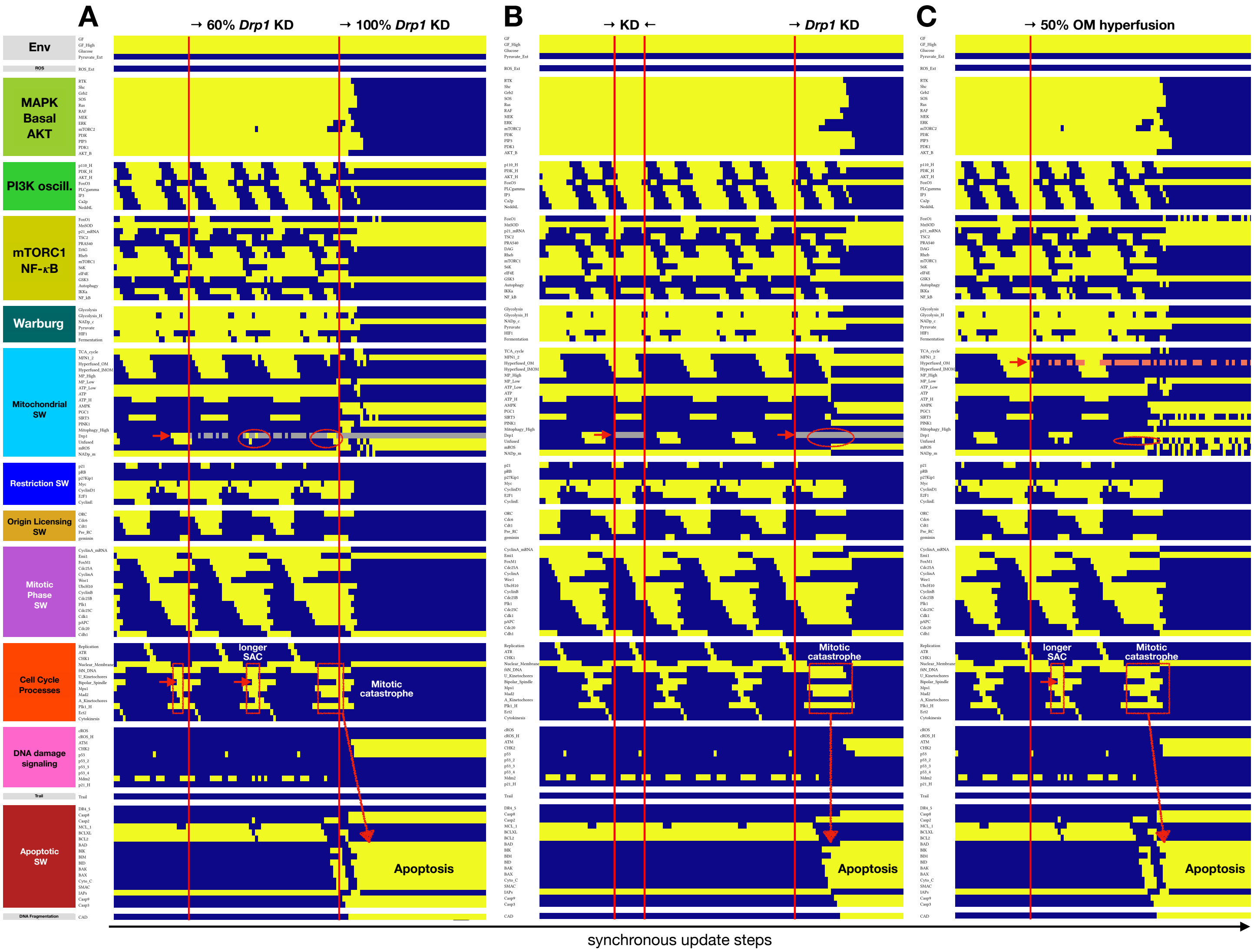

SM Figure 4. Model reproduces loss of mitotic mitochondrial fragmentation and spindle assembly defects induced by Drp1 knockdown and/or forced mitochondrial hyperfusion. A-C) Dynamics of regulatory molecule expression/activity during (A) partial vs. full *Drp1* knockdown (*middle:* for 50 steps/*last interval:* 50 update steps), (B) short vs. prolonged *Drp1* knockdown and (C) partial outer membrane hyperfusion in cycling cells, reproducing observations that: ***i)*** Cells expressing dominant negative mutant Drp1K38A (DRP1m) fail to undergo mitochondrial fission during mitosis (A, *red circles*) [PMID: 17301055]; ***ii)*** While there is no cell cycle inhibition in cells overexpressing DRP1m in a brief window before cells undergo mitosis (B, *left interval:* 10 update steps), perpetually hyperfused mitochondria (prolonged Drp1K38A expression) significantly decreases proliferation (B, *right interval*, predicting apoptosis) [PMID: 19617534]; ***iii)*** Cells treated with a mitochondrial division inhibitor (mdivi-1) to induce mitochondrial hyperfusion have severely misaligned metaphase chromosomes (C, *red box*) [PMID: 19617534]; ***iv)*** High concentrations of mdivi-1 not only block proliferation but also induce apoptosis (C) [PMID: 32147668]. *X-axis:* time-steps; *y-axis:* nodes organized in regulatory modules; *yellow/dark blue:* ON/OFF; *gray/pink:* forced OFF/ON states; *vertical red lines:* start/change in perturbation; *red arrows (top set):* start of *Drp1* knockdown (A,B) or outer membrane hyperfusion (C); *red boxes with arrow:* intervals between start of Metaphase (*U_Kinetochores* = ON) and Spindle Assembly Checkpoint passage (*Mad2* = OFF), with an arrow indicating the start of proper spindle alignment (*Bipolar_Spindle* = ON); *white/black labels:* relevant outcomes.

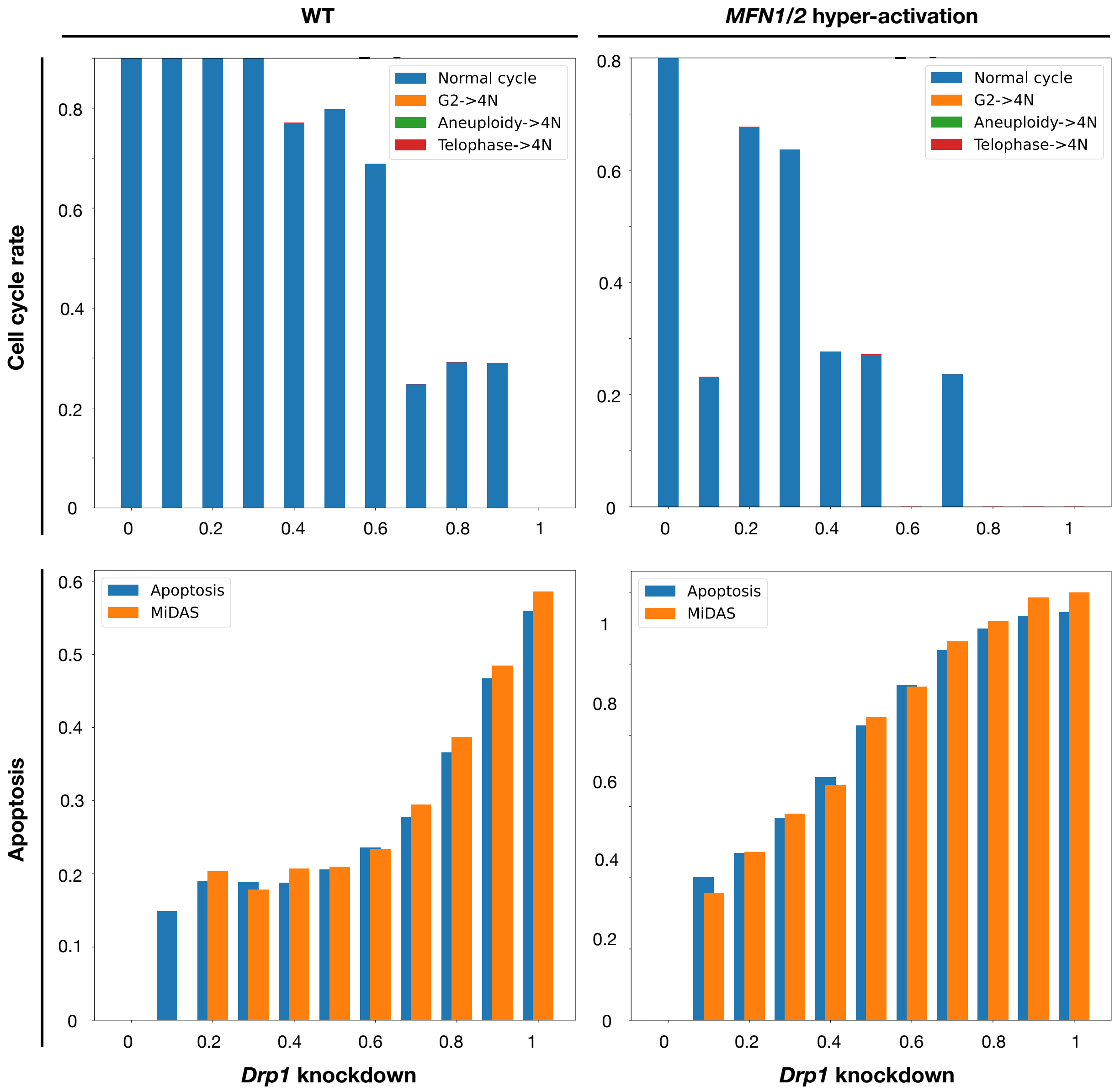

SM Figure 5. Model reproduces cell cycle arrest and apoptosis in cells with extreme mitochondrial hyperfusion. Response of cells dividing in 95% saturating growth stimuli to increasing levels of Drp1 knockdown alone (*left*) or in Mfn1/2 over-expressing cells (*right*). These two scenarios cover the range from normal dynamics to extreme hyper-fusion. Wild-type dynamics (*left, bars near 0*) and MFN1/2 hyper-activation that mitotic Drp1 can overpower (MFN1/2 *panels, bars near 0*) both show normal cell cycle progression. In contrast, the inability to form fragmented mitochondria without permanent hyperfusion (*wild-type, bars near 1 for 100%* Drp1 *knockdown*) and extreme hyperfusion (MFN1/2 *panels, bars near 1 for 100%* Drp1 *knockdown*) both block cell cycle progression and induce either apoptosis or MiDAS. Results reproduce the observed cell cycle arrest in cells with long-term *DRP1m* expression, where the mitochondrial network is hyperfused [PMID: 19617534]. They also *predict* a mix of apoptosis by mitotic catastrophe and MiDAS, supported by observations of apoptosis and mitochondrial dysfunction/senescence following Drp1 inhibition [PMIDs: 32147668, 32539155, 37517319] (these studies do not probe for specifically for MiDAS). *Top row:* rate of normal cell cycle completion (*blue*) vs. G2 → G1 reset (*orange,* not observed), aberrant mitosis (*green*, not observed), or failed cytokinesis followed by genome duplication (*red*, not observed), relative to the wild-type cell cycle length (25 time-steps) in wild-type (*left*) vs. *Mfn1/2* over-expressing cells (*right*), shown as stacked bar charts. *Bottom row:* rate of apoptosis (*blue*) relative to the wild-type cell cycle length (25 time-steps) in wild-type (*left*) vs. *Mfn1/2* over-expressing cells (*right*). *Initial state for sampling:* cycling cell in high glucose and no external pyruvate, ROS, or *Trail; sample size:* ≥ 2000 cells; *stop at:* apoptosis or MiDAS; *maximum length of single-cell tracks*: 250 update steps (10 wild-type cell cycle lengths); *total sampled time:* 500,000 steps; *update:* synchronous.

c) *Model reproduces experimentally observed mitochondrial dynamics under glucose starvation*

Relevant SM Tables and Figures:

- **SM Table 4.** Model reproduces experimentally observed mitochondrial dynamics under glucose starvation.
- **SM Figure 6.** Model predicts endoreduplication or apoptosis in rapidly cycling cells upon glucose withdrawal at different points along the G1/early-G2 window.

SM Table 4. Mitochondrial dynamics during glucose starvation

**Model behavior**<br>✤ new here / * inherited from [45] / ✲ prediction		**Figure**	**Experimentally observed cell behavior**
	✤ Cycling cells glucose withdrawal undergo reversible G1 arrest, activate AMPK, which leads to PGC1α-mediated MFN1/2 up-regulation and indirect Drp1 inhibition mediated by *p53* (itself induced by AMPK and increased cellular ROS production).<br>✤ MFN1/2 up-regulation and Drp1 inactivation in response to glucose withdrawal leads to reversible mitochondrial hyperfusion and no increase in mitophagy (in spite of low $\Delta\Psi_M$ due to lack of glucose).<br>✲ Hyperfused networks in glucose-starved cells fuse their outer membranes only, as inner membrane fusion requires normal $\Delta\Psi_M$.	**Fig. 3B**	– Non-lethal glucose starvation induces reversible G1 arrest [100] with strong AMPK activation [101].<br>– Nutrient depletion, including glucose starvation, leads to significantly elongated and interconnected mitochondria, mediated by down-regulation of Drp1 and unopposed mitochondrial fusion [101].<br>– Mitochondrial hyperfusion upon nutrient deprivation protects mitochondria from mitophagy [101].
	✲ Cells that experience glucose withdrawal in a pre-committed G1 state; one that passed the restriction point *before* finishing its previous cytokinesis, undergo G2 arrest followed by endo-reduplication. Predicted mechanism (positive feedback on **SM Fig. 6B**; regulatory link details in **SM Table 1**):<br>— Glucose withdrawal leads to ROS accumulation and p53-mediated Myc repression.<br>— Loss of Myc decreases FoxM1, a critical G2 transcription factor responsible for Cdc25A/B, as well as Cyclin A induction.<br>— Positive feedback from Cyclin A/Cdk2 aided by Cdc25A/B can keep an existing FoxM1 pool active in G2 (**SM Fig. 6A**, *inset, gray arrows*), but glucose withdrawal interferes with this feedback and dooms the cell to an internal G1 reset.<br>— Upon re-exposure to glucose, the cell starts another round of DNA replication (**SM Fig. 6A**, *bottom red circle*).	**SM Figs. 6A, 6B**	– *Indirect support*:<br>~ Harsh tumor environments involving both glucose withdrawal and acidosis resulted in a ~19.4% pool of survivor cells with 4N DNA content (7 days of nutrient withdrawal with acidosis) [97].<br>~ 24 hours after nutrient restoration the fraction of G2/M cells briefly peak to a level that is consistent with pre-withdrawal levels (diploid cells reaching G2/M) *plus* the percentage of cells stuck with 4N DNA at day 7 of the withdrawal (potentially slower to re-enter the cell cycle) [97].
	✲ Hyperfusion in response to glucose withdrawal, if it persists into mitosis, delays mitotic mitochondrial fragmentation long enough to cause spindle assembly defects and apoptosis by mitotic catastrophe.	**SM Fig. 6C**	– *Indirect support*: glucose-starved cells show a significant increase in spindle formation defects such as multipolar spindles [97].

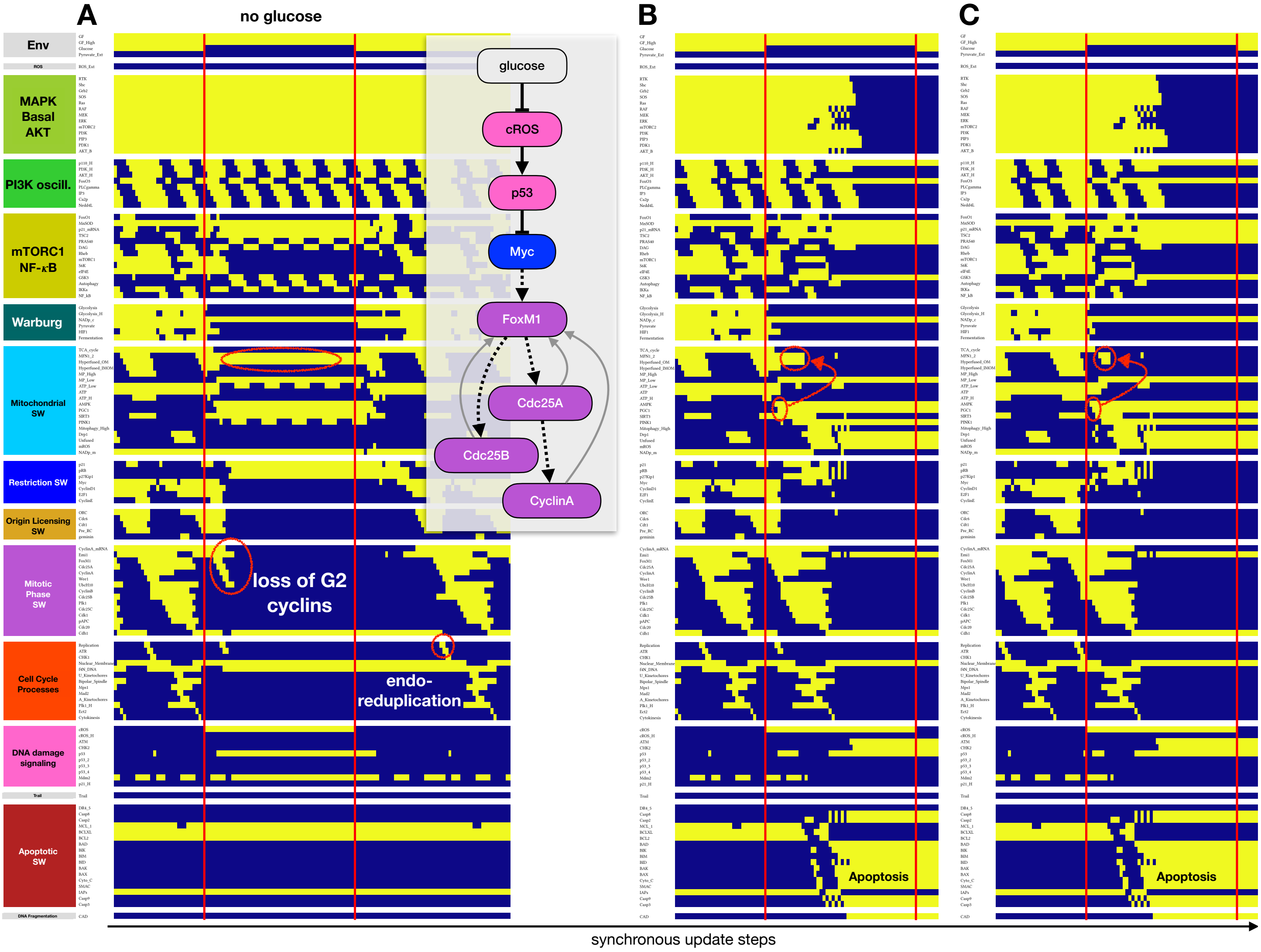

SM Figure 6. Model predicts endoreduplication or apoptosis in rapidly cycling cells upon glucose withdrawal at different points along the G1/early-G2 window. A-C) Dynamics of regulatory molecule expression/activity during glucose withdrawal for 50 update steps in (A) a pre-committed cell in early G1, which accumulates enough cyclin E to start DNA replication but cannot lock in a stable G2 state due to loss of Myc-induced FoxM1 accumulation (*inset*). (B-C) mid-G1 & early G2, where AMPK activation and hyperfusion prevent the timely mitotic fission of mitochondria (*red ovals with arrow*), causing bipolar spindle defects and mitotic catastrophe; *X-axis:* time-steps; *y-axis:* nodes organized in regulatory modules; *yellow/dark blue:* ON/OFF; *vertical red lines:* start/change in perturbation; *red ovals:* relevant molecular changes such as mitochondrial hyper-fusion (*top ovals*), loss of G2 cyclin A activity (A) and a second replication event with 4N DNA (A), AMPK → PGC1α activation (B, C); *white/black labels:* relevant outcomes.
