# Supplementary material for: Unlocking mitochondrial dysfunction-associated senescence (MiDAS) with NAD+ – A Boolean model of mitochondrial dynamics and cell cycle control
Source: Transl Oncol. 2024 Aug 19;49:102084. doi: 10.1016/j.tranon.2024.102084 (PMC11380032; doi:10.1016/j.tranon.2024.102084)
Supplement: Supplementary file 17 [file mmc17.pdf]

3. Results, section 3 - MiDAS

Relevant SM Tables and Figures:

- **SM Figure 7.** Model reproduces MiDAS in response to *SIRT3* loss and predicts that mitogen-arrested, but not glucose-arrested cells are protected from it.
- **SM Figure 8.** Model reproduces low $\Delta\Psi_M$-induced MiDAS prevented / rescued by pyruvate.
- **SM Figure 9.** Model predicts that external pyruvate can protect cells from MiDAS induced by *SIRT3* loss and reverse MiDAS to restore proliferation or healthy quiescence.
- **SM Figure 10.** Model predicts that sub-lethal MOMP that does not activate Caspase 9 but lowers $\Delta\Psi_M$ in cells with hyperfused mitochondria triggers MiDAS.

In addition to *SIRT3* knockdown, the electron transport chain inhibitor rotenone and mtDNA depletion were also shown to trigger MiDAS, a response that culturing cells in excess pyruvate could prevent [17]. To test whether our model can reproduce these experiments, we first mimicked lowered ETC activity by forcibly lowering the model cell's $\Delta\Psi_M$. Indeed, loss of $\Delta\Psi_M$ at the G1/S boundary in a cell committed to cell cycle triggered MiDAS, an effect rescued by pyruvate (**SM Fig. 8,** *middle vs. right window*). External pyruvate also prevented MiDAS following the loss of *SIRT3* (**SM Fig. 9A**). Moreover, the model predicted that pyruvate could rescue wild-type cells from MiDAS (early senescence only), freeing them to enter the cell cycle when exposed to mitogens (**SM Fig. 9B**) or restoring ATP generation and their potential for future proliferation in quiescence (**SM Fig. 9C**).

Further underscoring the key role of low $\Delta\Psi_M$ combined with mitochondrial hyperfusion, we have found that several perturbations described above for their role in disrupting the cell cycle can also trigger MiDAS – depending on their timing along the cycle. These include *Plk1* knockdown just before the cell clears the Spindle Assembly Checkpoint (SAC) (**SM Fig. 10A**), sub-lethal *Trail* exposure in cells pre-committed to the next cell cycle (**SM Fig. 10B**; late mitosis, starting hyperfusion for G1/S), and sub-lethal *Drp1* knockdown or mitochondrial hyperfusion in metaphase (**SM Fig. 10C-D**). The common predicted cause of MiDAS is a sub-lethal mitochondrial membrane permeabilization that lowers the $\Delta\Psi_M$ without activating executioner caspases (which would trigger apoptosis) – at a time when the mitochondria are hyperfused. As the cells they react to low $\Delta\Psi_M$ with *AMPK* activation, they lock in MiDAS.

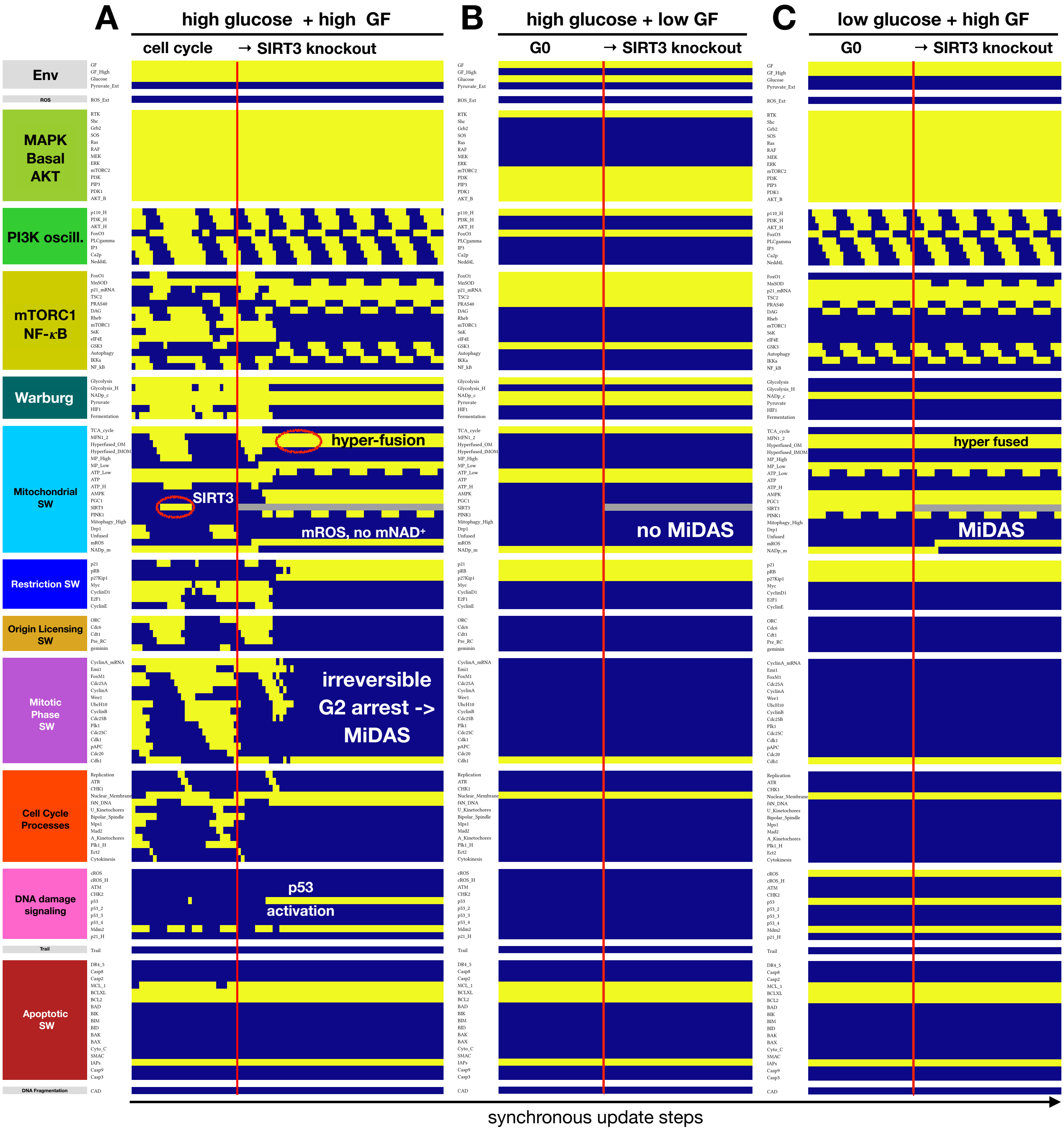

SM Figure 7. Model reproduces MiDAS in response to *SIRT3* loss and predicts that mitogen-arrested, but not glucose-arrested cells are protected from it. A-C) Dynamics of regulatory molecule expression/activity in: (A) a dividing cell responding to full *SIRT3* knockout in early G1 (full version of **Fig. 4B**); (B) a mitogen-arrested cell / (C) glucose-starved cell, responding to full *SIRT3* knockout, resulting in continued G0 / MiDAS, respectively. *X-axis:* time-steps; *y-axis:* nodes organized in regulatory modules; *yellow/dark blue:* ON/OFF; *pink/gray:* time-steps in which stochastic forced activation / knockdown has an effect; *vertical red lines:* start/change in perturbation; *white/black labels:* relevant outcomes.

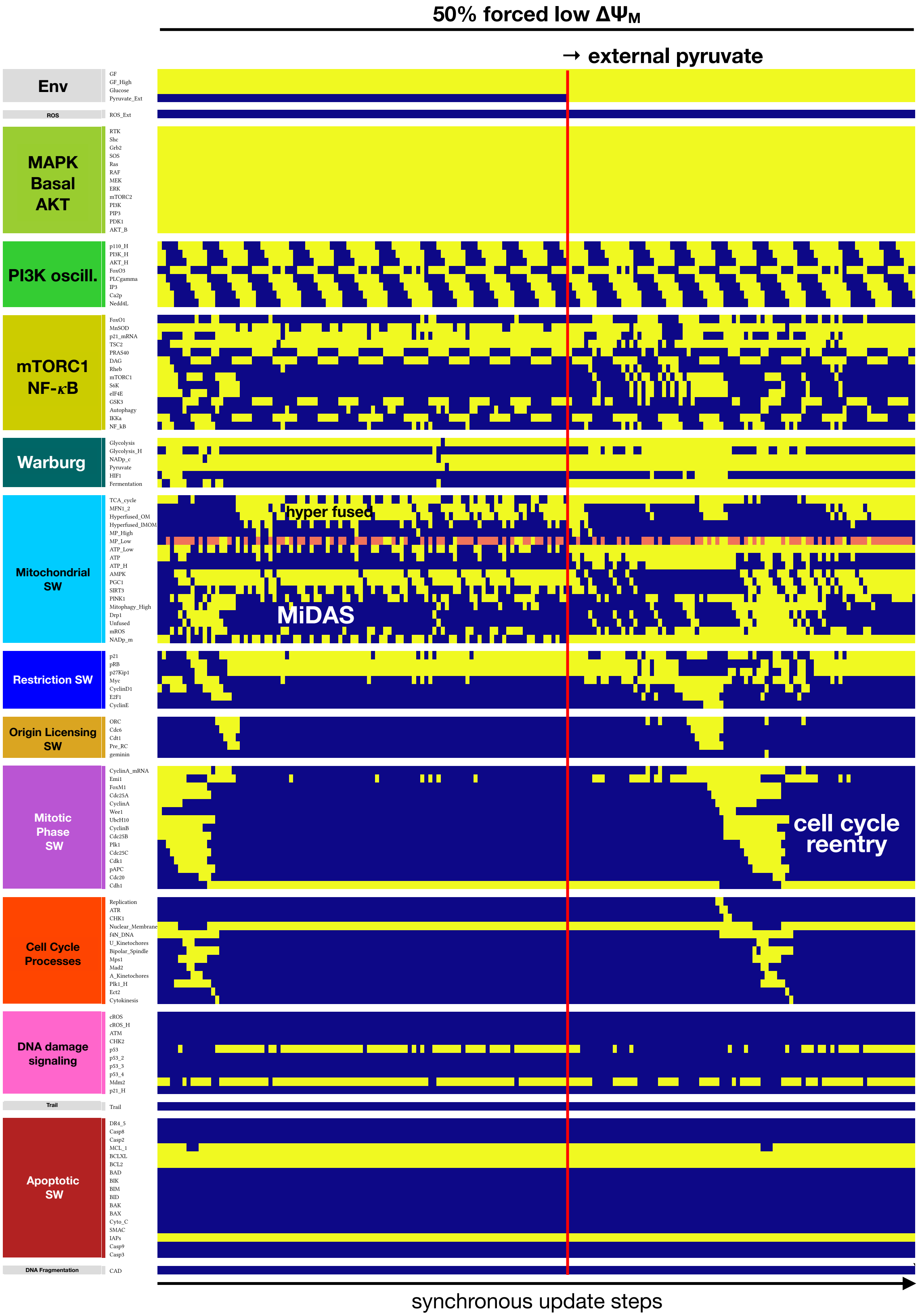

SM Figure 8. Model reproduces low $\Delta\Psi_M$-induced MiDAS prevented / rescued by pyruvate. Dynamics of regulatory molecule expression/activity in mitogen-stimulated cells exposed to an ETC inhibitor that lowers the $\Delta\Psi_M$ in 50% of the time steps, in the absence (*left*) vs. presence (*right*) of saturating external pyruvate. *X-axis:* time-steps; *y-axis:* nodes organized in regulatory modules; *yellow/dark blue:* ON/OFF; *pink/gray:* time-steps in which stochastic forced activation / knockdown has an effect; *vertical red lines:* start/change in perturbation; *white/black labels:* relevant outcomes.

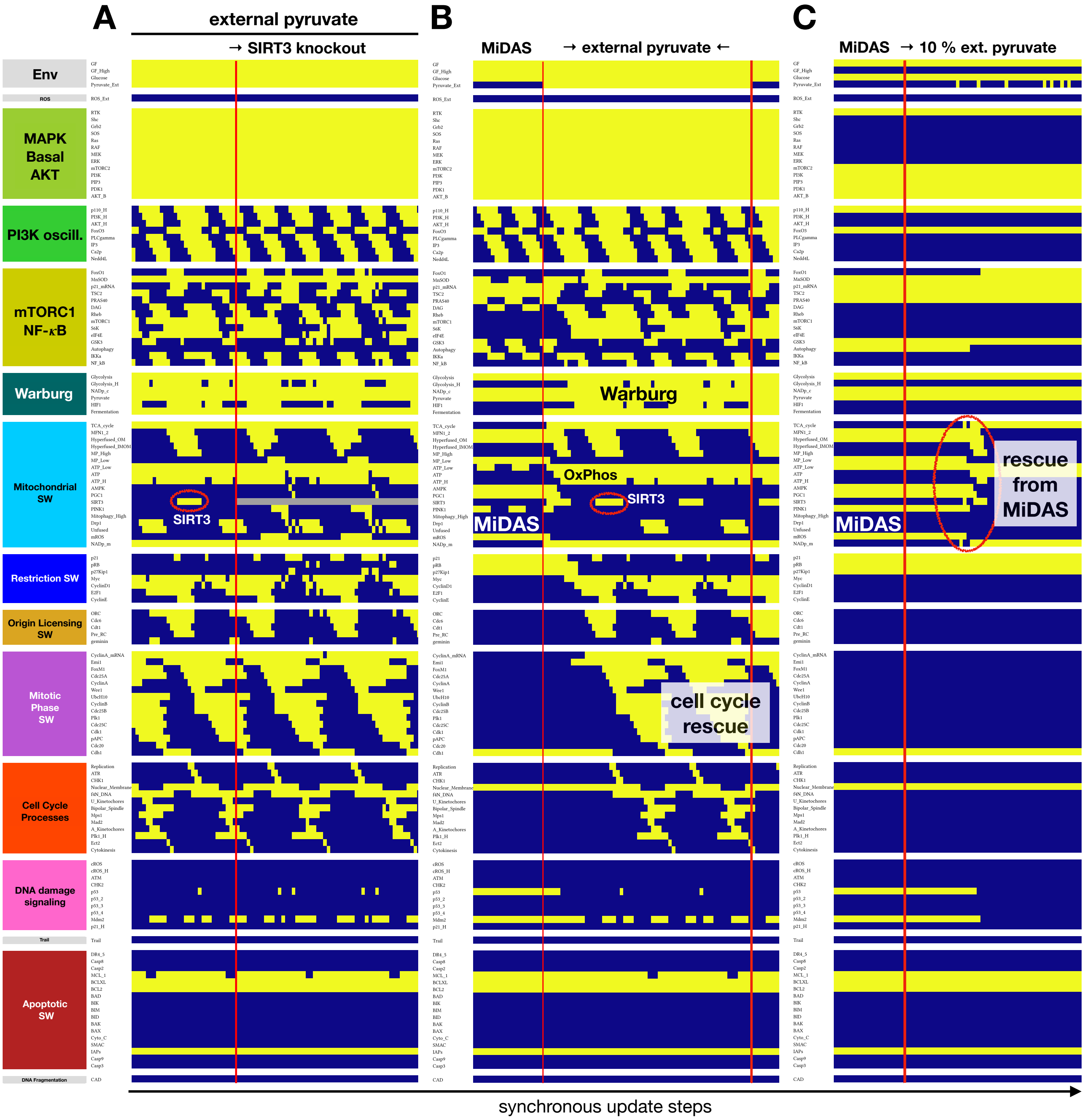

SM Figure 9. Model predicts that external pyruvate can protect cells from MiDAS induced by *SIRT3* loss and reverse MiDAS to restore proliferation or healthy quiescence. A) Dynamics of regulatory molecule expression/ activity in cycling cells with access to saturating external pyruvate, in response to full *SIRT3* knockout. **B)** Dynamics of regulatory molecule expression/activity in mitogen-exposed MiDAS cells in response to a prolonged pulse (60 steps) of external pyruvate. **C)** Dynamics of regulatory molecule expression/activity in MiDAS cells under low growth factor exposure, in exposed to low-dose (10%) external pyruvate. *X-axis:* time-steps; *y-axis:* nodes organized in regulatory modules; *yellow/dark blue:* ON/OFF; *gray:* full SIRT3 knockdown; *vertical red lines:* start/change in perturbation; *white/black labels:* relevant outcomes.

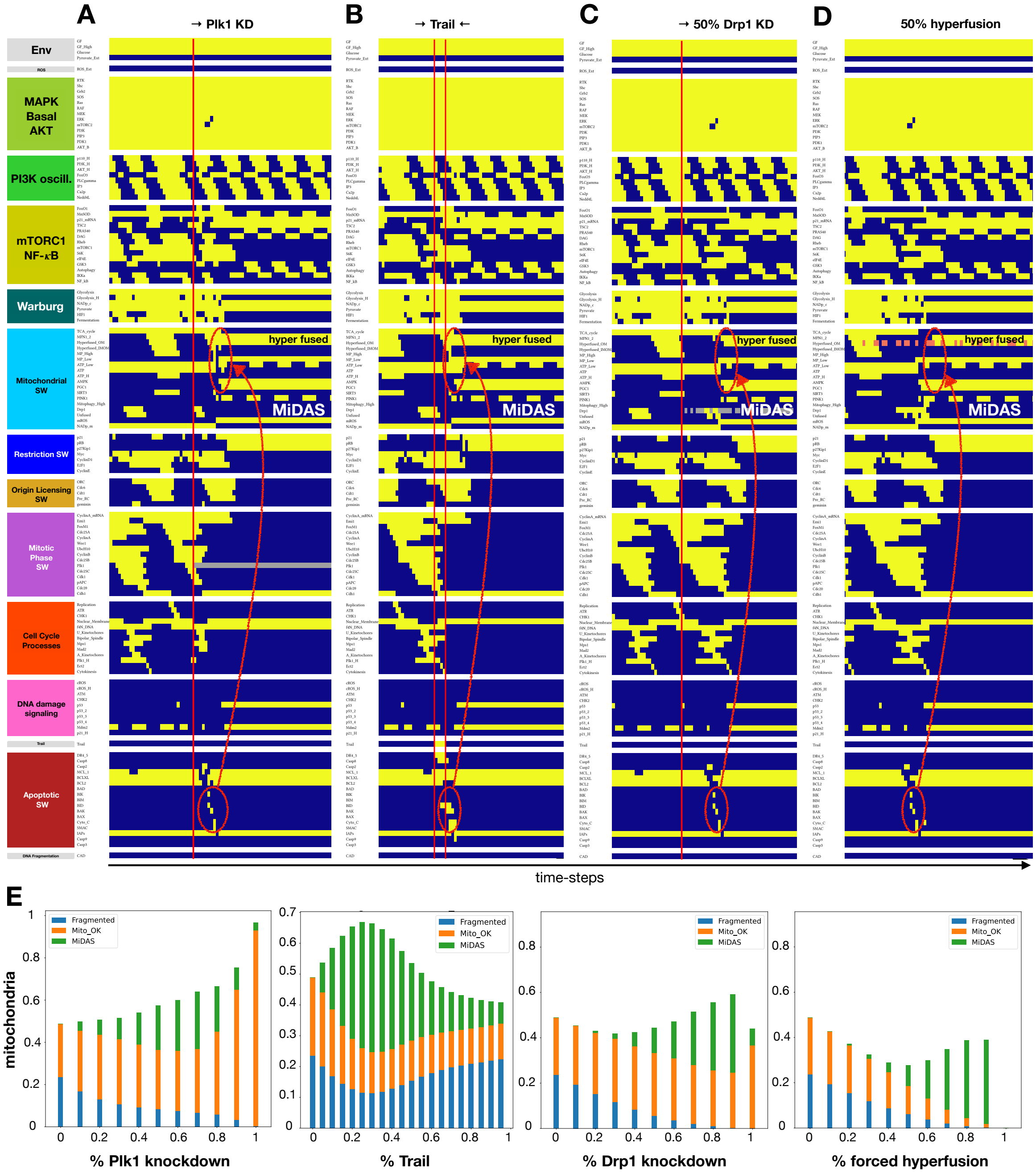

SM Figure 10. Model predicts that sub-lethal MOMP that does not activate Caspase 9 but lowers $\Delta\Psi_M$ in cells with hyperfused mitochondria triggers MiDAS. A) Dynamics of regulatory molecule expression/activity in cycling cells in response to full *Plk1* knockout in late metaphase, allowing them to narrowly avoid mitotic catastrophe but enter MiDAS. **B)** Dynamics of regulatory molecule expression/activity in cycling cells in response to a near-lethal *Trail* dose (4 steps) in anaphase, leading to MiDAS. **C)** Dynamics of regulatory molecule expression/activity in cycling cells in response to 50% *Drp1* knockdown, leading to prolonged SAC followed by MiDAS. **D)** Dynamics of regulatory molecule expression/activity in cycling cells in response to 50% outer mitochondrial membrane hyperfusion, leading to prolonged SAC followed by MiDAS. *X-axis:* time-steps; *y-axis:* nodes organized in regulatory modules; *yellow/dark blue:* ON/OFF; *gray/pink:* forced OFF/ON state; *red ovals & arrows:* start of MOMP (*bottom ovals*) leading to low $\Delta\Psi_M$ at a time of hyperfusion (*top ovals*); *vertical red lines:* start/change in perturbation; *white/black labels:* relevant outcomes. **E)** Response of cells dividing in 95% saturating growth stimuli to *Plk1* knockdown, *Trail, Drp1* knockdown and forced hyperfusion of the outer mitochondrial membrane (*left to right*), showing the fraction of time cells display normal mitochondria (*orange*), MiDAS (*green*), or fragmented mitochondria (*blue*). *Initial state for sampling:* cycling cell in high glucose, no external pyruvate, ROS, or *Trail; sample size:* ≥ 2000 cells; *stop at:* apoptosis; *maximum length of single-cell tracks*: 250 update steps (10 wild-type cycles); *total sampled time:* 500,000 steps; *update:* synchronous.
