# Supplementary material for: Unlocking mitochondrial dysfunction-associated senescence (MiDAS) with NAD+ – A Boolean model of mitochondrial dynamics and cell cycle control
Source: Transl Oncol. 2024 Aug 19;49:102084. doi: 10.1016/j.tranon.2024.102084 (PMC11380032; doi:10.1016/j.tranon.2024.102084)
Supplement: Supplementary file 18 [file mmc18.pdf]

4. Results, section 4 - ROS-induced MiDAS

Relevant SM Figures:

- **SM Figure 11.** Network neighborhood of the internal reactive oxygen species nodes *ROS* and *cROS_H*.
- **SM Figure 12.** Full version of Fig. 5: Model reproduces ROS-induced MiDAS in cycling cells and predicts protection from MiDAS in external pyruvate.
- **SM Figure 13.** Model reproduces reversible G2 arrest in response to brief / weak ROS exposure.
- **SM Figure 14.** Model predicts that *SIRT3* hyper-activation can protect from or reverse ROS-induced MiDAS, and reproduces MiDAS rescue by NAD+ boost.

In contrast to MiDAS in response to prolonged ROS, brief exposure led to *reversible* G2 arrest followed by mitosis (**SM Fig. 13**). Our model captures this difference via its four-node tracking of *p53* accumulation. In addition, our model predicted that quiescent cells were not as susceptible to ROS-induced MiDAS, owing their protection to continuous mitophagy (**Figs. 5C, SM 12C**). This was due to the lack of mitogen-induced AKT_H oscillations, aiding *FoxO1/3*-mediated mitophagy in blocking *MFN1/2*-driven hyperfusion. That said, cells that exited the cell cycle into MiDAS maintained MiDAS even in the absence of mitogens; (**Figs. 5A-B,** *right*).

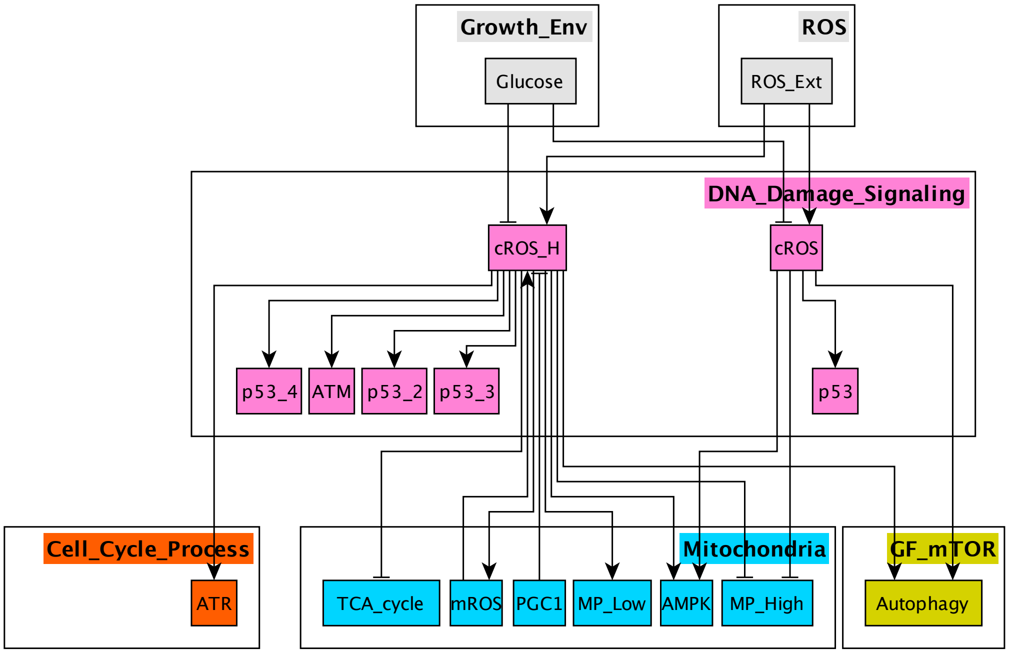

SM Figure 11. Network neighborhood of the internal reactive oxygen species nodes *ROS* and *cROS_H*. Part of our regulatory network model directly connected to the cROS and cROS_H nodes, including incoming and outgoing links. *Gray:* inputs representing environmental factors; *pink*: DNA damage response; *teal:* mitochondrial switch; *dark orange:* cell cycle processes; *mustard*: autophagy (new node in the mTORC1 growth module); → : activation; ⊣ : inhibition.

Given that external pyruvate could protect *SIRT3*-knockout cells from MiDAS by maintaining NAD^+ levels, we next asked whether pyruvate could also prevent or reverse ROS-induced MiDAS. Indeed, excess pyruvate prevented the drop in mitochondrial NAD^+ observed in our ROS-exposed model cells, allowing NAD^+ dependent *SIRT3* activity to prevent MiDAS (**Fig. 5D**). To test whether *SIRT3* hyper-activation itself could act in a similar way, we simultaneously exposed our model cell to external ROS and forced *SIRT3* activation (**SM Fig. 14A**). Our model predicted that cells with hyperactive *SIRT3* were protected from ROS-induced MiDAS, and that forced *SIRT3* activation could reverse MiDAS (**SM Fig. 14B**).

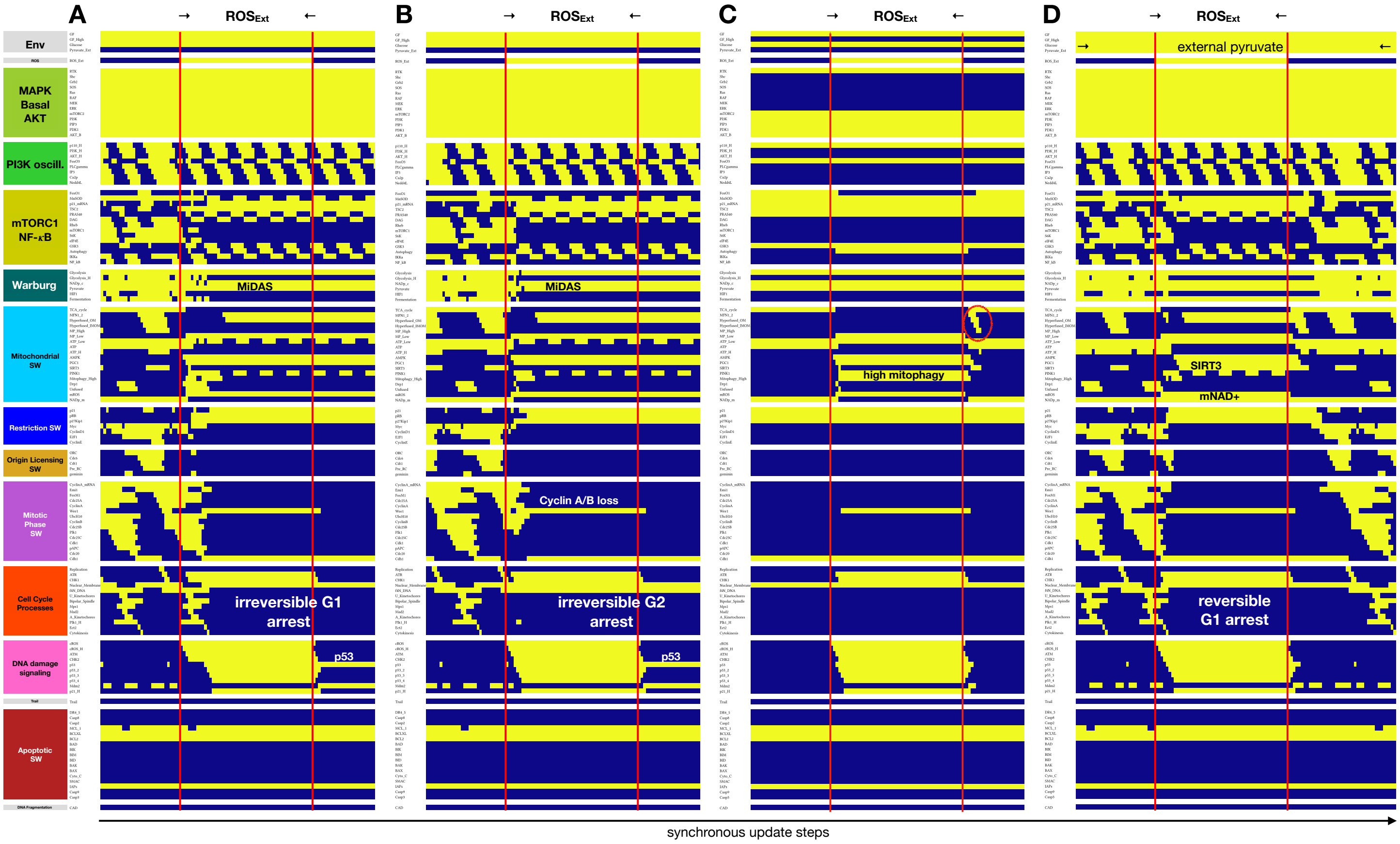

SM Figure 12. Full version of Fig. 5: Model reproduces ROS-induced MiDAS in cycling cells and predicts protection from MiDAS in external pyruvate. A-B) Dynamics of regulatory molecule expression/activity during exposure of a cycling cell to external ROS for 50 update steps in (A) prometaphase, leading to cytokinesis followed by MiDAS (2N DNA), vs. (B) early G2, leading to irreversible G2 arrest and MiDAS with 4N DNA. **C)** Dynamics of regulatory molecule expression/activity during exposure of a quiescent cell to external ROS for 50 update steps, leading to high mitophagy and short-term hyperfusion to restore ATP levels. **D)** Dynamics of regulatory molecule expression/ activity during exposure of a cycling cell exposed to saturating levels of external pyruvate to external ROS near the SAC, for 50 update steps, leading to reversible G1 arrest (cell cycle entry after external ROS is removed). *X-axis:* time-steps; *y-axis:* nodes organized by regulatory modules; *yellow/dark blue:* ON/OFF; *vertical red lines:* start/end of ROS exposure; *red oval on (C):* brief, homeostatic hyperfusion involving both outer and inner mitochondrial membranes; *white/black labels:* relevant molecular changes or outcomes.

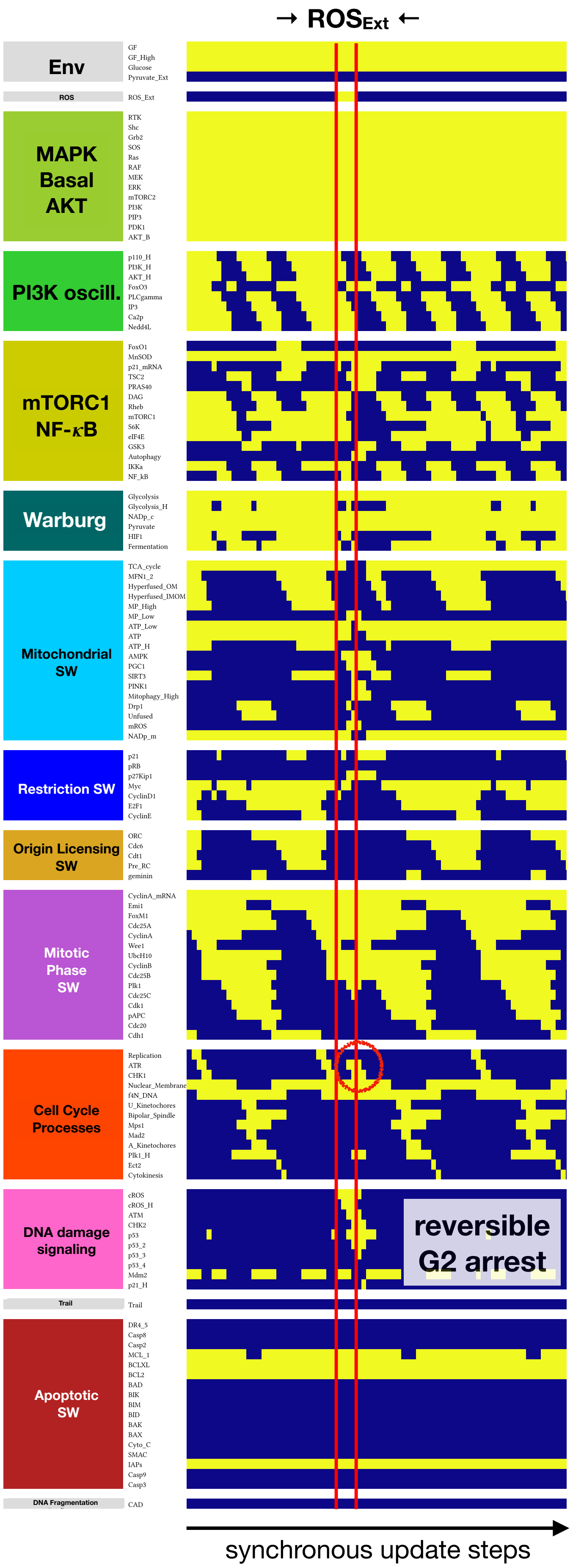

SM Figure 13. Model reproduces reversible G2 arrest in response to brief / weak ROS exposure. Dynamics of regulatory molecule expression/activity in cycling cells exposed to a brief (4 update step) pulse of external ROS. *y-axis:* nodes organized in regulatory modules; *yellow/dark blue:* ON/OFF; *vertical red lines:* start/end of high external ROS; *red oval:* prolonged G2, reversible arrest.

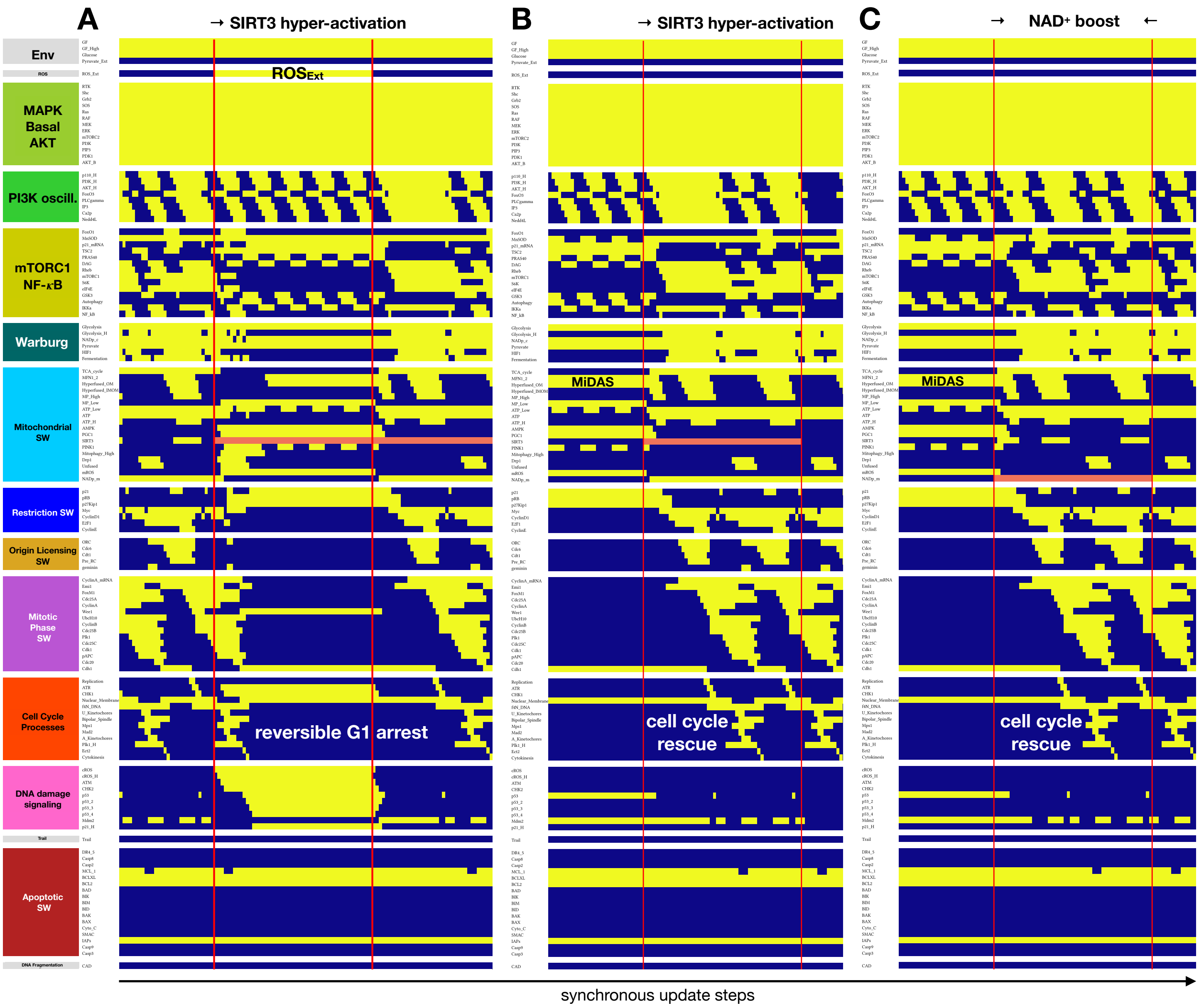

SM Figure 14. Model predicts that *SIRT3* hyper-activation can protect from or reverse ROS-induced MiDAS, and reproduces MiDAS rescue by NAD+ boost. A) Dynamics of regulatory molecule expression/activity in cycling cells exposed to external ROS (50 update steps) at the same time as *SIRT3* hyper-activation. **B-C)** Dynamics of regulatory molecule expression/activity in mitogen-exposed MiDAS cells in response to (B) *SIRT3* hyper-activation (50 update steps); (C) a boost in mitochondrial NAD^+ (50 update steps). *X-axis:* time-steps; *y-axis:* nodes organized in regulatory modules; *yellow/dark blue:* ON/OFF; *pink:* forced activation; *vertical red lines:* start/change in perturbation; *white/black labels:* relevant outcomes.
