# Supplementary material for: Unlocking mitochondrial dysfunction-associated senescence (MiDAS) with NAD+ – A Boolean model of mitochondrial dynamics and cell cycle control
Source: Transl Oncol. 2024 Aug 19;49:102084. doi: 10.1016/j.tranon.2024.102084 (PMC11380032; doi:10.1016/j.tranon.2024.102084)
Supplement: Supplementary file 19 [file mmc19.pdf]

5. Results, section 5 - Context-dependence of MiDAS

<u>Relevant SM Figures:</u>

- **SM Figure 15.** Model produces a heterogeneous mix of cell phenotypes modulated by mitogen, glucose, ROS, and pyruvate exposure.
- **SM Figure 16.** Full repertoire of model attractors organized by the environmental input combinations in which they are stable.

Loss of glucose sends healthy quiescent cells into a state characterized by energy stress with active *AMPK*, low $\Delta\Psi_M$, high mitophagy, and no hyperfusion (**SM Fig. 15**, *bottom plane,* <u>*no pyruvate,*</u> *green arrow* to *yellow-bordered cell*). Cycling cells, on the other hand, generally arrest in a state with reversible mitochondrial hyperfusion, distinguished from MiDAS by *SIRT3* mediated control of ROS paired with NAD^+ maintenance (*red arrow* to *orange-bordered cell;* **Fig. 3A**), Indeed, this state is reversible by glucose exposure — either during cell cycle re-entry (*bidirectional red arrow*), or quiescence.

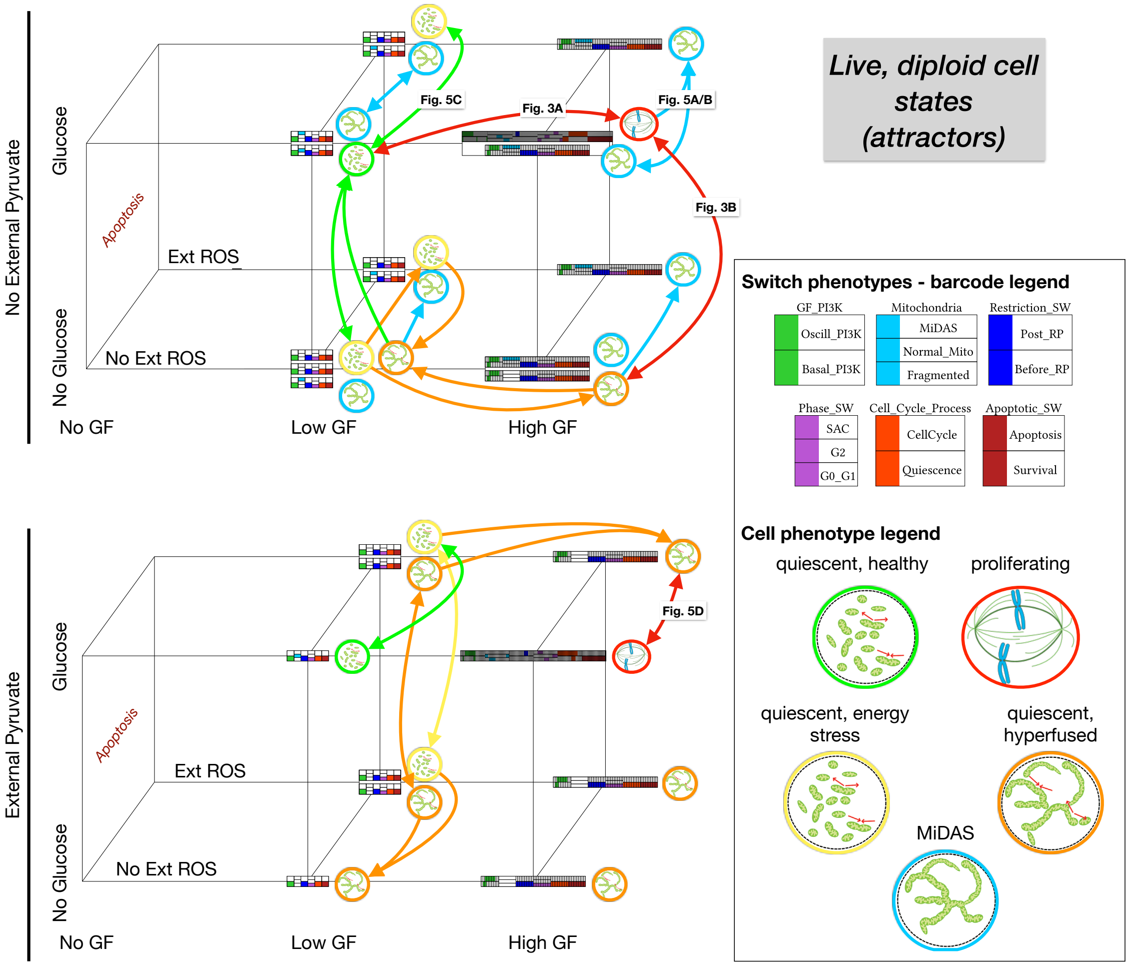

SM Figure 15. Model produces a heterogeneous mix of cell phenotypes modulated by mitogen, glucose, ROS, and pyruvate exposure. A) Summary of model cell states detected in every combination of no/low/high growth-factor (*x axis*), absence/presence of external ROS (*y axis*), low/high glucose (*z axis*), and absence/presence of external pyruvate (*left/right*). Apoptotic and tetraploid quiescent cell states were omitted for clarity (all attractors: **SM Fig. 16**. Barcodes representing each attractor were derived by comparing the expression of nodes in relevant modules to predetermined molecular signatures encoded in the model's *.dmms* file (barcode legend, *bottom left*). Oscillatory phenotypes have expanded barcodes that mark the transitions their regulatory switches undergo during the cycle (*left, high GF area*). Visual summaries of overall cell states indicate the shape and dynamics of the mitochondrial network, distinguish quiescent vs. cycling cells, as well as normal vs. dysfunctional mitochondria (cell phenotype legend, *bottom right*). *Figure labels:* time-courses with molecule-level view of state. *State transition arrows: light blue:* irreversible transition to MiDAS; *green:* reversible transitions from quiescence with healthy mitochondria & transition to it; *orange:* transitions between energy stressed non-MiDAS states; *red:* reversible cell cycle arrest upon glucose withdrawal.

Quiescent cells recovering from ROS exposure in the absence of glucose (**Fig. 15**, *bottom plane, <u>no pyruvate</u>*), get stuck with a hyperfused state that is not quite MiDAS (*orange-outlined cells, bottom plane*), given that subsequent glucose exposure can reestablish healthy mitochondria (*green up-arrow, front plane*). This protection is fragile however, as re-exposure to ROS in this state triggers MiDAS, regardless of growth signals strength (*light blue arrows, bottom plane*). Interestingly, lack of glucose but access to external pyruvate (*bottom plane, <u>pyruvate</u>*) forces this reversible hyperfusion in all but high ROS/low growth factor conditions, and loss of pyruvate in high growth factors triggers MiDAS (*arrow not shown*).

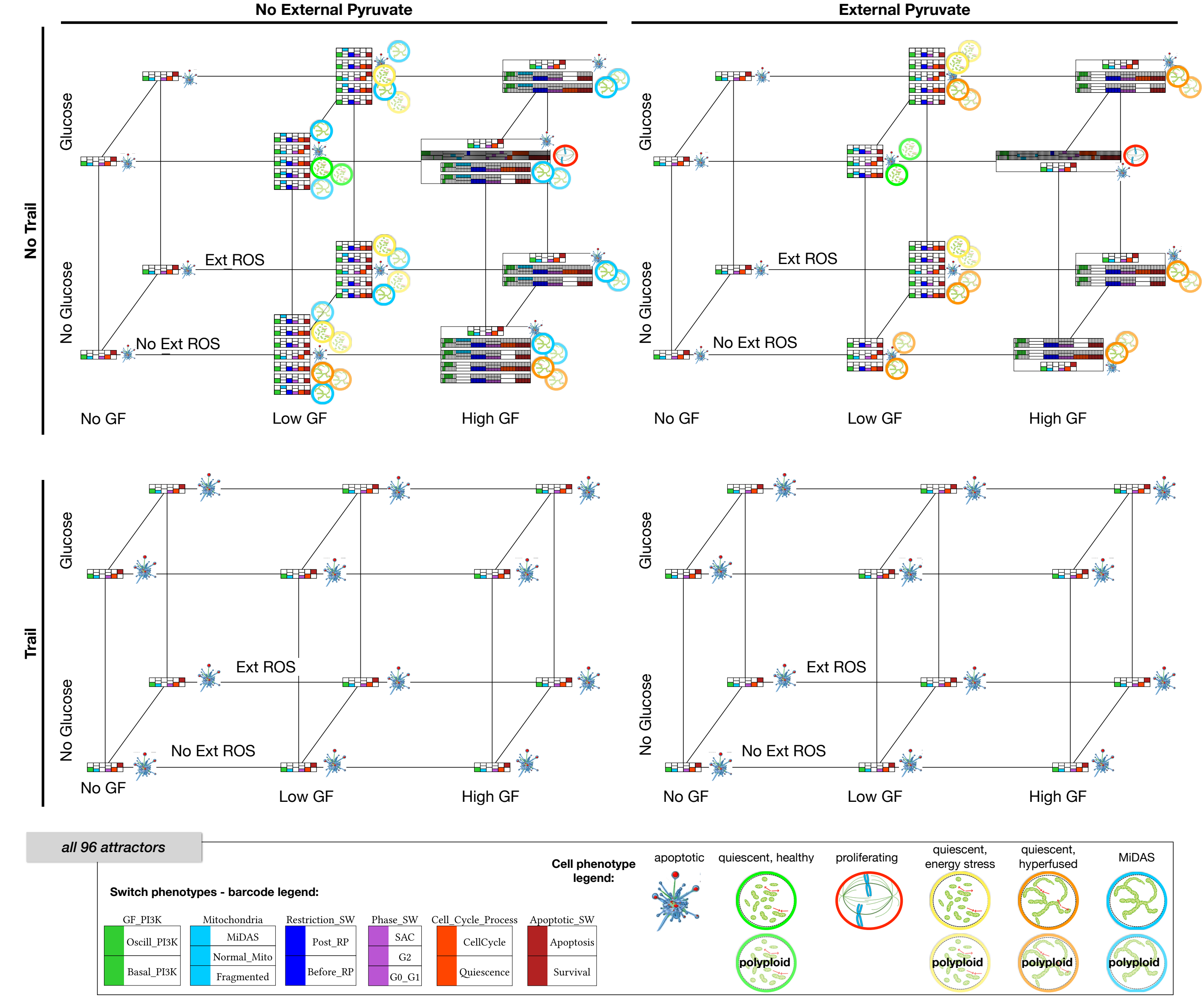

Figure 16. Full repertoire of model attractors organized by the environmental input combinations in which they are stable. A) Model cell states detected in every combination of no/low/high growth-factor (*x axis*), absence/presence of external ROS (*y axis*), low/high glucose (*z axis*), absence/presence of external pyruvate (*left/right*), and absence/presence of Trail (*top/bottom*). Barcodes representing each attractor were derived by comparing the expression of nodes in each relevant module to a pre-determined molecular signature known to represent a cell phenotype (e.g., apoptosis vs. survival) and encoded in the model's *.dmms* file (barcode legend, *bottom left*). Visual summaries of overall cell states indicate the shape and dynamics of the mitochondrial network, distinguish quiescent vs. cycling vs. apoptotic cells, normal vs. dysfunctional vs. fragmented mitochondria, and wild-type vs. tetraploid quiescence (cell phenotype legend, *bottom right*). Molecule-level ON/OFF state of the network in each attractor included in **SM File 7**.

6. Results, section 6 - MiDAS in cells with cancer-related mutations

Relevant SM Figures and Files:

- **SM Figure 17.** *p53* loss blocks MiDAS in response to mild/strong ROS in mitogen-stimulated cells.
- **SM File 14**, included as *MiDAS in cancer-associated mutants.pdf*: Simulation results for all cancer-related mutant cells for every combination of *GF_High*, *Glucose*, and *ROS_Ext* at 0, 5%, 25%, 50%, and 95% saturation with 0% and 50% external pyruvate (100% pyruvate blocked MiDAS, an affect unchanged by any of our perturbations; data not shown but can be generated with commands in **SM File 11**). *Hyper-activated nodes:* $PI3K_H$, Ras, RAF, mTORC1, AKT_H, Myc, HIF1, MEK, Cyclin E, Cyclin D1, ERK, $p21_H$, $p27^{Kip1}$; *knockouts:* SIRT3, p53, ATM, ATR, RB, p21, TSC2, FoxO1, FoxO3, Caspase 8, Caspase 9, Plk1 (results ordered by effect).

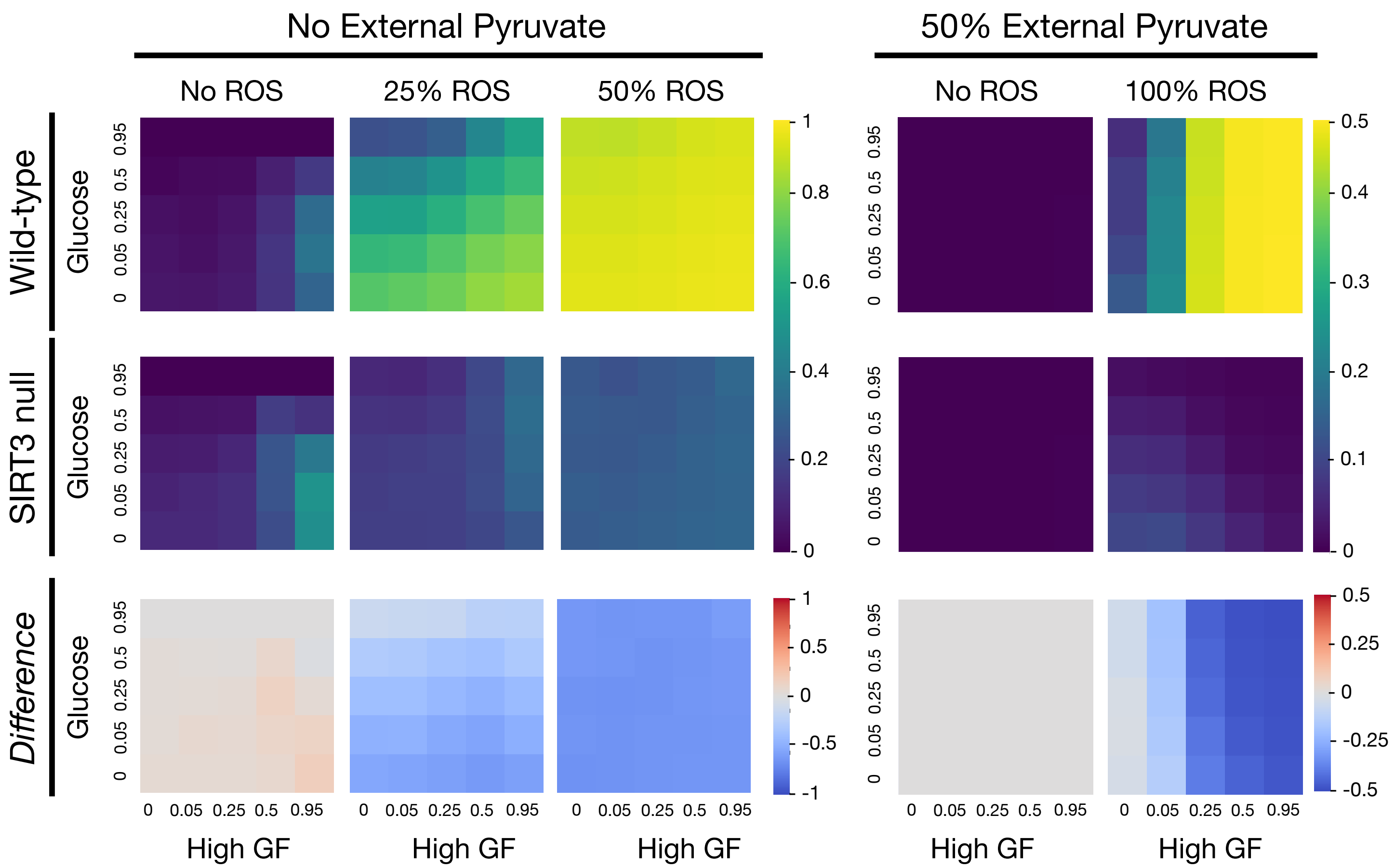

SM Figure 17. ***p53* loss blocks MiDAS in response to mild/strong ROS in mitogen-stimulated cells.** Fraction of time cells spend in a MiDAS state at varying levels of mitogen stimulation (*x* axis) and glucose (*y* axis) with no (0%), mild (5%) or strong (50% / 95%) ROS exposure, in the absence (left 3 columns) vs. presence of 50% external pyruvate (right 2 column). *Top/middle row:* wild-type/*p53* null cells; *bottom row:* increase in the fraction of time spent in MiDAS in *p53*-null cells. *Length of time-window for continuous runs:* 100 steps (10 wild-type cell cycle lengths); *total sampled time:* 500,000 steps; synchronous update; *initial state for sampling runs:* cycling cell in high glucose and no external pyruvate, ROS, or *Trail*.
