# Supplementary material for: Unlocking mitochondrial dysfunction-associated senescence (MiDAS) with NAD+ – A Boolean model of mitochondrial dynamics and cell cycle control
Source: Transl Oncol. 2024 Aug 19;49:102084. doi: 10.1016/j.tranon.2024.102084 (PMC11380032; doi:10.1016/j.tranon.2024.102084)
Supplement: Supplementary file 20 [file mmc20.pdf]

7. Results Reproduced with Biased Asynchronous Update

Relevant SM Figures and Files:

- **SM Figure 18.** PI3K oscillations correspond to complex attractors, while the cell cycle is a metastable oscillation under asynchronous update (asynchronous version of Fig. 3A).
- **SM Figure 19.** Asynchronous version of Fig. 3, ensemble average (model reproduces cell cycle-linked mitochondrial dynamics and G1 arrest in response to glucose withdrawal).
- **SM Figure 20.** Asynchronous version of Fig. 4B,D (model reproduces MiDAS in response to SIRT3 knockdown).
- **SM Figure 21.** Asynchronous version of Fig. 5A-B (model reproduces ROS-induced MiDAS in cycling cells).
- **SM Figure 22.** Asynchronous version of Fig. 5C-D (model predicts protection from MiDAS in quiescence or in external pyruvate).

Comparison of synchronous vs. asynchronous attractors. *Dynmod* detected 96 synchronous attractors: 78 fixed points, 16 cycles involving PI3K oscillations at high growth factor, and two cell cycles (with/without external pyruvate). AEON (https://github.com/sybila/biodivine-aeon-py) [63], the fastest asynchronous attractor detection algorithms we are aware of, is guaranteed to find *all* fixed-point and complex attractors under general asynchronous update for any network it can complete its algorithm on in a reasonable time [67]. This was indeed the case for our model, confirming that the only fixed points and complex attractors of this network match the 78 fixed points and 16 PI3K oscillations found by *dynmod* (**SM Fig. 17A**). We have previously shown that our cell cycle regulatory modules generate a meta-stable circular "valley" in state space akin to a 'Mexican hat' landscape, which can temporarily trap the asynchronous dynamics to mimic the cell cycle (SM. Fig. 5 in [45]). It is not a real trap space however, as there are a small number of update-orders that send the model into an apoptotic fixed point, or in the current model, also into MiDAS. While this occurs too often with random-order asynchronous update to be realistic, the paths the model follows to apoptosis and its 'shortcuts through the ring' mimic known cell cycle errors [45]. By contrast, limit cycles involving oscillations driven by the PI3K module are robust to asynchronous update (see [45] for details).

Robustness of modeling results to asynchronous update. In order to test whether the model can generate qualitatively and thus biologically similar responses to signal under synchronous vs. asynchronous update, we re-ran our simulations with a biased version of random-order asynchronous update. Namely, we randomized the update order of nodes in each time-step, but moved a small, select set nodes to the start and/or end of the update order, depending on their current state (encoded in the .dmms file in the model's *Metadata* block as follows; red is new since [45]):

```
BiasOrderFirst: (Cytokinesis, 0), (Pre_RC, 1), (Replication, 0), (U_Kinetochores, 0),
(A_Kinetochores, 1), (Plk1_H, 1), (CyclinB, 1), (Cdc20, 0)
BiasOrderLast: (Replication, 1), (f4N_DNA, 0), (f4N_DNA, 1), (Ect2, 0), (A_Kinetochores, 0),
(U_Kinetochores, 1), (FoxM1, 1), (CyclinE, 1), (Cdc20, 1), (Plk1_H, 0), (MP_Low, 0)
```

This change prevents a series of unrealistic node orders from occurring, and increases the stability of the metastable cell cycle trap region. The result is a drop in the incidence of cell cycle errors, apoptosis and MiDAS, due to unrealistic breaks in signal propagation. For example, rerunning Fig. 3A with an ensemble of 10,000 independently simulated biased asynchronous update time-courses generates individual cell fate tracks that cycle and return to quiescence (**SM Fig. 17B**), end up as polyploid quiescent cells indicating a cell cycle error (**SM Fig. 17C**), undergo apoptosis (**SM Fig. 17D**), or undergo MiDAS (**SM Fig. 17E**). Yet, the average time course of the entire ensemble (**SM Fig. 18A**), as well as the time spent in each phenotype of relevant switches (**SM Fig. 18B**), indicate that most cells return to quiescence with 2N DNA. As expected from [45], the

relative rate of events that deviate from a healthy cell cycle is lower with biased vs. fully random-order update (**SM Fig. 18C**). Similarly, loss of glucose (asynchronous version of Fig. 3B) results in a reversible G1 arrest with hyperfused mitochondria in the majority of cells (**SM Fig. 18D**), in accordance with Fig. 3B. Yet, the figure also indicates that a small fraction of cells undergoes apoptosis (observed with synchronous update, SM Fig. 6C), or enters MiDAS upon glucose re-exposure.

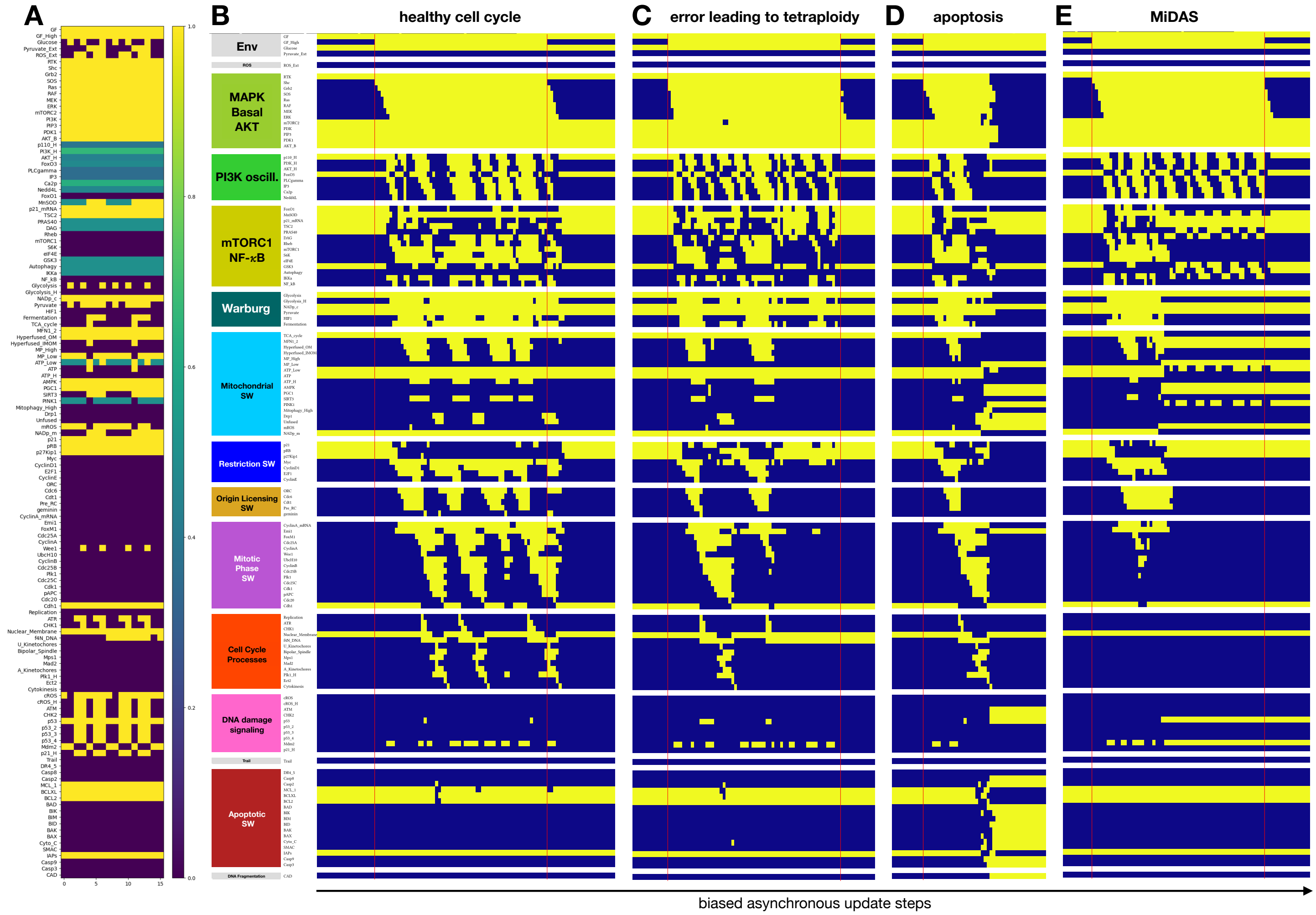

SM Figure 18. PI3K oscillations correspond to complex attractors, while the cell cycle is a metastable oscillation under asynchronous update (asynchronous version of Fig. 3A). A) Regulatory molecule expression/activity (*y axis*) averaged over all network states in the trap space of the 16 complex attractors (*x axis*) detected with AEON. *Yellow*: stable ON; purple: stable OFF; *teal*: toggling ON/OFF. **B-E)** Dynamics of regulatory molecule expression/activity in individual quiescent cells responding to strong growth signals (50 update steps) under biased asynchronous update, showing a heterogeneous mix of cell fates: (B) repeated normal cell cycle and return to quiescence; (C) error leading to polyploid cells that can undergo endo-reduplication; (D) spontaneous mitotic catastrophe and apoptosis; (E) spontaneous MiDAS. *X-axis:* biased random-order asynchronous update-steps; *y-axis:* nodes organized by regulatory modules; *yellow/dark blue:* ON/OFF; *vertical red lines:* start/end of strong growth signal.

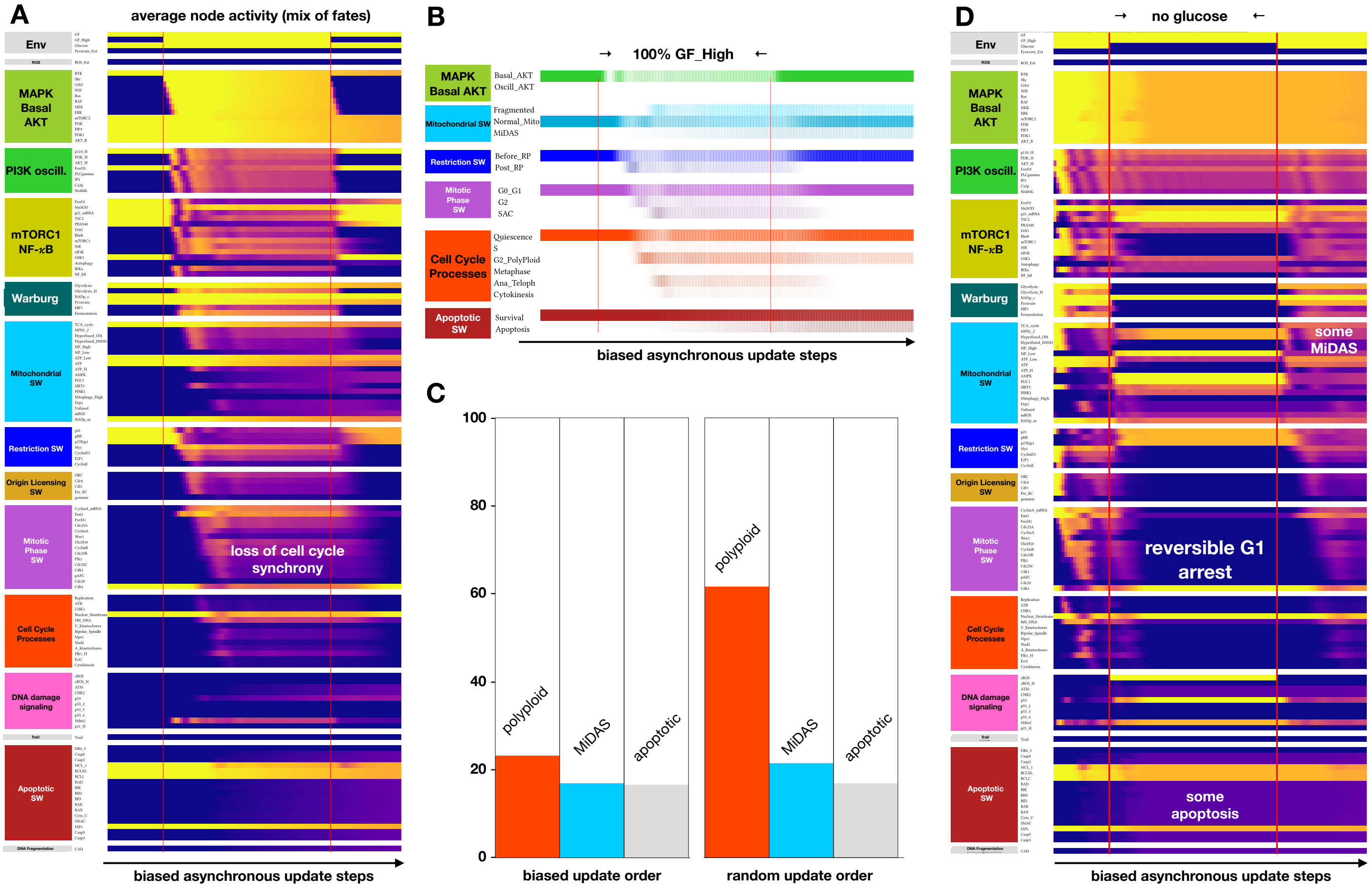

SM Figure 19. Asynchronous version of Fig. 3, ensemble average (model reproduces cell cycle-linked mitochondrial dynamics and G1 arrest in response to glucose withdrawal). A) Dynamics of regulatory molecule expression/activity in an ensemble of 10,000 cells responding to strong growth signals (50 update steps) with cell cycle entry, followed by de-synchronization of cell cycle progression and ~15% apoptosis. *X-axis:* biased random-order asynchronous update-steps; *y-axis:* nodes organized by regulatory modules; *yellow to dark blue:* ON to OFF; *vertical red line:* start/end of strong growth signals; **B)** Average number of cells with an internal state matching each regulatory switch-phenotype, as a function of biased random-order asynchronous update-steps in the simulation on (A). **C)** Average ON state of the 4N DNA node (*red*, marking tetraploid cells), the Hyperfused_OM node (*light blue*, marking MiDAS cells), and the CAD node (*gray*, marking apoptotic cells), averaged over 10,000 cells as well as a 50 time-step window following the removal of strong growth signals (last interval on A). **D)** Dynamics of regulatory molecule expression/activity in an ensemble of 10,000 dividing cells responding to glucose withdrawal (50 update steps) and its reversal, showing reversible cell cycle arrest, as well as a small uptick in apoptosis and MiDAS. *X-axis:* biased random-order asynchronous update-steps; *y-axis:* nodes organized by regulatory modules; *yellow to dark blue:* ON to OFF; *vertical red line:* start/end of glucose withdrawal; *labels:* relevant phenotypic changes.

Our main MiDAS results are generally recapitulated with biased asynchronous update:

— **SM Fig. 20:** asynchronous version of Fig. 4 (SIRT3 knockdown-induced MiDAS + some apoptosis)

— **SM Fig. 21**: asynchronous version of Fig. 5A-B (ROS-induced MiDAS following G1 or G2 arrest + some apoptosis)

— **SM Fig. 22**: asynchronous version of Fig. 5C-D (robust but not complete protection from ROS-induced MiDAS in quiescent cells; near-complete protection in cycling cells exposed to external pyruvate).

— **SM Fig. 23**: asynchronous version of Fig. 6B (context-dependent effect of SIRT3 knockdown).

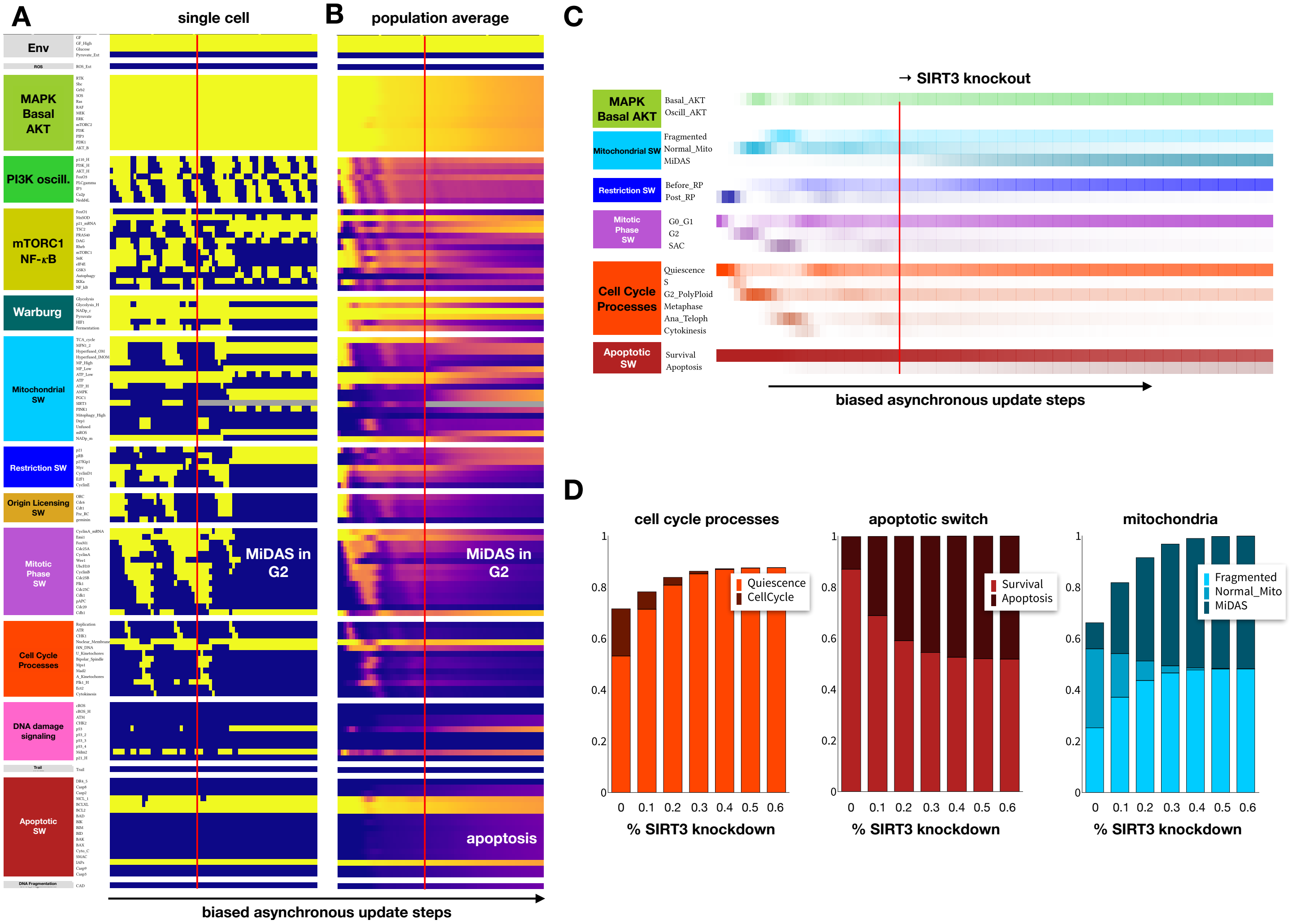

SM Figure 20. Asynchronous version of Fig. 4B,D (model reproduces MiDAS in response to SIRT3 knockdown). **A)** Dynamics of regulatory molecule expression/activity in a single dividing cell responding to full *SIRT3* knockout in early metaphase. **B)** Dynamics of regulatory molecule expression/activity in an ensemble of 10,000 asynchronously dividing cells responding to full *SIRT3* knockout. *X-axis:* biased random-order asynchronous update-steps; *y-axis:* nodes organized by regulatory modules; *yellow/dark blue:* ON/OFF; *vertical red line:* start of *SIRT3* knockout; *labels:* relevant molecular or phenotypic changes in response to *SIRT3* knockout, leading to irreversible G2 arrest and MiDAS with a mix of 2N and 4N DNA content, as well as ~35% apoptosis. **C)** Average number of cells with an internal state matching each regulatory switch-phenotype, as a function of biased random-order asynchronous update-steps in the simulation on (B). **D)** Response of cells dividing in 95% saturating growth stimuli to increasing SIRT3 knockdown, quantified as % time spent in each phenotype (*left:* quiescence vs. in cell cycle; *middle*: alive vs. apoptotic; *right*: fragmented vs. normal mitochondria vs. MiDAS), averaged over 1000 independent runs in each consecutive interval of SIRT3 knockdown (*x* axis), shown as stacked bar charts. *update:* biased asynchronous; *initial state for sampling runs:* cycling cell in high glucose and no external pyruvate, ROS, or *Trail*.

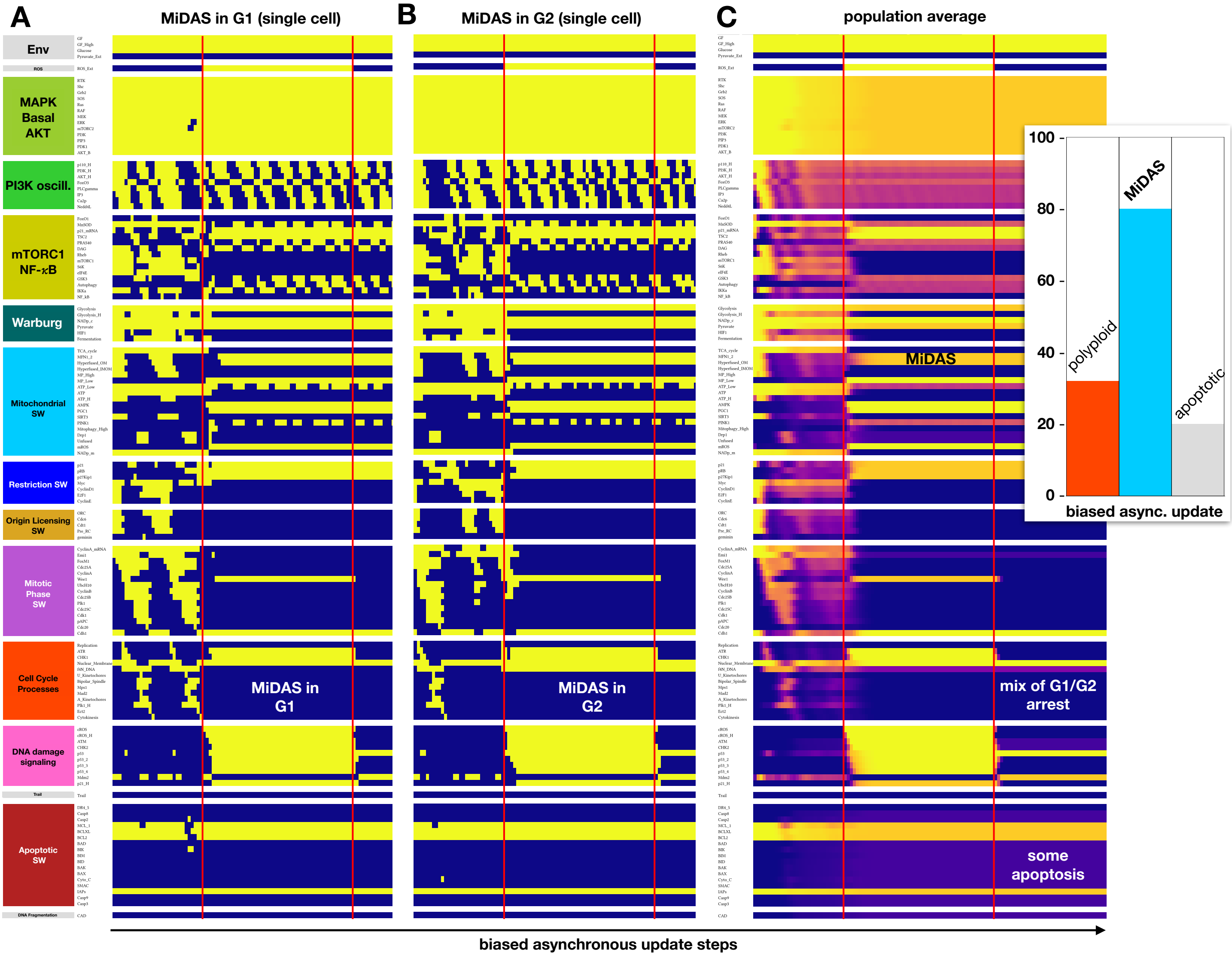

SM Figure 21. Asynchronous version of Fig. 5A-B (model reproduces ROS-induced MiDAS in cycling cells). A-B) Dynamics of regulatory molecule expression/activity during exposure of a cycling cell to external ROS in (A) early G1, leading to irreversible G1 arrest and MiDAS with 2N DNA, vs. (B) early G2, leading to irreversible G2 arrest and MiDAS with 4N DNA. **C)** Dynamics of regulatory molecule expression/activity during exposure of an ensemble of 10,000 asynchronously cycling cells to external ROS, leading to irreversible G1/G2 arrest and MiDAS with a mix of 2N (~65%) and 4N (~35%) DNA content, as well as ~20% apoptosis. *Inset:* average ON state of the 4N DNA node (*red*, marking tetraploid cells), the Hyperfused_OM node (*light blue,* marking MiDAS cells), and the CAD node (*gray*, marking apoptotic cells), averaged over 10,000 cells as well as the 50 time-step window following the removal of external ROS (last interval on C).

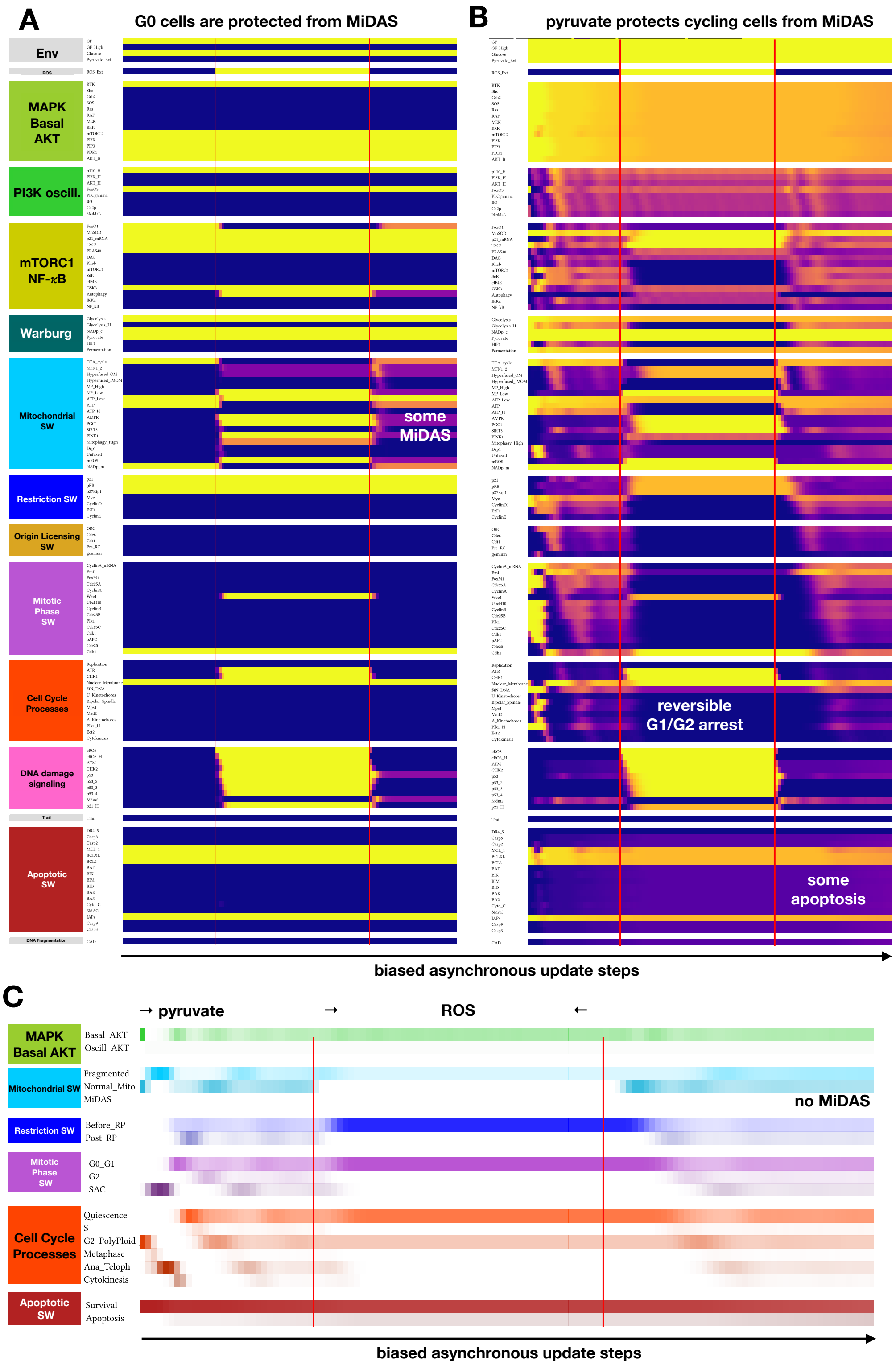

SM Figure 22. Asynchronous version of Fig. 5C-D (model predicts protection from MiDAS in quiescence or in external pyruvate). A) Dynamics of regulatory molecule expression/activity in an ensemble of 10,000 quiescent cells during exposure to external ROS, leading to high mitophagy and short-term hyperfusion to restore ATP levels. **B)** Dynamics of regulatory molecule expression/activity during exposure of an ensemble of 10,000 simulated cycling cells in saturating external pyruvate to external ROS, leading to reversible G1/G2 arrest. *X-axis:* biased random-order asynchronous update-steps; *y-axis:* nodes organized by regulatory modules; *yellow/dark blue:* ON/OFF; *vertical red lines:* start/end of ROS exposure; *white/black labels:* relevant molecular changes or outcomes. **C)** Average number of cells with an internal state matching each regulatory switch-phenotype, as a function of biased random-order asynchronous update-steps in the simulation on (B).

8. Model Behavior in Response to Random Network Errors

Relevant SM Figure:

- **SM Figure 23.** Cell cycle and MiDAS in response to SIRT3 knockdown are robust to random mutations and/ or errors in model construction.

We tested the model's robustness to structural errors by generating three distinct ensembles of random mutants:

— **node knockout / hyper-activation:** involved locking a number n_{Node} of random nodes ON or OFF;

— **link knockout:** removing a number n_{Link} of randomly chosen links;

— **gate error:** introducing a number n_{Gate} of random errors in gate output by flipping the expected output of a node for a single combination of inputs.

Averaging the dynamical behavior of the resulting mutant ensembles in a simulation that started with dividing cells, then entered MiDAS due to the loss of SIRT3, allowed us to probe the network's structural robustness during cell cycle (before SIRT3 knockdown) as well as its ability to enter / maintain MiDAS (after SIRT3 knockdown). In other words, we tested how Fig. 4B would change in our mutant ensembles. **SM Fig. 24A** indicates that our model can tolerate 1-2 full node knockout / hyper-activation mutations/cell before both the cell cycle and MiDAS from cell cycle becomes hard to detect in a population. It tolerates link removals best, starting to loose both cycling cells and the MiDAS phenotype by 15 link removals/ cell (**SM. Fig. 24B**). Altering the output of gates is similar but more potent; by the time 15 random nodes are responding incorrectly to a single combination of their inputs, the ensemble looses most of its cycling cells, some apoptosis appears and most cells show energy stress with active AMPK (**SM Fig. 24C**).

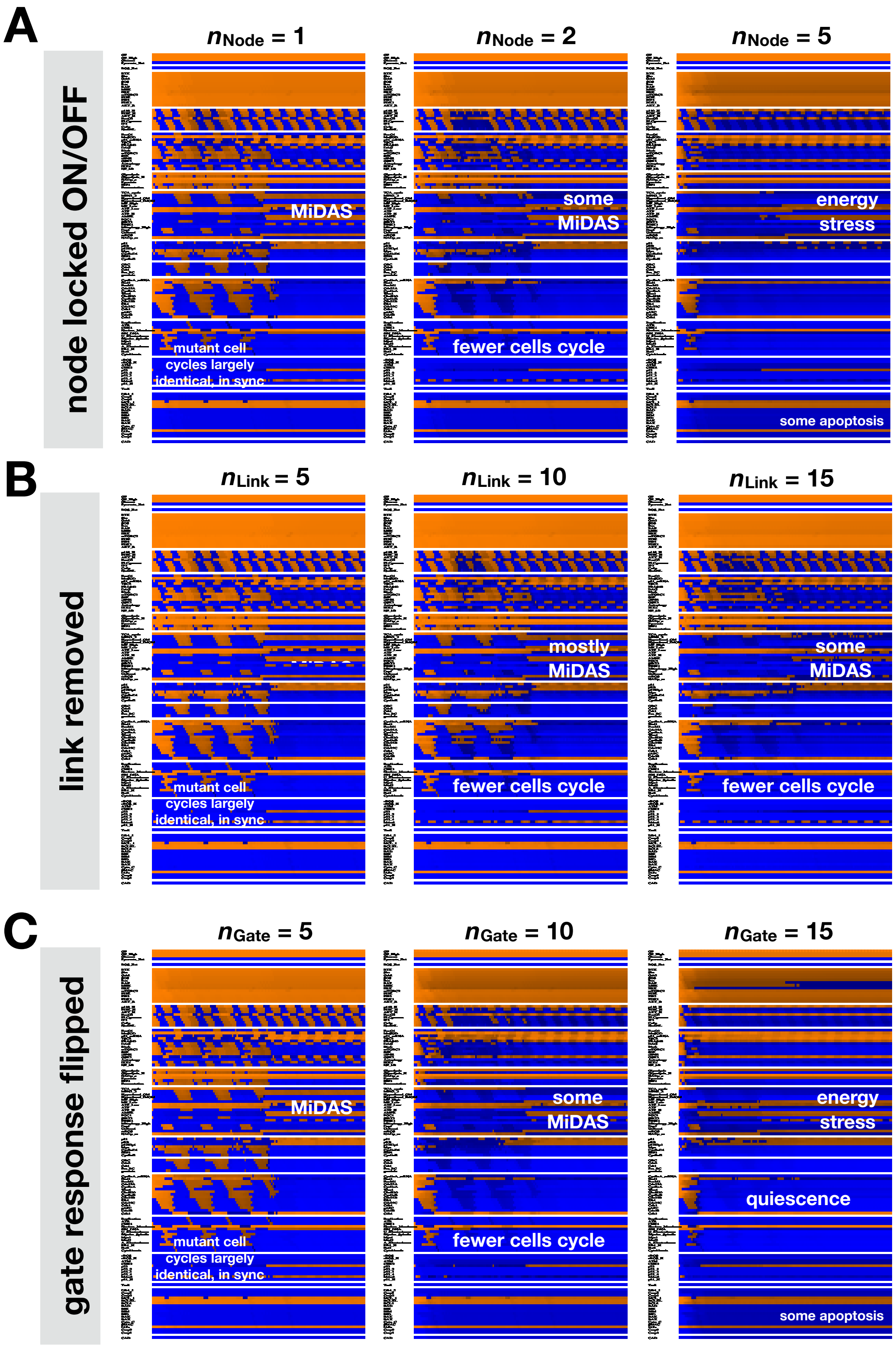

SM Figure 23. Cell cycle and MiDAS in response to SIRT3 knockdown are robust to random mutations and/or errors in model construction. Synchronous dynamics of regulatory molecule expression/activity during SIRT3 knockdown in an ensemble of growth-stimulated, cycling cells, averaged over 1000 distinct mutant networks with **A)** $n_{Node} \in \{1,2,5\}$ random nodes per network locked ON or OFF; **B)** $n_{Link} \in \{2,5,15\}$ random links per network removed; **C)** $n_{Gate} \in \{2,5,15\}$ random gate outputs per network flipped. *X-axis*: synchronous update steps; *y-axis*: nodes organized in regulatory modules; *orange/black/blue color-scale*: average expression of each molecule across 1000 time-courses from independently generated mutant models (orange = all ON; black = 50% ON/OFF; blue = all OFF); synchronous update; black/white labels: relevant molecular patterns.
